# Mechanistic Investigation of the Nickel-Catalyzed Transfer Hydrocyanation of Alkynes

**DOI:** 10.1021/acscatal.3c02977

**Published:** 2023-08-16

**Authors:** Julia C. Reisenbauer, Patrick Finkelstein, Marc-Olivier Ebert, Bill Morandi

**Affiliations:** ETH Zürich, Vladimir-Prelog-Weg 3, HCI, 8093 Zürich, Switzerland

**Keywords:** nickel catalysis, transfer hydrocyanation, shuttle catalysis, aliphatic nitrile activation, mechanistic study, Lewis acid-free

## Abstract

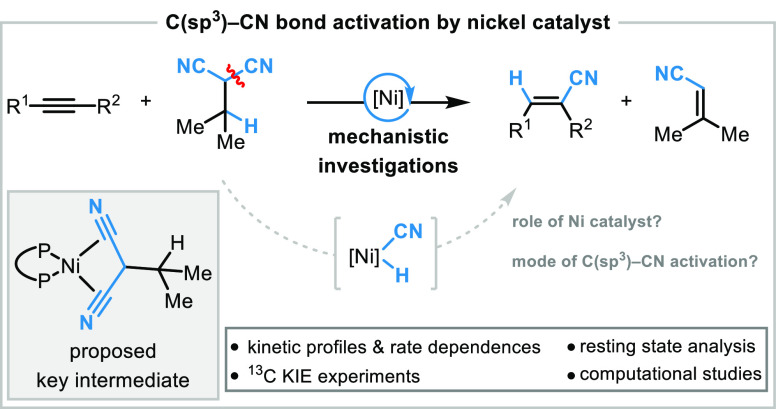

The implementation of HCN-free transfer hydrocyanation reactions on laboratory scales has recently been achieved by using HCN donor reagents under nickel- and Lewis acid co-catalysis. More recently, malononitrile-based HCN donor reagents were shown to undergo the C(sp^3^)–CN bond activation by the nickel catalyst in the absence of Lewis acids. However, there is a lack of detailed mechanistic understanding of the challenging C(sp^3^)–CN bond cleavage step. In this work, in-depth kinetic and computational studies using alkynes as substrates were used to elucidate the overall reaction mechanism of this transfer hydrocyanation, with a particular focus on the activation of the C(sp^3^)–CN bond to generate the active H–Ni–CN transfer hydrocyanation catalyst. Comparisons of experimentally and computationally derived ^13^C kinetic isotope effect data support a direct oxidative addition mechanism of the nickel catalyst into the C(sp^3^)–CN bond facilitated by the coordination of the second nitrile group to the nickel catalyst.

## Introduction

In the last decades, many powerful transition-metal catalyzed approaches have been developed to activate and transform covalent C–C bonds to facilitate synthetically useful transformations.^1−3^ However, mechanistic studies of catalytic C–C bond activation reactions remain scarce, particularly with regard to understanding the key factors enabling the C–C bond activation step. This stands in contrast to other fields of strong bond activation, such as C–H bond functionalization, where important mechanistic studies have opened new opportunities for these reactions.^4,5^

**Figure 1:**
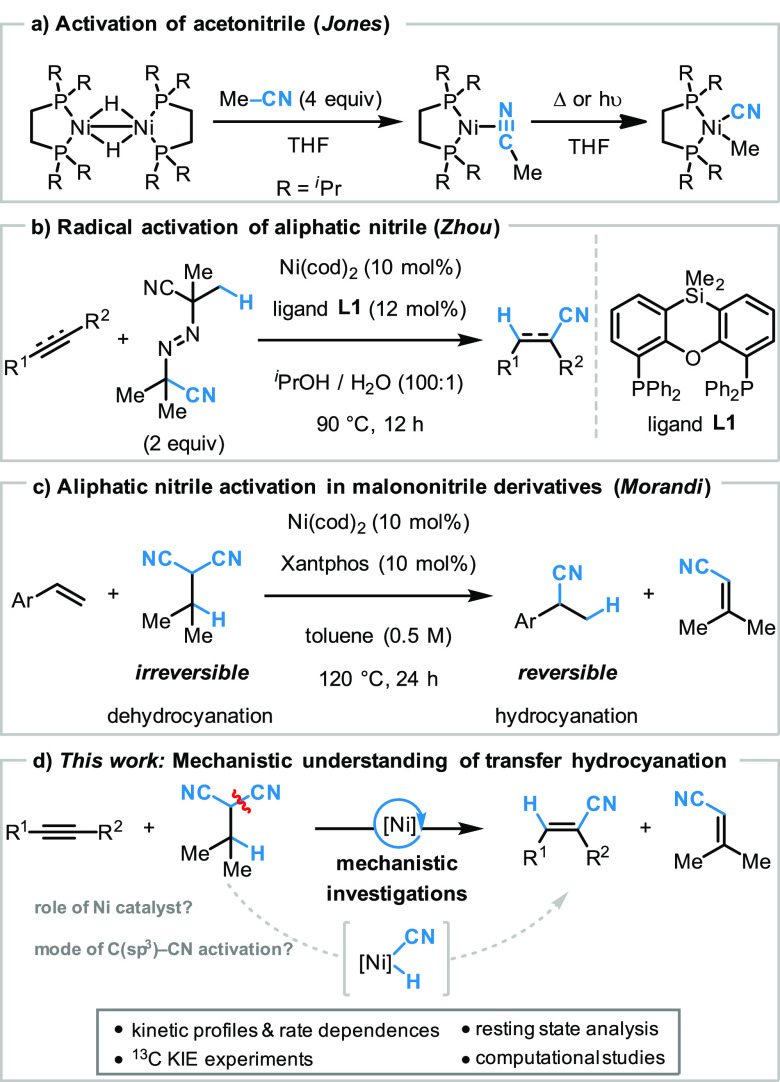
Overview of C(sp^3^)–CN bond activation of aliphatic nitriles and transfer by nickel complexes without the need for a Lewis acid preactivation.

The activation of C–CN bonds has been of interest due to the abundance of nitrile groups in commodity and pharmaceutically relevant compounds and their versatility to act as suitable precursors for a plethora of other functional groups.^6−9^ Initial reports showcasing the ability of low valent Ni(0) precursors^10−14^ to activate nitrile groups facilitated the development of a variety of aryl cyanation reactions.^15−24^ Based on these findings, further investigations towards the more challenging activation of alkyl nitriles showed that C(sp^3^)–CN bonds can be effectively activated by phosphine-supported nickel catalysts in combination with co-catalytic amounts of group 13-based Lewis acids, such as AlCl_3_.^6,15,25^ This dual catalytic system was also harnessed by our group in the development of a catalytic transfer hydrocyanation reaction.^26^ In contrast, nickel-mediated alkyl nitrile activation in the absence of Lewis acids has remained scarce and is still lacking mechanistic understanding.^27,28^ Pioneering work by the Jones group showcased that the C–CN bond in acetonitrile can be activated by Ni(0) precatalysts under thermal or photocatalytic conditions (Figure 1a); however, attempts to further extend this approach toward other alkyl nitriles remained unsuccessful.^29,30^

More recently, a transfer hydrocyanation approach relying on a proposed radical relay mechanism was developed by Fan and Zhou employing AIBN as the HCN donor (Figure 1b).^31^ Besides the use of AIBN, malononitrile-derived sacrificial HCN donor reagents have also been shown to efficiently transfer HCN to styrene derivatives to access the thermodynamically less stable, benzylic nitrile products under nickel catalysis (Figure 1c).^32^ This transfer hydrocyanation approach implemented by our group solely relies on nickel catalysis without the requirement of Lewis acid preactivation of the nitrile group, thus enabling a kinetically selective protocol to access the thermodynamically less stable branched hydrocyanation products and significantly increasing functional group tolerance. Key to the development of this Lewis acid-free protocol was the donation of HCN from a malononitrile-based donor reagent to the nickel catalyst to generate the active H–Ni–CN species. The HCN transfer from the donor to the nickel catalyst generates a stable, conjugated vinyl nitrile byproduct acting as a thermodynamic sink. However, the mode of activation of the C–CN bond in the malononitrile donor reagent by the nickel catalyst has not been studied so far. A better understanding of the underlying factors enabling the C(sp^3^)–CN bond activation in the absence of co-catalytic Lewis acid is crucial as it might lead to the discovery of new general principles for the design of other C–C bond activation reactions.

Herein, we report a catalytic transfer hydrocyanation of alkynes using malononitrile-based HCN donor reagents under nickel catalysis (Figure 1d). A combination of kinetic, organometallic, and computational studies was performed to elucidate the underlying mechanism of the overall transformation. This led to key mechanistic insights, particularly with regard to the challenging C(sp^3^)–CN bond activation in the privileged malononitrile-based donor reagents.

## Results and Discussion

### Development of the Transfer Hydrocyanation of Alkynes

**Figure 2:**
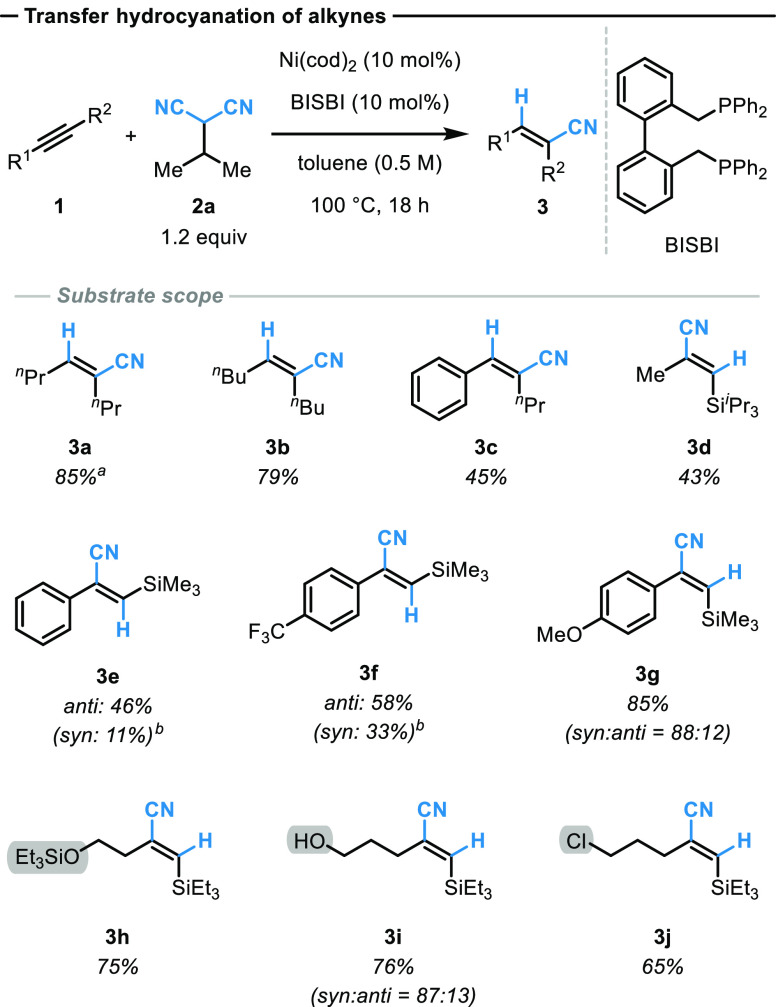
Substrate scope of the transfer hydrocyanation of alkynes in the absence of co-catalytic Lewis acid on a 0.50-mmol scale. ^a^Reaction was run on a 3.0-mmol scale. ^b^Isomers were isolated separately.

Our previous interest in transfer hydrocyanation reactions has sparked the development of different hydrocyanation protocols.^26,33^ Malononitrile-based HCN donor reagents were shown to enable the hydrocyanation of styrene derivatives to access the thermodynamically less favorable, branched hydrocyanation products selectively.^32^ Due to the reversibility of this reaction regarding the hydrocyanation products, we first wanted to establish a related, overall irreversible transformation relying on the implementation of malononitrile-based donor reagents to mechanistically evaluate the donor-nickel interaction. In this context, the transfer hydrocyanation of alkynes to access the corresponding alkenyl nitriles would be an ideal model reaction as competitive H–Ni–CN formation resulting from the dehydrocyanation of the alkenyl nitrile would be negligible. Initial test reactions revealed that Ni(cod)_2_ in combination with bisphosphine ligands, such as 2,2′-bis(diphenylphosphinomethyl)-1,1′-biphenyl (BISBI), is competent to catalyze the desired transformation using 2-isopropylmalononitrile **2a** as the HCN donor (Figure 2). After further optimization of the reaction conditions (for more details see Supporting Information), the best catalytic system was identified as 10 mol % of Ni(cod)_2_/BISBI in combination with 1.2 equiv of the HCN donor **2a** and toluene as the solvent system at 100 °C. We next evaluated the functional group tolerance of the method to unravel key differences with regard to the previously developed alkyne transfer hydrocyanation reaction under Lewis acid activation. Both 4-octyne and 5-decyne were transformed into the corresponding transfer hydrocyanation products **3a** and **3b** in good yields. Varying the steric bulk and electronics by either installing a phenyl or an isopropylsilyl next to the alkyne led to a decrease in yield, affording the hydrocyanation products **3c** and **3d** in 45 and 43% yield, respectively. For substrates **1a–d**, the *syn*-addition products were obtained exclusively; however, when a 1,2-disubstituted phenyl trimethylsilyl alkyne was subjected to the standard reaction conditions both the corresponding *anti*- and *syn*-addition products **3e** and **3e’** were obtained in 46 and 11% yield, respectively. Interestingly, an electron-withdrawing CF_3_ group on the phenyl ring also led to the predominant formation of the *anti*-addition product **3f** (58% yield), while the corresponding *syn*-addition product **3f’** was isolated in 33% yield. However, when an electron-donating methoxy group was installed, the *syn*-addition hydrocyanation product **3g** was obtained as the major isomer in high yield (75%), while only minor amounts of the *anti*-addition product were formed (10%). Most likely, the *anti*-addition products are obtained after *E*/*Z* isomerization of the intermediate alkenyl-nickel species.^34,35^ Showcasing the method’s compatibility with Lewis acid-sensitive functional groups that cannot be tolerated with other protocols, a silyl-ether, an unprotected primary alcohol, as well as a primary alkyl chloride was tolerated under the optimized reaction conditions affording the corresponding products **3h**, **3i**, and **3j** in 75, 76, and 65% yield respectively. In all cases, excellent regioselectivity was observed.

### Labeling Studies

**Figure 3:**
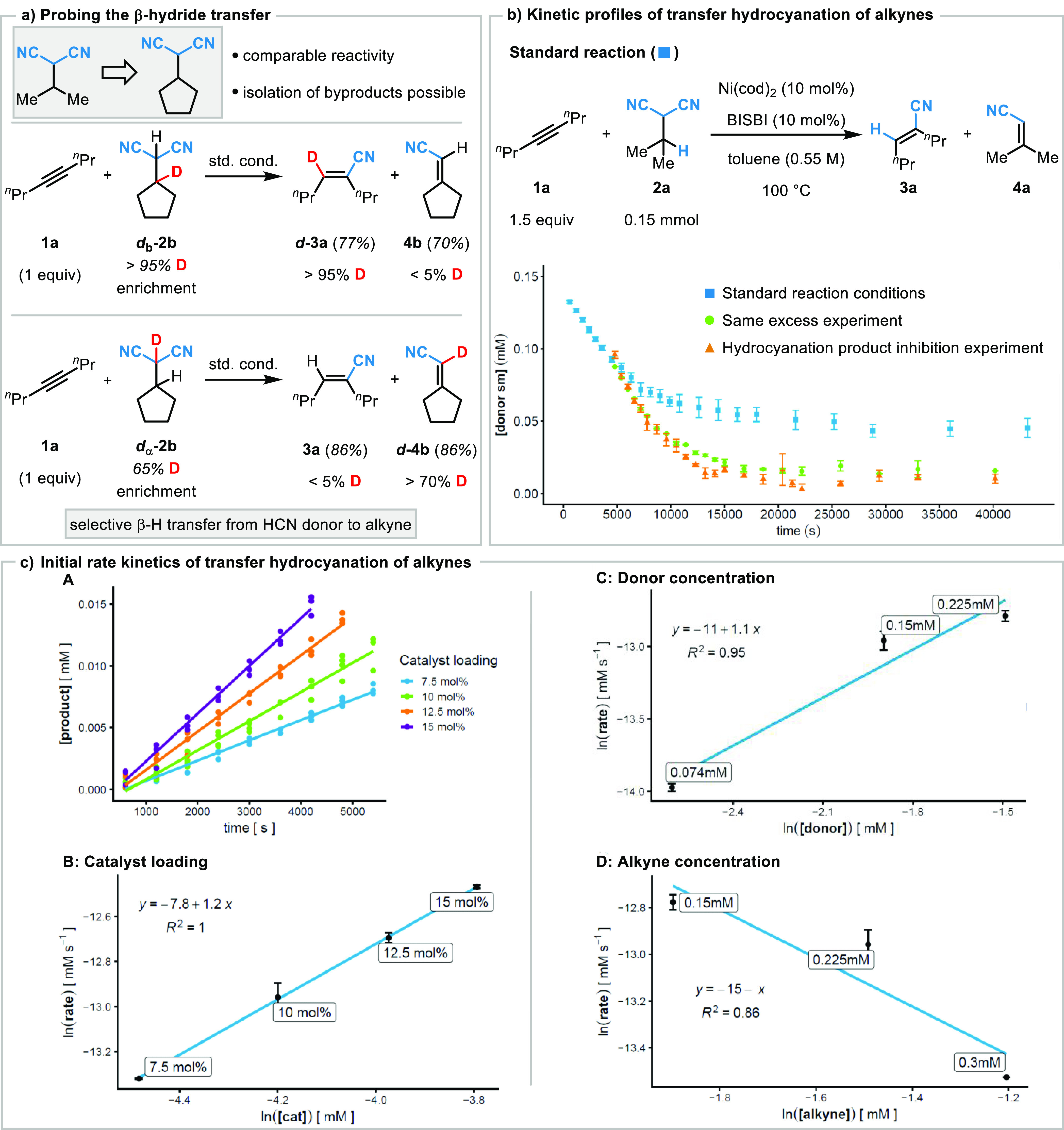
(a) Probing the origin of β-hydride transfer from the sacrificial HCN donor to the metal catalyst in the transfer hydrocyanation of alkynes. (b) Time-adjusted kinetic profiles of the transfer hydrocyanation reaction. The kinetic data are depicted for the standard reaction conditions (blue square), the same excess experiment, assuming 30% donor **2a** conversion at the start (green circle), and product inhibition experiment with additional 30% product **3a** at the start (orange triangle). (c) Initial rate kinetics based on different catalyst loadings (A, B), donor concentrations (C), and alkyne concentrations (D). Measurements were performed in duplicate, triplicates, or quadruplicates and are represented by the mean and the corresponding error bars representing the standard deviation of the mean (for more details see Supporting Information).

Having a robust protocol for the alkyne transfer hydrocyanation in hand, we next aimed to perform key mechanistic experiments to unravel the mechanistic intricacies of the system, particularly with regards to the key C–CN activation step mediated by the nickel catalyst in the absence of co-activating Lewis acid. The origin of the vinylic hydrogen atom in the hydrocyanation product was probed to confirm that the HCN is transferred from the malononitrile-based donor reagent (Figure 3a).

Initially, we proposed that a β-hydride elimination after C–CN bond activation from the sacrificial malononitrile-based donor reagent and subsequent HCN transfer to the alkyne can occur. To test this hypothesis, deuterium-labeled donors were used. We chose the deuterated form ***d*_β_-2b** of 2-cyclopentylmalononitrile donor **2b**. While exhibiting comparable reactivity towards HCN transfer, this reagent is however less volatile,^32^ making the investigation and isolation of generated byproducts more feasible. As expected, almost exclusive transfer of the β-deuterium atom was observed, and the corresponding product ***d*-3a** was isolated in 77% yield with more than 90% deuterium incorporation (Figure 3a). In contrast, the incorporation of an α-deuterium labeled donor molecule ***d*_α_-2b** yielded the hydrocyanation product **3a** with almost no deuterium enrichment. These experiments clearly show that the H atom in the product exclusively originates from the β-hydrogen of the donor reagent.

### Kinetics of the Transfer Hydrocyanation of Alkynes

**Figure 4:**
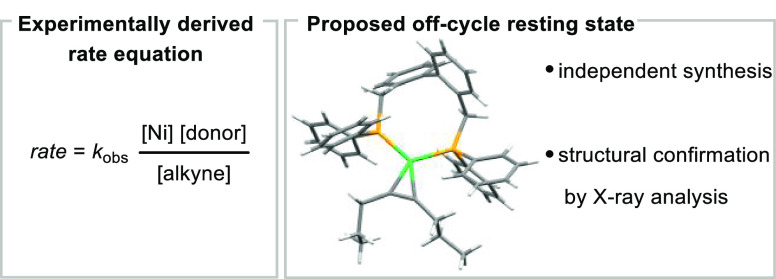
Experimentally derived rate equation and proposed off-cycle resting state.

To gain a better mechanistic understanding of the transfer hydrocyanation process, kinetic data were collected to evaluate the overall catalyst robustness during the transfer hydrocyanation reaction of alkynes.^36^ The reaction progress was monitored for 12 h under the standard reaction conditions (Figure 3b). To assess the catalyst robustness, the reaction kinetics were investigated under same excess conditions assuming 30% conversion of the donor substrate at the beginning while keeping the catalyst concentration the same.^37^ The time-adjusted kinetic profiles of both the standard and same excess experiment diverge, which is diagnostic for either catalyst deactivation or product inhibition. To distinguish between these two scenarios, the kinetics of the same excess conditions were reevaluated under product inhibition conditions as 30% of the hydrocyanation product **3a** was added at the start of the reaction. The kinetic profiles of both the same excess and the product inhibition experiment overlay well, suggesting catalyst deactivation over the course of the reaction. We thus continued our study using the methods of initial rates to determine the experimental rate law (Figure 3c). By varying the concentration of either the catalyst (Ni(cod)_2_/BISBI), alkyne **1a**, or donor **2a**, an approximate first-order dependence on the catalyst concentration and a first-order dependence on the HCN donor **2a** concentration was observed (for more details see Supporting Information). In contrast, the initial rate showed an inverse first-order dependence on the concentration in alkyne **1a**. Based on these results, the experimental rate law was derived (Figure 4).

### Elucidating the Catalyst Resting State of the Transfer Hydrocyanation

Due to our experimentally derived rate law, we hypothesized that decoordination of an alkyne molecule and coordination of the donor reagent from a putative off-cycle Ni(0) resting state could explain the observed orders in reagents. This proposed [(BISBI)Ni(alkyne)] resting state complex was independently synthesized, and its identity was further verified by single-crystal X-ray analysis (Figure 4). NMR-experiments showed that an equilibrium between the [(BISBI)Ni(alkyne)] and the [(BISBI)Ni(donor)] complexes was observed at room temperature, with the alkyne complex being favored, a result consistent with the former being the resting state under catalytic conditions (for more details see Supporting Information). In a subsequent experiment, the reaction was investigated under catalytic conditions by *in situ* VT-NMR analysis. The sample was initially heated at 80 °C for 2 h and then at 100 °C over 4 h, and ^1^H- and ^31^P{^1^H}-NMR spectra were recorded. Besides the formation of the hydrocyanation product **3a**, as well as the byproduct **4a**, the presence of a constant amount of the [(BISBI)Ni(alkyne)] species was also confirmed in the ^31^P{^1^H}-NMR spectra, suggesting that the [(BISBI)Ni(alkyne)] complex is the resting-state of the catalytic reaction.^38^ Notably, the [(BISBI)Ni(donor)] complex could not be observed under these catalytic reaction conditions (for more details see Supporting Information).

### Elucidating whether β-Hydride Transfer Is the Rate-Determining Step

**Figure 5:**
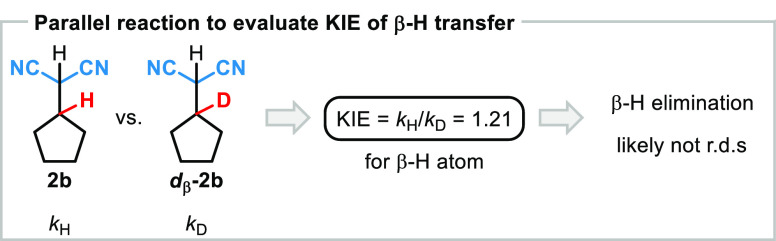
Determining the KIE for β-hydride transfer from the sacrificial HCN donor to the metal catalyst.

From the experimentally derived rate law and the observation that the ligand exchange occurs at room temperature, we proposed that either the nitrile activation or the β-hydride transfer could be turnover limiting, thus requiring additional experiments to differentiate between these two kinetically possible scenarios. To investigate whether the β-hydride elimination is the rate-determining step of the reaction, parallel experiments with isotopically labeled substrates were performed to determine the relative rates for this elementary step, comparing initial rate kinetics for both **2b** and ***d*_β_-2b** donor reagents (for more details see Supporting Information).^39^ A secondary KIE of 1.21 was observed for the β-hydride transfer, suggesting that this step is most likely not the rate-determining step of the reaction (Figure 5, for more details see Supporting Information).^40,41^

### Kinetic Isotope Effect of the ^13^C at Natural Abundance

Based on the experimentally derived rate law, the identified resting state of the reaction, and the small KIE for the β-hydride elimination, we reasoned that the nitrile activation step is most likely the rate-determining step of the reaction. This would be consistent with the usual challenges encountered in activating strong C–C bonds,^42^ particularly in the absence of any Lewis acid assistance. To support this hypothesis experimentally, we determined the ^13^C KIE at natural abundance for the donor molecule using quantitative ^13^C{^1^H}-NMR analysis, in accordance with the method introduced by Singleton.^43^ If the C–CN bond activation of the malononitrile-based reagent is the rate-determining step of the reaction, donors containing ^13^C-carbons in the α-position and/or the nitrile would react more slowly compared to more naturally abundant ^12^C atoms. Thus, fractional enrichment at these two carbon positions in the donor substrate **2a** would be observed, resulting from a large primary ^13^C KIE.^44^

**Figure 6:**
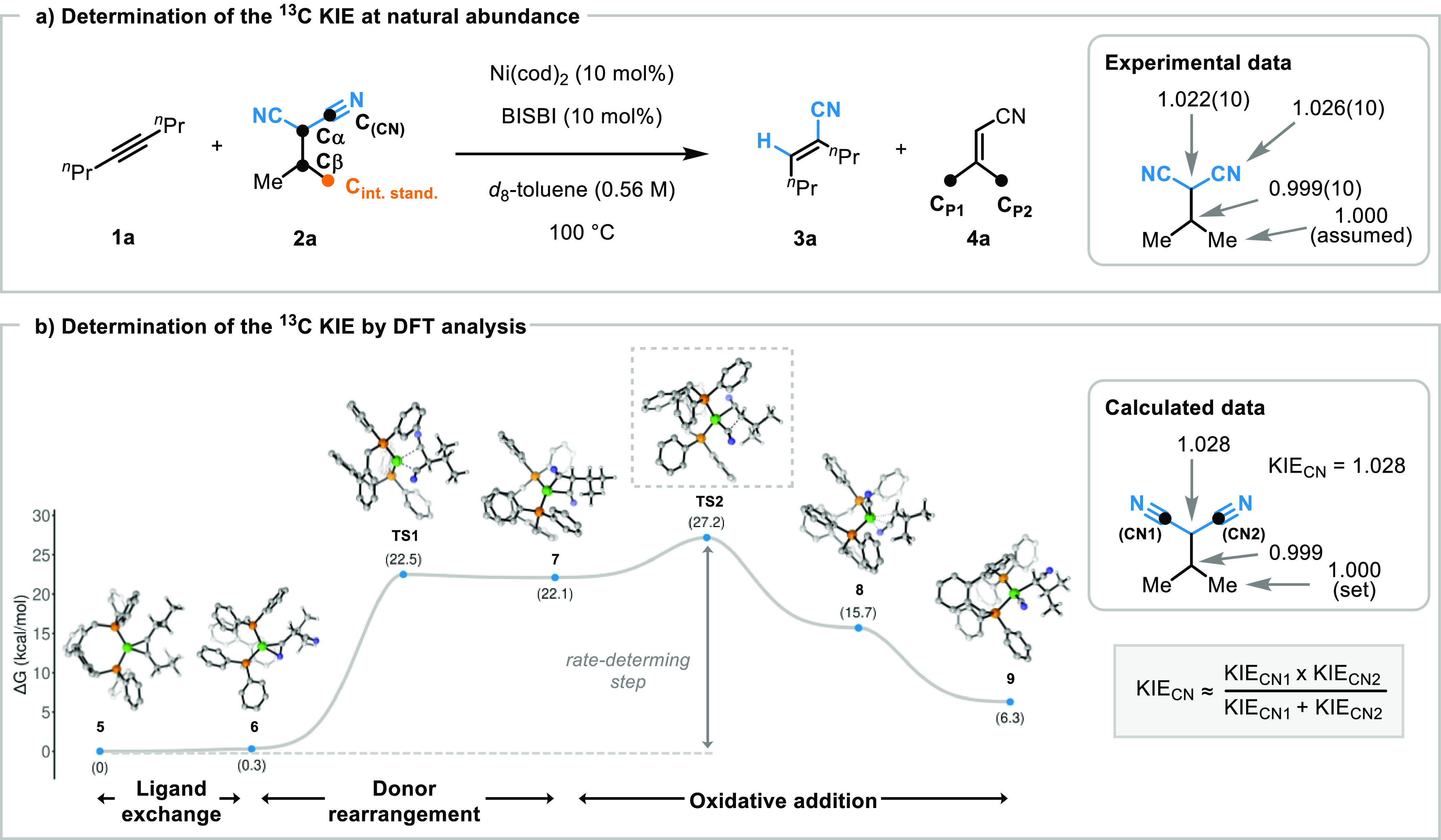
Probing ^13^C KIE at natural abundance to support nitrile activation as the rate-determining step of the transfer hydrocyanation of alkynes. (a) Experimentally derived ^13^C KIEs obtained from four independent runs. Values in brackets correspond to standard deviation. (b) Geometry optimization and frequency calculations were run with PBE0-D3BJ def2-TZVP(Ni)/def2-SVP (other atoms), solvation effects were included using cpcm (toluene), and the frequency temperature was set to 373.15 K. Single point energies were run with PBE0-D3BJ def2-QZVP(Ni)/def2-TZVP (other atoms), and solvation effects were included using cpcm (toluene) (for more details see Supporting Information). The plot was generated with EveRplot.^48^

We investigated the standard reaction by running four independent reactions to moderate or high conversion (58–83%). The integrals of the donor **2a** before the reaction were then compared to the integrals of the remaining donor substrate after the reaction, relying on quantitative ^13^C{^1^H}-NMR techniques (for more details see Supporting Information). A significant primary KIE for both the α-carbon (1.022 ± 0.010) and the nitrile groups (1.026 ± 0.010) was observed (Figure 6a). This clear primary kinetic isotope effect further supports the nitrile activation step as being rate-determining.

To further investigate the mode of the C–CN bond activation, computational modeling was performed to assess the energy profile of the reaction and compare experimental and theoretical ^13^C KIEs (Figure 6b, for more details see Supporting Information).^45^ In accordance with the experimentally observed resting state, these calculations suggest that the [(BISBI)Ni(alkyne)] **5** complex is likely the lowest energy intermediate prior to the rate-determining step. This is followed by decoordination of the alkyne and coordination of the donor resulting in the formation of the [(BISBI)Ni(donor)] complex **6**, which is only slightly uphill in energy. The experimental NMR studies support a larger energy difference for these two species, which is not adequately captured at this level of theory. To achieve the C–CN bond activation, this nickel intermediate rearranges into a tetrahedral geometry forming complex **7** featuring coordination to both nitrile groups. Interestingly, this nitrile directing effect through side-on coordination has also been occasionally proposed in C–H functionalization.^46^ This arrangement then primes the complex for the oxidative addition of one of the nitriles, with an energetically accessible barrier of Δ*G*^TS^ = 27.2 kcal/mol (**TS2**), leading to intermediate **8** featuring an allyl-like coordination of the vinyl-nitrile, which can rearrange to the square planar [(BISBI)Ni(alkyl)(CN)] **9**. The barrier of Δ*G*^TS^ = 27.2 kcal/mol for the oxidative addition of donor **2a** is significantly lower than the Δ*G*^TS^ = 40.3 kcal/mol calculated for the C–CN bond activation in mononitriles such as isovaleronitrile (for more details see Supporting Information),^47^ which explains why the C–CN bond activation can proceed without Lewis acid preactivation. Based on this computed reaction coordinate, the largest energy difference between the resting state and a high energy transition state along the reaction coordinate is featured between the [(BISBI)Ni(alkyne)] **5** and transition state (**TS2**) describing the C–CN bond activation step.

To compare the theoretical pathway with experimental data, we computed the ^13^C KIEs using the Bigeleisen-Mayer equation with tunneling corrections (for more details see the Supporting Information). The proposed pathway shows a strong primary ^13^C KIE for both the α-carbon and the nitrile carbons, in line with the experimental values. In contrast, the KIE for the β-carbon is very close to unity, again in line with the experimental measurements. The calculated ^13^C KIEs are within the margin of error of the experimental values, thus not contradicting our proposed C(sp^3^)–CN bond oxidative addition pathway, which is facilitated by the coordination of the second nitrile moiety.

**Figure 7:**
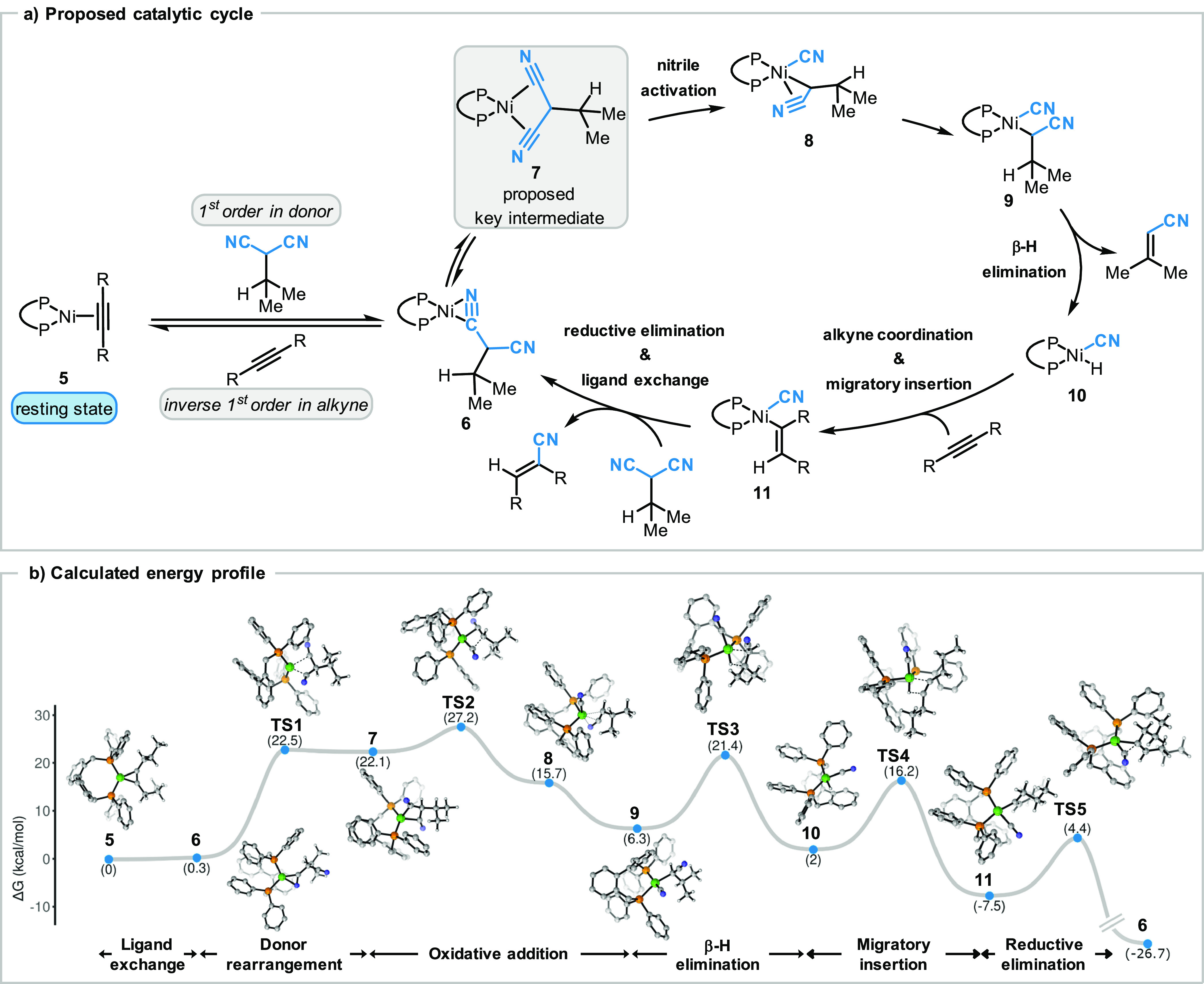
(a) Proposed catalytic cycle of the transfer hydrocyanation of alkynes. (b) Geometry optimization and frequency calculations were run with PBE0-D3BJ def2-TZVP(Ni)/def2-SVP (other atoms), solvation effects were included using cpcm (toluene), and the frequency temperature was set to 373.15 K. Single point energies were run with PBE0-D3BJ def2-QZVP(Ni)/def2-TZVP (other atoms), and solvation effects were included using cpcm (toluene) (for more details see Supporting Information). The plot was generated with EveRplot.^48^

Finally, we examined the remaining, kinetically invisible elementary steps of the transfer hydrocyanation of alkynes by DFT analysis (for more details see Supporting Information). The C–CN bond activation is followed by a lower activation barrier for the β-H elimination, further supporting the experimental observation that the oxidative addition is the rate-limiting step and not the β-H elimination (Figure 7). The rest of the computed pathway suggests an increasingly exothermic trajectory, resulting in an overall Δ*G* = −26.7 kcal/mol in favor of the hydrocyanation product and the corresponding vinyl nitrile byproduct.

### Proposed Catalytic Cycle

Based on both experimental and theoretical data, we propose the following catalytic cycle for the transfer hydrocyanation of alkynes (Figure 7a). The catalytic resting state is the [(BIBSI)Ni(alkyne)] species **5**, while the coexistence of the malononitrile donor coordination complex **6** could be identified as a minor species at room temperature. After rate-determining C–CN bond activation by the nickel catalyst, which we verified by the experimentally derived large ^13^C KIE for both the α-carbon and the nitriles and additional DFT calculations, a proposed [(BISBI)Ni(alkyl)(CN)] intermediate **9** is formed. After β-H elimination, the active hydrocyanation catalyst, [(BISBI)Ni(H)(CN)] **10**, and the byproduct **4a** are generated.

Based on previous mechanistic studies on the nickel-catalyzed hydrocyanation reaction,^47^ a migratory insertion of an alkyne is proposed to afford an alkenyl-nickel-nitrile intermediate **11**. Finally, reductive elimination releases the hydrocyanation product, and ligand exchange enables the regeneration of the Ni(0) catalyst, with an overall strong exothermic driving force.

## Conclusions

In conclusion, a transfer hydrocyanation of alkynes was developed to investigate the activation of the malononitrile-based HCN donor reagent by the nickel catalyst in the absence of preactivating Lewis acids. Alkenyl nitriles could be selectively accessed from the corresponding alkynes in good yields. Kinetic analysis of the transfer hydrocyanation reaction and deuterium labelling experiments of the donor reagents revealed that the [(BIBSI)Ni(alkyne)] species is likely the resting state and the C(sp^3^)–CN bond activation is the rate-determining step of the overall transformation. These results were further confirmed by experimentally determining the ^13^C KIE of the donor reagent at natural abundance using quantitative ^13^C NMR techniques. The mode of activation of the aliphatic C–CN bond by the nickel catalyst in the absence of preactivating Lewis acids was further examined by comparing experimentally and computationally derived values. Overall, an oxidative addition into the C(sp^3^)–CN bond is proposed, facilitated by the side-on coordination of the second nitrile group in the malononitrile-derived HCN donor reagent to the nickel catalyst. This directing effect of a nitrile moiety to facilitate the activation of strong carbon–carbon bonds can potentially be exploited in other settings and should thus inspire the design of new reagents for organic synthesis.

## Supporting Information Available

Experimental procedures, spectral, crystallographic data, and computational details (PDF)
Cartesian coordinates (TXT)
