# Supplementary material for: Mechanistic Investigation of the Nickel-Catalyzed Transfer Hydrocyanation of Alkynes
Source: ACS Catal. 2023 Aug 16;13(17):11548–55. doi: 10.1021/acscatal.3c02977 (PMC10476158; doi:10.1021/acscatal.3c02977)
Supplement: Supplementary file 1 — cs3c02977_si_001.pdf [file cs3c02977_si_001.pdf]

Mechanistic Investigation of the Nickel-Catalyzed Transfer Hydrocyanation of Alkynes

Julia C. Reisenbauer, Patrick Finkelstein, Marc-Olivier Ebert, and Bill Morandi*

ETH Zürich, Vladimir-Prelog-Weg 3, HCI, 8093 Zürich, Switzerland
Corresponding author: bill.morandi@org.chem.ethz.ch

Supporting Information

1. General information 2
2. Reagent synthesis 4
3. Optimization of the transfer hydrocyanation reaction of alkynes 7
4. Substrate scope of the transfer hydrocyanation of alkynes 8
5. Large-scale reaction 11
6. Unsuccessful substrates 11
7. Initial rate kinetics 12
   a) Kinetic profile of the transfer hydrocyanation reaction of alkynes: 12
   b) Order in reagents 15
8. Kinetic isotope effects 26
   a) Differentiation between α-H and β-H: Evaluation of reaction products 26
   b) H/D kinetic isotope effects – parallel reactions (β-H transfer) 27
   c) H/D kinetic isotope effects – parallel reactions (α-H transfer) 30
   d) ^{13}C kinetic isotope effect 31
9. Theoretical derivation of the rate law for the transfer hydrocyanation of alkynes 38
10. Synthesis of nickel complexes 39
11. NMR studies 40
   a) Formation of coordination complexes at room temperature 40
   b) VT-NMR analysis of standard reaction conditions 41
   c) Attempts to identify oxidative addition complexes 43
   d) Probing SET pathways 45
12. Computational details 48
   a) General information 48
   b) Activation of 3-methylbutanenitrile donor 48
   c) Kinetic isotope effect 49
13. NMR data 51
14. X-ray 91
   a) Crystal data of [(BISBI)Ni(cod)] complex 91
   b) Crystal data of [(BISBI)Ni(4-octyne)] complex 93
   c) Crystal data of hexameric Ni-complex after reaction with 2-isopropylmalononitrile 95
   d) Crystal data of hexameric Ni-complex after reaction with 2-isocyano-2-(*p*-tolyl)acetonitrile 96
15. References 98

1. General information

Unless otherwise stated, reagents were used as supplied from commercial sources without any further purification. Bis(1,5-cyclooctadiene)nickel(0), Ni(cod)$_2$, was purchased from Strem and 2,2'-bis(diphenylphosphinomethyl)-1,1'-biphenyl (BISBI) from abcr GmbH. Both reagents were used as received and stored in a glovebox at –36 °C under an argon atmosphere. Solvents were dried using an LC Technology Solutions solvent purification system under an atmosphere of N_2 (H_2O content < 10 ppm, as determined by Karl-Fischer titration) and stored over molecular sieves.
All glassware was dried for at least one hour in an oven set at 100 °C prior to use. All reactions using Ni(cod)$_2$ were carried out in 4 mL screw-cap vials and were set up under an argon atmosphere glovebox (LABmaster Pro SP, MBraun).

NMR: ^{1}H, ^{13}C, ^{19}F, and ^{31}P NMR spectra were recorded on a Bruker AVIII 400 MHz, a Bruker Neo 400 MHz or a Bruker Neo 500 MHz spectrometer and are reported in parts per million (ppm). ^{1}H NMR spectra are calibrated with respect to the corresponding residual solvent peak ($CHCl_3$: 7.26 ppm, toluene: 2.08 ppm, THF: 1.72 ppm). ^{13}C NMR spectra were recorded with broadband ^{1}H decoupling and are calibrated with respect to the corresponding residual solvent peak ($^{13}CDCl_3$: 77.16 ppm, d_8-toluene: 20.43 ppm, d_8-THF: 67.21 ppm). Multiplet signals are reported as follows: s = singlet, d = doublet, t = triplet, q = quartet, quint = quintet, sept = septet, m = multiplet, br = broad, or combinations thereof. ^{13}C and ^{19}F signals are singlets unless otherwise stated. ^{2}H NMR spectra were recorded on a Bruker AVIII HD 500 MHz spectrometer and are reported in parts per million (ppm).

^{13}C Kinetic Isotope Effects at Natural Abundance: Quantitative ^{13}C spectra and inversion experiments were recorded on a Bruker AVIII 600 MHz spectrometer equipped with a DCH cryoprobe optimized for ^{13}C detection. The interscan delay (d1 + acquisition time) was set to 90 s. For each spectrum, 67 072 complex points were acquired. The data points were extended by zero-filling to give a spectral size of 262 144 points. The spectral width was 248.5 ppm and the transmitter position was set at 65.4 ppm (o1p). Exponential apodization was applied with a line broadening of 2 Hz for inversion experiments and 0.5 Hz for quantitative ^{13}C experiments.
In each reaction, the recovered crude reaction mixture was compared with the starting material of the same lot. The NMR samples of the starting and recovered material were prepared identically in d_8-toluene. All spectra were manually integrated using Mestrenova. Each individual peak was independently integrated three times with slightly different phasing and integration regions to account for processing and integration errors.

Gas chromatography (GC-FID) was recorded on a Shimadzu GC-2025 (capillary column: Macherey-Nagel OPTIMA 5, 30.0 m × 0.25 × 0.25 µm; carrier gas: H_2). To determine GC yields, calibration curves using dodecane or tetradecane as an internal standard were generated.

Analytical thin layer chromatography was performed using silica gel 60 F254 coated aluminium sheets (Merck). Visualization was achieved by ultraviolet fluorescence (λ = 254 nm) and/or staining with potassium permanganate ($KMnO_4$).

Flash column chromatography and automated flash column chromatography (Biotage: Isolera One) were performed using silica gel 60 (pore size = 60 Å, mesh: 40-63 µm from Sigma-Aldrich or SiliCycle).

High-resolution mass spectrometry (HRMS) data were obtained by the mass spectrometry service in the Laboratorium für Organische Chemie at ETH Zürich on VG-TRIBRIB for electron impact ionization (EI), a Varian IonSpec Spectrometer for electrospray ionization (ESI) or an IonSpec Ultima Fourier Transform Mass Spectrometer for matrix-assisted laser desorption/ionization (MALDI) and are reported as (m/z).

X-Ray analysis: Single crystalline samples were measured on a Riguka Oxford Diffraction XtaLAB Synergy-S Dualflex kappa diffractometer equipped with a Dectris Pilatus 300 HPAD detector and using microfocus sealed tube Cu-Kα radiation with mirror optics (λ = 1.54178 Å). All measurements were carried out at 100 K (unless otherwise noted) using an Oxford Cryosystems Cryostream 800 sample cryostat. Data collected on the Rigaku instrument were integrated using CrysAlisPro and corrected for absorption effects using a combination of empirical (ABSPACK) and numerical corrections.[1] The structures were solved using SHELXT[2] or SHELXS[3] and refined by full-matrix least-squares analysis (SHELXL),[4] using the program package OLEX2.[5] Unless otherwise indicated below, all non-hydrogen atoms were refined anisotropically and hydrogen atoms were constrained to ideal geometries and refined with fixed isotropic displacement parameters (in terms of a riding model).

Fourier Transformed Infrared spectroscopy (FT-IR) measurements were carried out using a Bruker INVENIO-R FT-IR Spectrometer equipped with a diamond ATR. Selected bands are reported. Bands arising from atmospheric CO_2 are sometimes visible around 2361 cm^{-1} and 2335 cm^{-1} due to incomplete background subtraction.

2. Reagent synthesis

2-Isopropylmalononitrile (**2a**)

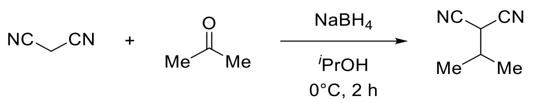

A solution of malononitrile (3.30 g, 50.0 mmol) and acetone (7.30 mL, 100 mmol, 2.0 equiv) in ^{i}PrOH (100 mL) was cooled to 0 °C and $NaBH_4$ (1.89 g, 50.0 mmol, 1.0 equiv) was added portionwise. The reaction mixture was stirred at 0 °C for 2 hours and was then acidified by addition of 1 M HCl solution. The aqueous phase was extracted with CH_2Cl_2 and the combined organic extracts were washed with brine, then dried over Na_2SO_4. After filtration, the solvents were removed under reduced pressure, and the residue was purified by flash column chromatography (SiO_2, 0 – 15% Et_2O in pentanes) to afford the product **2a** as a colorless oil (yield = 2.54 g, 47%).

Note: Prior to the use of the donor molecule in the catalytic transformations, the pure donor product was filtered over a plug of activated neutral alumina in the glovebox.

^{1}H NMR (400 MHz, $CDCl_3$) δ 3.59 (d, J = 5.4 Hz, 1H), 2.36 (m, 1H), 1.24 (d, J = 6.8 Hz, 6H).
^{13}C NMR (101 MHz, $CDCl_3$) δ 112.1, 31.3, 30.4, 19.6.
HRMS: m/z for $C_6H_9N_2$ $[M+H]^+$ calc. 109.0760; found 109.0761.
The spectral data are consistent with those reported in the literature.[6]

2-(Propan-2-yl-1,1,1,3,3,3-d$_6$)malononitrile (***d*-2a**)

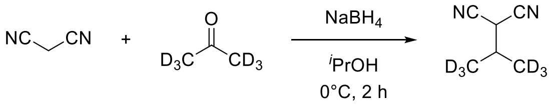

A solution of malononitrile (3.30 g, 50.0 mmol) and d_6-acetone (7.30 mL, 100 mmol, 2.0 equiv) in ^{i}PrOH (100 mL) was cooled to 0 °C and $NaBH_4$ (3.80 g, 100 mmol, 2.0 equiv) was added portionwise. The reaction mixture was stirred at 0 °C for 2 hours and was then acidified by addition of 1 M HCl solution. The aqueous phase was extracted with CH_2Cl_2 and the combined organic extracts were washed with brine, then dried over Na_2SO_4. After filtration, the solvents were removed under reduced pressure, and the residue was purified by flash column chromatography (SiO_2, 0 – 6% Et_2O in pentanes) to afford the product ***d*-2a** as a colorless oil (yield = 3.15 g, 55% with > 95% D incorporation).

^{1}H NMR (400 MHz, $CDCl_3$) δ 3.58 (d, J = 5.5 Hz, 1H), 2.33 (d, J = 5.5 Hz, 1H).
^{2}H NMR (77 MHz, $CDCl_3$) δ 1.22 (d, J = 1.0 Hz).
^{13}C NMR (101 MHz, $CDCl_3$) δ 112.1, 30.9, 30.4, 18.7 (dt, J = 39.0, 19.4 Hz).
HRMS: m/z for $C_6H_3D_6N_2$ $[M+H]^+$ calc. 115.1137; found 115.1137.
IR (FT-IR, 64 scans, cm^{-1}): ν = 2916, 2258, 2224, 2073, 1338, 1219, 1140, 1047, 877, 721, 490.

2-Cyclopentylmalononitrile (**2b**)

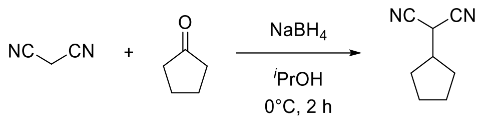

A solution of malononitrile (6.61 g, 100 mmol) and cyclopentanone (17.7 mL, 200 mmol, 2.0 equiv) in ^{i}PrOH (200 mL) was cooled to 0 °C and $NaBH_4$ (7.57 g, 200 mmol, 2.0 equiv) was added portionwise. The reaction mixture was stirred at 0 °C for 2 hours and was then acidified by addition of 1 M HCl solution. The aqueous phase was extracted with CH_2Cl_2 and the combined organic extracts were washed with brine, then dried over Na_2SO_4. After filtration, the solvents were removed under reduced pressure, and the residue was purified by flash column chromatography (SiO_2, 0 – 5% EtOAc in hexanes) to afford the product **2b** as a colorless oil (yield = 3.80 g, 28%).

^{1}H NMR (400 MHz, $CDCl_3$) δ 3.68 (d, *J* = 6.6 Hz, 1H), 2.57 – 2.46 (m, 1H), 2.13 – 1.92 (m, 2H), 1.85 – 1.75 (m, 2H), 1.75 – 1.64 (m, 2H), 1.59 – 1.46 (m, 2H).
^{13}C NMR (101 MHz, $CDCl_3$) δ 112.6, 41.0, 30.5, 27.6, 25.3.
HRMS: m/z for $C_8H_{10}N_2$ $[M-H]^+$ calc. 133.0760; found 133.0762.
IR (FT-IR, 64 scans, cm^{-1}): ν = 2960, 2947, 2877, 2256, 1452, 1317, 1003, 883, 577, 451.

2-(Cyclopentyl-1-*d*)malononitrile (***d***$_β$**-2b**)

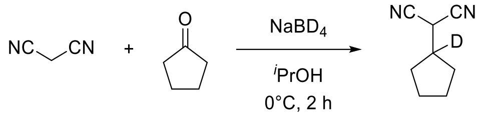

A solution of malononitrile (0.66 g, 10 mmol) and cyclopentanone (1.8 mL, 20 mmol, 2.0 equiv) in ^{i}PrOH (20 mL) was cooled to 0 °C and $NaBD_4$ (0.84 g, 20 mmol, 2.0 equiv) was added portionwise. The reaction mixture was stirred at 0 °C for 2 hours and was then acidified by addition of 1 M HCl solution. The aqueous phase was extracted with CH_2Cl_2 and the combined organic extracts were washed with brine, then dried over Na_2SO_4. After filtration, the solvents were removed under reduced pressure, and the residue was purified by flash column chromatography (SiO_2, 0 – 5% EtOAc in hexanes) to afford the product ***d***$_β$**-2b** as a colorless oil (yield = 0.67 g, 49% with 98% D incorporation).

^{1}H NMR (400 MHz, $CDCl_3$) δ 3.67 (s, 1H), 2.10 – 1.98 (m, 2H), 1.87 – 1.74 (m, 2H), 1.74 – 1.62 (m, 2H), 1.56 – 1.47 (m, 2H).
^{2}H NMR (92 MHz, $CDCl_3$) δ 2.50 (s).
^{13}C NMR (101 MHz, $CDCl_3$) δ 112.6, 40.6 (m), 30.4, 27.6, 25.3.
HRMS: m/z for $C_8H_9DN_2$ $[M-H]^+$ calc. 134.0823; found 134.0825.
IR (FT-IR, 64 scans, cm^{-1}): ν = 2960, 2947, 2868, 2256, 1452, 1444, 1196, 930, 577.

2-Cyclopentylmalononitrile-*d* (***d***$_α$**-2b**)

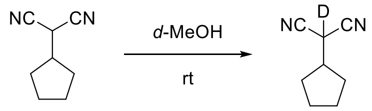

2-Cyclopentylmalononitrile **2b** (100 mg, 0.750 mmol) was dissolved in CD_3OD (1 mL) and stirred for 2 minutes at room temperature. Then the mixture was concentrated under reduced pressure. This process was repeated 5 times. The product was obtained as a colorless oil (100 mg, >99% with 65 % D content).

^{1}H NMR (600 MHz, toluene-d_8) δ 1.45 – 1.38 (m, 1H), 1.37 – 1.30 (m, 2H), 1.30 – 1.22 (m, 2H), 1.16 – 1.07 (m, 2H), 0.93 – 0.85 (m, 2H).
^{2}H NMR (92 MHz, toluene-d_8) δ 2.06 (d, *J* = 6.9 Hz).
^{13}C NMR (100 MHz, toluene-d_8) δ 113.2, 40.9, 30.5, 26.8, 25.5.
HRMS: m/z for $C_8H_9N_2$ $[M-D]^+$ calc. 133.0760, found 133.0760.
IR (FT-IR, 64 scans, cm^{-1}): ν = 2960, 2874, 2256, 1454, 1315, 864, 841, 577.

2-(*p*-Tolyl)malononitrile **2c**

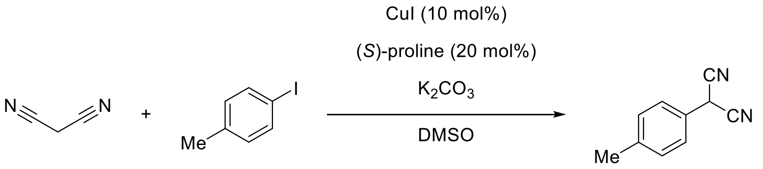

2-(*p*-Tolyl)malononitrile **2c** was synthesized following a modified procedure.[7] In a flame-dried and nitrogen filled 100 mL Schlenk flask, K_2CO_3 (5.53 g, 40.0 mmol, 4.0 equiv), CuI (190 mg, 1.00 mmol, 10 mol%), (S)-proline (0.23 g, 2.0 mmol, 20 mol%) and malononitrile (1.98 g, 30.0 mmol, 3.0 equiv) were suspended in dry DMSO (20 mL). After the addition of 4-methyliodobenzene

(1.12 mL, 10.0 mmol), the flask was sealed and the reaction mixture was stirred for 18 h at 90 °C. After cooling to room temperature, the suspension was poured into aq. HCl (2 M, 100 mL). The aqueous layer was extracted with EtOAc three times. The combined organic layers were washed with water and brine, dried over Na_2SO_4, filtered, and the solvent was removed under reduced pressure. The product was purified by column chromatography (SiO_2, 0 – 50% CH_2Cl_2 in hexanes) and obtained as a pinkish solid (1.04 g, 67%).

^{1}H NMR (400 MHz, toluene-d_8) δ 6.82 – 6.72 (m, 2H), 6.70 – 6.61 (m, 2H), 3.38 (s, 1H), 1.91 (s, 3H).
^{13}C NMR (101 MHz, toluene-d_8) δ 140.3, 130.8, 127.3, 124.7, 112.8, 27.1, 21.1.
The spectral data are consistent with those reported in the literature.[8]

3. Optimization of the transfer hydrocyanation reaction of alkynes

To an oven-dried 4 mL screw-cap vial, 2-isopropylmalononitrile **2a** (27 mg, 0.25 mmol, 1.0 equiv), anhydrous toluene (0.05 mL), and 4-octyne **1a** (37 µL, 0.25 mmol) were added under an argon atmosphere in a glovebox. In another oven-dried vial, BISBI (14 mg, 0.025 mmol, 10 mol%) and $Ni(cod)_2$ (6.9 mg, 0.025 mmol, 10 mol%) were dissolved in anhydrous toluene (0.45 mL) and the mixture was stirred until complete dissolution (solution turned dark red). The precatalyst was then added to the starting material in one portion. The vial was sealed and removed from the glovebox then heated at 100 °C for 18 hours. After cooling to room temperature, *n*-dodecane (20 µL) was added as an internal standard, the crude mixture was diluted with EtOAc, an aliquot (0.2 mL) was filtered through a plug of cellulose and then subjected to GC-FID analysis.

Table S1 Optimization of transfer hydrocyanation of alkynes.

Entry	Deviation from initial conditions	GC Yield of 3a [%]
1	none	91
2	2-isopropylmalononitrile (1.2 equiv instead of 1.0 equiv)	97
3	2-isopropylmalononitrile (1.5 equiv instead of 1.0 equiv)	96
4	2-isopropylmalononitrile (1.7 equiv instead of 1.0 equiv)	95
5	2-isopropylmalononitrile (2.0 equiv instead of 1.0 equiv)	96
6	0.4 M instead of 0.5 M	88
7	0.3 M instead of 0.5 M	91
8	0.25 M instead of 0.5 M	79
9	80 °C instead of 100 °C	91
10	90 °C instead of 100 °C	95
11	110 °C instead of 100 °C	91
12	120 °C instead of 100 °C	94
13	7.5 mol% $Ni(cod)_2$/BISBI instead of 10 mol% $Ni(cod)_2$/BISBI	93
14	5.0 mol% $Ni(cod)_2$/BISBI instead of 10 mol% $Ni(cod)_2$/BISBI	71
15	2.5 mol% $Ni(cod)_2$/BISBI instead of 10 mol% $Ni(cod)_2$/BISBI	35
16	1.0 mol% $Ni(cod)_2$/BISBI instead of 10 mol% $Ni(cod)_2$/BISBI	12

4. Substrate scope of the transfer hydrocyanation of alkynes

General procedure for the hydrocyanation of alkynes:

To an oven-dried 4 mL screw-cap vial, 2-isopropylmalononitrile **2a** (65 mg, 0.60 mmol, 1.2 equiv), anhydrous toluene (0.15 mL), and alkyne (0.50 mmol) were added under an argon atmosphere in a glovebox. In another oven-dried vial, BISBI (28 mg, 0.050 mmol, 10 mol%) and $Ni(cod)_2$ (14 mg, 0.050 mmol, 10 mol%) were dissolved in anhydrous toluene (0.6 mL) and the mixture was stirred until complete dissolution (solution turned dark red). The precatalyst was then added to the starting materials in one portion. The vial was sealed and removed from the glovebox then heated at 100 °C for 18 hours. After cooling to room temperature, the crude reaction mixture was concentrated under reduced pressure and purified by flash column chromatography.

(*E*)-2-Butylhept-2-enenitrile (**3b**)

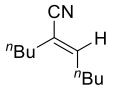

3b

Prepared according to the general procedure using dec-5-yne (90 µL, 0.50 mmol). Purified by flash column chromatography (SiO_2, 0–5% EtOAc in hexanes) to give the product **3b** as a yellowish oil (yield = 65.4 mg, 79%).

^{1}H NMR (400 MHz, $CDCl_3$) δ 6.33 (tt, J = 7.5, 1.2 Hz, 1H), 2.23 – 2.13 (m, 4H), 1.57 – 1.47 (m, 2H), 1.45 – 1.27 (m, 6H), 0.92 (q, J = 7.2 Hz, 6H).

^{13}C NMR (101 MHz, $CDCl_3$) δ 148.2, 120.4, 115.1, 30.7, 30.3, 28.3, 28.3, 22.4, 22.2, 13.9, 13.9.

HRMS: m/z for $C_{11}H_{18}N$ $[M-H]^+$ calc. 164.1434, found 164.1435.

The spectral data are consistent with those reported in the literature.[9]

(*E*)-2-Benzylidenepentanenitrile (**3c**)

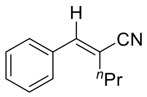

3c

Prepared according to the general procedure using 1-phenyl-1-pentyne (62 µL, 0.50 mmol). Purified by flash column chromatography (SiO_2, hexanes) to give the product **3c** as a yellowish oil (yield = 39.7 mg, 45%). Based on NOESY NMR data, a coupling between the vinylic H and the protons of the phenyl ring as well as a coupling between the alkyl chain and the protons of the phenyl group are observed, suggesting the formation of product **3c** as the only isomer.

^{1}H NMR (400 MHz, $CDCl_3$) δ 7.42 – 7.36 (m, 3H), 7.32 – 7.28 (m, 2H), 7.23 (s, 1H), 2.47 – 2.42 (m, 2H), 1.75 – 1.65 (m, 2H), 0.98 (t, J = 7.4 Hz, 3H).

^{13}C NMR (101 MHz, $CDCl_3$) δ 144.4, 134.3, 129.3, 129.2, 128.8, 120.5, 116.1, 31.4, 21.7, 13.7.

HRMS: m/z for $C_{12}H_{14}N$ $[M-H]^+$ calc. 172.1121, found 172.1122.

IR (FT-IR, 64 scans, cm^{-1}): ν = 3028, 2962, 2874, 2212, 1493, 1446, 1076, 928, 748, 696.

(*E*)-2-Methyl-3-(triisopropylsilyl)acrylonitrile (**3d**)

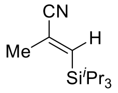

3d

Prepared according to the general procedure using triisopropyl(porp-1-yn-1-yl)silane (0.12 mL, 0.50 mmol). Purified by flash column chromatography (SiO_2, hexanes) to give the product **3d** as a colorless oil (yield = 48.3 mg, 43%).

^{1}H NMR (400 MHz, $CDCl_3$) δ 6.52 (q, J = 1.3 Hz, 1H), 2.04 (d, J = 1.3 Hz, 3H), 1.24 – 1.14 (m, 3H), 1.06 (d, J = 7.1 Hz, 18H).

^{13}C NMR (101 MHz, $CDCl_3$) δ 146.9, 125.3, 120.7, 21.6, 18.8, 12.0.

HRMS: m/z for $C_{13}H_{25}NSi$ $[M]^+$ calc. 223.1751, found 223.1751.

The spectral data are consistent with those reported in the literature.[10]

(*Z*)-2-Phenyl-3-(trimethylsilyl)acrylonitrile (**3e**), (*E*)-2-phenyl-3-(trimethylsilyl)acrylonitrile (**3e'**)

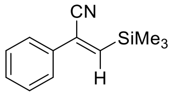

3e

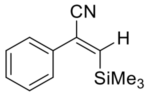

3e'

Prepared according to the general procedure using 1-phenyl-2-(trimethylsilyl)-acetylene (98 µL, 0.50 mmol). Purified by flash column chromatography (SiO_2, hexanes) to give the products **3e** and **3e'** as colorless oils (major isomer = 46.3 mg, 46%; minor isomer = 11.1 mg, 11%).

Major isomer 3e:

^{1}H NMR (400 MHz, $CDCl_3$) δ 7.41 – 7.34 (m, 5H), 6.88 (s, 1H), 0.02 (s, 9H).

^{13}C NMR (101 MHz, $CDCl_3$) δ 152.4, 135.5, 129.6, 129.2, 128.7, 128.4, 119.5, -0.4.

HRMS: m/z for $C_{12}H_{16}NSi$ $[M\text{-}H]^+$ calc. 202.1047, found 202.1044.

IR (FT-IR, 64 scans, cm^{-1}): ν = 2956, 1562, 1493, 1444, 1250, 1005, 862, 833, 750, 696, 625.

The spectral data are consistent with those reported in the literature.[11]

Minor isomer 3e':

^{1}H NMR (400 MHz, $CDCl_3$) δ 7.64 – 7.60 (m, 2H), 7.44 – 7.36 (m, 3H), 7.10 (s, 1H), 0.34 (s, 9H).

The spectral data are consistent with those reported in the literature.[11]

(*Z*)-2-(4-(Trifluoromethyl)phenyl)-3-(trimethylsilyl)acrylonitrile (**3f**), (*E*)-2-(4-(trifluoromethyl)phenyl)-3-(trimethylsilyl)acrylonitrile (**3f'**)

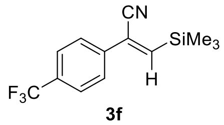

3f

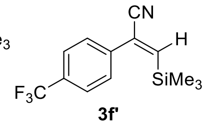

3f'

Prepared according to the general procedure using 1-[(trimethylsilyl)ethynyl]-4-(trifluoromethyl)benzene (0.12 mL, 0.50 mmol). Purified by flash column chromatography (SiO_2, hexanes) to give the products **3f** and **3f'** as pale-yellow oils (major isomer = 77.6 mg, 58%; minor isomer = 44.2 mg, 33%).

Major isomer 3f:

^{1}H NMR (500 MHz, $CDCl_3$) δ 7.75 – 7.71 (m, 2H), 7.69 – 7.64 (m, 2H), 7.21 (s, 1H), 0.36 (s, 9H). The *anti*-addition isomer was confirmed by NOESY NMR analysis, as a coupling between the vinylic proton and the protons of the phenyl ring was observed.

^{13}C NMR (126 MHz, $CDCl_3$) δ 151.0, 138.3, 131.6 (q, J = 32.9 Hz), 126.7, 126.2, 126.1 (q, J = 3.8 Hz), 123.9 (q, J = 272.3 Hz), 117.5, -1.4.

^{19}F NMR (376 MHz, $CDCl_3$) δ -62.77.

HRMS: m/z for $C_{13}H_{15}F_3NSi$ $[M\text{-}H]^+$ calc. 270.0920, found 270.0917.

IR (FT-IR, 64 scans, cm^{-1}): ν = 2960, 1616, 1412, 1325, 1126, 1070, 837, 679.

Minor isomer 3f':

^{1}H NMR (400 MHz, $CDCl_3$) δ 7.97 – 7.91 (m, 2H), 7.55 – 7.47 (m, 2H), 7.01 (s, 1H), 0.03 (s, 6H).

(*E*)-2-(4-Methoxyphenyl)-3-(trimethylsilyl)acrylonitrile (**3g**) and (*Z*)-2-(4-methoxyphenyl)-3-(trimethylsilyl)acrylonitrile (**3g'**)

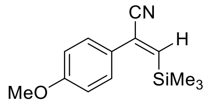

3g

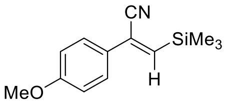

3g'

Prepared according to the general procedure using (4-methoxyphenyl-ethynyl)trimethylsilane (0.11 mL, 0.50 mmol). Purified by flash column chromatography (SiO_2, 0–10% EtOAc in hexanes) to give the products **3g** and **3g'** as pale-yellow oils (yield = 98.3 mg, 85%, including 10% of minor isomer).

Major isomer 3g:

^{1}H NMR (500 MHz, $CDCl_3$) δ 7.33 – 7.28 (m, 2H), 6.92 – 6.88 (m, 2H), 6.77 (s, 1H), 3.84 (s, 3H), 0.04 (s, 9H).

^{13}C NMR (126 MHz, $CDCl_3$) δ 160.5, 150.5, 129.6, 128.7, 127.9, 119.6, 113.8, 55.4, -0.5.

HRMS: m/z for $C_{13}H_{18}NOSi$ $[M\text{-}H]^+$ calc. 232.1152, found 232.1150.

IR (FT-IR, 64 scans, cm^{-1}): ν = 2956, 1606, 1508, 1294, 1248, 1173, 1034, 831, 750.

Minor isomer 3g' (tentatively assigned)

^{1}H NMR (500 MHz, $CDCl_3$) δ 7.88 – 7.82 (m, 2H), 7.09 (s, 1H), 6.95 – 6.92 (m, 2H), 3.85 (s, 3H), 0.31 (s, 9H).

^{13}C NMR (126 MHz, $CDCl_3$) δ 161.5, 153.6, 131.0, 128.5, 127.0, 120.0, 114.2, 55.4, -1.9.

(*E*)-2-((Triethylsilyl)methylene)-4-((triethylsilyl)oxy)butanenitrile (**3h**)

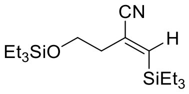

3h

Prepared according to the general procedure using 1-triethylsilyl-4-triethylsilyloxy-1-butyne (0.18 mL, 0.50 mmol). Purified by flash column chromatography (SiO_2, 0–10% EtOAc in hexanes) to give the product **3h** as a colorless oil (yield = 121.4 mg, 75%).

^{1}H NMR (500 MHz, $CDCl_3$) δ 6.59 (t, J = 0.9 Hz, 1H), 3.81 (t, J = 6.6 Hz, 2H), 2.50 (td, J = 6.6, 0.9 Hz, 2H), 0.96 (t, J = 7.9 Hz, 18H), 0.73 – 0.66 (m, 6H), 0.65 – 0.56 (m, 6H).

^{13}C NMR (126 MHz, $CDCl_3$) δ 149.4, 127.6, 119.6, 60.7, 37.9, 7.4, 6.9, 4.4, 4.2.

HRMS: m/z for $C_{17}H_{36}NOSi_2$ $[M-H]^+$ calc. 326.2330, found 326.2325.

IR (FT-IR, 64 scans, cm^{-1}): ν = 2955, 2875, 1458, 1238, 1099, 1003, 719.

(*E*)-5-Hydroxy-2-((triethylsilyl)methylene)pentanenitrile (**3i**) (*Z*)-5-hydroxy-2-((triethylsilyl)methylene)pentanenitrile (**3i'**)

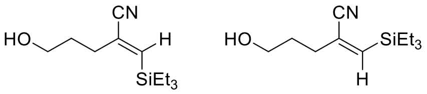

3i **3i'**

Prepared according to the general procedure using 5-(triethylsilyl)-4-pentyn-1-ol (0.12 mL, 0.50 mmol). Purified by flash column chromatography (SiO_2, 0–40% EtOAc in hexanes) to give the products 3**i** and 3**i'** as a yellowish oil (yield = 85.5 mg, 76%, including 10% of minor isomer).

Major isomer 3i

^{1}H NMR (500 MHz, $CDCl_3$) δ 6.53 (t, J = 1.0 Hz, 1H), 3.71 (t, J = 6.2 Hz, 2H), 2.43 – 2.36 (m, 2H), 1.89 – 1.79 (m, 2H), 1.33 (s, 1H), 0.96 (t, J = 7.9 Hz, 9H), 0.73 – 0.64 (m, 6H).

^{13}C NMR (126 MHz, $CDCl_3$) δ 147.8, 130.0, 119.7, 61.8, 31.6, 31.1, 7.5, 4.2.

HRMS: m/z for $C_{12}H_{24}NOSi$ $[M-H]^+$ calc. 226.1622, found 226.1620.

IR (FT-IR, 64 scans, cm^{-1}): ν = 3429, 2955, 2875, 1587, 1456, 1414, 1236, 1057, 1014, 858, 717.

Minor isomer 3i'

^{1}H NMR (500 MHz, $CDCl_3$) δ 6.38 (t, J = 1.4 Hz, 1H), 3.70 (s, 2H), 2.48 – 2.42 (m, 2H), 1.89 – 1.80 (m, 2H), 1.33 (s, 1H), 0.96 (t, J = 7.9 Hz, 9H), 0.79 – 0.73 (m, 6H).

^{13}C NMR (126 MHz, $CDCl_3$) δ 147.2, 129.2, 119.1, 61.5, 35.8, 30.9, 7.4, 3.4.

(*E*)-5-Chloro-2-((triethylsilyl)methylene)pentanenitrile (**3j**)

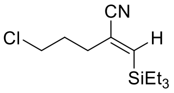

3j

Prepared according to the general procedure using 1-chloro-5-triethylsilyl-4-pentyne (0.11 g, 0.50 mmol). Purified by flash column chromatography (SiO_2, 0–10% EtOAc in hexanes) to give the product **3j** as a colorless oil (yield = 79.4 mg, 65%).

^{1}H NMR (400 MHz, $CDCl_3$) δ 6.55 (d, J = 1.0 Hz, 1H), 3.58 (t, J = 6.1 Hz, 2H), 2.44 (dd, J = 9.4, 6.3 Hz, 2H), 2.11 – 1.99 (m, 2H), 0.95 (t, J = 7.9 Hz, 9H), 0.69 (q, J = 7.8 Hz, 6H).

^{13}C NMR (101 MHz, $CDCl_3$) δ 148.7, 128.8, 119.4, 44.0, 31.9, 31.2, 7.4, 4.1.

HRMS: m/z for $C_{12}H_{21}ClNSi$ $[M-H]^+$ calc. 242.1126, found 242.1123.

IR (FT-IR, 64 scans, cm^{-1}): ν = 2945, 2868, 1464, 1385, 997, 883, 729, 663.

5. Large-scale reaction

Synthesis on a 3-mmol scale

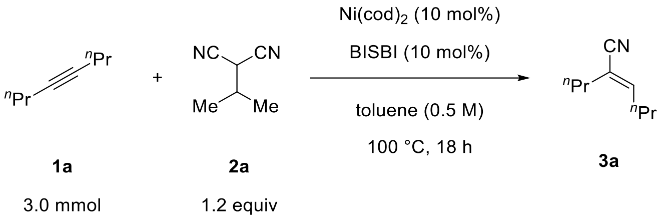

To an oven-dried 16 mL screw-cap vial, 2-isopropylmalononitrile **2a** (389 mg, 3.60 mmol, 1.2 equiv), anhydrous toluene (0.48 mL), and 4-octyne **1a** (0.44 mL, 3.0 mmol) were added under an argon atmosphere in a glovebox. In two separate oven-dried vials, BISBI (165 mg, 0.300 mmol, 10 mol%) was dissolved in anhydrous toluene (1.5 mL) and Ni(cod)$_2$ (82.5 mg, 0.300 mmol, 10 mol%) was dissolved in anhydrous toluene (2.5 mL), respectively. After stirring at room temperature until complete dissolution, the nickel precatalyst and the ligand solution were combined and then added to the starting material mixture in one portion. The vial was sealed and removed from the glovebox then heated at 100 °C for 18 hours. After cooling to room temperature, the crude reaction mixture was purified by flash column chromatography (SiO$_2$, 0–5% EtOAc in hexanes) to give the product **3a** as a colorless oil (yield = 350 mg, 85%).

6. Unsuccessful substrates

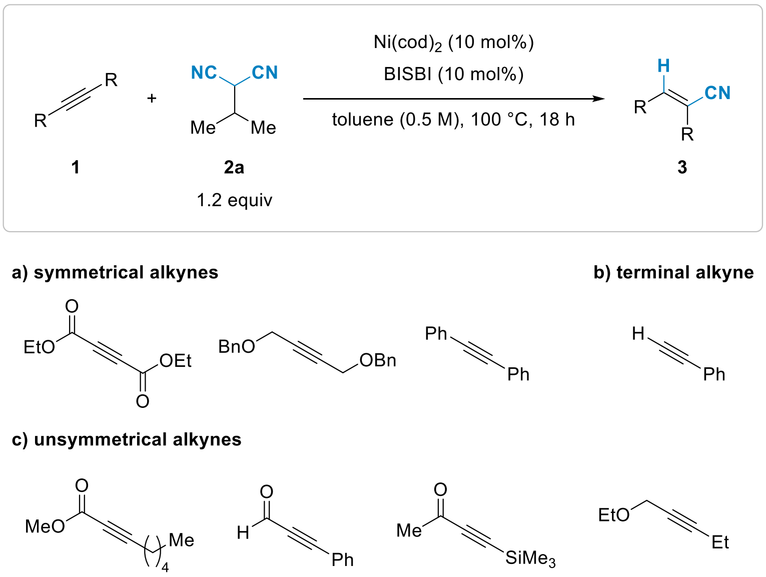

Figure S1 Unsuccessful substrates in the transfer hydrocyanation of alkynes.

7. Initial rate kinetics

a) Kinetic profile of the transfer hydrocyanation reaction of alkynes:

i. Standard reaction:

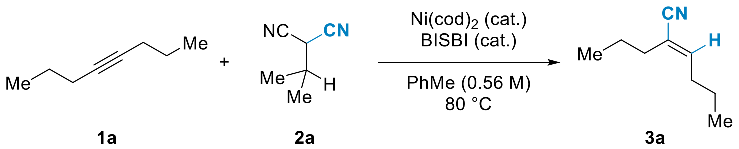

1a (0.23 mmol) **2a** (0.15 mmol) **3a**

General procedure:

Under an argon atmosphere in a glovebox in an oven-dried 4 mL screw-cap vial, a stock solution was prepared containing 2-isopropylmalononitrile **2a** (376 mg, 0.15 mmol per reaction, 1.0 equiv), anhydrous toluene (1.18 mL), and 4-octyne **1a** (0.78 mL, 0.23 mmol per reaction, 1.5 equiv). The mixture was then divided by taking each time 0.08 mL of the stock solution and adding it to oven-dried 4 mL screw-cap vials containing a magnetic stirring bar (set-up for 23 reactions). In two separate vials equipped with stirring bars, BISBI (153.6 mg, 0.015 mmol per reaction, 10 mol%), and Ni(cod)$_2$ (96.4 mg, 0.015 mmol per reaction, 10 mol%) were dissolved in anhydrous toluene (2.35 mL and 2.82 mL, respectively) and the mixtures were stirred until complete dissolution. Then the ligand and catalyst solution were combined and mixed, which led to a dark red precatalyst solution. Then 0.22 mL of the precatalyst solution was added to each of the 22 reaction vials. The vials were sealed and removed from the glovebox then heated at 100 °C for the indicated time. After cooling to room temperature, *n*-dodecane (20 μL) was added as an internal standard, the crude mixture was diluted with EtOAc, filtered through a plug of silica, and then subjected to GC-FID analysis to evaluate the reaction progress by determining the yield of the hydrocyanation product **3a**.

Table S2 Kinetic data of the hydrocyanation reaction under standard reaction conditions.

Entry	Reaction time	GC Yield of 3a [mmol]			
		Run 1	*Run 2*	*Run 3*	*Run 4*
1	10 min	0.003957	0.004041	0.007220	0.007171
2	20 min	0.010186	0.010232	0.014282	0.014020
3	30 min	0.016872	0.016746	0.020622	0.022020
4	40 min	0.022450	0.023298	0.031616	0.029496
5	50 min	0.031728	0.031492	0.037252	0.034205
6	60 min	0.038331	0.036745	0.044185	0.041847
7	75 min	0.046609	0.044864	0.050783	0.052850
8	90 min	0.052916	0.051191	-	0.060922
9	105 min	0.058598	0.055973	0.066056	0.069430
10	120 min	0.063760	0.063148	0.081555	0.078269
11	135 min	0.069083	0.067053	0.078177	0.080312
12	150 min	0.068891	0.068384	0.083535	0.084378
13	165 min	0.075895	0.074422	0.086895	0.085243
14	180 min	0.075076	0.068758	0.090256	0.094135
15	210 min	0.075946	0.070609	0.095116	0.099231
16	240 min	0.075040	0.074681	0.097384	0.101548
17	270 min	0.083019	0.081959	0.095565	0.101551
18	300 min	0.081974	0.081856	0.098455	0.099450
19	360 min	0.082503	0.083354	0.104876	0.107171
20	420 min	0.085015	0.091172	0.096647	0.110179
21	480 min	0.042003	0.093911	0.106526	0.107659
22	600 min	0.093084	0.092114	0.114021	0.105377

ii. Same excess experiment

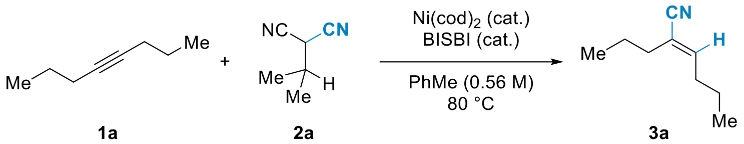

1a (0.18 mmol) **2a** (0.11 mmol) **3a**

General procedure:

Under an argon atmosphere in a glovebox in an oven-dried 4 mL screw-cap vial, a stock solution was prepared containing 2-isopropylmalononitrile **2a** (259 mg, 0.12 mmol per reaction, 1.0 equiv), anhydrous toluene (1.18 mL), and 4-octyne **1a** (0.61 mL, 0.18 mmol per reaction, 1.5 equiv). The mixture was then divided by taking each time 0.08 mL of the stock solution and adding it to oven-dried 4 mL screw-cap vials containing a magnetic stirring bar (set-up for 23 reactions). In two separate vials equipped with stirring bars, BISBI (195.1 mg, 0.015 mmol per reaction, 10 mol%), and Ni(cod)$_2$ (96.4 mg, 0.015 mmol per reaction, 10 mol%) were dissolved in anhydrous toluene (2.35 mL and 2.82 mL, respectively) and the mixtures were stirred until complete dissolution. Then the ligand and catalyst solution were combined and mixed, which led to a dark red precatalyst solution. Then 0.22 mL of the precatalyst solution was added to each of the 22 reaction vials. The vials were sealed and removed from the glovebox then heated at 100 °C for the indicated time. After cooling to room temperature, *n*-dodecane (20 µL) was added as an internal standard, the crude mixture was diluted with EtOAc, filtered through a plug of silica and then subjected to GC-FID analysis to evaluate the reaction progress by determining the yield of the hydrocyanation product **3a**.

Table S3 Kinetic data for the same excess experiment of the transfer hydrocyanation.

Entry	Reaction time	GC Yield of 3a [mmol]	
		Run 1	*Run 2*
1	10 min	0.004294	0.004490
2	20 min	0.012651	0.013303
3	30 min	0.021524	0.021652
4	40 min	0.028777	0.028178
5	50 min	0.036974	0.035331
6	60 min	0.040916	0.042317
7	75 min	0.051100	0.049821
8	90 min	0.054475	0.055140
9	105 min	0.063340	0.061051
10	120 min	0.063416	0.062536
11	135 min	0.067859	0.070308
12	150 min	0.070174	0.072087
13	165 min	0.075338	0.073521
14	180 min	0.074476	0.078658
15	210 min	0.083326	0.078959
16	240 min	0.081086	0.081924
17	270 min	0.084272	0.081248
18	300 min	0.085895	0.080199
19	360 min	0.082551	0.075539
20	420 min	0.084779	0.085165
21	480 min	0.075773	0.087355
22	600 min	0.082670	0.082758

iii. Product inhibition experiment

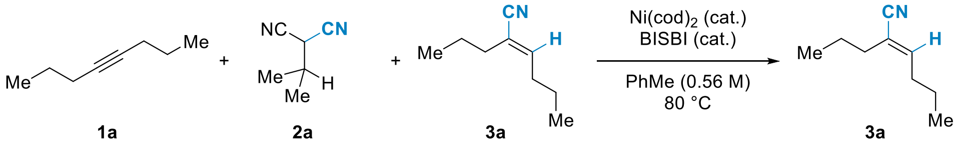

1a (0.18 mmol) **2a** (0.11 mmol) **3a** (0.045 mmol) **3a**

General procedure:

Under an argon atmosphere in a glovebox in an oven-dried 4 mL screw-cap vial, a stock solution was prepared containing 2-isopropylmalononitrile **2a** (268 mg, 0.11 mmol per reaction, 1.0 equiv), anhydrous toluene (1.18 mL), 4-octyne **1a** (0.62 mL, 0.18 mmol per reaction, 1.5 equiv), and (*E*)-2-propylhex-2-enenitrile **3a** (146 mg, 0.045 mmol per reaction, 0.30 equiv). The mixture was then divided by taking each time 0.08 mL of the stock solution and adding it to oven-dried 4 mL screw-cap vials containing a magnetic stirring bar (set-up for 23 reactions). In two separate vials equipped with stirring bars, BISBI (195.1 mg, 0.015 mmol per reaction, 10 mol%), and Ni(cod)$_2$ (96.4 mg, 0.015 mmol per reaction, 10 mol%) were dissolved in anhydrous toluene (2.35 mL and 2.82 mL, respectively) and the mixtures were stirred until complete dissolution. Then the ligand and catalyst solution were combined and mixed, which led to a dark red precatalyst solution. Then 0.22 mL of the precatalyst solution was added to each of the 22 reaction vials. The vials were sealed and removed from the glovebox then heated at 100 °C for the indicated time. After cooling to room temperature, *n*-dodecane (20 µL) was added as an internal standard, the crude mixture was diluted with EtOAc, filtered through a plug of silica and then subjected to GC-FID analysis to evaluate the reaction progress by determining the yield of the hydrocyanation product **3a**.

Table S4 Kinetic data for the product inhibition experiment of the transfer hydrocyanation.

Entry	Reaction time	GC Yield of 3a [mmol]			
		Run 1	*Run 2*	*Run 3*	*Run 4*
1	10 min	0.041795	0.041087	0.050221	0.048845
2	20 min	0.061426	0.066596	0.058285	0.059890
3	30 min	0.071865	0.070816	0.069379	0.065051
4	40 min	0.083273	0.082979	0.078418	0.076455
5	50 min	0.089188	0.088877	0.086923	0.078462
6	60 min	0.098647	0.110184	0.086269	0.092055
7	75 min	0.107821	0.106369	0.096682	0.094752
8	90 min	0.112453	0.119598	0.101396	0.103939
9	105 min	0.116544	0.124180	0.103812	0.110744
10	120 min	0.122167	0.122449	0.125766	0.118755
11	135 min	0.128909	0.130204	0.125940	0.128298
12	150 min	0.133369	0.143515	0.125566	0.135713
13	165 min	0.136367	0.139119	0.128236	0.135306
14	180 min	0.131592	0.135151	0.127079	0.132751
15	210 min	0.136034	0.133229	0.137104	0.138670
16	240 min	0.148000	0.131378	0.137956	0.139337
17	270 min	0.142787	0.121007	0.139966*	0.144295*
18	300 min	0.141477	0.153872	0.140028	0.150788
19	360 min	0.142667	0.144678	0.144069	0.137881
20	420 min	0.128764	0.142976	0.130984	0.143456
21	480 min	0.133277	0.141311	0.137698	0.138102
22	600 min	0.144888	0.132502	0.143671	0.134032

*Reactions were stopped after 285 min.

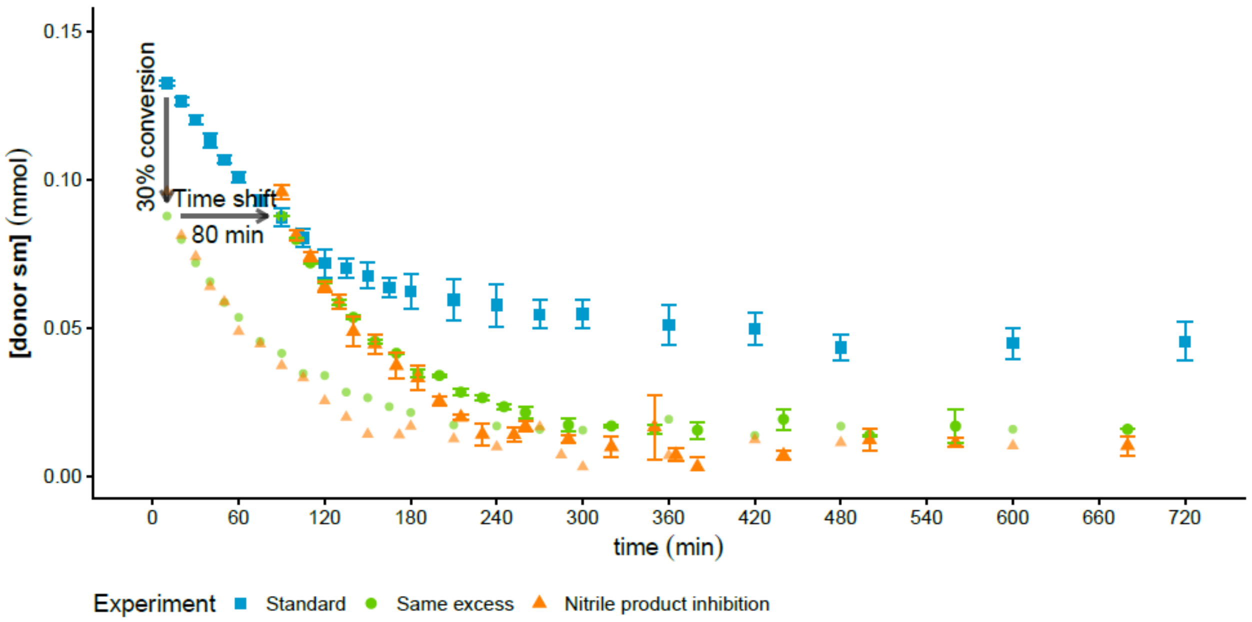

Figure S2 Comparison of the kinetic profiles of the transfer hydrocyanation reaction. The kinetic data of the standard reaction conditions are depicted in blue (■). The kinetic profiles for the same excess experiment (●) and product inhibition experiment (▲) are shown. Each data point is the average of at least two experiments with the standard deviation of the mean depicted as error bars. The reaction profiles indicate that the transfer hydrocyanation reaction suffers from catalyst deactivation, indicated by the overlay of both kinetic profiles of the same excess as well as the product inhibition experiment.[12]

b) Order in reagents

The reaction orders in catalyst, alkyne and donor substrate were determined by using both initial rate kinetics as well as variable time normalization graphical analysis (VTNA) described by Blackmond[12,13] and Burés[14].

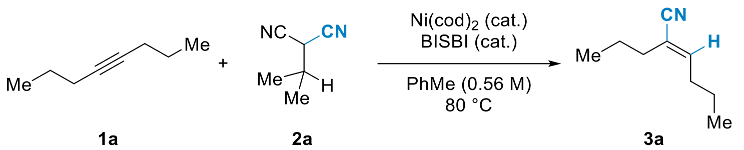

1a **2a** **3a**

General procedure:

Under an argon atmosphere in a glovebox in an oven-dried 4 mL screw-cap vial, a stock solution was prepared containing 2-isopropylmalononitrile **2a** (160 mg, 0.150 mmol, 1.0 equiv), anhydrous toluene (0.5 mL), and 4-octyne **1a** (0.33 mL, 0.23 mmol, 1.5 equiv). The mixture was then divided by taking each time 0.08 mL of the stock solution and adding it to oven-dried 4 mL screw-cap vials containing a magnetic stirring bar (set-up for nine reactions). In two separate vials equipped with stirring bars, BISBI (83 mg, 0.015 mmol, 10 mol%), and Ni(cod)$_2$ (41 mg, 0.015 mmol, 10 mol%) were dissolved in anhydrous toluene (1.0 mL and 1.2 mL, respectively) and the mixtures were stirred until complete dissolution. Then the ligand and catalyst solution were combined and mixed, which led to a dark red precatalyst solution. Then 0.22 mL of the precatalyst solution was added to each of the nine reaction vials. The vials were sealed and removed from the glovebox then heated at 80 °C for the indicated time. After cooling to room temperature, *n*-dodecane (20 µL) was added as an internal standard, the crude mixture was diluted with EtOAc, filtered through a plug of cellulose and then subjected to GC-FID analysis to evaluate the reaction progress by determining the yield of the hydrocyanation product **3a**.

Table S5 Kinetic data of the transfer hydrocyanation under standard reaction conditions using 10 mol% Ni(cod)2 and 10 mol% BISBI.

Entry	Reaction time	GC Yield of 3a [mmol]			
		Run 1	*Run 2*	*Run 3*	*Run 4*
1	10 min	0.000921	0.000204	0.000143	0.000604
2	20 min	0.001817	0.000990	0.000823	0.001342
3	30 min	0.003087	0.002129	0.001860	0.002351
4	40 min	0.004645	0.004367	0.003622	0.003505
5	50 min	0.005556	0.005354	0.004931	0.004657
6	60 min	0.007077	0.007039	0.007040	0.005826
7	70 min	0.008814	0.008312	0.008782	0.007247
8	80 min	0.010052	0.010821	0.010438	0.008751
9	90 min	0.011893	0.012158	0.012160	0.009628

i. Order in catalyst:

The order in nickel precatalyst was determined by varying the concentration of both the Ni(cod)$_2$ and BISBI.

Table S6 Kinetic data of the transfer hydrocyanation catalyzed by 7.5 mol% Ni(cod)$_2$ and 7.5 mol% BISBI.

Entry	Reaction time	GC Yield of 3a [mmol]		
		Run 1	*Run 2*	*Run 3*
1	10 min	0.00051	0.00061	0.000101
2	20 min	0.00117	0.00131	0.000651
3	30 min	0.00194	0.00215	0.001351
4	40 min	0.00307	0.00300	0.002434
5	50 min	0.00365	0.00390	0.004152
6	60 min	0.00457	0.00484	0.004530
7	70 min	0.00561	0.00620	0.005924
8	80 min	0.00675	0.00718	0.007449
9	90 min	0.00776	0.00855	0.008012

Table S7 Kinetic data of the transfer hydrocyanation catalyzed by 12.5 mol% Ni(cod)$_2$ and 12.5 mol% BISBI.

Entry	Reaction time	GC Yield of 3a [mmol]		
		Run 1	*Run 2*	*Run 3*
1	10 min	0.001081	0.001268	0.000167
2	20 min	0.002441	0.002880	0.001142
3	30 min	0.004063	0.004583	0.002871
4	40 min	0.005792	0.006253	0.005232
5	50 min	0.007708	0.007797	0.007043
6	60 min	0.009284	0.009918	0.009240
7	70 min	0.011181	0.012146	0.011430
8	80 min	0.013457	0.013723	0.014018
9	90 min	0.014943	0.016265	0.015933

Table S8 Kinetic data of the transfer hydrocyanation catalyzed by 15 mol% $Ni(cod)_2$ and 15 mol% BISBI.

**Entry**	**Reaction time**	**GC Yield of 3a [mmol]**		
		Run 1	*Run 2*	*Run 3*
1	10 min	0.00139	0.00150	0.000360
2	20 min	0.00325	0.00361	0.001719
3	30 min	0.00515	0.00586	0.004826
4	40 min	0.00755	0.00819	0.007202
5	50 min	0.00970	0.01039	0.009231
6	60 min	0.01176	0.01280	0.011997
7	70 min	0.01405	0.01525	0.015571
8	80 min	0.01623	0.01753	0.019576
9	90 min	0.01877	0.02103	0.022535

The order in precatalyst was then analyzed by using initial rate kinetics.

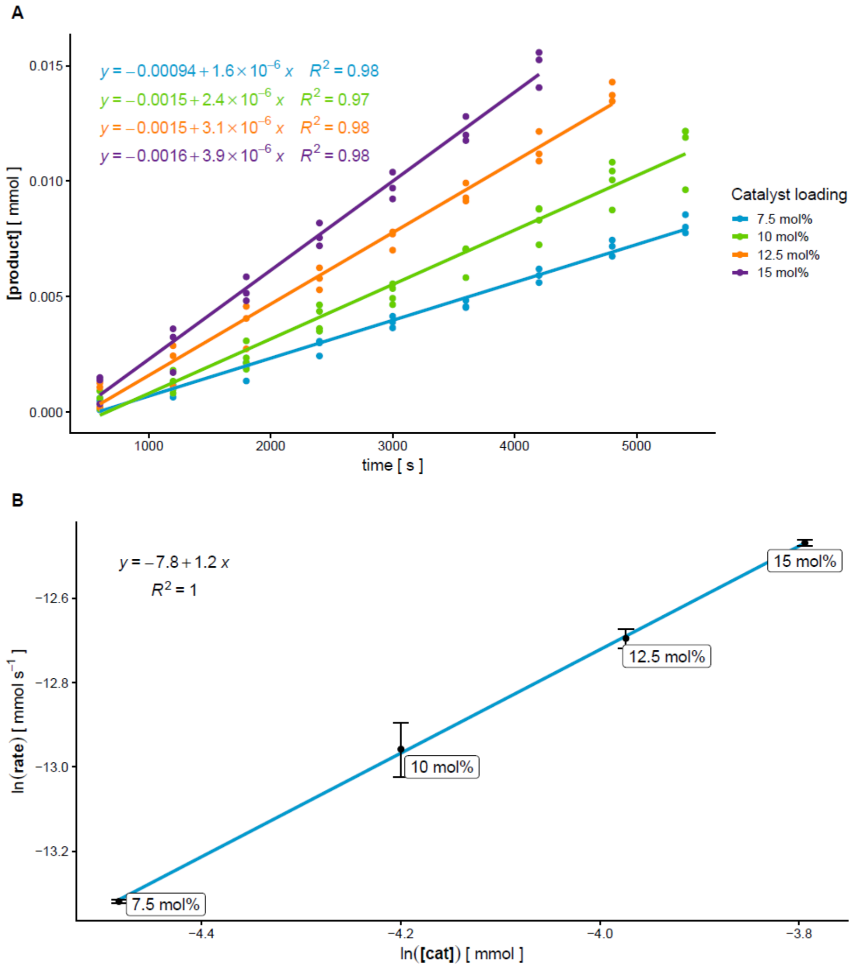

Figure S3 Initial rate kinetics of the transfer hydrocyanation reaction of alkynes at different catalyst concentrations. **(A)** Each data point represents an independent reaction. **(B)** Each data point represents the average of three independent runs with the standard deviation of the mean represented by the error bars. The data were fitted using linear regression after logarithmic calculus, suggesting that the reaction rate is approximately first-order in catalyst concentration (b = 1.23 $\pm$ 0.02).

The same data set was also analyzed using non-linear fitting parameters.

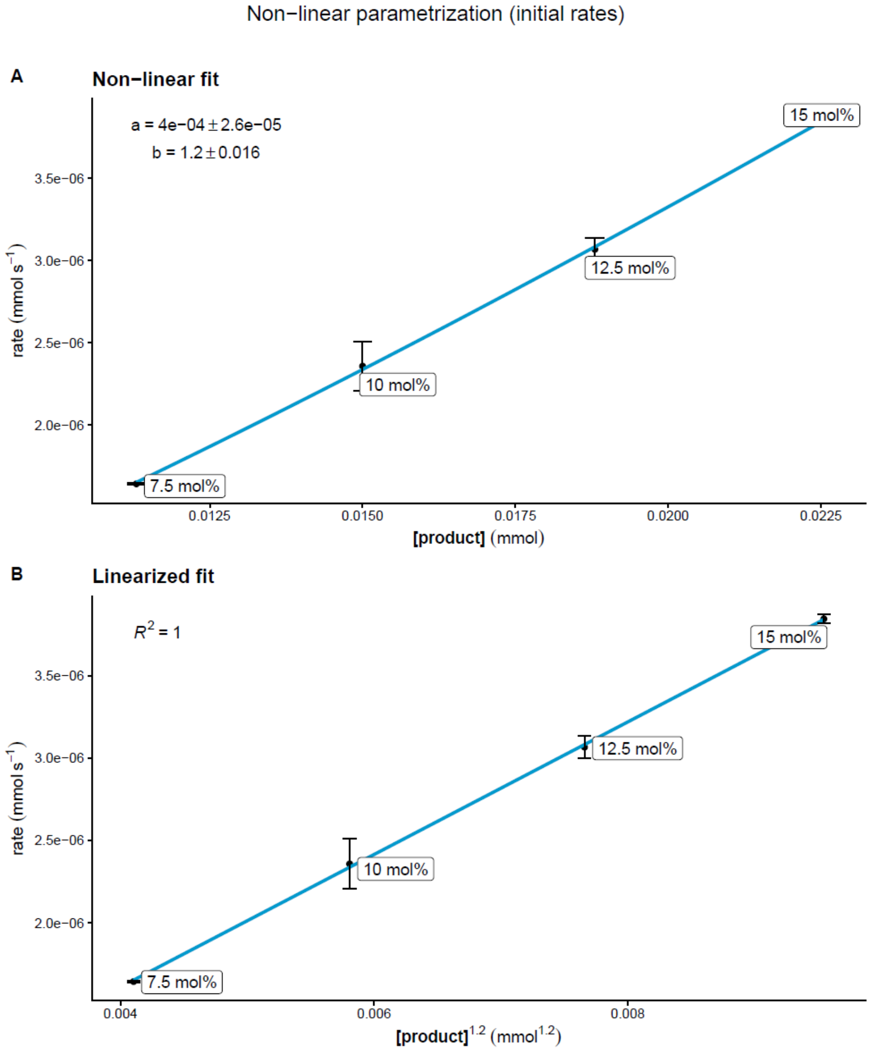

Figure S4 Initial rate kinetics of the transfer hydrocyanation at different catalyst concentrations. **(A)** Using a non-linear regression model to obtain fitting parameters. **(B)** Parametrization using fitting parameters obtained from non-linear fit.

Analysis of the kinetic data by the VTNA method.

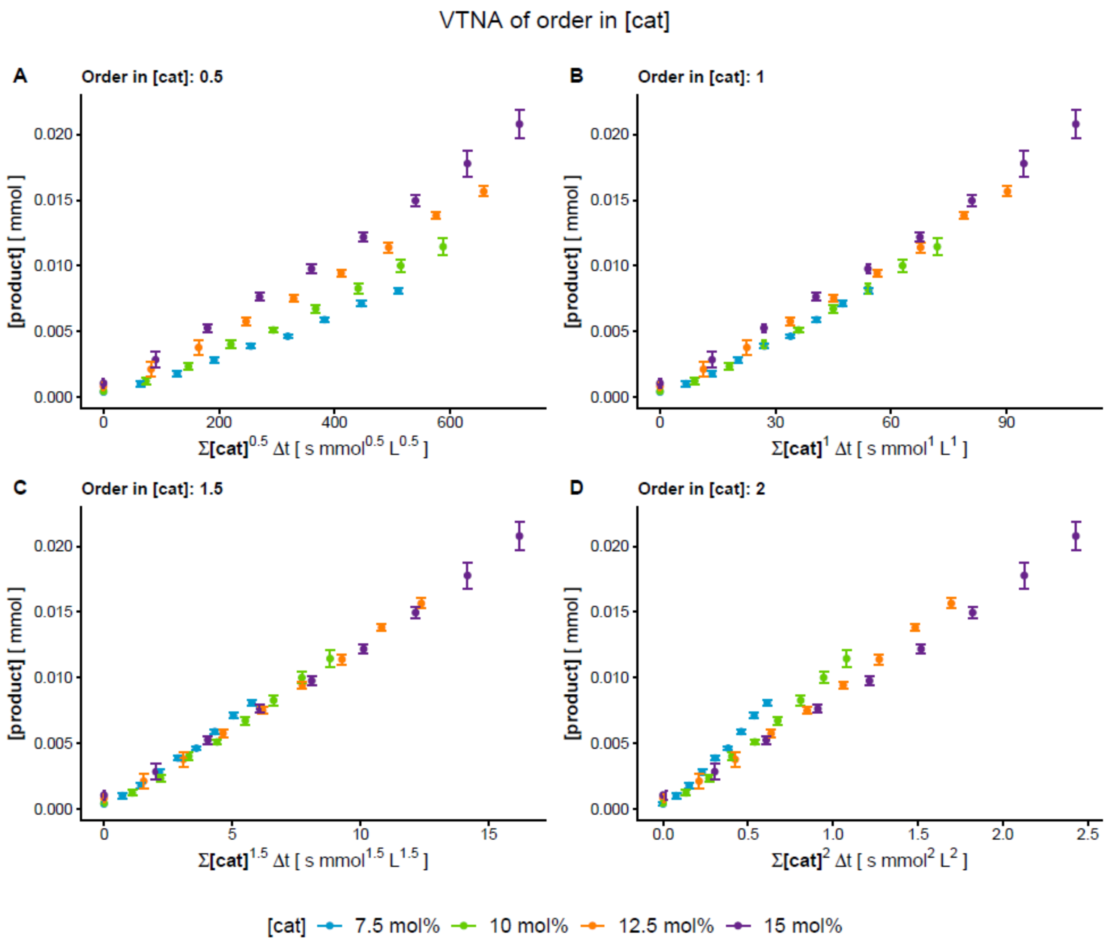

Figure S5 VTNA of the transfer hydrocyanation constructed from the data provided in **Table S5–S8**. **(A)** Assuming a ½-order d ependence of the initial reaction rate on the catalyst concentration. **(B)** Assuming a 1st-order dependence of the initial reaction rate on the catalyst concentration. **(C)** Assuming a 1.5-order dependence of the initial reaction rate on the catalyst concentration. **(D)** Assuming a 2nd-order dependence of the initial reaction rate on the catalyst concentration.

Due to catalyst degradation over time and the limited amount of data points, analysis of the data by VTNA is difficult and not appropriate as the active catalyst concentration can not easily be determined experiementally.[14]

ii. Order in alkyne 1a:

Table S9 Kinetic data of the transfer hydrocyanation using 1.0 equiv of 4-octyne.

Entry	Reaction time	GC Yield of 3a [mmol]		
		Run 1	*Run 2*	*Run 3*
1	10 min	0.000805	0.000749	0.000818
2	20 min	0.002127	0.002045	0.002025
3	30 min	0.00374	0.003553	0.003424
4	40 min	0.005751	0.005095	0.005090
5	50 min	0.007644	0.00665	0.007987
6	60 min	0.009173	0.008439	0.010080
7	70 min	0.011256	0.010617	0.011755
8	80 min	0.013283	0.012632	0.013664
9	90 min	0.014781	0.013528	0.000818

Table S10 Kinetic data of the transfer hydrocyanation using 2.0 equiv of 4-octyne.

Entry	Reaction time	GC Yield of 3a [mmol]	
		Run 1	*Run 2*
1	10 min	0.000451	0.000552
2	20 min	0.001119	0.001193
3	30 min	0.001785	0.001887
4	40 min	0.002473	0.002712
5	50 min	0.003272	0.003549
6	60 min	0.004257	0.004384
7	70 min	0.005022	0.005098
8	80 min	0.005972	0.006085
9	90 min	0.006762	0.006884

The initial rate dependence on the alkyne concentration was then analyzed by using initial rate kinetics.

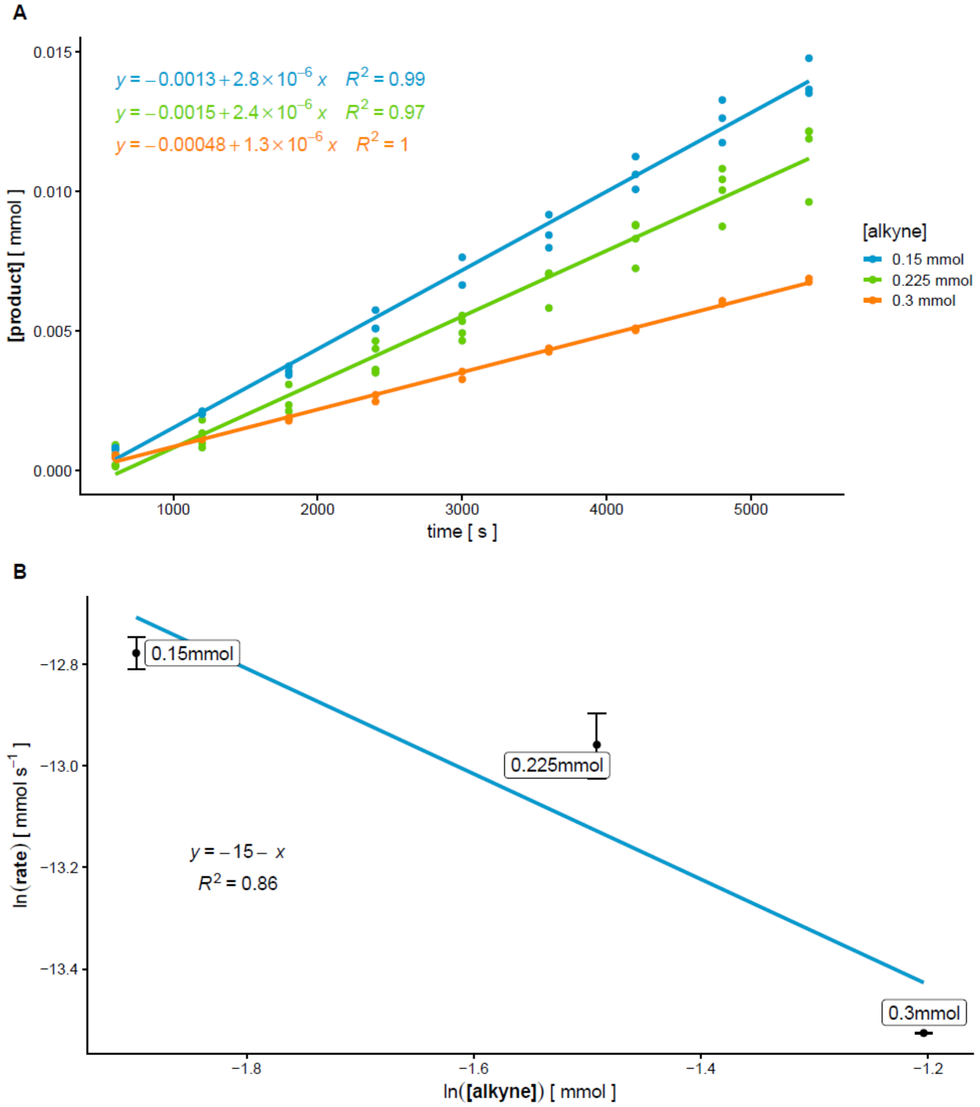

Figure S6 Initial rate kinetics of the transfer hydrocyanation at different alkyne concentrations. **(A)** Each data point represents an independent reaction. **(B)** Each data point represents the average of two or three independent runs with the standard deviation of the mean represented by the error bars. The data were fitted using linear regression after logarithmic calculus, suggesting that the reaction rate is approximately inverse first-order in alkyne concentration (b = –1.04 ± 0.43).

The same data set was also analyzed using non-linear fitting parameters.

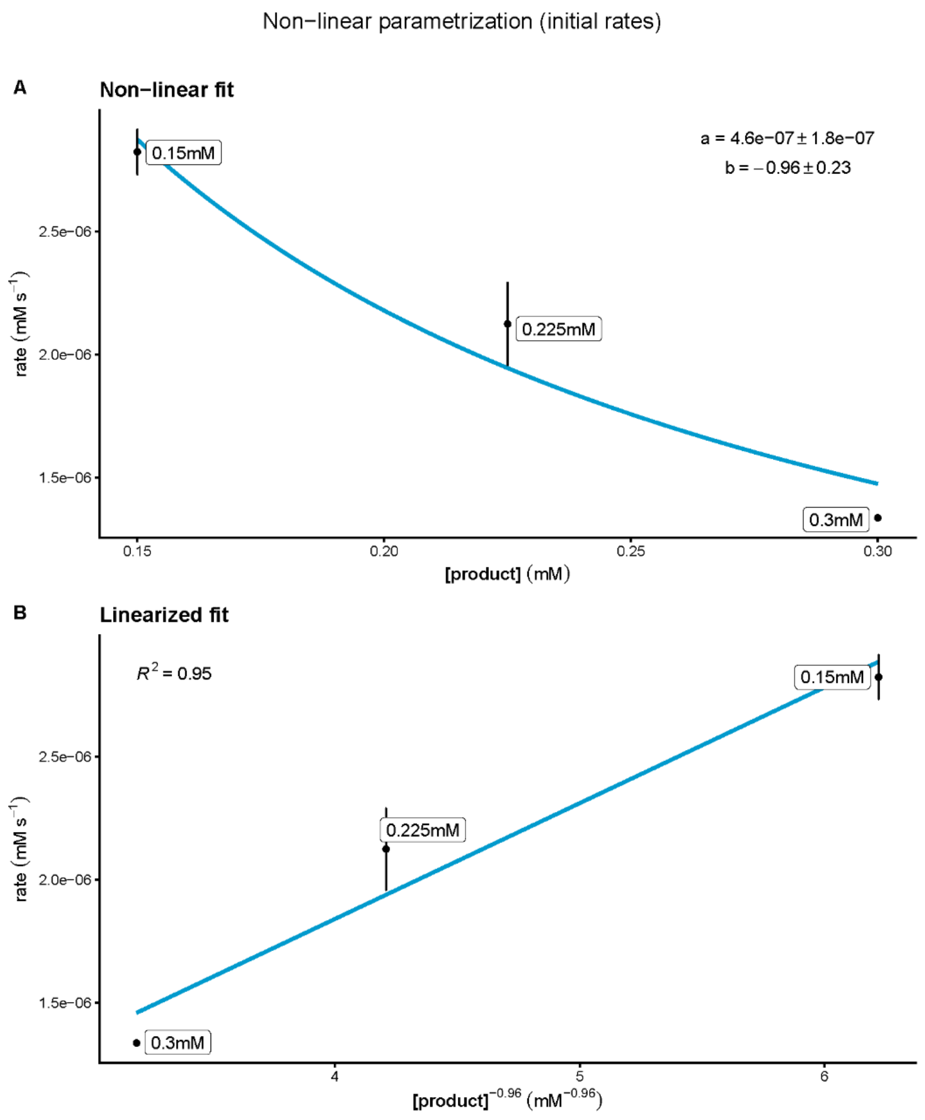

Figure S7 Initial rate kinetics of the hydrocyanation reaction at different alkyne concentrations. (A) Using a non-linear regression model to obtain fitting parameters. (B) Parametrization using fitting parameters obtained from non-linear fit.

Analysis of the kinetic data by the VTNA method.

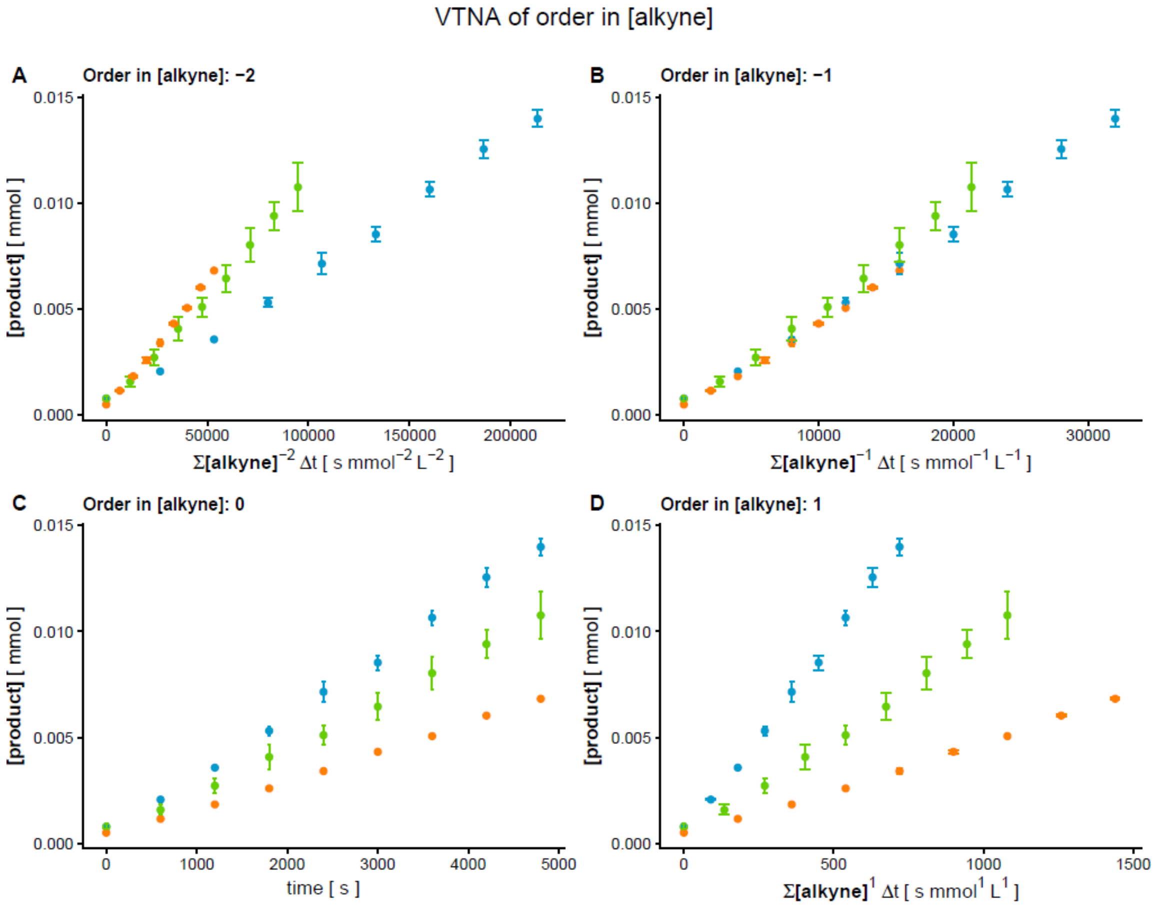

Figure S8 VTNA of the transfer hydrocyanation of alkynes constructed from the data provided in **Table S5, S9,** and **S10**. **(A)** Assuming an inverse 2nd-order of the initial reaction rate on the alkyne concentration. **(B)** Assuming an inverse 1st-order dependence of the initial reaction rate on the alkyne concentration. **(C)** Assuming a 0th-order dependence of the initial reaction rate on the alkyne concentration. **(D)** Assuming a 1st-order dependence of the initial reaction rate on the alkyne concentration.

iii. Order in donor substrate 2a:

Table S11 Kinetic data of the transfer hydrocyanation using 0.5 equiv of 2-isopropylmalononitrile **2a**.

Entry	Reaction time	GC Yield of 3a [mmol]	
		Run 1	*Run 2*
1	10 min	0.0003	0.0003
2	20 min	0.0007	0.0007
3	30 min	0.0011	0.0012
4	40 min	0.0016	0.0016
5	50 min	0.002	0.0022
6	60 min	0.0027	0.0027
7	70 min	0.0032	0.0034
8	80 min	0.0037	0.0038
9	90 min	0.0042	0.0045

Table S12 Kinetic data of the transfer hydrocyanation using 1.5 equiv of 2-isoproplymalononitrile **2a**.

Entry	Reaction time	GC Yield of 3a [mmol]		
		Run 1	*Run 2*	*Run3*
1	10 min	0.0010	0.0008	0.0008
2	20 min	0.0026	0.0021	0.0021
3	30 min	0.0042	0.0036	0.0023
4	40 min	0.0059	0.0050	0.0049
5	50 min	0.0080	0.0068	0.0064
6	60 min	0.0100	0.0082	0.0084
7	70 min	0.0114	0.0099	0.0097
8	80 min	0.0135	0.0115	0.0114
9	90 min	0.0154	0.0140	0.0135

The initial rate dependence on the donor **2a** concentration was then analyzed by using initial rate kinetics.

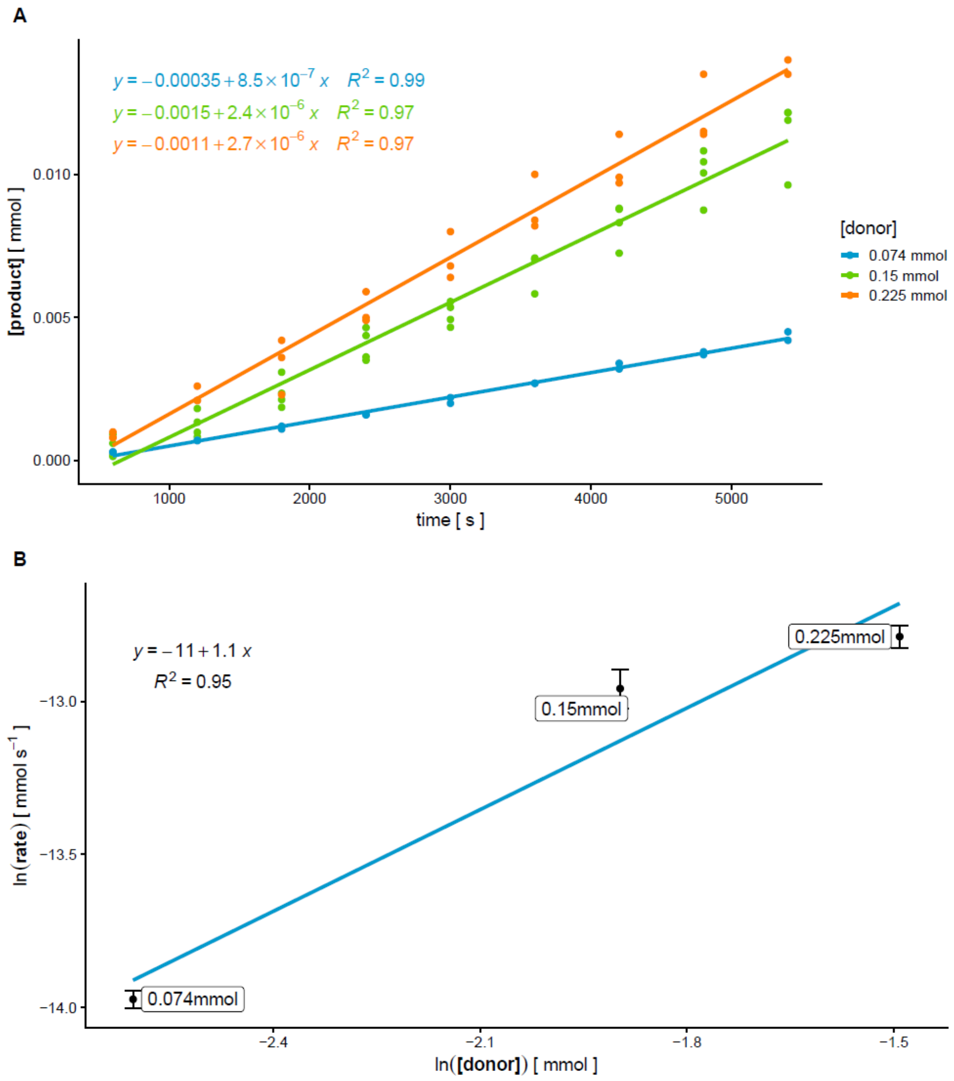

Figure S9 Initial rate kinetics of the transfer hydrocyanation at different donor concentrations. **(A)** Each data point represents an independent reaction. **(B)** Each data point represents the average of two or three independent runs with the standard deviation of the mean represented by the error bars. The data were fitted using linear regression after logarithmic calculus suggesting that the initial reaction rate is approximately first-order dependent on the donor concentration (b = 1.11 ± 0.27).

The same data set was also analyzed using non-linear fitting parameters.

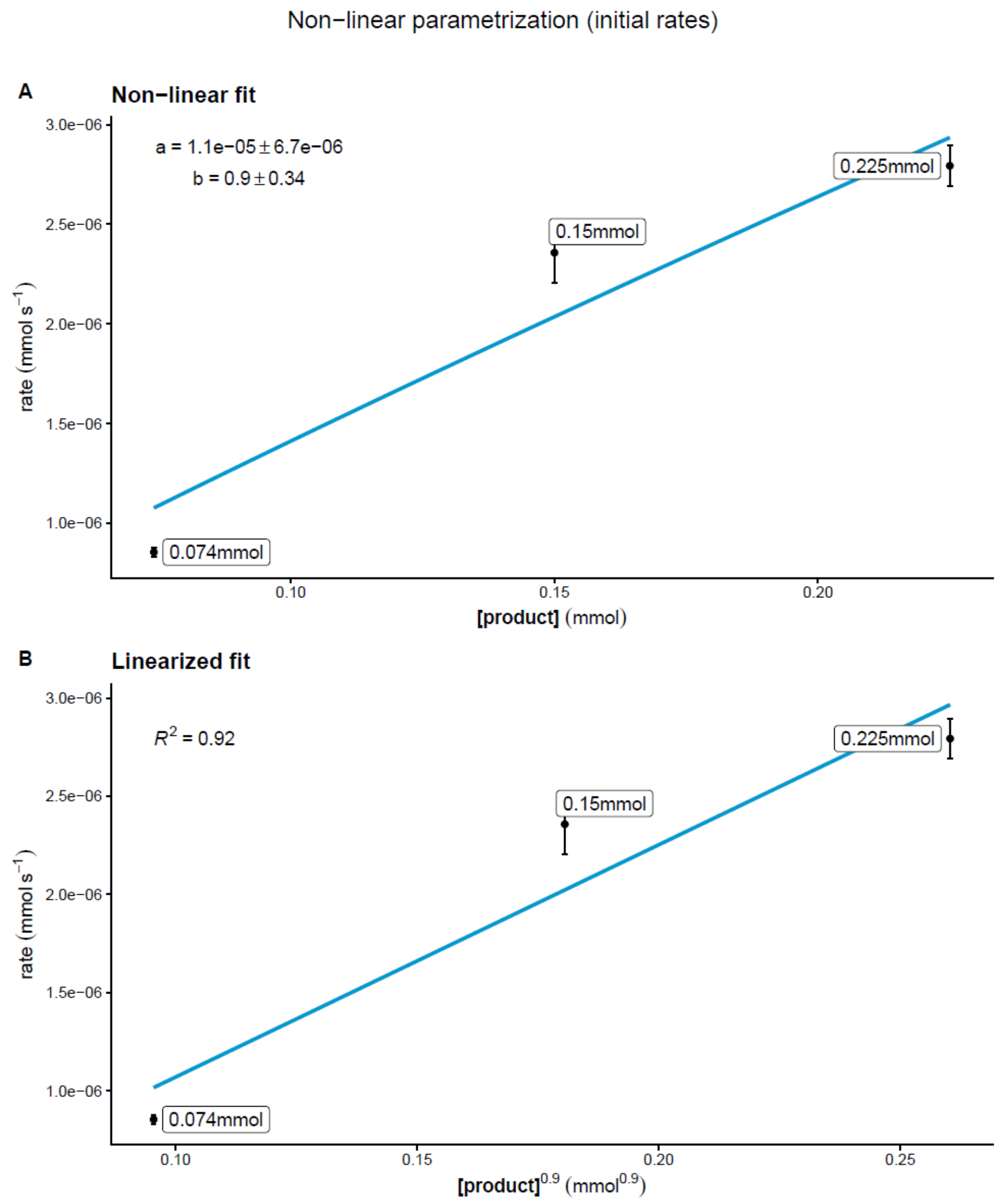

Figure S10 Initial rate kinetic of the transfer hydrocyanation at different donor concentrations. **(A)** Using a non-linear regression model to obtain fitting parameters. **(B)** Parametrization using fitting parameters obtained from non-linear fit.

Analysis of the kinetic data by the VTNA method.

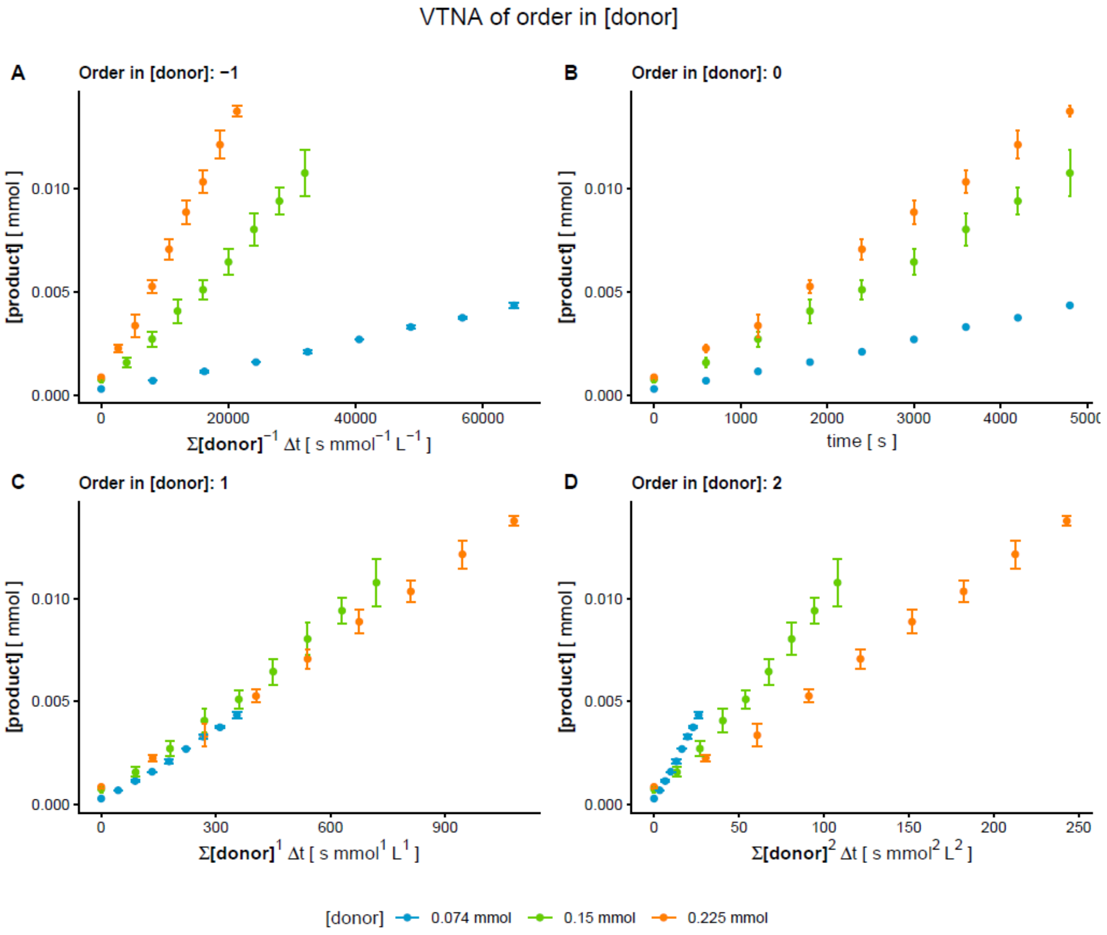

Figure S11 VTNA of the transfer hydrocyanation constructed from the data given in **Table S5, S11, S12**. **(A)** Assuming an inverse 1st-order dependence of the initial reaction rate on the donor concentration. **(B)** Assuming a 0th-order dependence of the initial reaction rate on the donor concentration. **(C)** Assuming a 1st-order dependence of the initial reaction rate on the alkyne concentration. **(D)** Assuming a 2nd-order dependence of the initial reaction rate on the donor concentration.

8. Kinetic isotope effects

a) Differentiation between α-H and β-H: Evaluation of reaction products

β-H transfer:

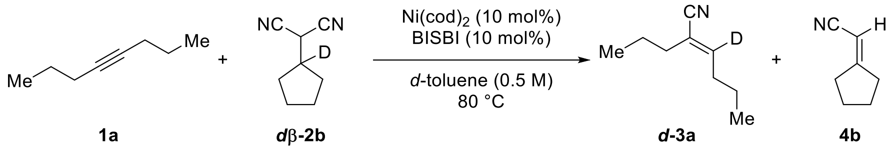

1a **dβ-2b** ***d*-3a** **4b**

To an oven-dried 4 mL screw-cap vial, 2-(cyclopentyl-1-*d*)malononitrile ***d*$_b$-2b** (34 mg, 0.25 mmol, 1.0 equiv), anhydrous toluene (0.05 mL), and 4-octyne **1a** (37 μL, 0.25 mmol) were added under an argon atmosphere in a glovebox. In another oven-dried vial, BISBI (14 mg, 0.025 mmol, 10 mol%), and Ni(cod)$_2$ (6.9 mg, 0.025 mmol, 10 mol%) were dissolved in anhydrous toluene (0.45 mL) and the mixture was stirred until complete dissolution (solution turned dark red). The precatalyst solution was then added to the starting materials in one portion. The vial was sealed and removed from the glovebox then heated at 80 °C for 24 hours. After cooling to room temperature, the crude mixture was concentrated under reduced pressure and purified by flash column chromatography (SiO_2, 0–10% Et_2O in pentanes) to give the product ***d*-3a** as a colorless oil (yield = 27 mg, 77% with >90% D incorporation) and byproduct **4b** as a colorless oil (yield = 19 mg, 70% with <5% D incorporation).

Product (***d*-3a**)

^{1}H NMR (600 MHz, $CDCl_3$) δ 2.20 – 2.13 (m, 4H), 1.57 (h, *J* = 7.4 Hz, 2H), 1.49 – 1.42 (m, 2H), 0.94 (dt, *J* = 8.6, 7.4 Hz, 6H).

^{2}H NMR (92 MHz, $CDCl_3$) δ 6.38 (s, 1H).

^{13}C NMR (101 MHz, $CDCl_3$) δ 148.2 (t), 120.2, 114.8, 30.3, 30.3, 21.8, 21.3, 13.7, 13.4.

HRMS: m/z for $C_9H_{14}N$ $[M]^+$ calc.138.1262, found 138.1261.

Byproduct (**4b**)

^{1}H NMR (600 MHz, $CDCl_3$) δ 5.24 – 5.22 (m, 1H), 2.63 – 2.57 (m, 2H), 2.48 – 2.43 (m, 2H), 1.84 – 1.73 (m, 4H).

^{13}C NMR (101 MHz, $CDCl_3$) δ 174.3, 117.5, 90.6, 34.7, 33.7, 26.5, 26.0.

HRMS: m/z for $C_9H_{14}N$ $[M]^+$ calc. 107.0730, found 107.0726.

α-H transfer:

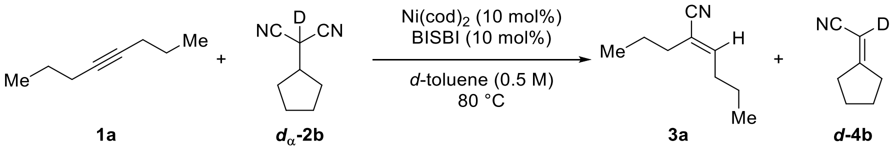

1a ***d*$_α$-2b** **3a** ***d*-4b**

To an oven-dried 4 mL screw-cap vial, 2-cyclopentylmalononitrile-*d* ***d*$_a$-2b** (34 mg, 0.25 mmol, 1.0 equiv), anhydrous toluene (0.05 mL), and 4-octyne **1a** (37 μL, 0.25 mmol) were added under an argon atmosphere in a glovebox. In another oven-dried vial, BISBI (14 mg, 0.025 mmol, 10 mol%) and Ni(cod)$_2$ (6.9 mg, 0.025 mmol, 10 mol%) were dissolved in anhydrous toluene (0.45 mL) and the mixture was stirred until complete dissolution (solution turned dark red). The precatalyst solution was then added to the starting materials in one portion. The vial was sealed and removed from the glovebox then heated at 80 °C for 24 hours. After cooling to room temperature, the crude mixture was concentrated and purified by flash column chromatography (SiO_2, 0–10% Et_2O in pentanes) to give the product **3a** (yield = 29.5 mg, 86% with >95% H incorporation) and byproduct ***d*-4b** as colorless oils (yield = 23.3 mg, 86% with 73% D incorporation).

Product (**3a**)

^{1}H NMR (600 MHz, $CDCl_3$) δ 6.33 (t, *J* = 7.6 Hz, 1H), 2.21 – 2.11 (m, 4H), 1.61 – 1.52 (m, 2H), 1.48 – 1.39 (m, 2H), 0.93 (dt, *J* = 8.8, 7.4 Hz, 6H).

^{13}C NMR (101 MHz, $CDCl_3$) δ 148.2, 120.3, 115.0, 30.5, 30.4, 21.9, 21.4, 13.8, 13.4.

HRMS: m/z for $C_9H_{14}N$ [M-H]$^+$ calc. 136.1121, found 136.1121.

Byproduct (***d*-4b**)

^{1}H NMR (600 MHz, $CDCl_3$) δ 5.21 (s, 0.27H), 2.62 – 2.54 (m, 2H), 2.47 – 2.42 (m, 2H), 1.81 – 1.71 (m, 4H).

^{2}H NMR (92 MHz, $CDCl_3$) δ 7.24 (s).

^{13}C NMR (101 MHz, $CDCl_3$) δ 174.1, 117.5, 90.5, 34.6, 33.6, 26.4, 25.9.

HRMS: m/z for C_7H_8DN [M]$^+$ calc. 108.0792, found 108.0790.

Control reaction:

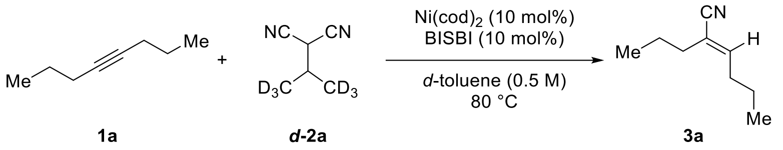

To an oven-dried 4 mL screw-cap vial, 2-(propanyl-1,1,1,3,3,3-d_6)malononitrile ***d*-2a** (29 mg, 0.25 mmol, 1.0 equiv), anhydrous toluene (0.05 mL), and 4-octyne **1a** (37 μL, 0.25 mmol) were added under an argon atmosphere in a glovebox. In another oven-dried vial, BISBI (14 mg, 0.025 mmol, 10 mol%) and Ni(cod)$_2$ (6.9 mg, 0.025 mmol, 10 mol%) were dissolved in anhydrous toluene (0.45 mL) and the mixture was stirred until complete dissolution (solution turned dark red). The precatalyst solution was then added to the starting materials in one portion. The vial was sealed and removed from the glovebox then heated at 80 °C for 24 hours. After cooling to room temperature, the crude mixture was concentrated and purified by flash column chromatography (SiO_2, 0–10% Et_2O in pentanes) to give the product **3a** as a colorless oil (yield = 25 mg, 73% with >95% H incorporation).

Product (**3a**)

^{1}H NMR (600 MHz, $CDCl_3$) δ 6.33 (t, J = 7.6 Hz, 1H), 2.19 – 2.11 (m, 4H), 1.56 (h, J = 7.4 Hz, 2H), 1.44 (h, J = 7.4 Hz, 2H), 0.96 – 0.89 (m, 6H).

^{13}C NMR (101 MHz, $CDCl_3$) δ 148.2, 120.3, 115.0, 30.5, 30.4, 21.9, 21.4, 13.8, 13.4.

HRMS: m/z for $C_9H_{14}N$ [M-H]$^+$ calc. 136.1121, found 136.1122.

b) H/D kinetic isotope effects – parallel reactions (β-H transfer)

Reaction #1

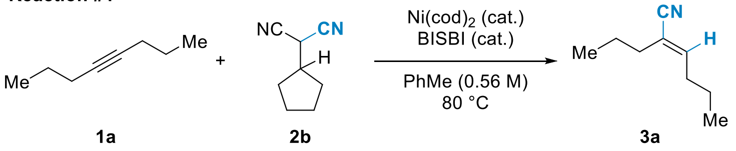

Reaction #2

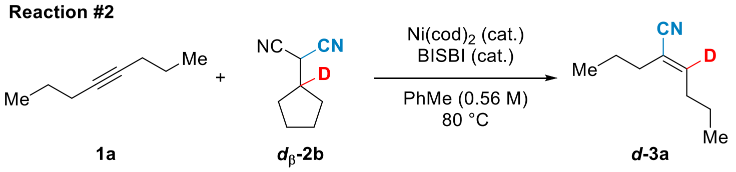

General procedure:

Reaction #1

Under an argon atmosphere in a glovebox in an oven-dried 4 mL screw-cap vial, a stock solution was prepared containing 1H-cyclopentylmalononitrile **2b** (220 mg, 0.150 mmol per reaction, 1.0 equiv), anhydrous toluene (0.55 mL), and 4-octyne **1a** (0.36 mL, 0.23 mmol per reaction, 1.5 equiv). The mixture was then divided by taking each time 0.08 mL of the stock solution and adding it to oven-dried 4 mL screw-cap vials containing a magnetic stirring bar (set-up for ten reactions). In two separate vials equipped with stirring bars, BISBI (174 mg, 0.0150 mmol per reaction, 10 mol%) and Ni(cod)$_2$ (86 mg, 0.015 mmol per reaction, 10 mol%) were dissolved in anhydrous toluene (2.1 mL and 2.52 mL, respectively) and the mixtures were stirred until complete dissolution. The ligand and catalyst solution were combined and mixed, which lead to a dark red precatalyst solution. Then 0.22 mL of the precatalyst solution was added to each of the ten reaction vials. The vials were sealed and removed from the glovebox then heated at 80 °C for the indicated time. After cooling to room temperature, tetradecane (20 µL) was added as an internal standard, the crude mixture was diluted with EtOAc, filtered through a plug of cellulose and then subjected to GC-FID analysis to evaluate the reaction progress by determining the yield of the hydrocyanation product **3a**.

Reaction #2

Under an argon atmosphere in a glovebox in an oven-dried 4 mL screw-cap vial, a stock solution was prepared containing 1D-cyclopentylmalononitrile ***d*$_\beta$-2b** (220 mg, 0.150 mmol per reaction, 1.0 equiv), anhydrous toluene (0.55 mL), and 4-octyne **1a** (0.36 mL, 0.23 mmol per reaction, 1.5 equiv). The mixture was then divided by taking each time 0.08 mL of the stock solution and adding it to oven-dried 4 mL screw-cap vials containing a magnetic stirring bar (set-up for ten reactions). In two separate vials equipped with stirring bars, BISBI (174 mg, 0.0150 mmol per reaction, 10 mol%), and Ni(cod)$_2$ (86 mg, 0.015 mmol per reaction, 10 mol%) were dissolved in anhydrous toluene (2.1 mL and 2.52 mL, respectively) and the mixtures were stirred until complete dissolution. The ligand and catalyst solution were combined and mixed, which lead to a dark red precatalyst solution. Then 0.22 mL of the precatalyst solution was added to each of the ten reaction vials. The vials were sealed and removed from the glovebox then heated at 80 °C for the indicated time. After cooling to room temperature, tetradecane (20 µL) was added as an internal standard, the crude mixture was diluted with EtOAc, filtered through a plug of cellulose and then subjected to GC-FID analysis to evaluate the reaction progress by determining the yield of the hydrocyanation product **3a**.

Table S13 Kinetic data of the transfer hydrocyanation – parallel experiments to determine KIE.

**Reaction #1**	**Reaction time**	**GC Yield of 3a [mmol]**	
**Entry**		*Run 1*	*Run 2*
1	10 min	0.011	0.012
2	20 min	0.025	0.027
3	30 min	0.039	0.041
4	40 min	0.056	0.057
5	50 min	0.069	0.073

**Reaction #2**	**Reaction time**	**GC Yield of 3a [mmol]**	
**Entry**		*Run 1*	*Run 2*
1	10 min	0.010	0.010
2	20 min	0.021	0.023
3	30 min	0.033	0.034
4	40 min	0.046	0.048
5	50 min	0.059	0.061

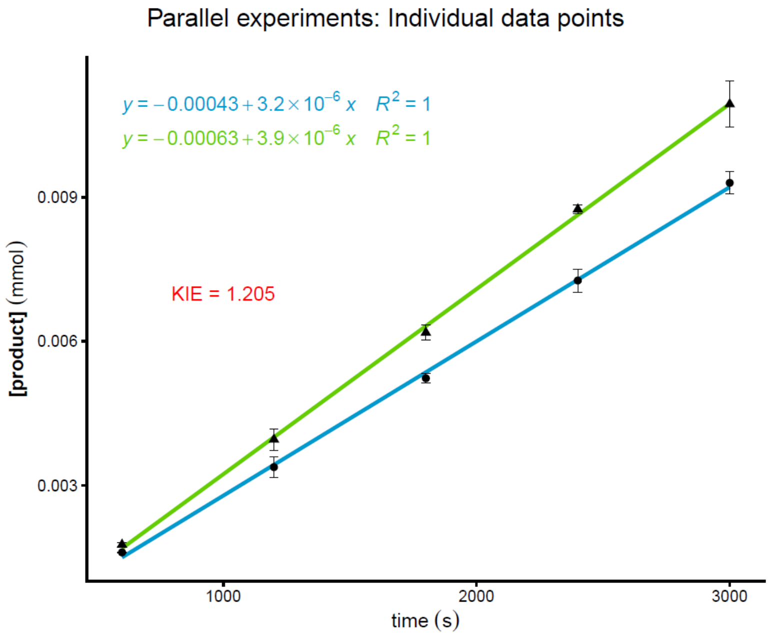

Figure S12 The KIE of the β-H atom in the donor substrate was determined to be $k_H/k_D = 1.20538 \pm 0.00007$ considering 98% (determined by quantitative ^{1}H NMR analysis) of *D* incorporation in the donor substrate.

Possible explanation for observed secondary KIE:

As a deviation from unity for the KIE of the β-H atom is observed, an effect of the β-H atom on the nitrile activation step is proposed. Similar effects were previously reported in literature and referred to as hyperconjugation effects,[15,16] as the rate of the carbocation formation in the α-position was dependent on the bond nature (C–H vs. C–D) in the β-position (Figure S13). Similarly, herein a partially positive charge build-up due to the C(sp^3)–CN bond activation by the nickel catalyst is better stabilized by the hyperconjugation of a C–H bond in the β-position compared to a C–D bond. Thus, this effect can explain the observed secondary KIE.

Hyperconjugation effects in solvolysis of carbocations (*Shiner and Humphrey*, **1963**)

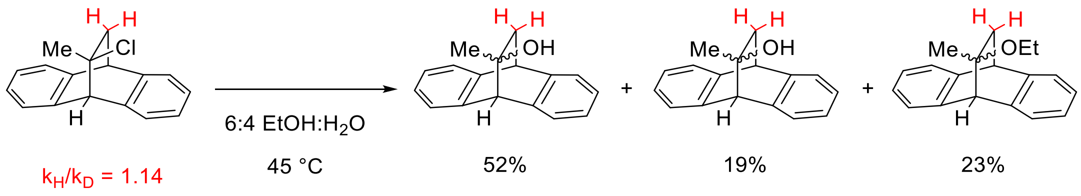

Possible explanation for the secondary KIE$_{(\beta\text{-H atom})}$

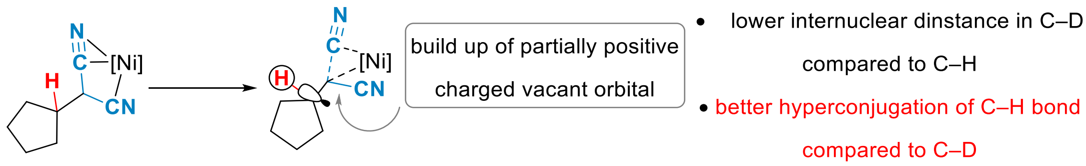

Figure S13 Secondary KIE due to hyperconjugation effects.

c) H/D kinetic isotope effects – parallel reactions (α-H transfer)

Reaction #1

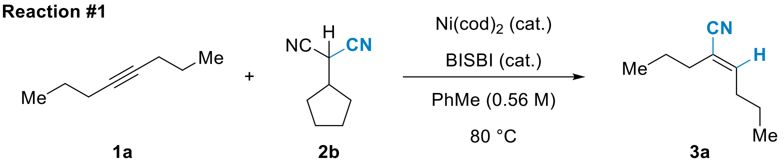

Reaction #2

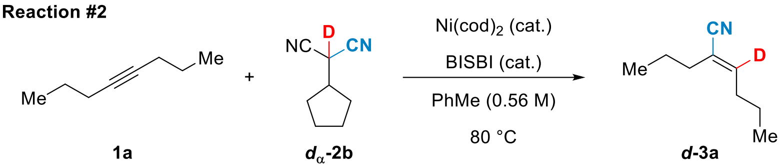

General procedure:

Reaction #1

Under an argon atmosphere in a glovebox in an oven-dried 4 mL screw-cap vial, a stock solution was prepared containing 6H-cyclopentylmalononitrile **2b** (220 mg, 0.15 mmol per reaction, 1.0 equiv), anhydrous toluene (0.55 mL), and 4-octyne **1a** (0.36 mL, 0.23 mmol per reaction, 1.5 equiv). The mixture was then divided by taking each time 0.08 mL of the stock solution and adding it to oven-dried 4 mL screw-cap vials containing a magnetic stirring bar (set-up for ten reactions). In two separate vials equipped with stirring bars, BISBI (174 mg, 0.0150 mmol per reaction, 10 mol%) and $Ni(cod)_2$ (86 mg, 0.015 mmol per reaction, 10 mol%) were dissolved in anhydrous toluene (2.1 mL and 2.52 mL, respectively) and the mixtures were stirred until complete dissolution. The ligand and catalyst solution were combined and mixed, which led to a dark red precatalyst solution. Then 0.22 mL of the precatalyst solution was added to each of the ten reaction vials. The vials were sealed and removed from the glovebox then heated at 80 °C for the indicated time. After cooling to room temperature, tetradecane (20 µL) was added as an internal standard, the crude mixture was diluted with EtOAc, filtered through a plug of cellulose and then subjected to GC-FID analysis to evaluate the reaction progress by determining the yield of the hydrocyanation product **3a**.

Reaction #2

Under an argon atmosphere in a glovebox in an oven-dried 4 mL screw-cap vial, a stock solution was prepared containing 6D-cyclopentylmalononitrile ***d*$_a$-2b** (220 mg, 0.150 mmol per reaction, 1.0 equiv), anhydrous toluene (0.55 mL), and 4-octyne **1a** (0.36 mL, 0.23 mmol per reaction, 1.5 equiv). The mixture was then divided by taking each time 0.08 mL of the stock solution and adding it to oven-dried 4 mL screw-cap vials containing a magnetic stirring bar (set-up for ten reactions). In two separate vials equipped with stirring bars, BISBI (174 mg, 0.015 mmol per reaction, 10 mol%) and $Ni(cod)_2$ (86 mg, 0.015 mmol per reaction, 10 mol%) were dissolved in anhydrous toluene (2.1 mL and 2.52 mL, respectively) and the mixtures were stirred until complete dissolution. The ligand and catalyst solution were combined and mixed, which led to a dark red precatalyst solution. Then 0.22 mL of the precatalyst solution was added to each of the ten reaction vials. The vials were sealed and removed from the glovebox then heated at 80 °C for the indicated time. After cooling to room temperature, tetradecane (20 µL) was added as an internal standard, the crude mixture was diluted with EtOAc, filtered through a plug of cellulose and then subjected to GC-FID analysis to evaluate the reaction progress by determining the yield of the hydrocyanation product **3a**.

Table S14 Kinetic data of the transfer hydrocyanation – parallel experiments to determine KIE.

**Reaction #1**	**Reaction time**	**GC Yield of 3a [mmol]**	
**Entry**		*Run 1*	*Run 2*
1	10 min	0.0107	0.0097
2	20 min	0.0227	0.0226
3	30 min	0.0338	0.0336
4	40 min	0.0518	0.0525
5	50 min	0.0637	0.0618

Reaction #2 Entry	Reaction time	GC Yield of 3a [mmol]	
		Run 1	*Run 2*
1	10 min	0.0097	0.0097
2	20 min	0.0239	0.0240
3	30 min	0.0374	0.0382
4	40 min	0.0568	0.0476
5	50 min	0.0720	0.0616

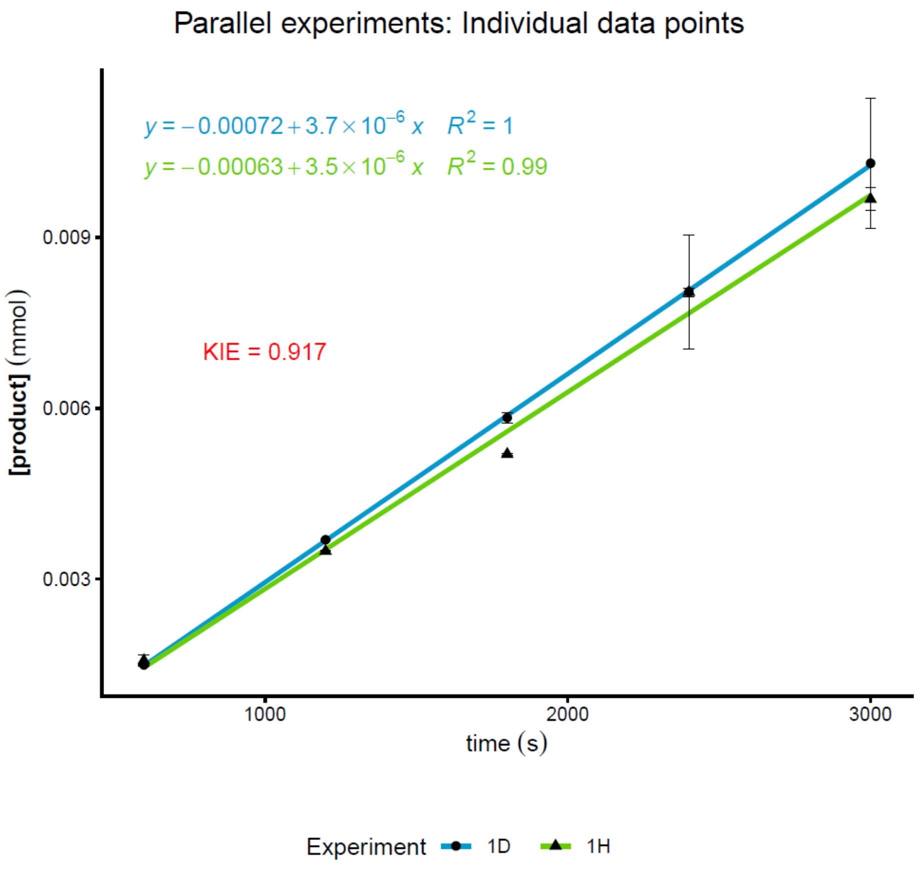

Figure S14 The KIE of the α-H atom in the donor substrate was determined to be $k_H/k_D = 0.9167 \pm 0.0001$ considering 65% (determined by quantitative ^{1}H NMR analysis) of *D* incorporation in the donor substrate.

d) **^{13}C kinetic isotope effect**

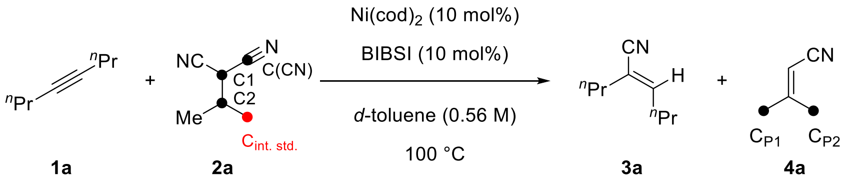

Under an argon atmosphere in a glovebox in an oven-dried 4 mL screw-cap vial, a stock solution was prepared containing 2-isopropylmalononitrile **2a** (40 mg, 0.15 mmol per reaction, 1.0 equiv), anhydrous d_8-toluene (0.13 mL), and 4-octyne **1a** (83 µL, 0.23 mmol per reaction, 1.5 equiv). The mixture was then divided by taking each time 0.08 mL of the stock solution and adding it to oven-dried 4 mL screw-cap vials containing a magnetic stirring bar (set-up for two reactions). In two separate vials equipped with stirring bars, BISBI (20.8 mg, 0.0150 mmol per reaction, 10 mol%) and Ni(cod)$_2$ (10.3 mg, 0.0150 mmol per reaction, 10 mol%) were dissolved in anhydrous d_8-toluene (0.25 mL and 0.30 mL, respectively) and the mixtures were stirred until

complete dissolution. The ligand and catalyst solution were combined and mixed, which led to a dark red precatalyst solution. Then 0.22 mL of the precatalyst solution was added to each of the two reaction vials. The vials were sealed and removed from the glovebox then heated at 100 °C for 7 hours. After cooling to room temperature, the crude mixture was filtered four times over a plug of cellulose using d_8-toluene as an eluent. The filtered crude mixture was subjected to quantitative ^{13}C NMR measurements. ^{13}C measurements were carried out for a total of 4 reactions. The integral of the two methyl groups (**$C_{int.\ std.}$**) in each spectrum was set to 200. All spectra were integrated manually. For each run, the same batch of donor substrate was used and the standard results corresponding to this batch are shown in the **Table S15**. The raw data of each run was processed independently and integrated three times with slightly different phasing and integration regions to account for processing and integration errors. Since all integrals are referenced internally, experimental errors introduced by instrumental instabilities of the NMR spectrometer can be neglected.

To evaluate the ^{13}C KIE of the transfer hydrocyanation of alkynes, the following considerations have been made according to Singleton and co-workers.[17] During the course of the reaction, the more slowly reacting isotopologue of the starting material accumulates if it is involved in the rate-determing step of the reaction. The following relation between the ratio of the abundance of this isotope in the recovered starting material (R) compared to its initial abundance (R_0), the conversion of the starting material (F) and the relative rates for the two isotopologues (kinetic isotope effect: KIE) can be used. Based on this, the KIE can be calculated as follows.

$$\frac{R_0}{R} = (1 - F)^{(1/KIE)-1}$$

$$KIE_{calc} = \frac{ln(1 - F)}{\ln\left[(1 - F)\, {}^{R}\!/_{R_0}\right]}$$

The fractional conversion of the starting material (F) is defined by the amount of recovered starting material (S_r) and product (P_r). The integral of the isopropyl methyl groups in the starting material **2a** was used as internal reference and set to 200 (see above). Thus the following expression could be derived, where $I_{P,Me1}$ and $I_{P,Me2}$ refer to the integrals of the two methyl groups of the alkenyl nitrile byproduct.

$$F = 1 - \frac{S_r}{S_r + P_r}$$

$$F = 1 - \frac{200}{200 + I_{P,Me1} + I_{P,Me2}}$$

All integrations were performed manually. Every sample (run) was measured once. To monitor differences in relaxation behavior between samples, potentially leading to systematic changes in signal intensities (*e.g.* due to the presence of paramagnetic impurities in a subset of the samples), a single delay adiabatic ^{13}C inversion recovery experiment was carried out for each sample (recovery delay: 12 s). According to an initial determination of ^{13}C T1 values in starting material and byproduct (see Figure S16) the interscan delay (d1+ acquisition time) was set to 90s. This ensures complete relaxation of all ^{13}C nuclei involved in our analysis, as the following T1 values for all relevant carbons in the starting material **2a** were determined: C(CN) = 12.7 s, C_α = 7.9 s, C_β = 7.6 s, $C_{int.\ stand.}$ = 4.2 s.

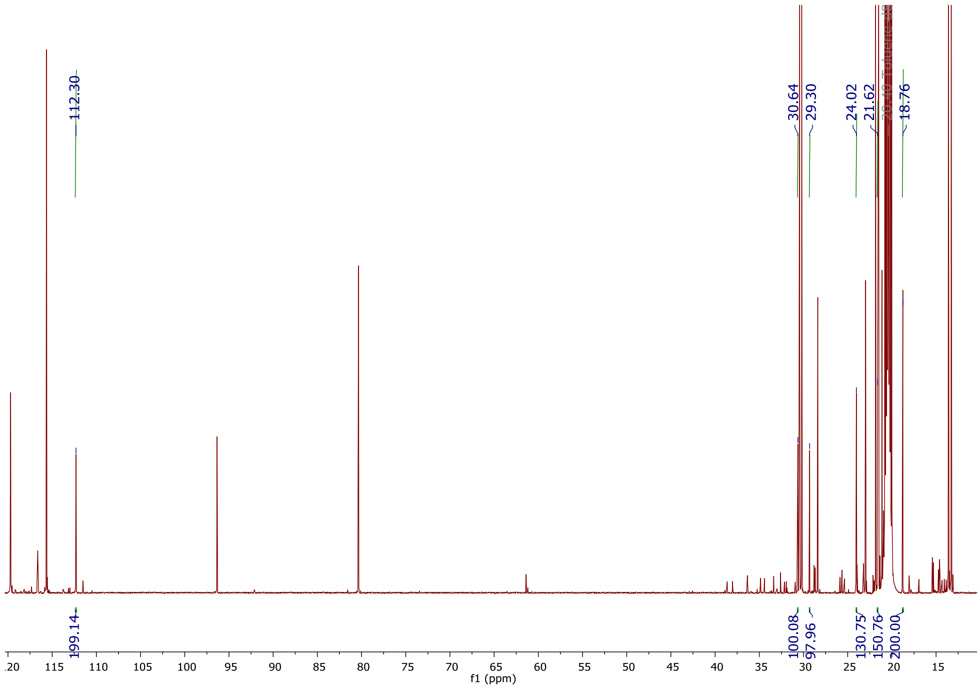

Figure S15 Representative example to evaluate ^{13}C content of the recovered starting material in the crude reaction (151 MHz, d_8-toluene)

The following peaks were considered in the evaluation of the recovered starting material as well as the alkenyl nitrile byproduct.

^{13}C NMR assignments (151 MHz, *d*-toluene)

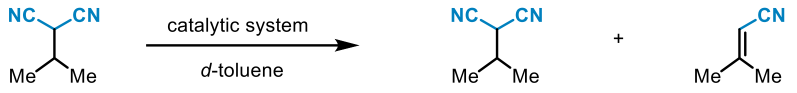

δ(CN) = 112.3 ppm, T1 = 12.7 s

δ(Cα) = 30.6 ppm, T1 = 7.9 s

δ(Cβ) = 29.3 ppm, T1 = 7.6 s

δ(Me) = 18.8 ppm, T1 = 4.2 s

δ(Me1) = 24.0 ppm

δ(Me2) = 21.6 ppm

Figure S16 Assignment of starting material and byproduct in the crude reaction mixture.

Based on these assignments, R/R_0 and F were calculated relative to the methyl peaks of **2a** ($C_{int. stand.}$) after the reaction and in the initial donor batch, as it was assumed that these were not involved in the rate-determining step of the reaction.

Four independent runs were performed and the filtered samples were submitted to quantitative NMR analysis. First, the ^{13}C content of the initial donor batch was evaluated, then the ^{13}C content after the reaction was analyzed. For each sample, the raw data were independently processed and integrated three times to account for processing and integration errors.

Table S15 Integrals of ^{13}C NMR spectrum to determine ^{13}C KIE.

	C(CN)	C1	C2	$C_{int. std.}$	C_{P1}	C_{P2}
**Initial**	194.5	97.4	99.9	200		
	194.2	97.3	98.1	200		
	194.3	97.3	98.2	200		
**Mean Initial**	194.3	97.3	98.7	200		

**Run 1**	199.8	98.8	97.3	200	127.6	122.4
	196.2	100.6	99.1	200	128.0	122.9
	198.1	99.0	97.4	200	126.2	119.0
**Run 2**	206.2	101.5	99.8	200	241.5	267.6
	198.5	101.3	98.2	200	243.4	264.3
	201.7	99.4	99.2	200	241.5	260.4
**Run 3**	205.1	100.1	99.5	200	461.8	451.5
	203.7	100.5	99.1	200	443.7	464.7
	201.5	99.3	99.6	200	438.6	488.4
**Run 4**	200.3	101.6	101.7	200	469.1	483.6
	202.8	99.2	98.1	200	468.1	484.6
	200.4	99.4	98.0	200	467.4	494.9

Based on these initial integrations, we also assumed that the error for the initial integration of the starting material can be neglected, and the mean initial content was used as a reference (R_0), thus the following R/R_0 values were calculated.

Table S16 R/R_0 values.

	Conversion	**C(CN)**	**C1**	**C2**	**$C_{int. std.}$**
**Run 1**	0.5555	1.028130	1.015068	0.985483	1
	0.5564	1.009605	1.033562	1.003714	1
	0.5507	1.019383	1.017123	0.986496	1
**Run 2**	0.7179	1.061063	1.042808	1.010804	1
	0.7173	1.021441	1.040753	0.994598	1
	0.7150	1.037907	1.021233	1.004727	1
**Run 3**	0.8203	1.055403	1.028425	1.007765	1
	0.8195	1.048199	1.032534	1.003714	1
	0.8225	1.036878	1.020205	1.008778	1
**Run 4**	0.8264	1.030703	1.043836	1.030047	1
	0.8264	1.043568	1.019178	0.993585	1
	0.8279	1.031218	1.021233	0.992573	1

Based on these results, the KIE_{calc} for the ^{13}C at natural abundance were calculated based on the equation shown above.

Table S17 Calculated KIE values.

	Conversion [%]	**C(CN)**	**C1**	**C2**	**$C_{int. std.}$**
**Run 1**	55.6	1.0354	1.0188	0.9823	1.0354
	55.6	1.0119	1.0423	1.0046	1.0119
	55.1	1.0246	1.0217	0.9833	1.0246
**Run 2**	71.8	1.0491	1.0343	1.0086	1.0491
	71.7	1.0171	1.0326	0.9957	1.0171
	71.5	1.0305	1.0170	1.0038	1.0305
**Run 3**	82.0	1.0324	1.0166	1.0045	1.0324
	82.0	1.0283	1.0191	1.0022	1.0283
	82.3	1.0214	1.0117	1.0051	1.0214
**Run 4**	82.6	1.0176	1.0251	1.0172	1.0176
	82.6	1.0250	1.0110	0.9963	1.0250
	82.8	1.0178	1.0121	0.9958	1.0178

These results were then used to determine the mean KIE and the standard deviation of the mean KIE.

Table S18 Mean KIE values.

	**C(CN)**	**C1**	**C2**	**$C_{int. std.}$**
**Mean KIE ± std. deviation**	1.026 ± 0.010	1.022 ± 0.010	0.999 ± 0.010	1

Below the integrated raw data are presented for the initial donor batch as well as all the four independent runs.

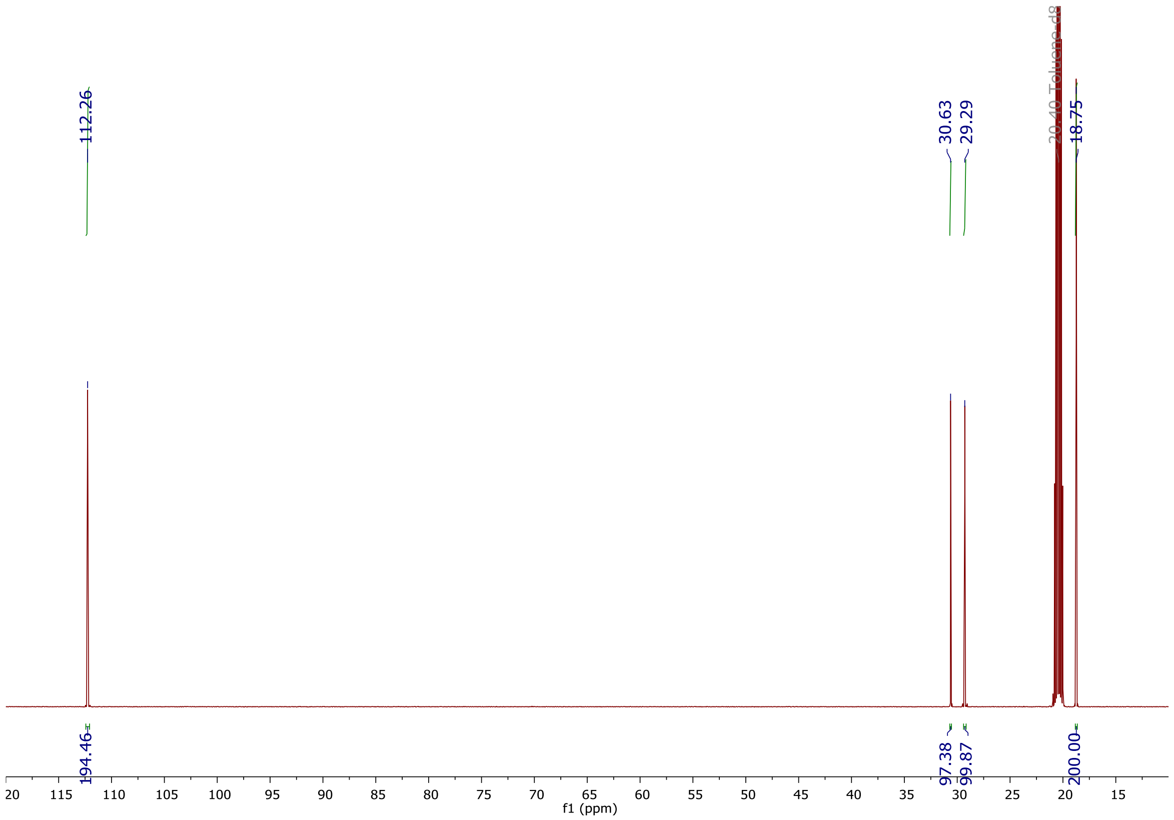

Figure S17 Quantitative ^{13}C NMR of the initial donor batch.

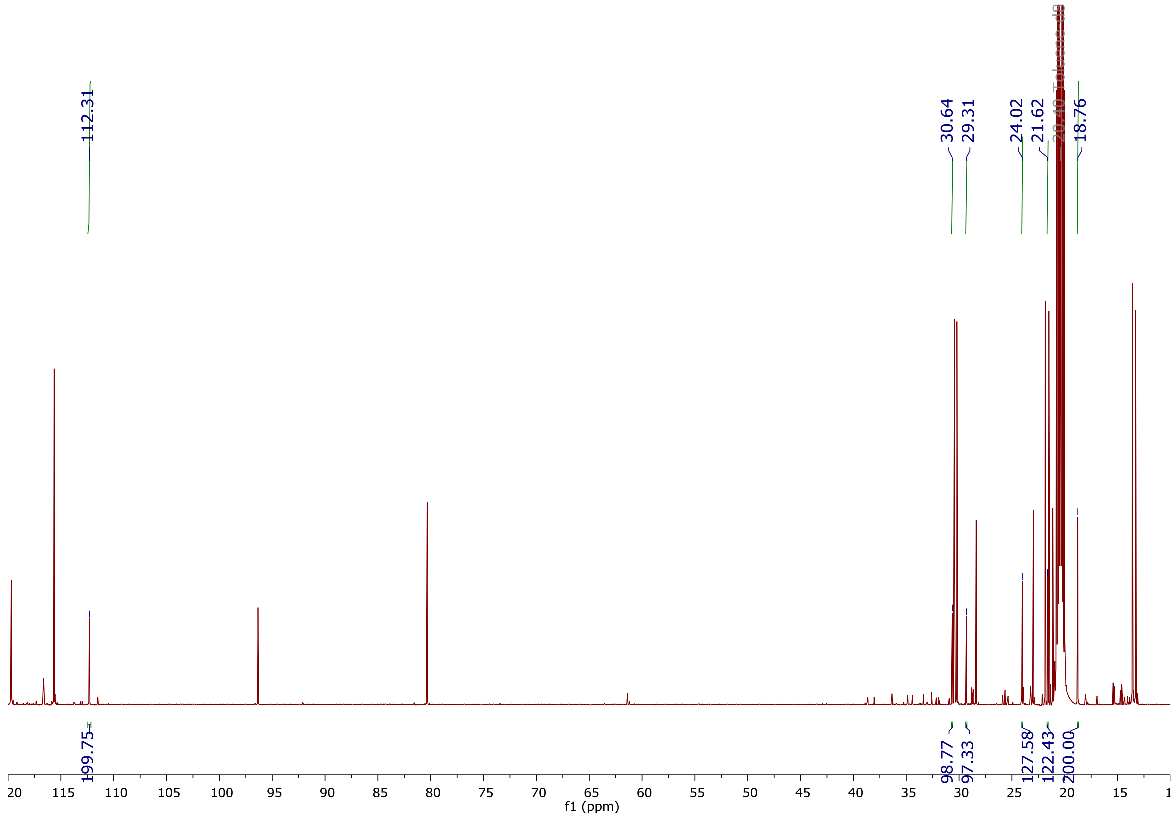

Figure S18 ^{13}C NMR spectrum of run 1. For peak assignments see above.

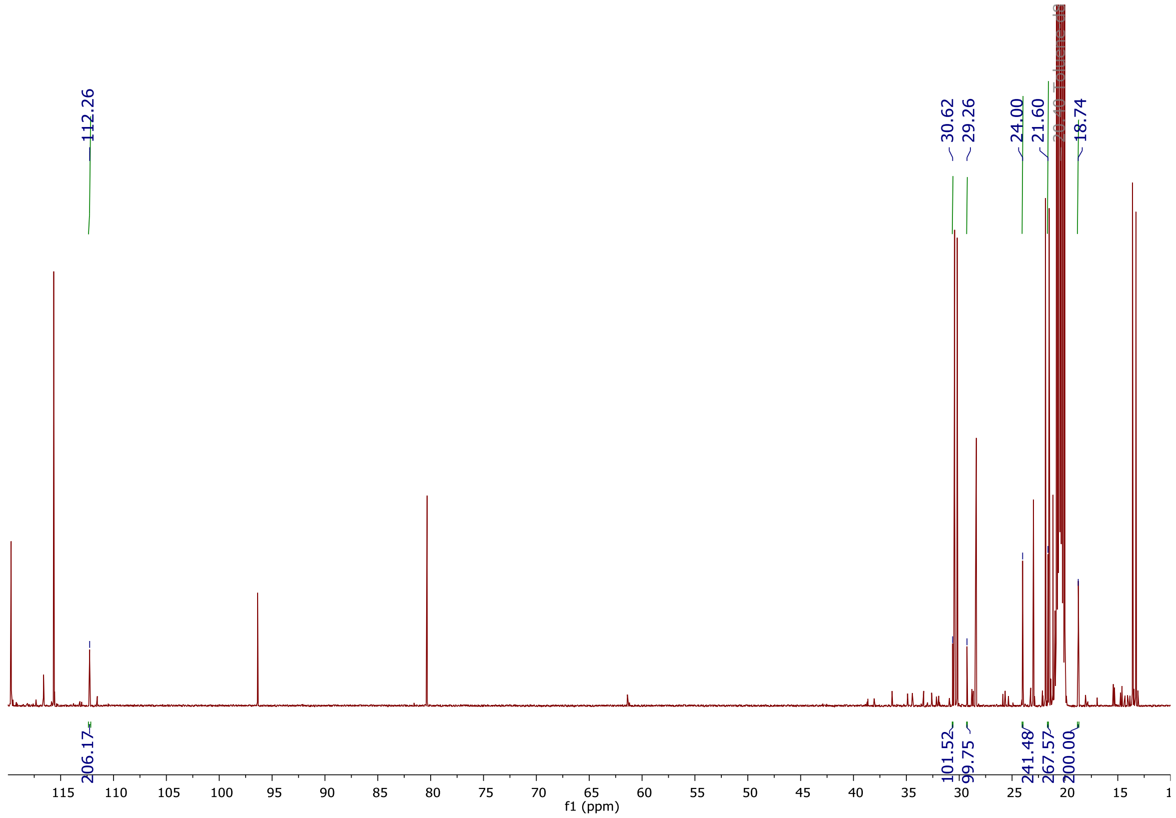

Figure S19 ^{13}C NMR spectrum of run 2. For peak assignments see above.

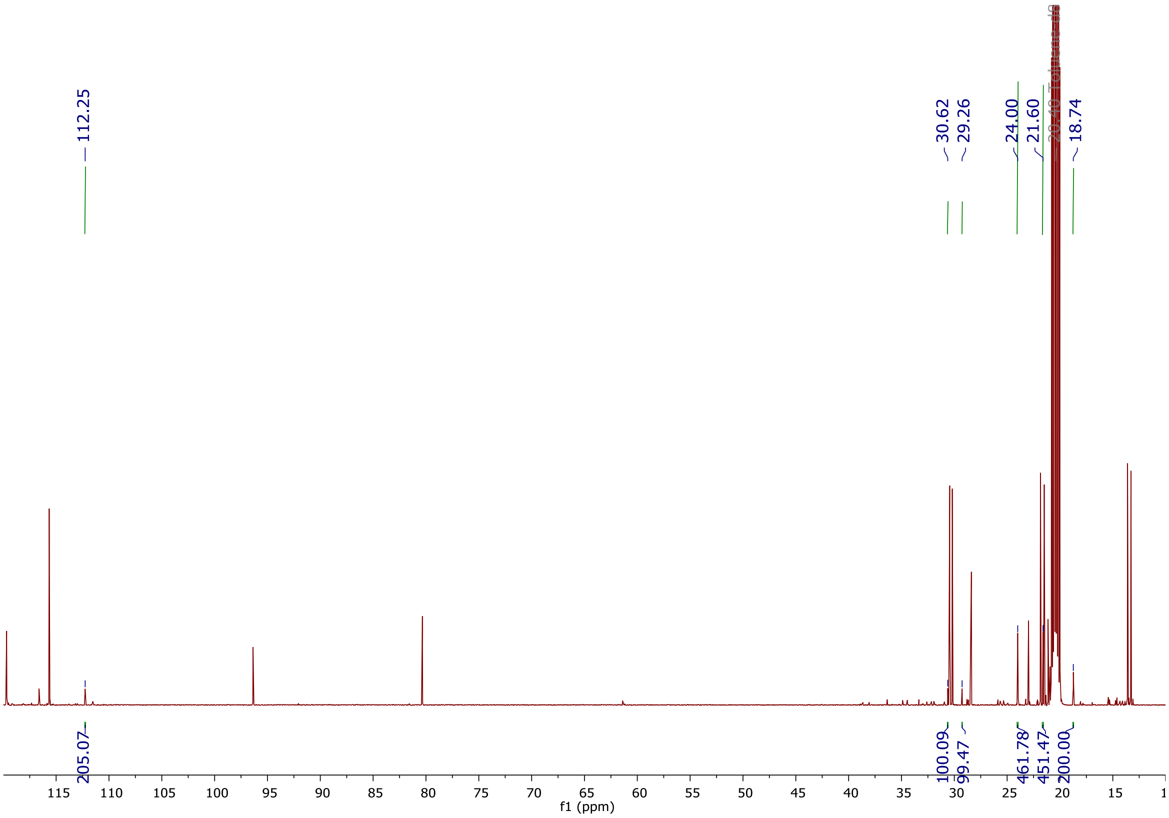

Figure S20 ^{13}C NMR spectrum of run 3. For peak assignments see above.

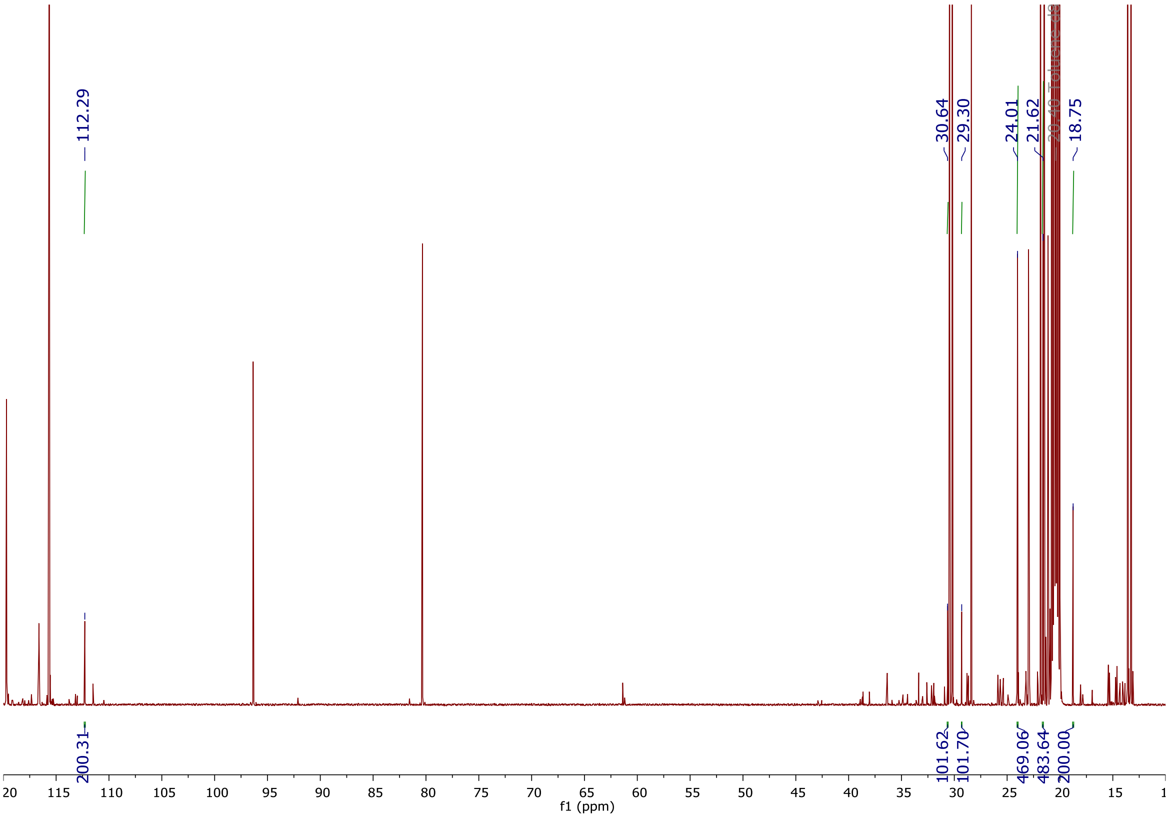

Figure S21 ^{13}C NMR spectrum of run 4. For peak assignments see above.

9. Theoretical derivation of the rate law for the transfer hydrocyanation of alkynes

The theoretical rate law for the transfer hydrocyanation of alkynes in the presence of nickel catalyst was derived by considering the following pathway (see **Scheme d-1**).

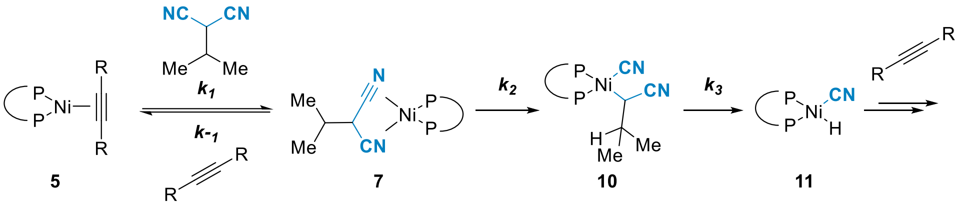

Scheme d-1 Overview of the proposed intermediates in the transfer hydrocyanation of alkynes.

Initially, the rate of the production formation can be described as follows, [D] and [A] refers to donor and alkyne concentration respectively.

$$\frac{dP}{dt} = -\frac{d[11]}{dt} = -\frac{d[10]}{dt} = -\frac{d[7]}{dt} = k_2[7] - k_1[5][D] + k_{-1}\,[7][A]$$

As the rate-determining step of the reaction is the C–CN bond activation (***k***$\boldsymbol{k_2}$), the concentration of intermediate **10** and **11** can be assumed to be neglectable. Therefore, this assumption can be made:

$$[cat] = [5] + [7]$$

The rate of the reaction can then be expressed as follows:

$$\text{rate} = (k_2 + k_{-1}[A])[7] - k_1[cat][D] + k_1[7][D]$$

Assuming **5** is the resting state of the reaction, the following steady-state approximation can be applied ($k_1 < k_{-1}$).

$$\frac{d[5]}{dt} = -k_1[5][D] + k_{-1}\,[7][A] = 0$$

$$= -k_1[5][D] + k_{-1}\,[cat][A] - k_{-1}\,[5][A] = 0$$

$$[7] = \frac{k_1}{k_1[D] + k_{-1}[A]}[cat][D]$$

Based on these assumptions, the rate can be expressed as follows:

$$\text{rate} = (k_2 + k_{-1}[A])\frac{k_1}{k_1[D]+k_{-1}[A]}[cat][D] - k_1[cat][D] + \frac{k_1^2}{k_1[D]+k_{-1}[A]}[cat][D]^2$$

$$\text{rate} = (k_2 + k_{-1}[A])\frac{k_1}{k_1[D]+k_{-1}[A]}[cat][D] - k_1[cat][D] + \frac{k_1^2}{k_1[D]+k_{-1}[A]}[cat][D]^2$$

$$\text{rate} = \frac{k_1k_2[cat][D]+k_1k_{-1}[A][cat][D]+k_1^2[cat][D]^2-k_1k_{-1}[A][cat][D]-k_1^2[cat][D]^2}{k_1[D]+k_{-1}[A]}$$

Finally, introducing an equilibrium constant K, the rate can be simplified to:

$$\text{rate} = \frac{k_1k_2[cat][D]}{k_1[D]+k_{-1}[A]} = \frac{Kk_2[cat][D]}{K[D]+[A]} \quad \text{with} \quad K = \frac{k_1}{k_{-1}}$$

For $k_{-1} > k_1$, the following expression can be derived:

$$\text{rate} = \frac{Kk_2[cat][D]}{[A]}$$

10. Synthesis of nickel complexes

Synthesis of [(BISBI)Ni(cod)]

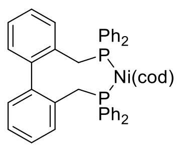

In a glovebox under an argon atmosphere, $Ni(cod)_2$ (6.9 mg, 0.025 mmol, 1.0 equiv) and BISBI (14 mg, 0.025 mmol, 1.0 equiv) were dissolved in *d*$_8$-toluene (0.5 mL) in an NMR tube. Then the tube was sealed with a rubber septum. The NMR tube was removed from the glovebox and sonicated for 2 minutes to ensure complete dissolution. Then the NMR spectra were recorded. X-ray quality crystals were obtained from this saturated toluene solution kept at room temperature overnight.

^{1}H NMR (500 MHz, *d*$_8$-toluene) δ 7.65 – 7.57 (m, 4H), 7.15 – 7.08 (m, 7H), 7.05 – 6.94 (m, 5H), 6.93 – 6.86 (m, 4H), 6.85 – 6.79 (m, 4H), 6.76 – 6.67 (m, 4H), 4.45 – 4.33 (m, 2H), 4.17 (dd, J = 14.0, 7.0 Hz, 2H), 3.98 (q, J = 8.6 Hz, 2H), 3.78 (dd, J = 14.0, 9.5 Hz, 2H), 1.69 – 1.49 (m, 4H), 1.34 – 1.18 (m, 2H), 0.55 – 0.39 (m, 2H). NOTE: residues of free cod ligand are present.

^{13}C NMR (126 MHz, *d*$_8$-toluene) δ 145.1 (dd, J = 21.2, 6.0 Hz), 142.4 (d, J = 22.6 Hz), 141.9 (d, J = 3.8 Hz), 137.3, 134.2 (d, J = 10.5 Hz), 132.8 (d, J = 11.5 Hz), 131.4 (d, J = 4.5 Hz), 130.4 (d, J = 2.0 Hz), 128.3, 125.8 (d, J = 2.7 Hz), 83.7 (d, J = 5.8 Hz), 83.0 (d, J = 4.7 Hz), 38.5 (d, J = 8.7 Hz), 30.5, 27.8. NOTE: residues of free cod ligand are present.

^{31}P NMR (202 MHz, *d*$_8$-toluene) δ 36.21.

Synthesis of [(BISBI)Ni(4-octyne)] (5)

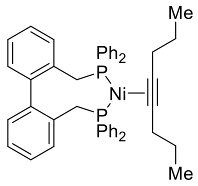

NMR-data: In a glovebox under an argon atmosphere, $Ni(cod)_2$ (6.9 mg, 0.025 mmol, 1.0 equiv) and BISBI (14 mg, 0.025 mmol, 1.0 equiv) were dissolved in *d*$_8$-toluene (0.5 mL) in an NMR tube. Then 4-octyne (3.7 μL, 0.025 mmol, 1.0 equiv) was added. The tube was sealed with a rubber septum. The NMR tube was removed from the glovebox and sonicated for 2 minutes to ensure complete dissolution. Then the NMR spectra were recorded. X-ray quality crystals were obtained by layering the saturated toluene solution with hexane and slow diffusion and evaporation at room temperature over several days in the glovebox under an argon atmosphere.

Large-scale reaction: In an oven-dried 50 mL Schlenk tube equipped with a magnetic stirring bar, $Ni(cod)_2$ (100 mg, 0.364 mmol, 1.00 equiv) and BISBI (200 mg, 0.364 mmol, 1.00 equiv) were dissolved in toluene (10 mL). Then 4-octyne (1.10 mL, 0.750 mmol, 2.06 equiv) were added. The flask was sealed and removed from the glovebox. The mixture was stirred at room temperature for 30 minutes. The reaction mixture was filtered into another 50 mL Schlenk tube using a filter cannula. The toluene was evaporated until approx. 1 mL of toluene was left. Then dried and deoxygenated hexane (10 mL) was added, resulting in the formation of a precipitate. The crude mixture was vigorously stirred for 10 minutes. Stirring was stopped and the residual solvent was filtered into another flask using a filter cannula. Hexane was added again to the filtrate (2 x 10 mL) and was again filtered into the separate flask. All hexane washes were combined and concentrated under reduced pressure using Schlenk technique. The residual beige solid was dried under high vacuum and transferred into the glovebox to yield the product as a light-yellow solid (134 mg, 51%).

^{1}H NMR (500 MHz, *d*$_8$-toluene) δ 8.03 (s, 4H), 7.50 (s, 4H), 7.13 – 7.07 (m, 5H), 7.06 – 7.00 (m, 2H), 7.00 – 6.92 (m, 9H), 6.89 – 6.81 (m, 2H), 6.50 (d, J = 7.7 Hz, 2H), 3.72 – 3.63 (m, 2H), 3.55 (d, J = 13.1 Hz, 2H), 2.08 – 1.92 (m, 4H), 1.31 – 1.20 (m, 2H), 1.21 – 1.10 (m, 2H), 0.62 (t, J = 7.3 Hz, 6H).

^{13}C NMR (126 MHz, *d*$_8$-toluene) δ 142.6 (t, J = 1.8 Hz), 140.9 – 140.0 (m), 136.9, 136.6 (dd, J = 16.9, 14.6 Hz), 136.1 (t, J = 7.6 Hz), 131.7 (t, J = 6.5 Hz), 130.2, 129.9 (d, J = 12.6 Hz), 127.12, 125.8 (d, J = 1.5 Hz), 122.01 – 121.3 (m), 40.1 (t, J = 5.1 Hz), 29.6 (t, J = 7.7 Hz), 24.3, 14.3.

^{31}P NMR (202 MHz, *d*$_8$-toluene) δ 37.97.

11. NMR studies

a) Formation of coordination complexes at room temperature

Formation of [(BISBI)Ni(donor)] complex 6:

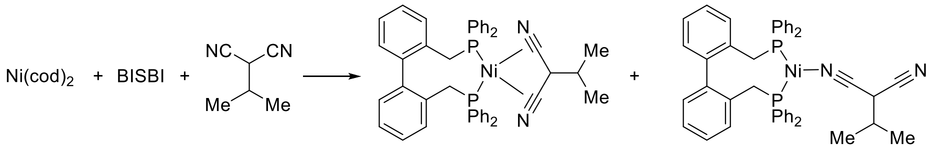

In a glovebox under an argon atmosphere, Ni(cod)$_2$ (6.9 mg, 0.025 mmol, 1.0 equiv) and BISBI (14 mg, 0.025 mmol, 1.0 equiv) were dissolved in d_8-toluene (0.5 mL) in an NMR tube. Then 2-isopropylmalononitrile **2a** (5.4 mg, 0.050 mmol, 2.0 equiv) was added. The tube was sealed with a rubber septum. The NMR tube was removed from the glovebox and sonicated for 2 minutes to ensure complete solvation. Then the NMR spectra were recorded.

Formation of [(BISBI)Ni(donor)] complex 6:

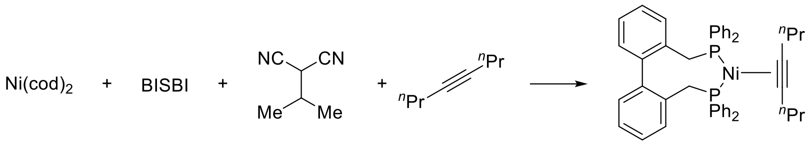

In a glovebox under an argon atmosphere, Ni(cod)$_2$ (6.9 mg, 0.025 mmol, 1.0 equiv) and BISBI (14 mg, 0.025 mmol, 1.0 equiv) were dissolved in d_8-toluene (0.5 mL) in an NMR tube. Then 2-isopropylmalononitrile **2a** (5.4 mg, 0.050 mmol, 2.0 equiv) and 4-octyne **1a** (3.75 µL, 0.025 mmol, 1.0 equiv) were added. The tube was sealed with a rubber septum. The NMR tube was removed from the glovebox and sonicated for 2 minutes to ensure complete solvation. Then the NMR spectra were recorded.

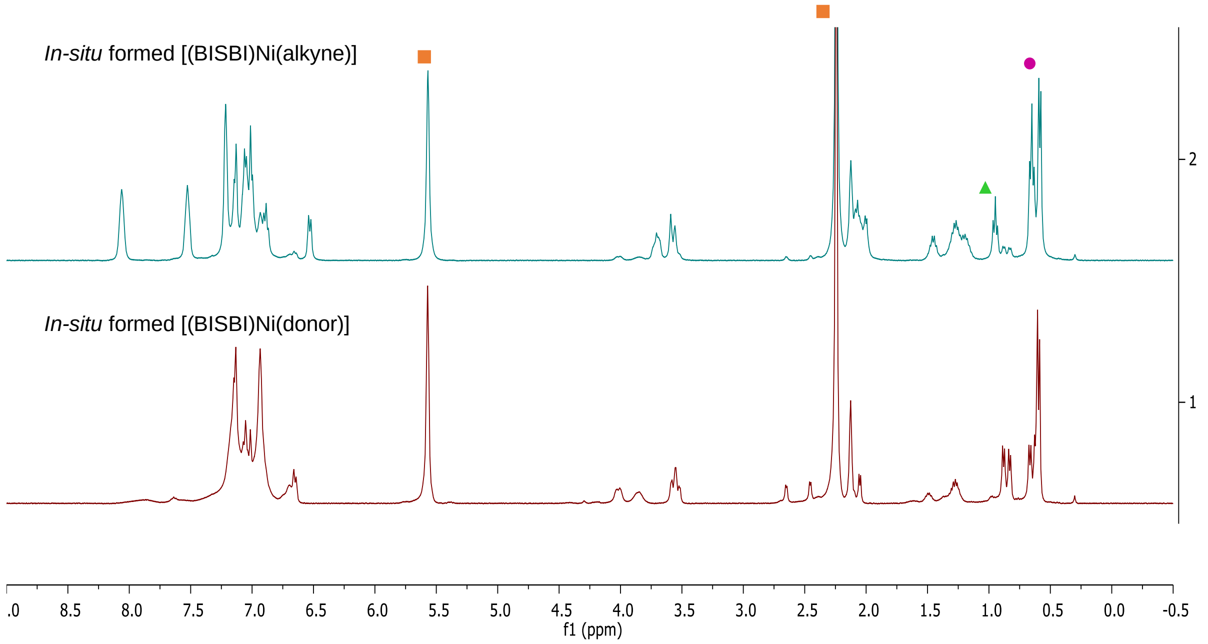

Figure S22 Comparison of ^{1}H NMR of *in-situ* formed [(BISBI)Ni(donor)] complex and [(BSBI)Ni(alkyne)] complex. Uncoordinated COD (■), uncoordinated alkyne (▲) and uncoordinated donor (●) are depicted.

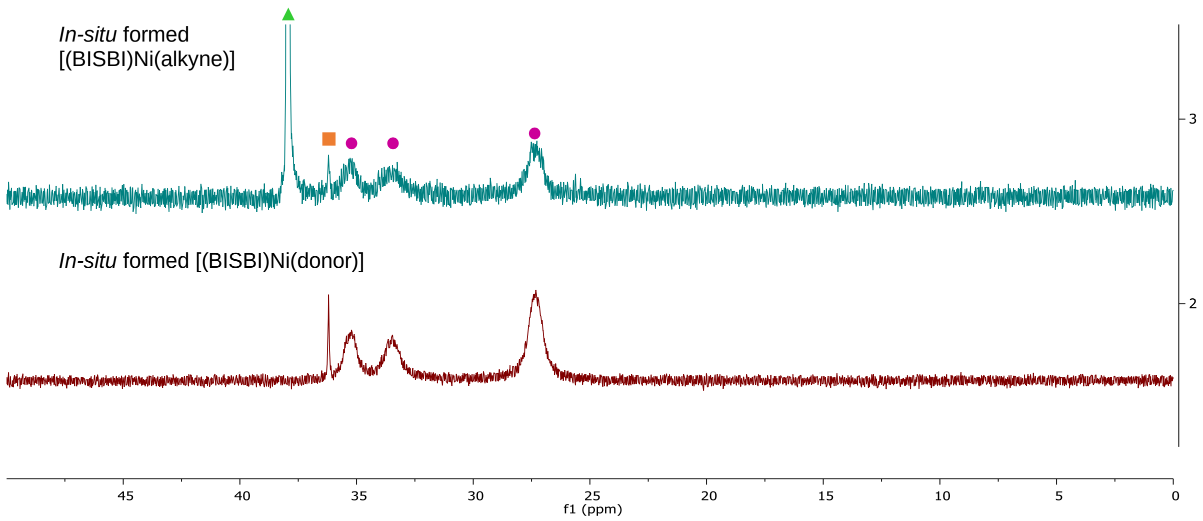

Figure S23 Comparison of $^{31}P\{^{1}H\}$ NMR of *in-situ* formed [(BISBI)Ni(donor)] complex and [(BSBI)Ni(alkyne)] complex. [(BISBI)Ni(COD)] (■), [(BISBI)Ni(alkyne)] (▲) and [(BISBI)Ni(donor)] (●) are depicted.

When mixing the nickel pre-catalyst with the diphosphine as well as the HCN donor, the formation of four distinct peaks in the $^{31}P\{^{1}H\}$ NMR can be detected. The sharp peak refers to the [(BISBI)Ni(COD)] complex while the three broader peaks were tentatively assigned to a side-on (symmetric) and a potential end-on donor coordination complex (unsymmetric, see above).

b) VT-NMR analysis of standard reaction conditions

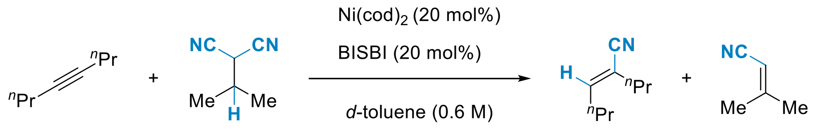

1a **2a** **3a** **4a**

In a glovebox under an argon atmosphere, Ni(cod)$_2$ (15 mg, 0.055 mmol, 20 mol%) and BISBI (30 mg, 0.055 mmol, 20 mol%) were dissolved in d_8-toluene (0.50 mL) in an NMR tube. Then 2-isopropylmalononitrile **2a** (30 mg, 0.28 mmol, 1.0 equiv) and 4-octyne **1a** (61 μL, 0.42 mmol, 1.5 equiv) were added. The tube was sealed with a rubber septum and parafilm. The NMR tube was removed from the glovebox and sonicated for 5 minutes to ensure complete dissolution. Then the NMR spectra were recorded at the given temperatures.

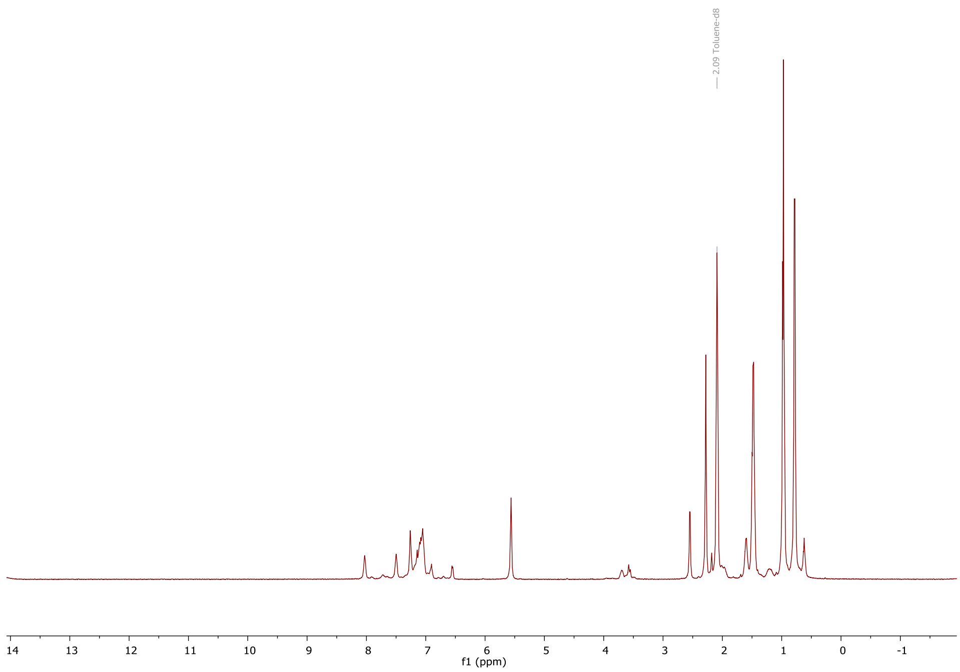

Figure S24 ^{1}H-NMR (500 MHz, d_8-toluene) at t = 0 min, 80 °C

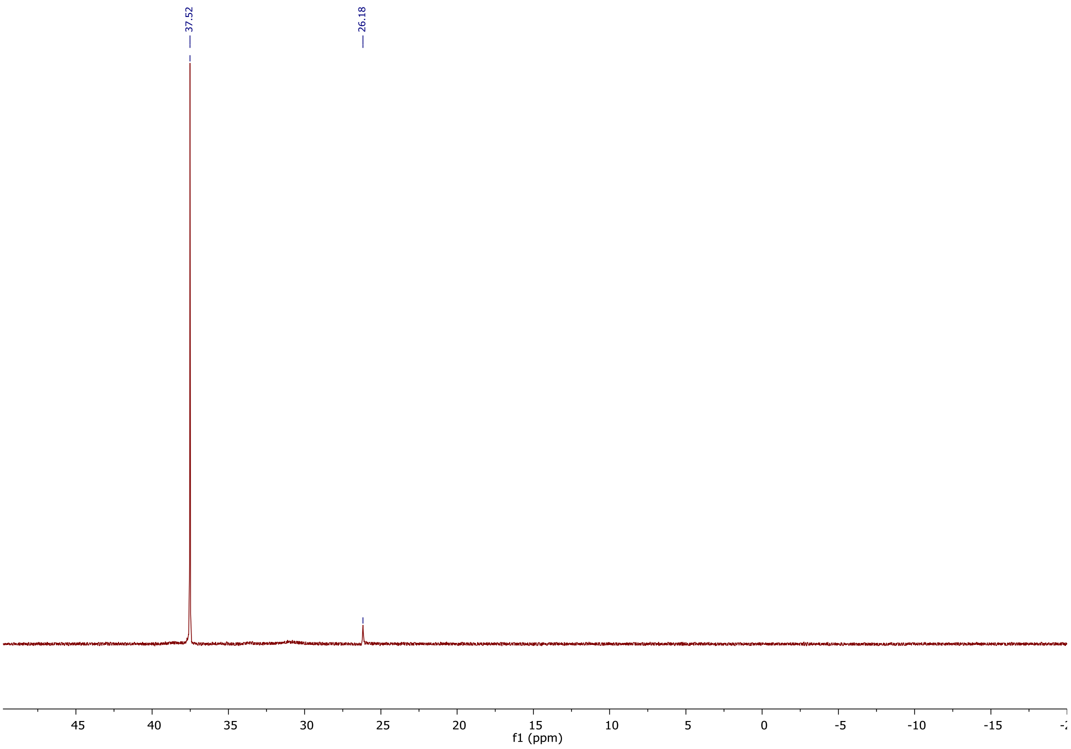

Figure S25 $^{31}P\{^{1}H\}$ NMR (202 MHz, d_8-toluene) at t = 0 min, 80 °C. Signal at δ 37.52 ppm corresponds to [(BISBI)Ni(alkyne)] complex.

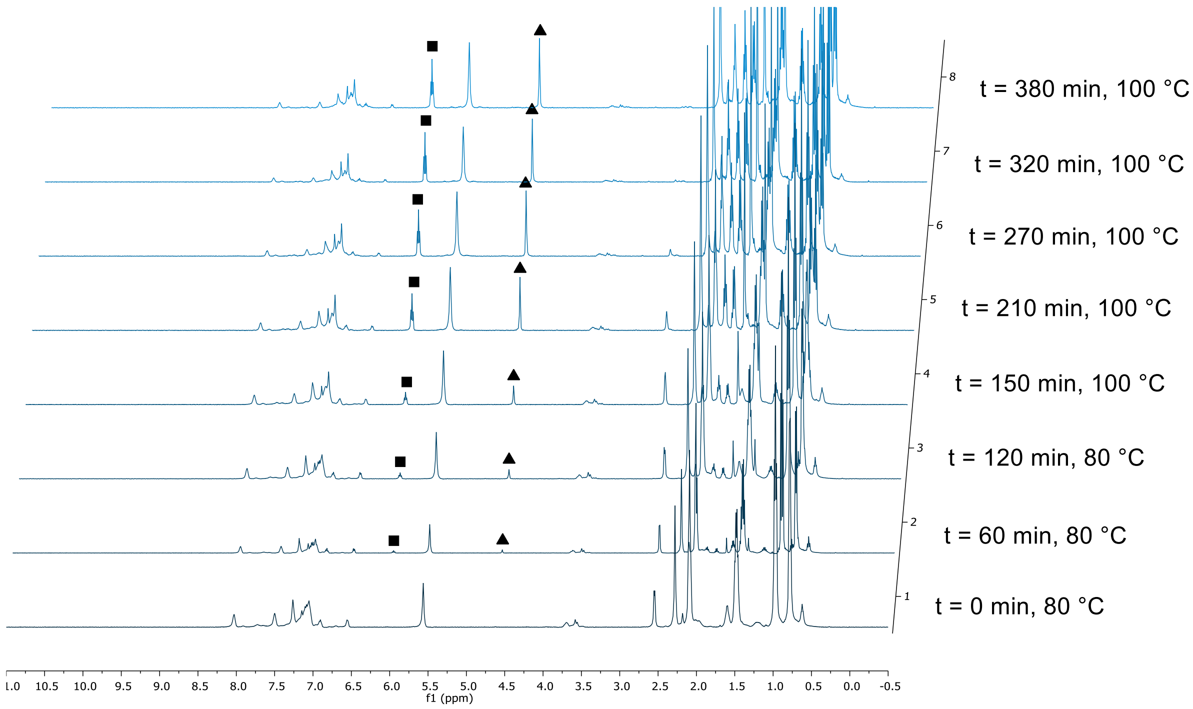

Figure S26 Comparison of ^{1}H-NMR (500 MHz, d_8-toluene) over time at indicated temperatures. The major species corresponds to the signal at δ 37.52 ppm in the $^{31}P\{^{1}H\}$ spectrum, indicating the presence of the [(BISBI)Ni(alkyne)] complex as the resting state of the reaction. Formation of hydrocyanated product 3a (■) and side-product 4a (▲) over time.

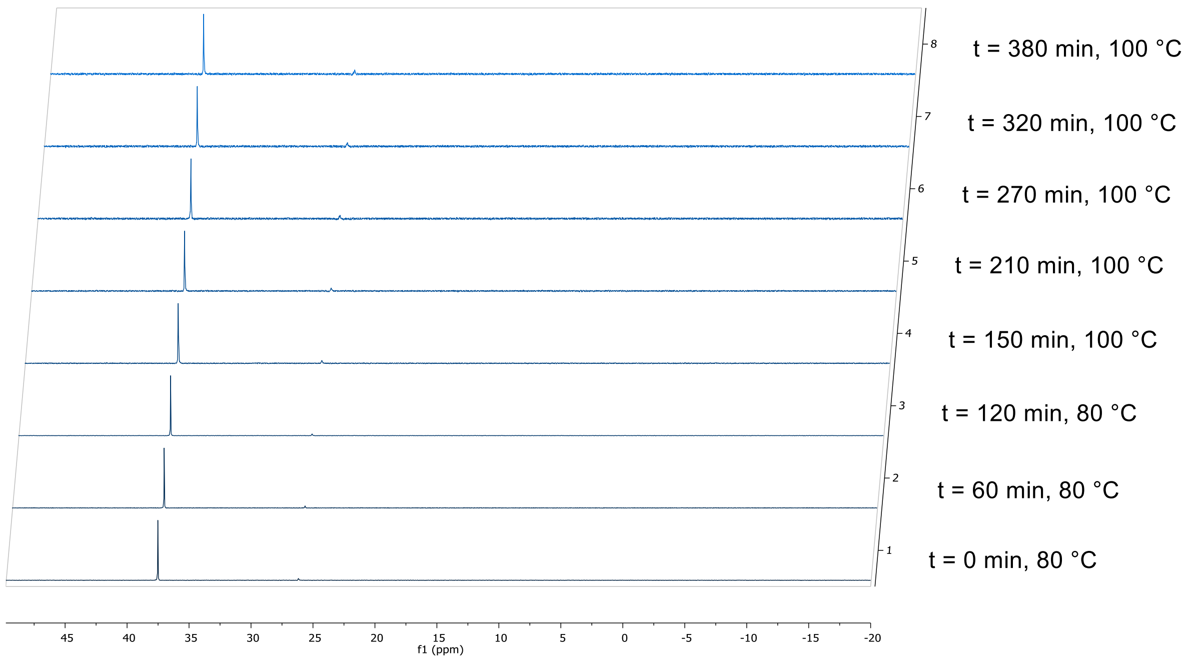

Figure S27 Comparison of $^{31}P\{^{1}H\}$-NMR (202 MHz, d_8-toluene) over time at indicated temperatures. Major species corresponds to the signal at δ 37.52 ppm indicating the presence of the [(BISBI)Ni(alkyne)] complex as the resting state of the reaction.

c) Attempts to identify oxidative addition complexes

In initial experimental attempts, the identity of the proposed oxidative addition complexes [(BISBI)Ni(CN)(alkyl)] was examined. Stoichiometric experiments were run to either detect the formation of these complexes *in-situ* by ^{1}H or $^{31}P\{^{1}H\}$ NMR analysis or by crystallization to identify any of these complexes via single-crystal X-ray analysis.

***Stoichiometric experiments* – [(BIBSI)Ni(cod)] and 2-isopropylmalononitrile 2a**

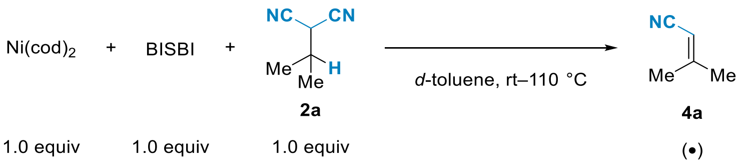

In a glovebox under an argon atmosphere, $Ni(cod)_2$ (6.9 mg, 0.025 mmol, 1.0 equiv), BISBI (14 mg, 0.025 mmol, 1.0 equiv), and 2-isopropylmalononitrile **2a** (4.0 mg, 0.025 mmol, 1.0 equiv) were dissolved in d_8-toluene (0.50 mL) in an NMR tube. The NMR tube was sealed, removed from the glovebox, and sonicated for 2 minutes. ^{1}H- and $^{31}P\{^{1}H\}$-NMR spectra were recorded at the start, after heating the sample for 1 hour at 60 °C, for 1 hour at 80 °C, for 1 hour at 90 °C and subsequently for 10.5 hours at 110 °C.

The identity of any additional signals hinting towards the formation of oxidative addition complexes could not be detected in the $^{31}P\{^{1}H\}$ spectra, while the formation of byproduct **4a** was confirmed by ^{1}H NMR analysis.

Isolation of oligomeric metal cluster

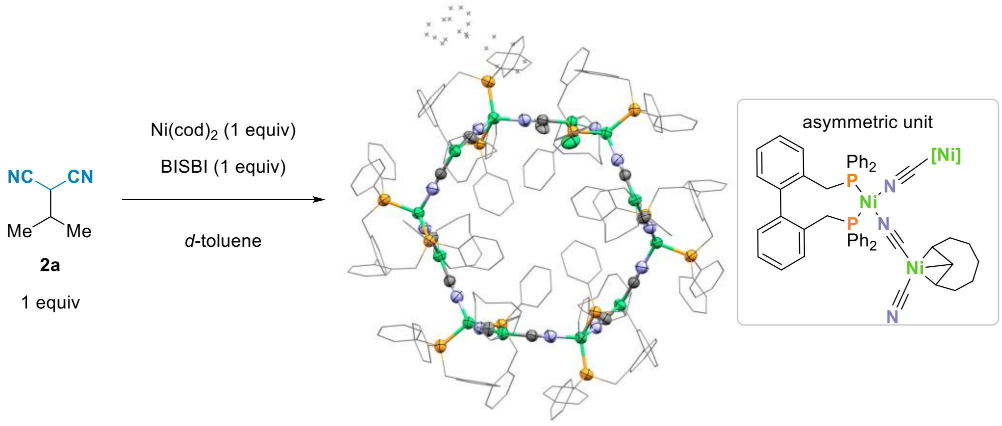

In a glovebox under an argon atmosphere, Ni(cod)$_2$ (6.9 mg, 0.025 mmol, 1.0 equiv), BISBI (14 mg, 0.025 mmol, 1.0 equiv), 2-isopropylmalononitrile **2a** (2.7 mg, 0.025 mmol, 1.0 equiv) were dissolved in d_8-toluene (0.50 mL) in an NMR tube. The NMR tube was sealed, removed from the glovebox, and sonicated for 2 minutes. Then the NMR tube was placed in an oil bath and heated at 80 °C for several hours. After cooling down to room temperature, the NMR tube was kept at room temperature under an inert atmosphere for several days until the formation of crystals was observed, which were subsequently submitted to X-ray analysis.

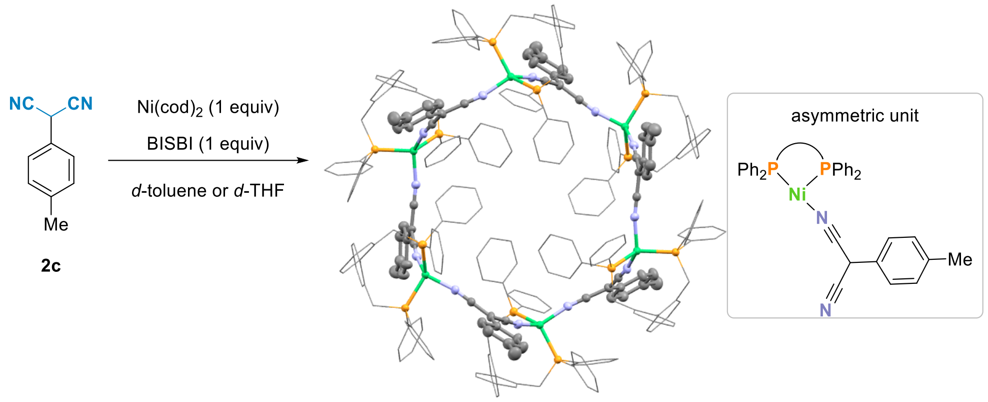

In a glovebox under an argon atmosphere, Ni(cod)$_2$ (6.9 mg, 0.025 mmol), BISBI (14 mg, 0.025 mmol) or dppf (14 mg, 0.025 mmol, 1.0 equiv), 2-(*p*-tolyl)malononitrile **2c** (3.9 mg, 0.025 mmol, 1.0 equiv) or 2-(*tert*-butyl)malononitrile **2d** (3.1 mg, 0.025 mmol, 1.0 equiv) were dissolved in d_8-THF (0.50 mL) in an NMR tube. The NMR tube was sealed, removed from the glovebox, and sonicated for 2 minutes. Then the NMR tube was placed in an oil bath and heated at the indicated temperature. After cooling down to room temperature, the NMR tube was kept at room temperature for several days under an inert atmosphere until the formation of crystals was observed, which were subsequently submitted to X-ray analysis.

Isomerization of cod ligand induced by the addition of donor substrates

In a glovebox under an argon atmosphere, Ni(cod)$_2$ (6.9 mg, 0.025 mmol, 1.0 equiv), BISBI (14 mg, 0.025 mmol, 1.0 equiv), 2-isopropylmalononitrile **2a** (2.7 mg, 0.025 mmol, 1.0 equiv), or 2-(*p*-tolyl)malononitrile **2c** (3.9 mg, 0.025 mmol, 1.0 equiv), or 2-(*tert*-butyl)malononitrile **2d** (3.1 mg, 0.025 mmol, 1.0 equiv) were dissolved in d_8-toluene (0.50 mL) in an NMR tube. The NMR tube was sealed, removed from the glovebox, and sonicated for 2 minutes. Then the NMR tube was placed in an oil bath and heated at the indicated temperature. After cooling down to room temperature, a ^{1}H-NMR spectrum was measured.

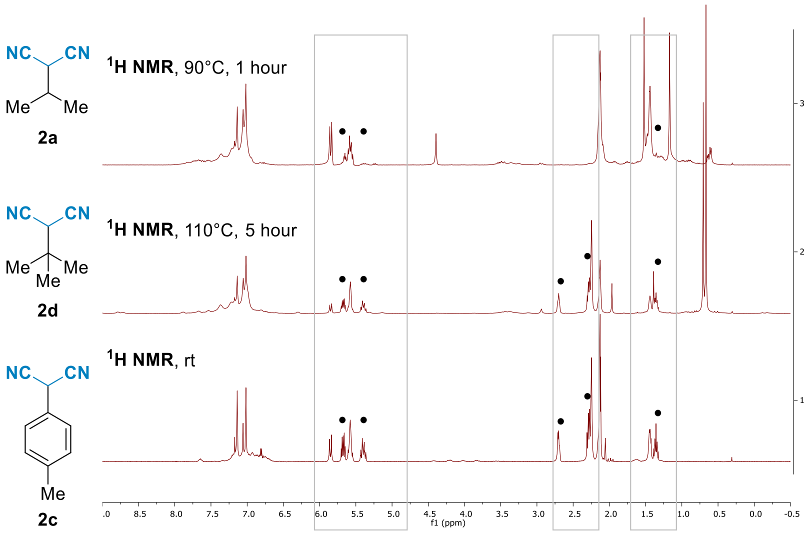

Figure S28 ^{1}H-NMR spectrum of the crude reaction mixture indicates isomerization of the cod ligand via proposed nickel–hydride intermediates in the presence of HCN donor reagents. ● refers to isomerized cod ligand.

d) Probing SET pathways

Initially, single electron transfer (SET) pathways for the activation of the C(sp^3)–CN bond by the nickel catalyst were considered. The presence of any radical intermediates was probed by using radical scavengers, such as TEMPO and BHT. As no trapped intermideates were detected, the experiments remained inconclusive.

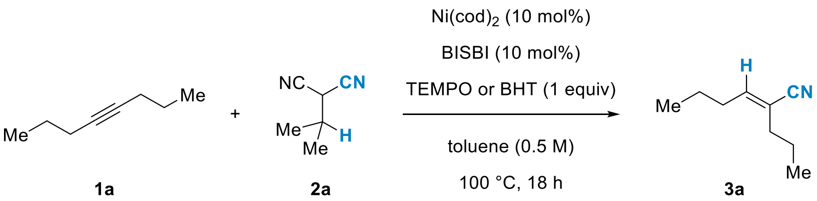

To an oven-dried 4 mL screw-cap vial, 2-isopropylmalononitrile **2a** (65 mg, 0.60 mmol, 1.2 equiv), d_8-toluene (0.20 mL), 4-octyne **1a** (73 µL, 0.50 mmol), and the indicated additive (0.50 mmol, 1.0 equiv) were added under an argon atmosphere in a glovebox. In another oven-dried vial, BISBI (28 mg, 0.050 mmol, 10 mol%), and Ni(cod)$_2$ (14 mg, 0.050 mmol, 10 mol%) were dissolved in d_8-toluene (1.30 mL) and the mixture was stirred until complete dissolution (solution turned dark red). The precatalyst solution was then added to the starting material in one portion. The vial was sealed and removed from the glovebox then heated at 100 °C for 18 hours. After cooling to room temperature, 1,1,2,2,-tetrachloroethane (53 µL) was added as an internal standard, and the mixture was filtered over a plug of silica into an NMR tube. The tube was sealed and subjected to NMR analysis of the crude reaction mixture.

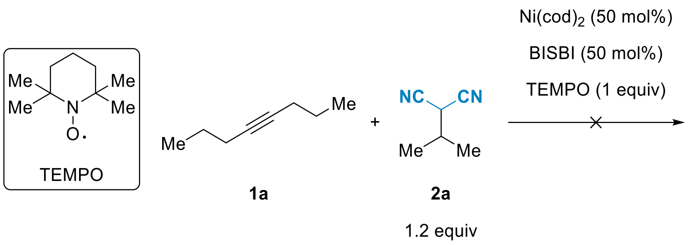

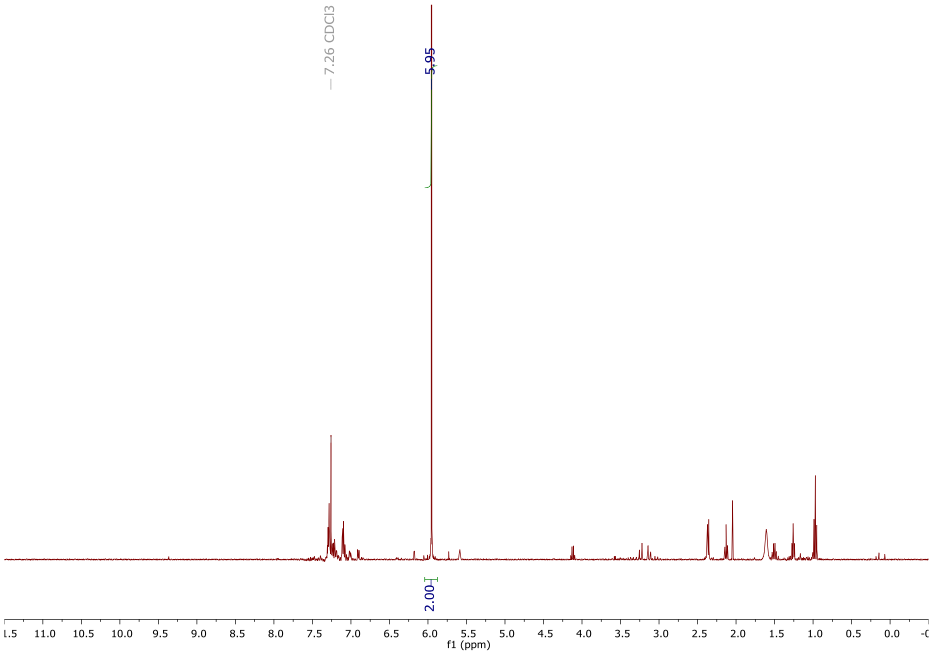

Figure S29 ^{1}H NMR of the crude reaction mixture after adding 1 equiv of TEMPO to the reaction (400 MHz, $CDCl_3$). The peak at 5.95 ppm corresponds to the internal standard.

When TEMPO was used as a radical scavenger in the transfer hydrocyanation of alkynes, the formation of the desired transfer hydrocyanation product could not be observed.

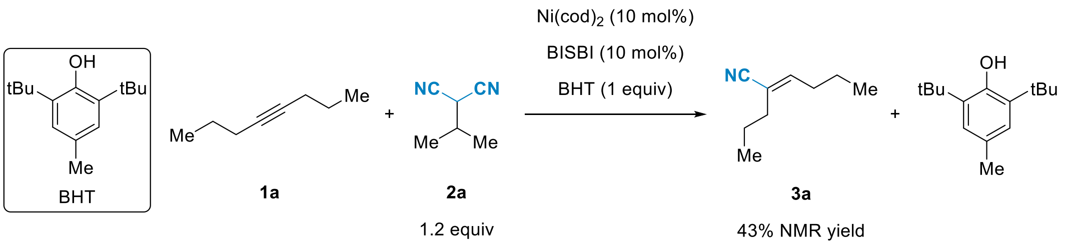

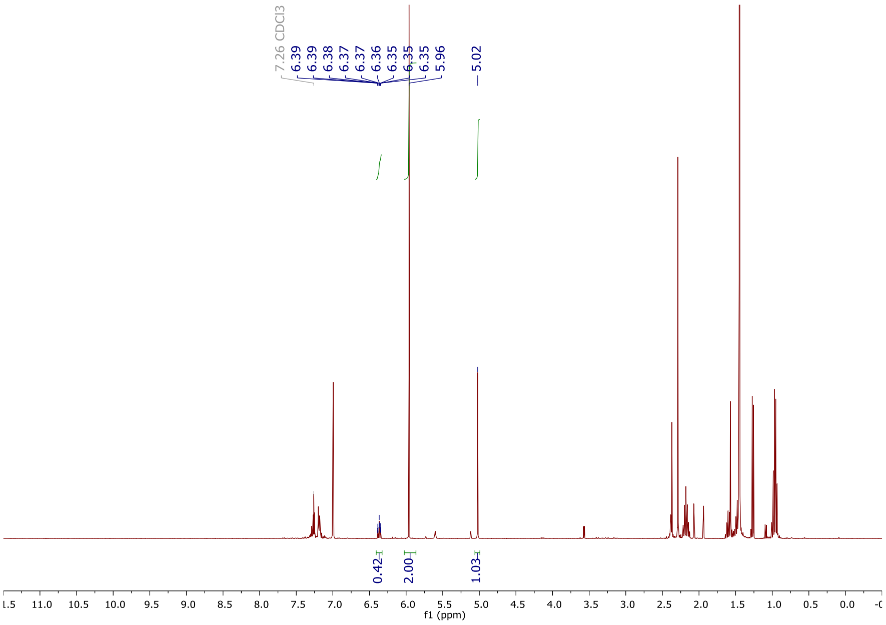

Figure S30 ^{1}H-NMR of the crude reaction mixture after using 1 equiv of BHT in the reaction (400 MHz, $CDCl_3$). The peak at 5.96 ppm corresponds to the internal standard.

When BHT was used as a radical scavenger in the transfer hydrocyanation of alkynes, the formation of the desired transfer hydrocyanation product could be observed. Based on the internal standard, 42% of hydrocyanation product **3a** along with 1 equiv of the dehydrocyanation byproduct 4a were obtained.

Based on these experiments, the formation of any radical intermediates during the transfer hydrocyanation reaction could not be identified.

12. Computational details

a) General information

Unless stated otherwise, all proposed intermediates were based on X-ray structures or modelled in ChemCraft[18]. For the structures in the main text conformer searches were run with CREST version 2.11[19,20] and xTB version 6.4.0.[21,22] All subsequent calculations were run with ORCA 5.0.3.[23] Geometry optimizations and frequency calculations were run with the PBE0 functional,[24] the def2-TZVP[25] basis set for Ni, and the def2-SVP[25] basis set for all other atoms. Grimme's atom-pairwise dispersion correction with the Becke-Johnson damping scheme was used,[26,27] as was the RIJCOSX algorithm,[28] and the def2/J auxiliary basis set[29] was chosen. The cpcm solvent model was used for toluene. The temperature for the frequency calculations was set to 373.15 K. Transition states were located from relaxed potential energy surface scans and were confirmed by the number of imaginary frequencies (N_{if}=1) and by either an IRC calculation or by manual displacement of the imaginary frequency. Structures **8** and **TS6** have one additional small imaginary frequency, but due to the magnitude, ~3i in both cases, this should not greatly influence the energy of the system.

Input example for geometry optimization and frequency calculations (and transition state optimizations, where the tightopt keyword was replaced with opts):

```
! PBE0 def2-SVP D3BJ RIJCOSX def2/J DefGrid2 tightscf tightopt freq cpcm(toluene)
%basis
newgto Ni "def2-tzvp" end
end
%Freq temp 373.15 END
*xyzfile 0 1 <coordinate file>
```

Single point energies were calculated with the def2-QZVP basis set[25] for Ni and the def2-TZVP basis set for all other atoms. Again, Grimme's atom-pairwise dispersion correction with the Becke-Johnson damping scheme was used, as was the RIJCOSX algorithm, and the def2/J auxiliary basis set was chosen. The cpcm solvent model was used with toluene.

Input example for single point calculations:

```
! PBE0 def2-TZVP D3BJ RIJCOSX def2/J DefGrid2 tightscf cpcm(toluene)
%basis
newgto Ni "def2-qzvp" end
end
*xyzfile 0 1 <coordinate file>
```

The Gibbs free energy of the reaction was calculated by adding the electronic energy from the single point calculation to the thermochemical correction from the frequency calculation.
The depicted structures in the main text and the SI were visualized with CYLView20.[30] The cartesian coordinates of all optimized structures are given in a separate text file.

b) Activation of 3-methylbutanenitrile donor

To assess the stabilizing effect of the second nitrile group, we computed the reaction energy profile for the activation of the donor with only one nitrile. The proposed intermediates were based on X-ray structures or modelled in ChemCraft, starting from the structures obtained in the main computational study. Geometry optimizations, frequency calculations, and single point calculations were run with the same level of theory as the calculations in the main computational study.

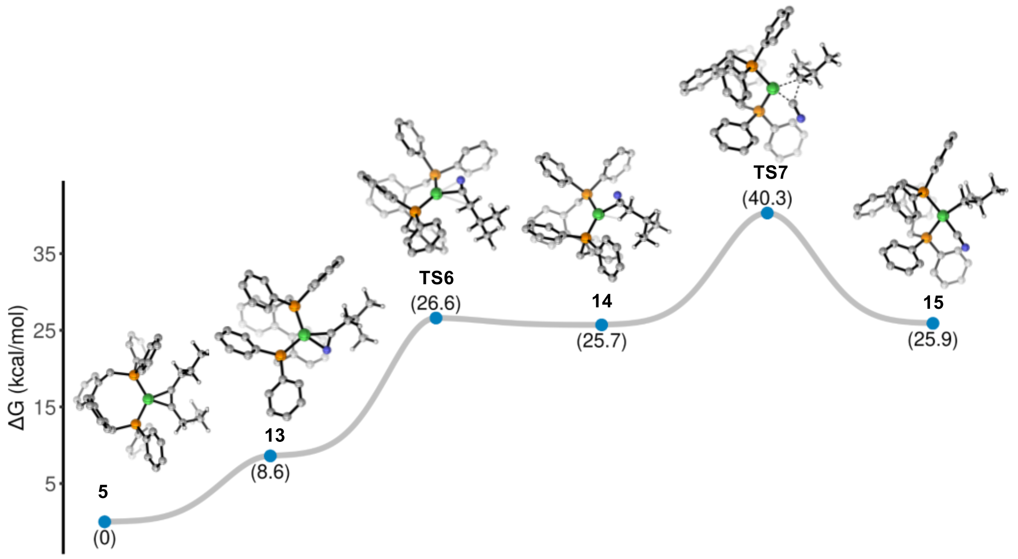

Figure S31 Calculated reaction pathway for the activation of the 3-methylbutanenitrile donor.

c) Kinetic isotope effect

The kinetic isotope effects were calculated using *PyQuiver*,[31] an open-source Python program using harmonic frequencies and the Bigeleisen-Mayer equation and tunnelling corrections. For this, the hessian file (generated by the frequency calculation) of the resting state (**5** + **2a**) and the transition state (**TS2**) are required, and all carbons of interest have to be declared with their atom numbering. C_CN1 is the carbon of the "coordinating" nitrile, C_CN2 is the carbon of the "reacting" nitrile, and we chose C_gamma1 as the reference for the calculations.

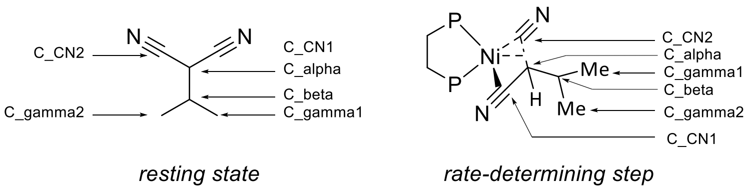

Configuration file for the KIE determination:

```
scaling 0.970
imag_threshold 50
temperature 373
mass_override_isotopologue default
reference_isotopomer C_gamma1
isotopomer C_alpha 4 77 13C
isotopomer C_beta 2 75 13C
isotopomer C_CN1 7 80 13C
isotopomer C_CN2 5 78 13C
isotopomer C_gamma1 3 76 13C
isotopomer C_gamma2 1 74 13C
```

The result of this is:

		Wigner KIE
Isotopologue	C_alpha	1.0276
Isotopologue	C_beta	0.9985
Isotopologue	C_CN1	1.0119
Isotopologue	C_CN2	1.0451
Isotopologue	C_gamma2	0.9987

We used the Wigner KIE for our study (for clarity, the non-corrected and the inverted parabola KIEs that are typically also printed are omitted from the table above), and all the listed KIEs are referenced to “isotopologue C_gamma1”, whose absolute KIE is 1.0011.

As there are two nitriles in the donor which cannot be distinguished spectroscopically, the apparent KIE that is derived experimentally cannot be directly compared to the calculated KIE. To enable such a comparison between the apparent (experimental) KIE and the individual calculated KIEs for the two nitriles, a number of assumptions were made:

- We do not account for doubly labeled ^{13}C-donor as the natural abundance for this case is very low, the results would not differ significantly, and we thus assume that the error introduced by this is negligible.
- As the barrier for the subsequent β-H elimination is significantly lower (by more than 5 kcal/mol) than the reverse reaction (reductive elimination to reform the malononitrile) and the overall transformation is strongly exothermic, we assume that there is a negligible reversible character of C–CN bond activation step.

The rates for the different isotopologues of the donor are defined below.

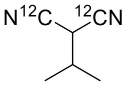

The rate of the light isotopologue is simply k_{12}.

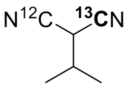

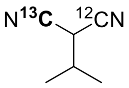

The rate of the heavy isotopologue is the sum of the rate when the ^{13}CN is the reacting nitrile (C_{CN2}) and the rate when the ^{13}CN is the coordinated nitrile (C_{CN1}): $0.5 \times \frac{k12}{KIE(C_{CN1})} + 0.5 \times \frac{k12}{KIE(C_{CN2})}$

The apparent KIE is thus:

$$\frac{k(light)}{k(heavy)} = \frac{k12}{0.5 \times \frac{k12}{KIE(C_{CN1})} + 0.5 \times \frac{k12}{KIE(C_{CN2})}} = \frac{k12}{\frac{0.5 \times k12 \times KIE(C_{CN2}) + 0.5 \times k12 \times KIE(C_{CN1})}{KIE(C_{CN1}) \times KIE(C_{CN2})}} =$$

$$\frac{k12 \times KIE(C_{CN1}) \times KIE(C_{CN2})}{0.5 \times k12 \times KIE(C_{CN2}) + 0.5 \times k12 \times KIE(C_{CN1})} = \frac{KIE(C_{CN1}) \times KIE(C_{CN2})}{0.5 \times (KIE(C_{CN2}) + KIE(C_{CN1}))} = \frac{2 \times KIE(C_{CN1}) \times KIE(C_{CN2})}{KIE(C_{CN2}) + KIE(C_{CN1})}$$

Inserting the values obtained from *PyQuiver* gives the apparent KIE for the nitriles:

$$KIE(CN)_{apparent} = \frac{2 \times KIE(C_{CN1}) \times KIE(C_{CN2})}{KIE(C_{CN2}) + KIE(C_{CN1})} = \frac{2 \times 1.0119 \times 1.0451}{1.0119 + 1.0451} = \frac{2.1151}{2.057} = 1.0282$$

13. NMR data

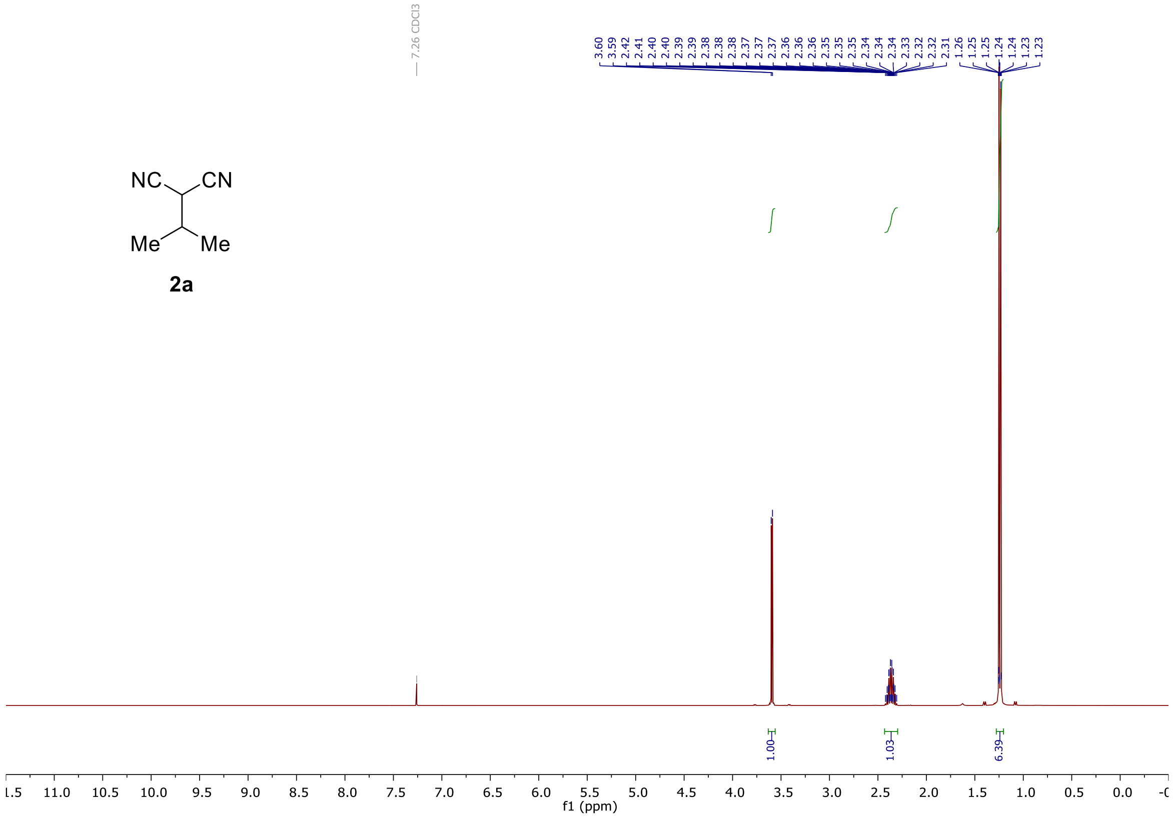

Figure S32 ^{1}H NMR spectrum of **2a** (400 MHz, $CDCl_3$).

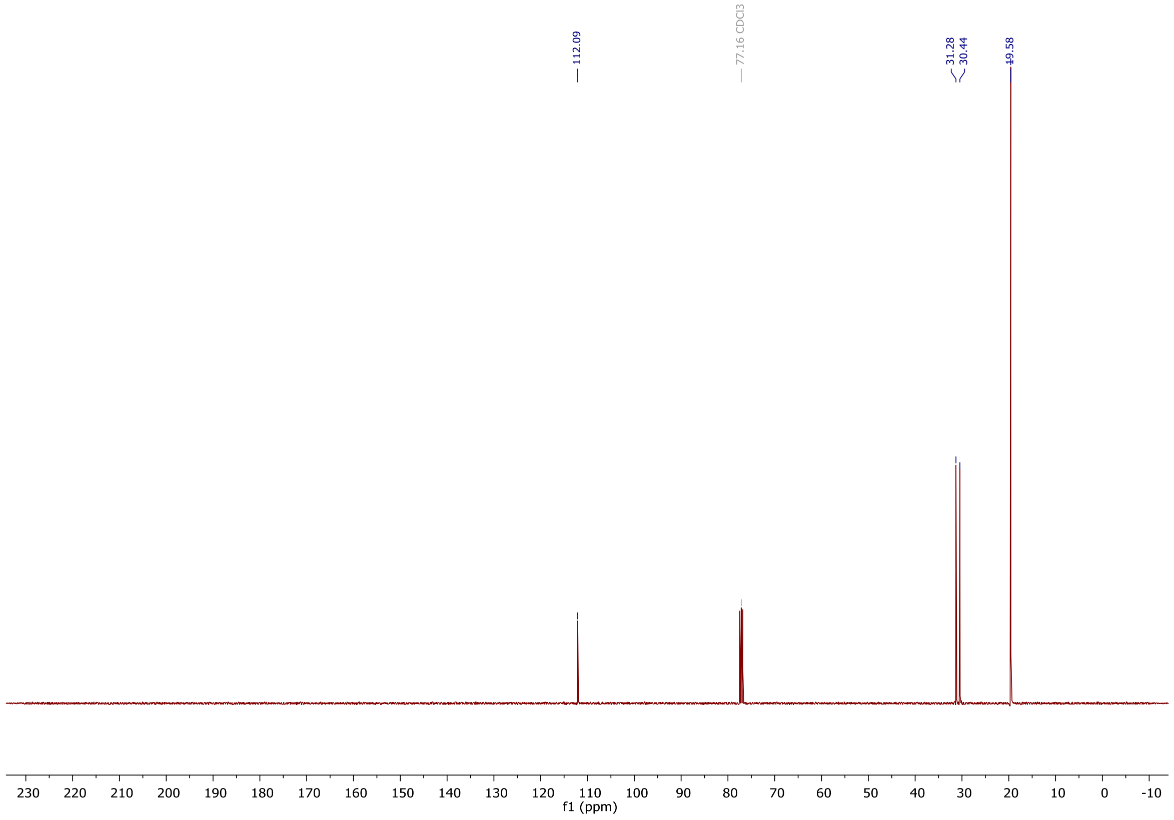

Figure S33 ^{13}C NMR spectrum of **2a** (101 MHz, $CDCl_3$).

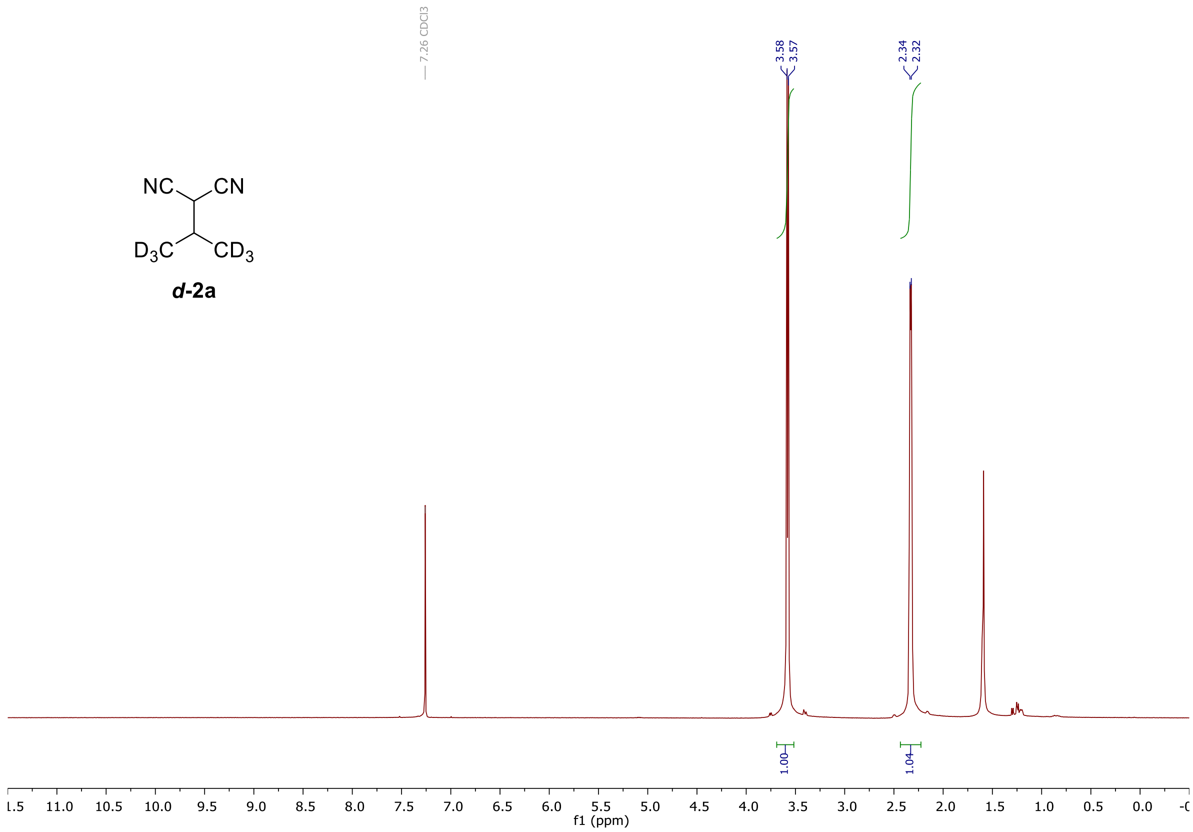

Figure S34 ^{1}H NMR spectrum of ***d*-2a** (400 MHz, $CDCl_3$).

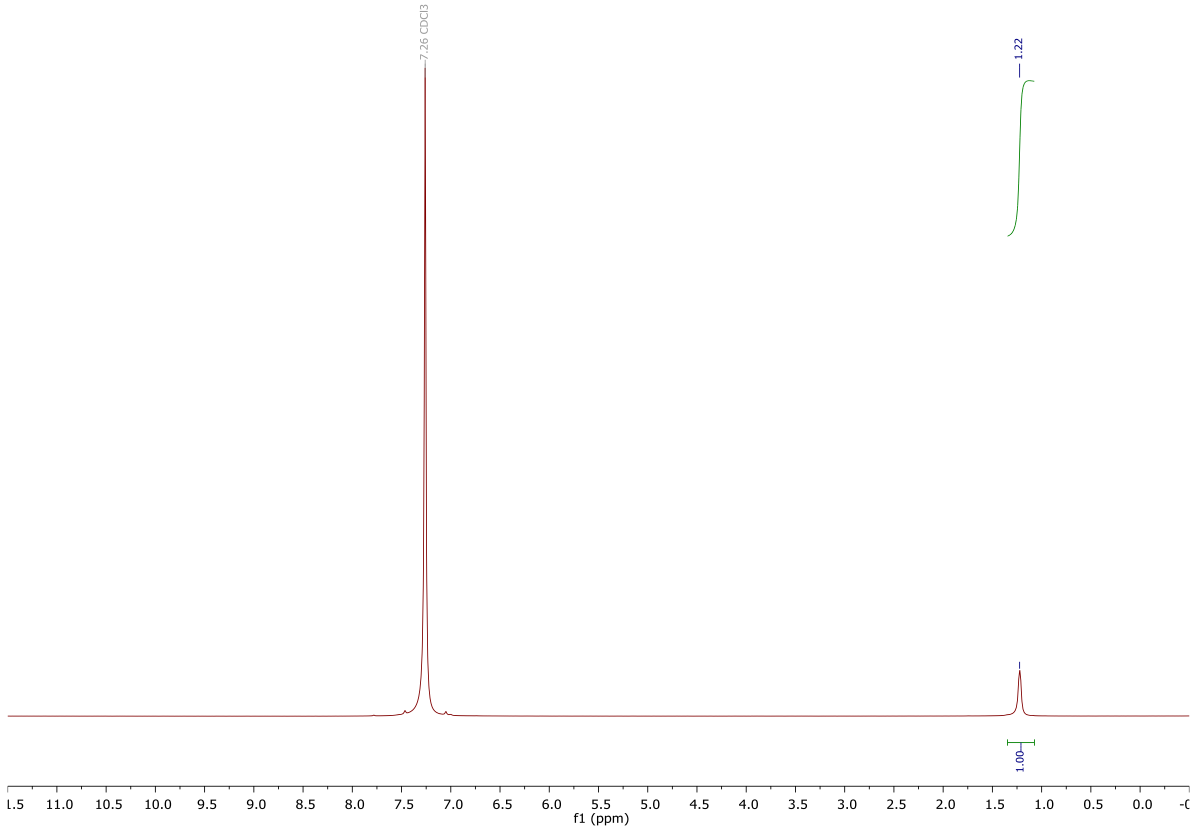

Figure S35 ^{2}H NMR spectrum of ***d*-2a** (77 MHz, $CDCl_3$).

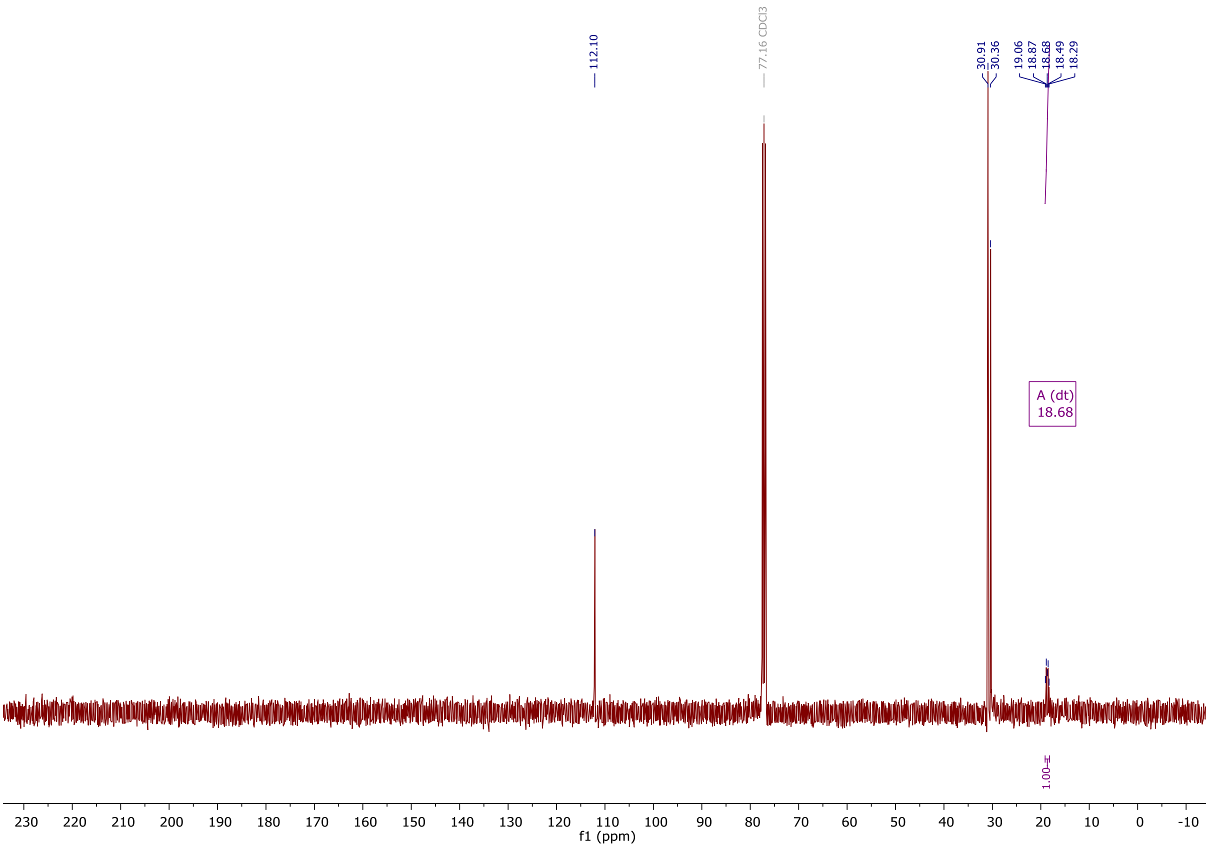

Figure S36 ^{13}C NMR spectrum of ***d*-2a** (101 MHz, $CDCl_3$).

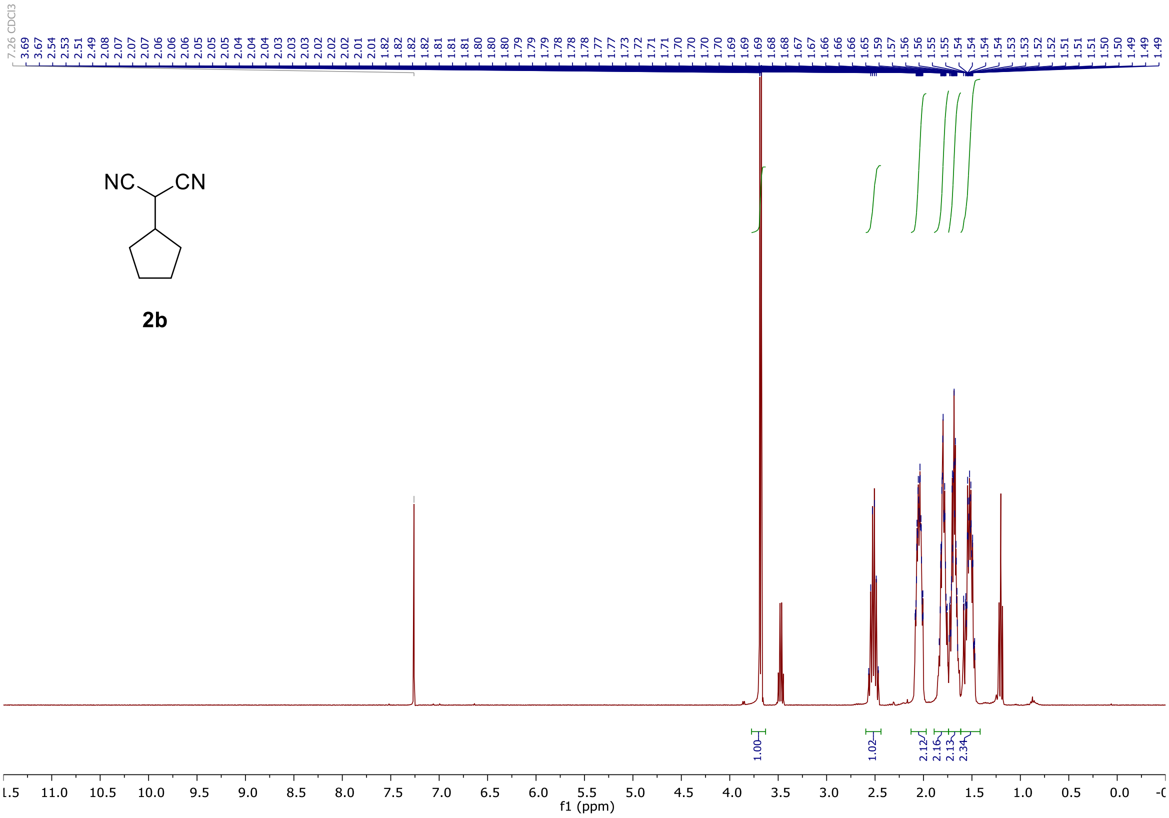

2b

Figure S37 ^{1}H NMR spectrum of **2b** (400 MHz, $CDCl_3$).

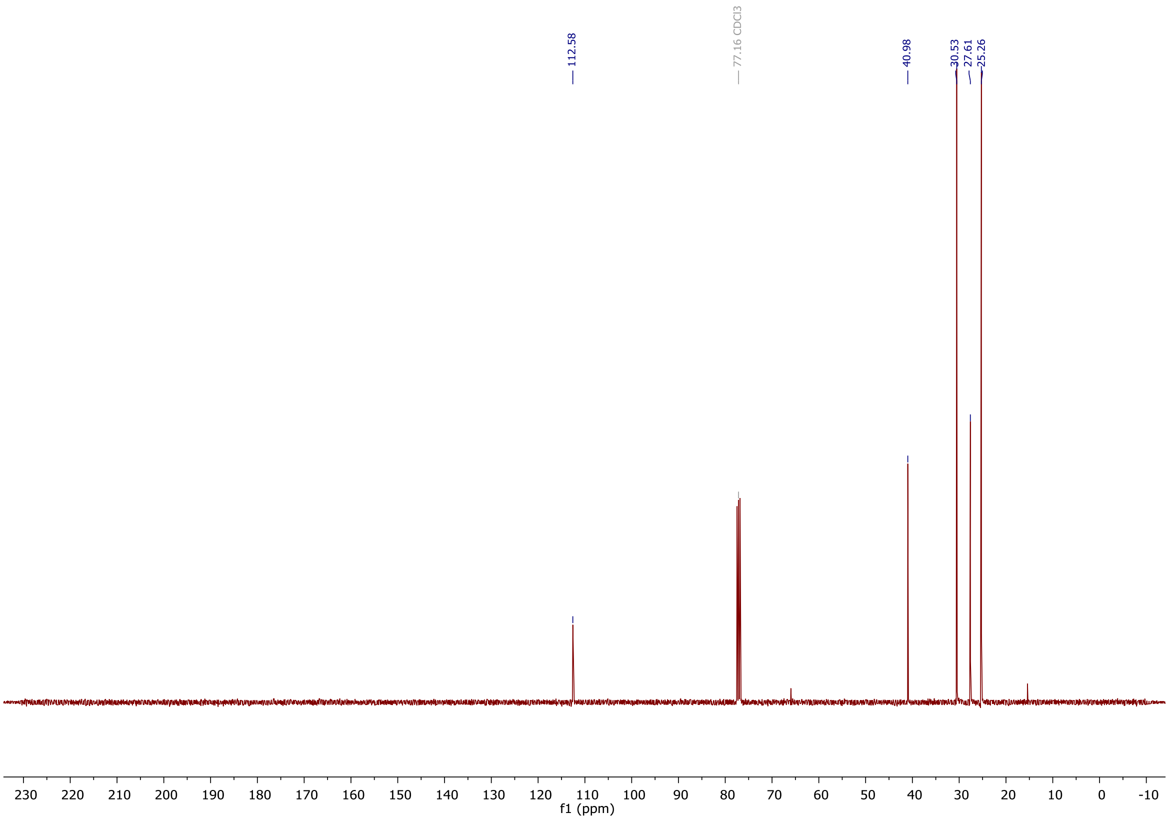

Figure S38 ^{13}C NMR spectrum of **2b** (101 MHz, $CDCl_3$).

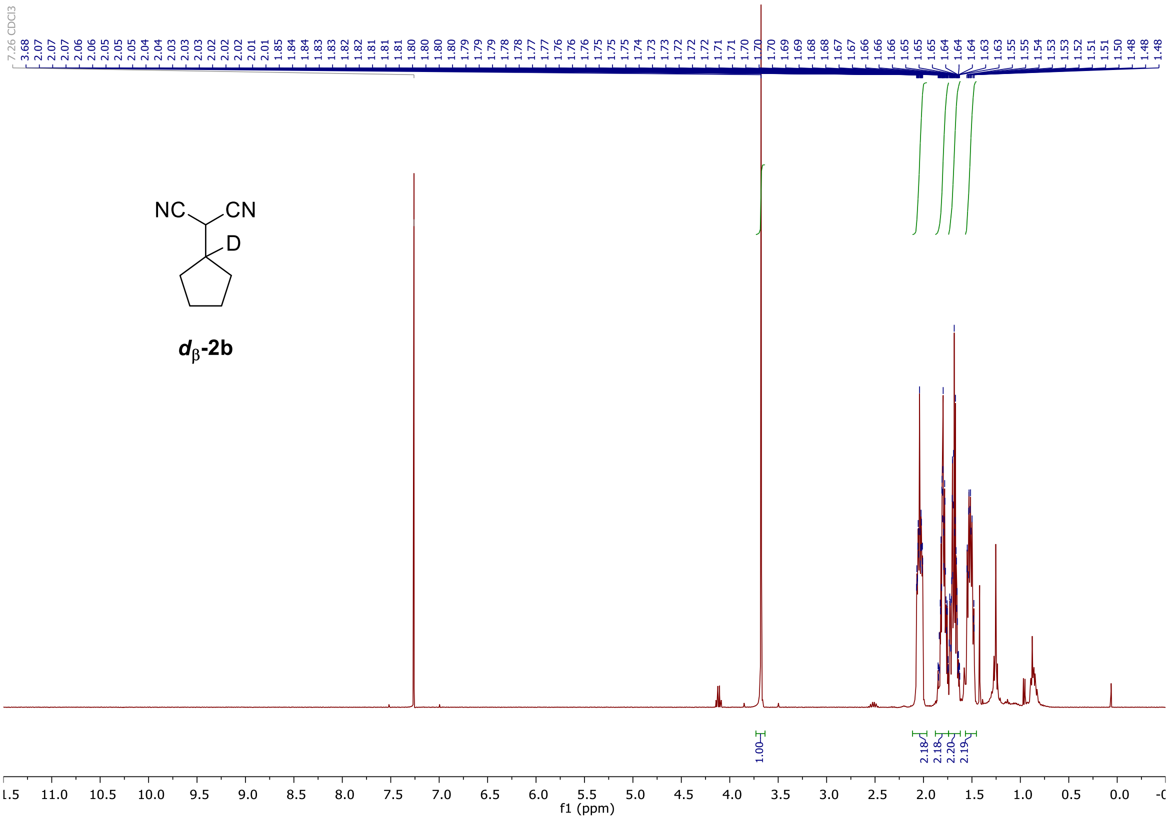

Figure S39 ^{1}H NMR spectrum of ***d*$_\beta$-2b** (400 MHz, $CDCl_3$).

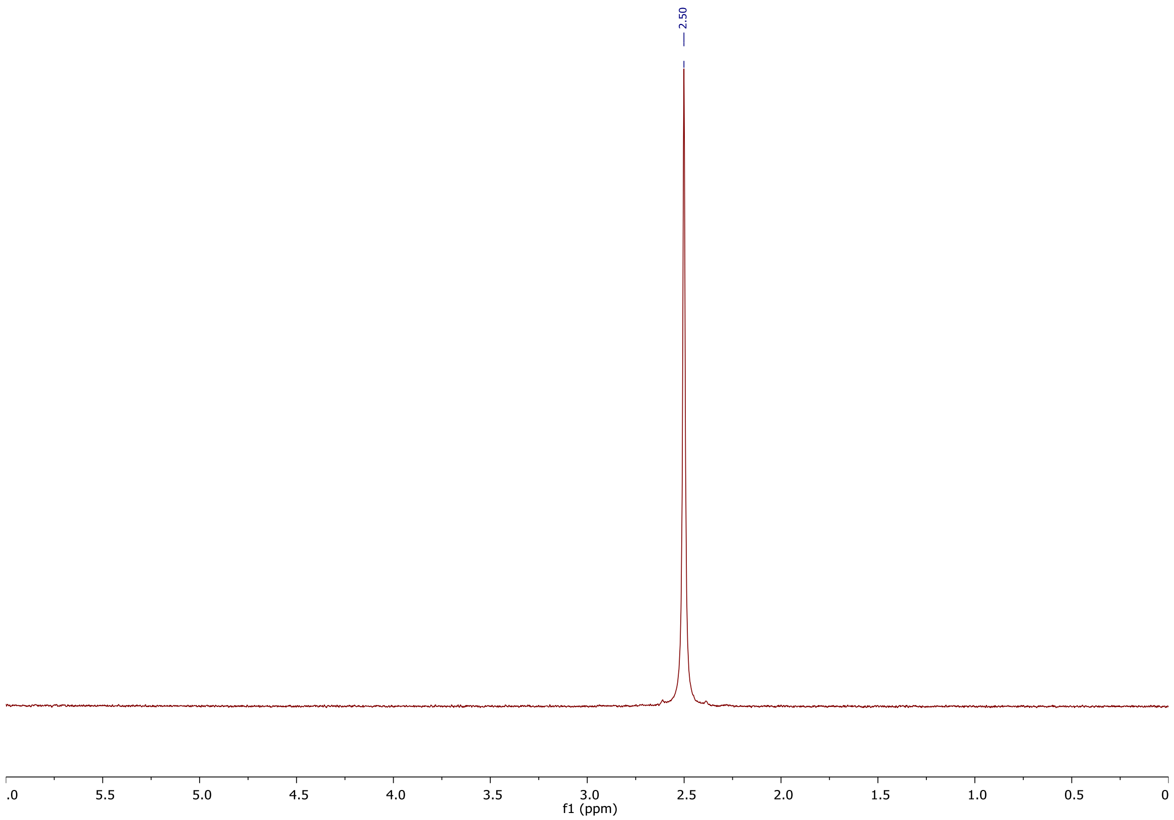

Figure S40 ^{2}H NMR spectrum of ***d*$_{\beta}$-2b** (92 MHz, $CDCl_3$).

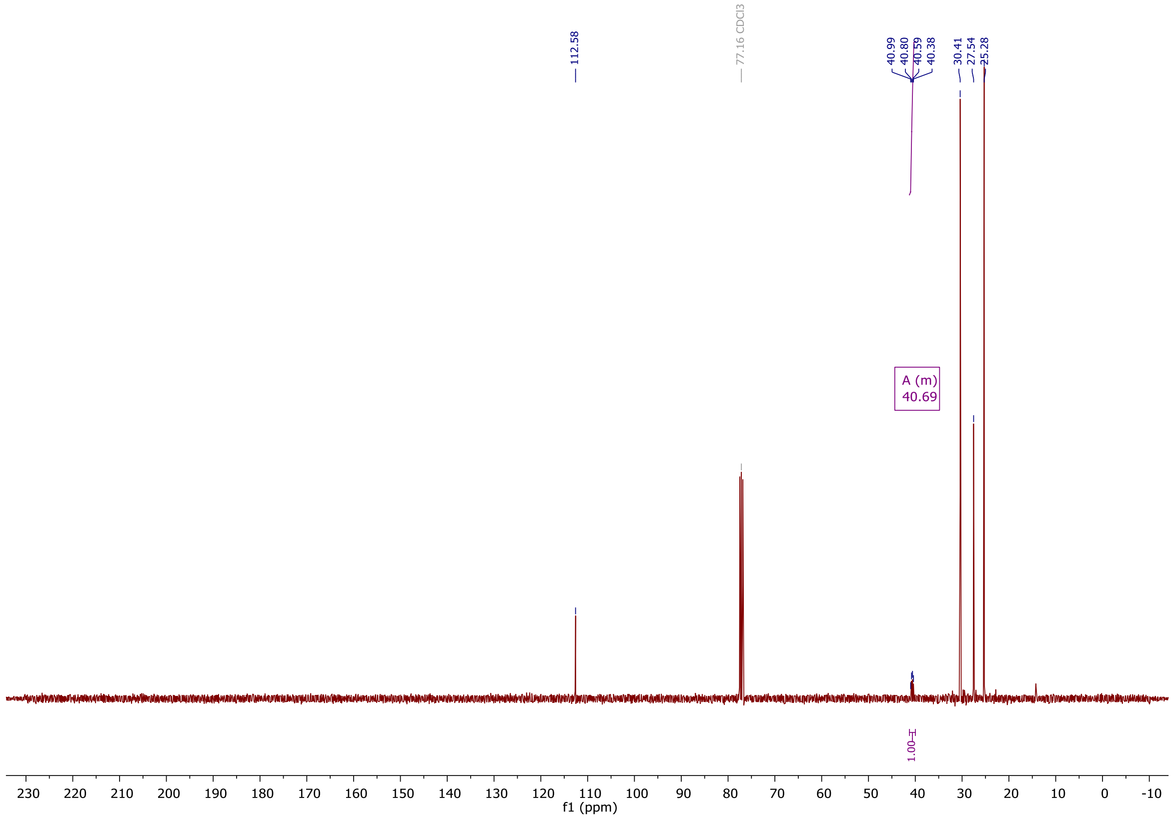

Figure S41 ^{13}C NMR spectrum of ***d*$_{\beta}$-2b** (101 MHz, $CDCl_3$).

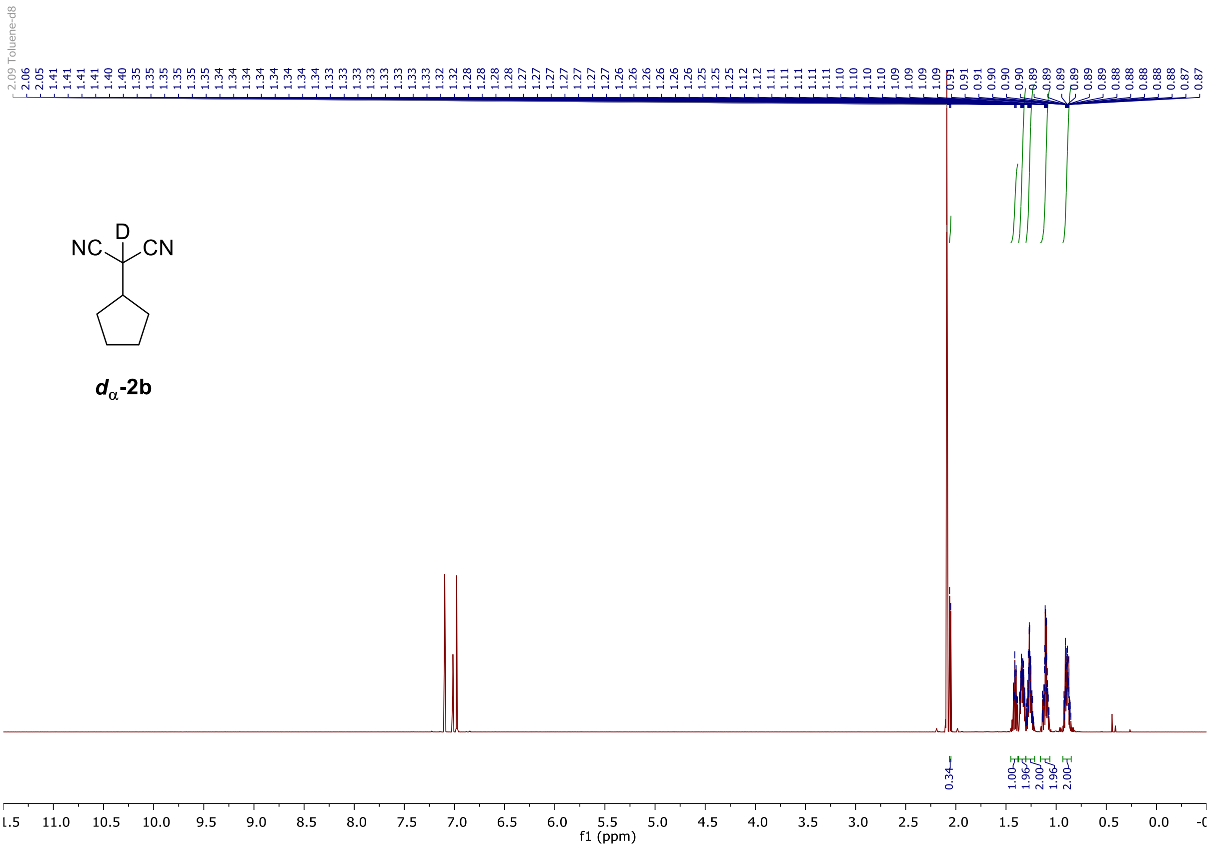

Figure S42 ^{1}H NMR spectrum of $\boldsymbol{d_\alpha}$**-2b** (600 MHz, d_8-toluene).

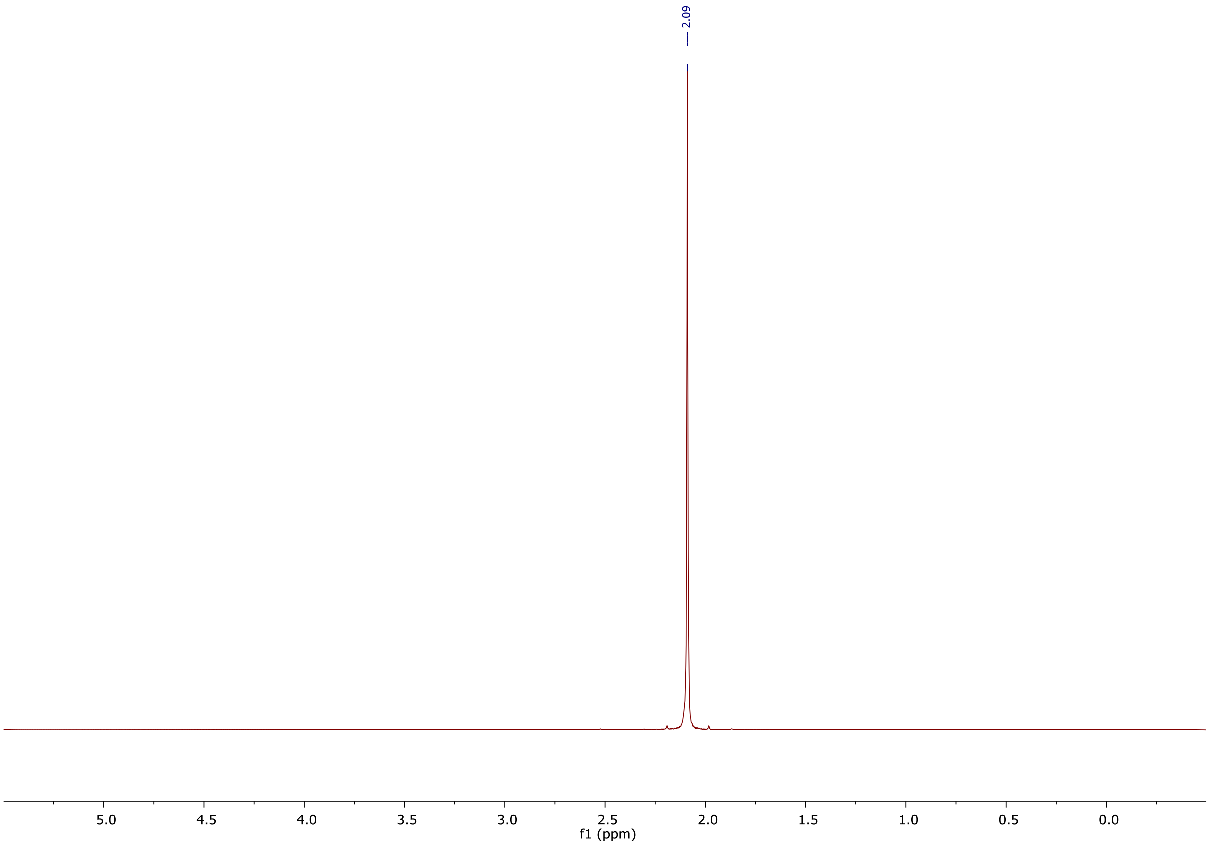

Figure S43 ^{2}H NMR spectrum of $\boldsymbol{d_\alpha}$**-2b** (92 MHz, d_8-toluene).

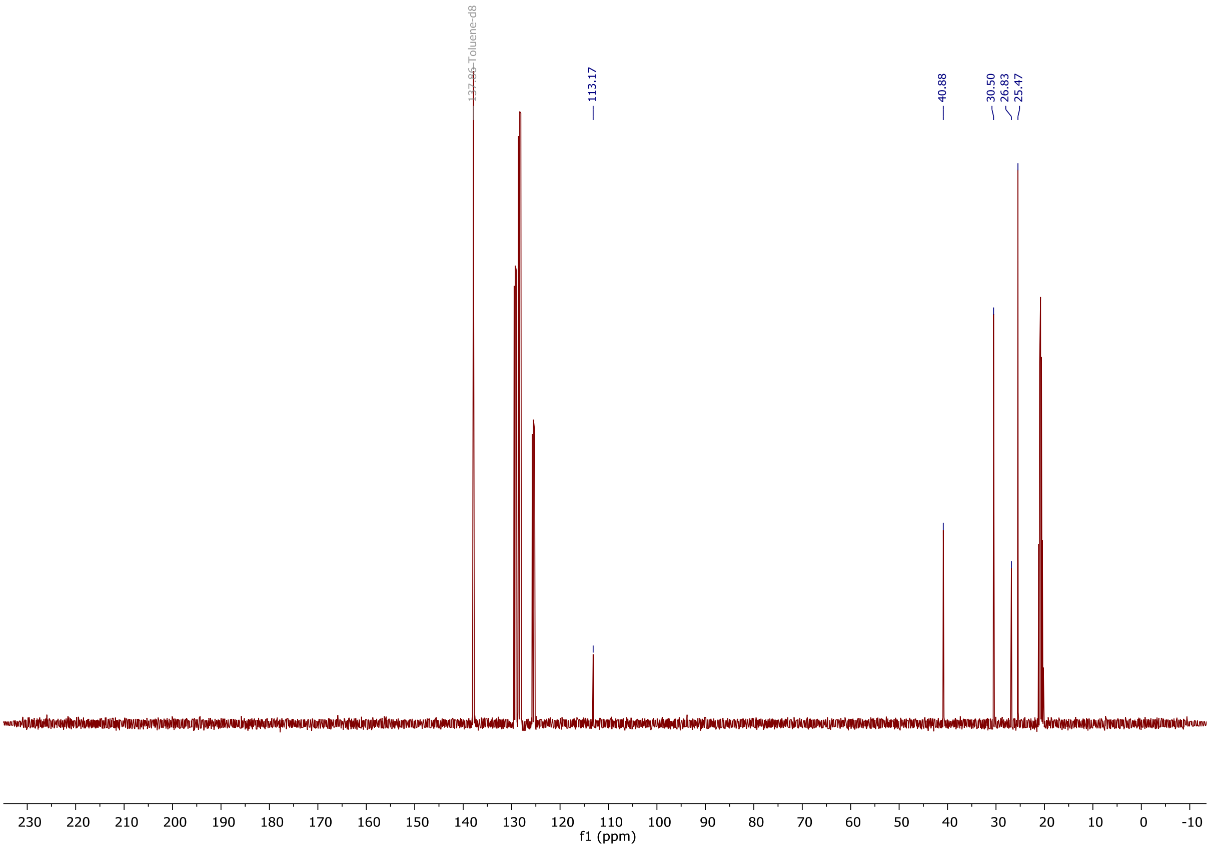

Figure S44 ^{13}C NMR spectrum of ***d*$_{\alpha}$-2b** (100 MHz, d_8-toluene).

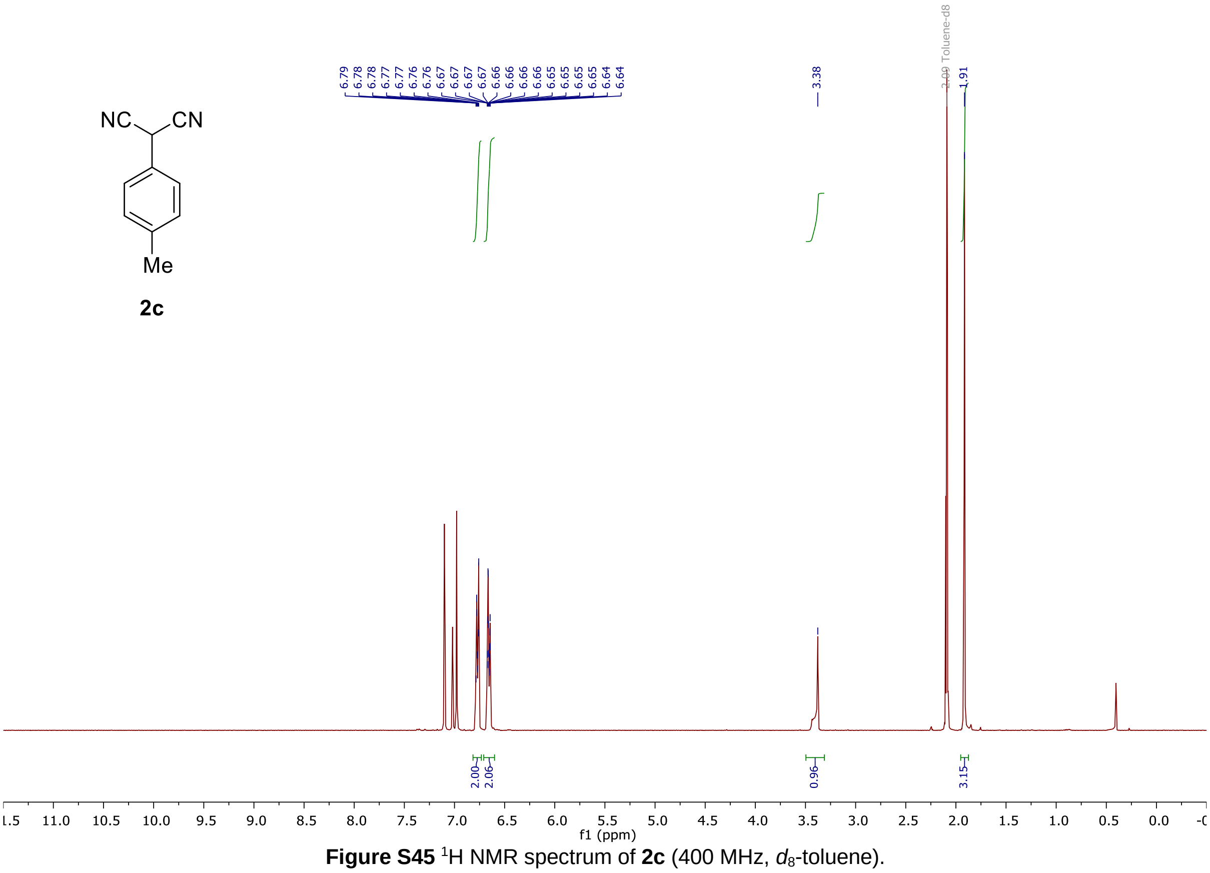

Figure S45 ^{1}H NMR spectrum of **2c** (400 MHz, d_8-toluene).

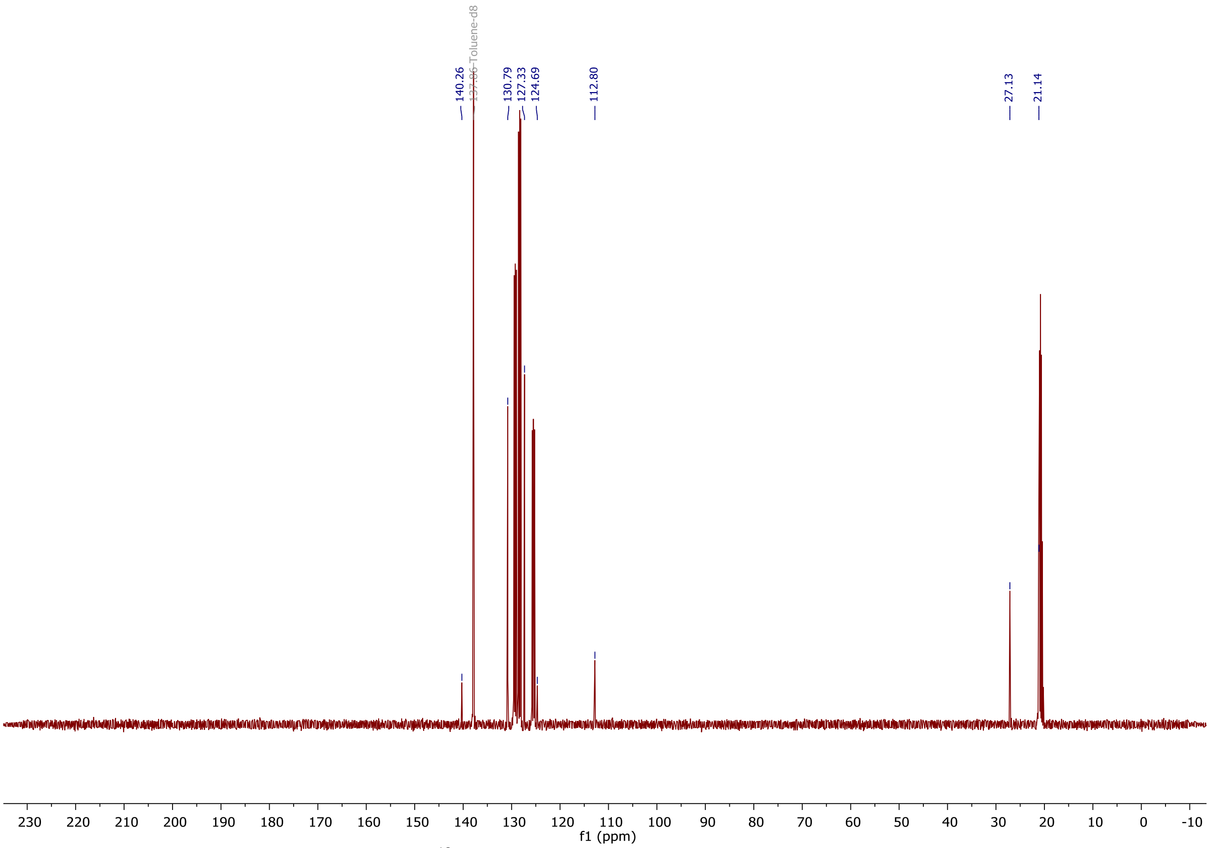

Figure S46 ^{13}C NMR spectrum of **2c** (100 MHz, d_8-toluene).

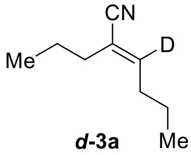

***d*-3a**

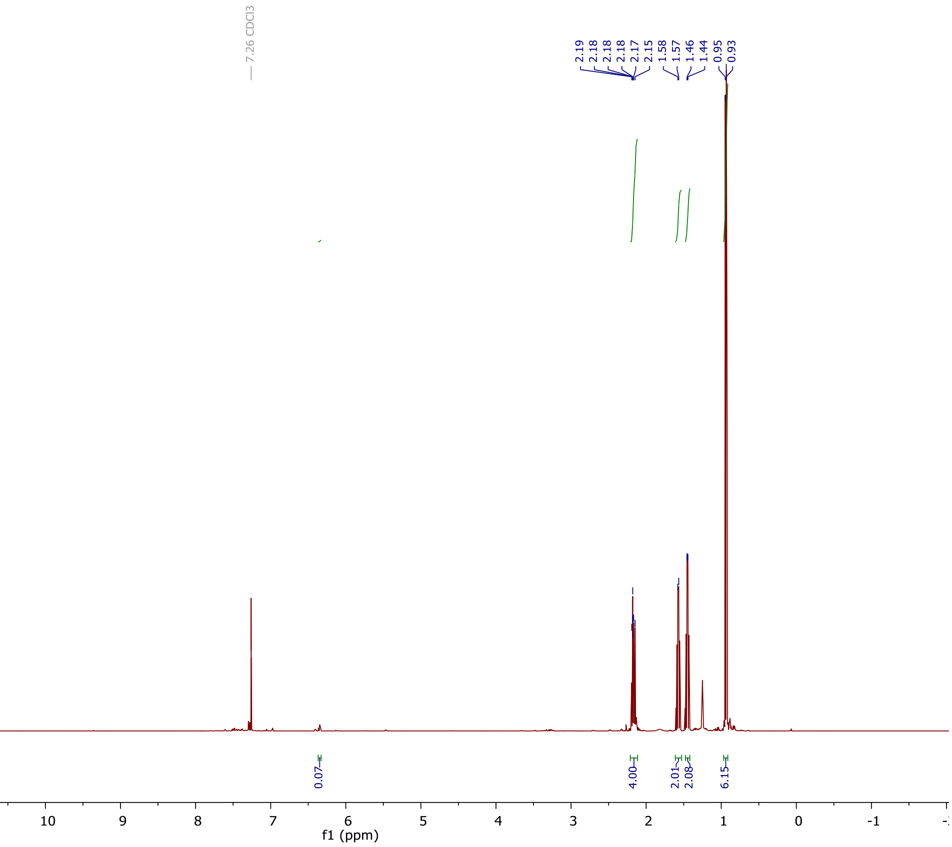

Figure S47 ^{1}H NMR spectrum of ***d*-3a** (600 MHz, $CDCl_3$).

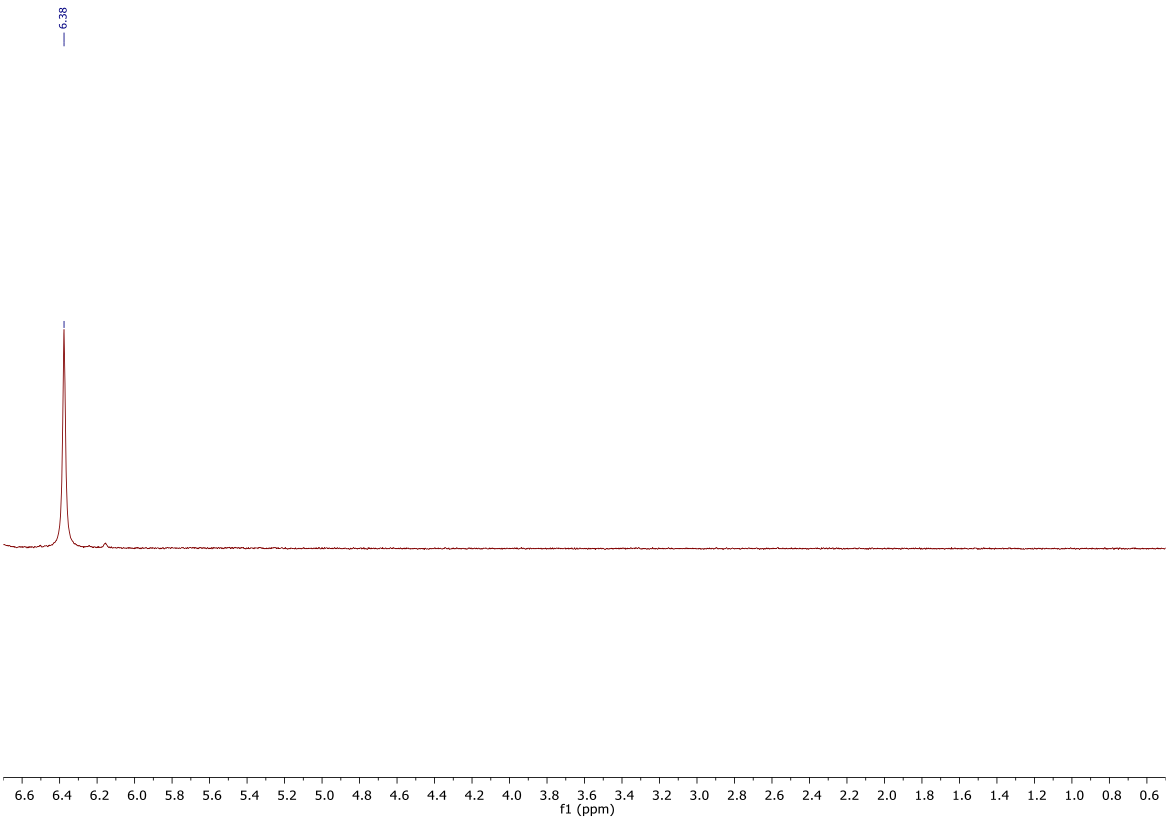

Figure S48 ^{2}H NMR spectrum of ***d*-3a** (92 MHz, $CDCl_3$).

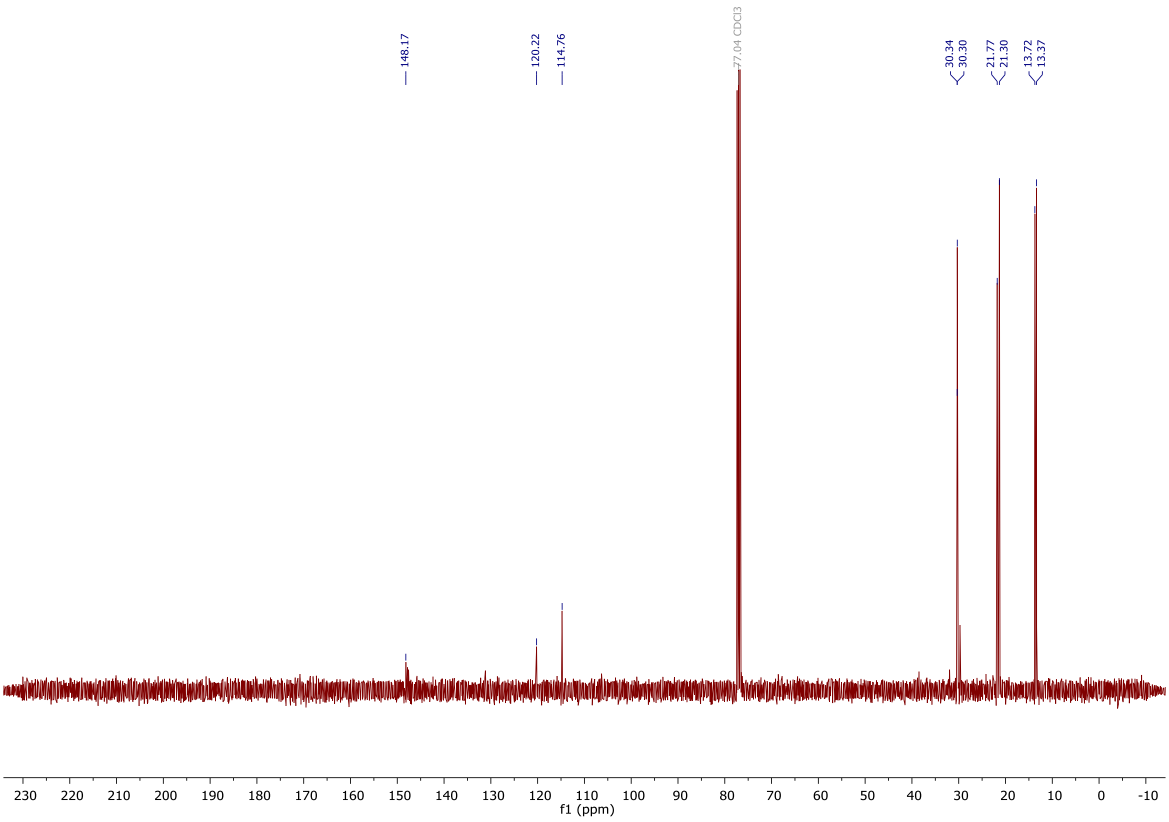

Figure S49 ^{13}C NMR spectrum of ***d*-3a** (101 MHz, $CDCl_3$).

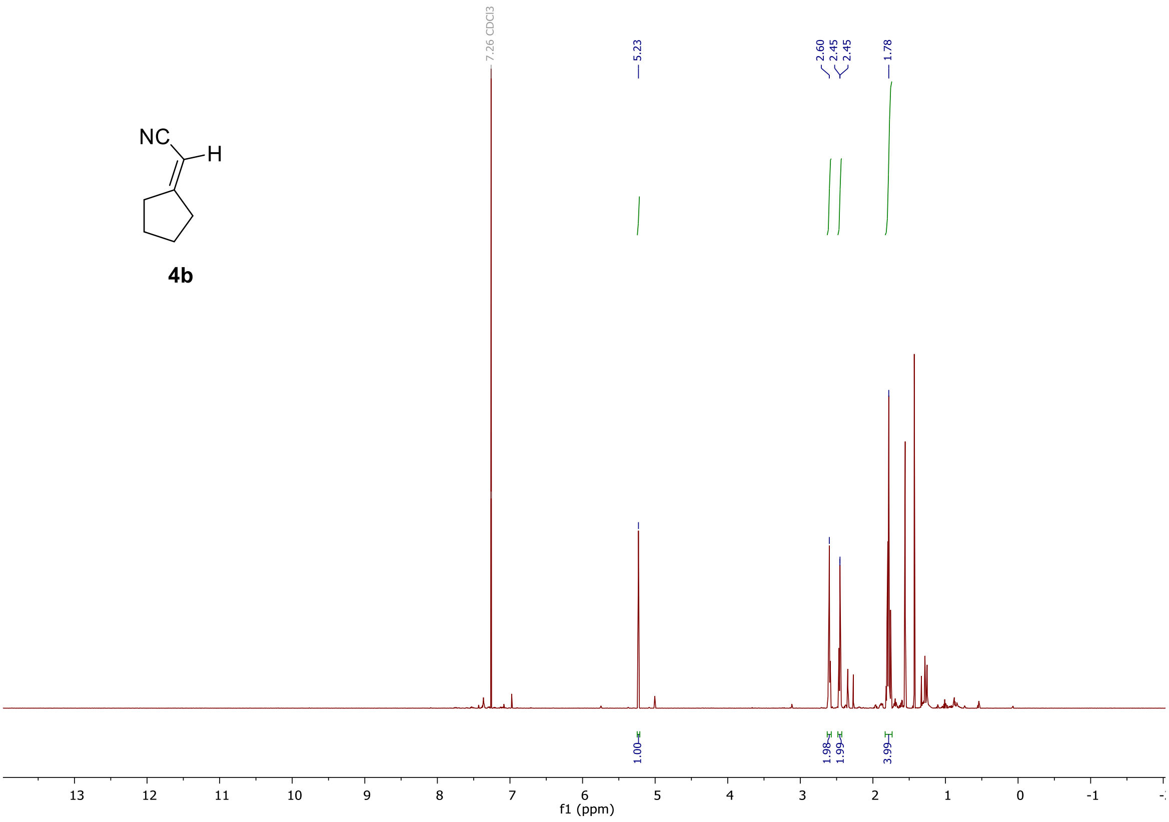

Figure S50 ^{1}H NMR spectrum of **4b** (600 MHz, $CDCl_3$).

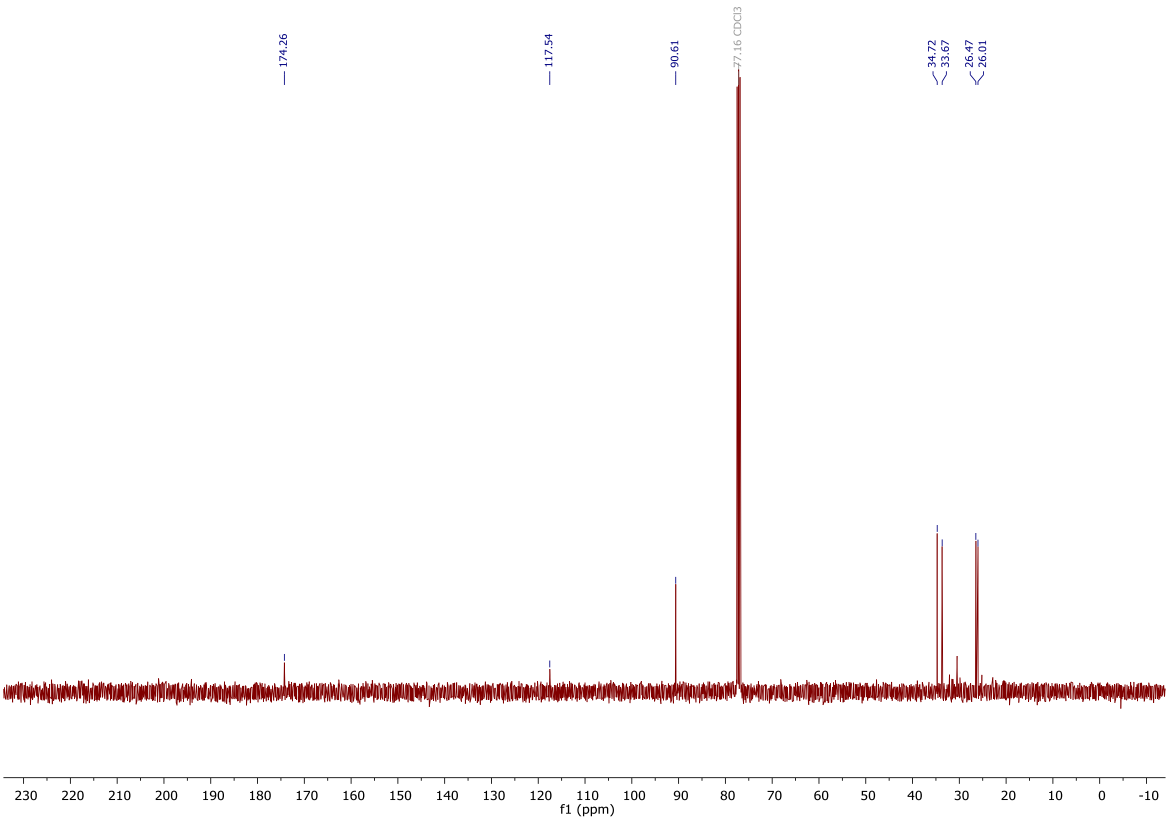

Figure S51 ^{13}C NMR spectrum of **4b** (101 MHz, $CDCl_3$).

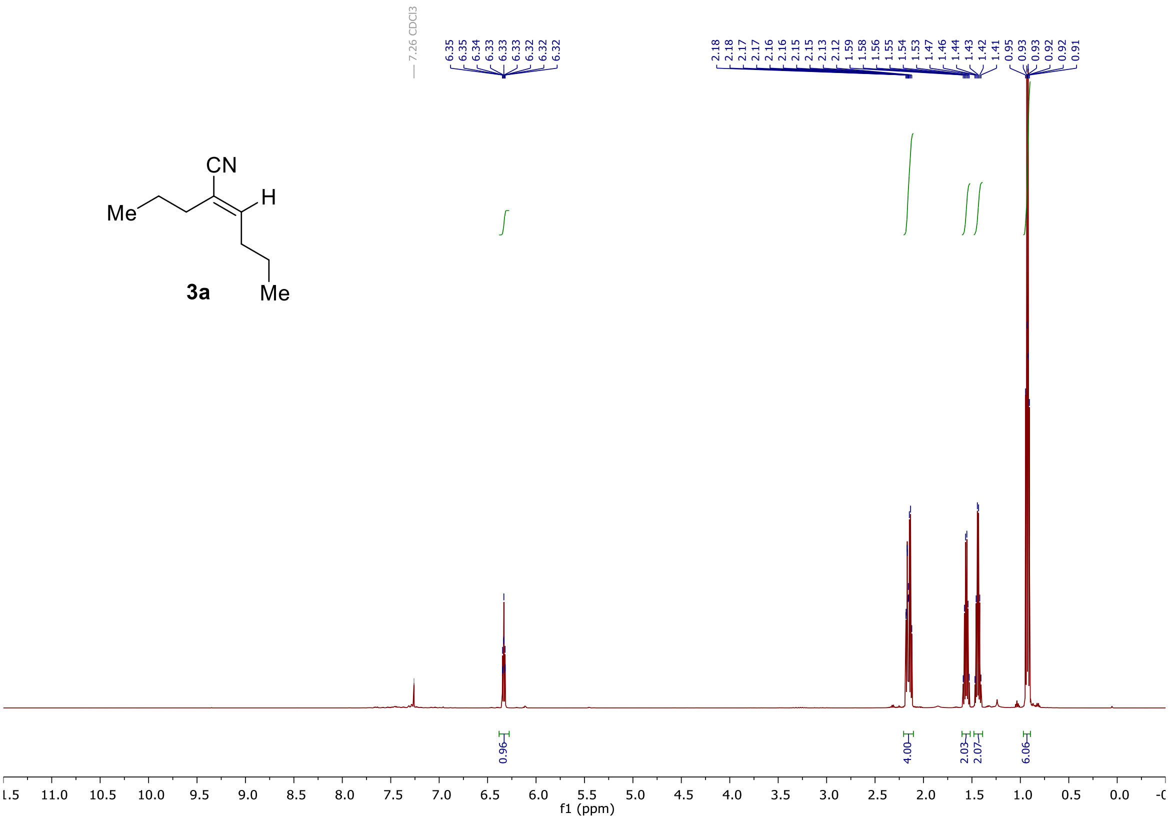

Figure S52 ^{1}H NMR spectrum of **3a** (600 MHz, $CDCl_3$).

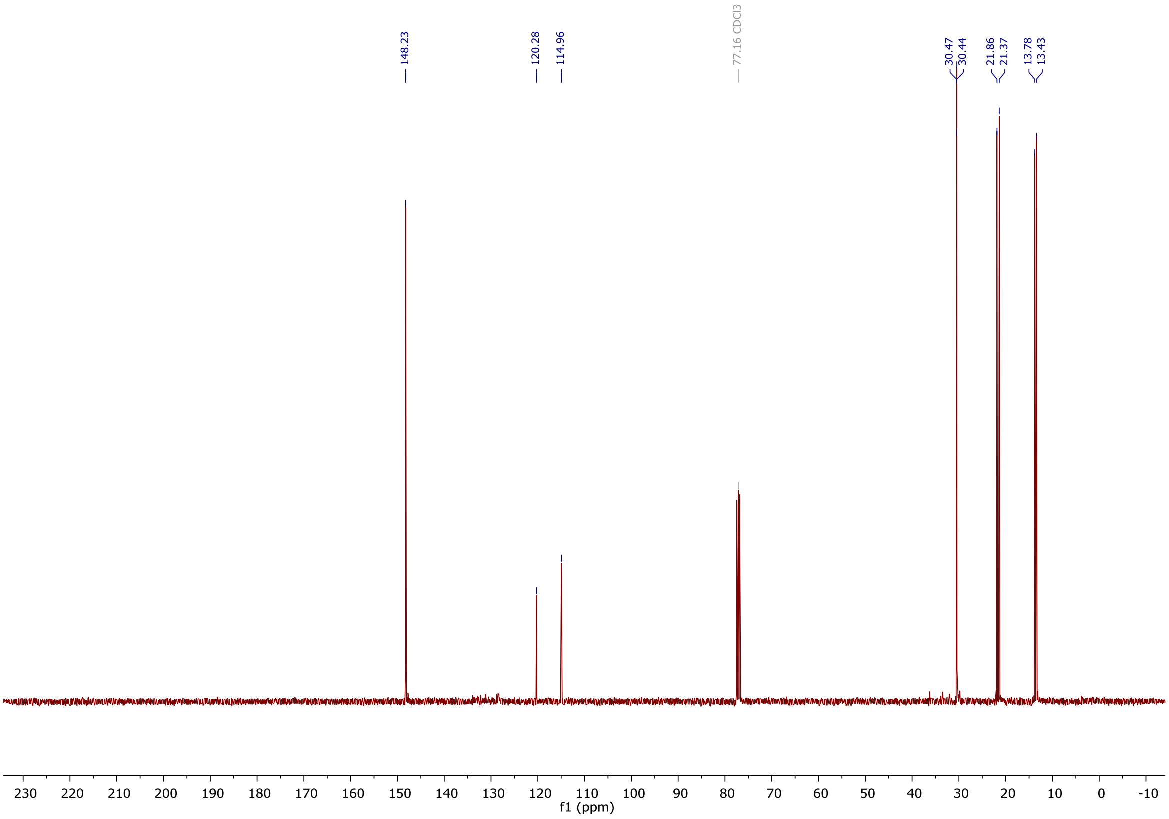

Figure S53 ^{13}C NMR spectrum of **3a** (101 MHz, $CDCl_3$).

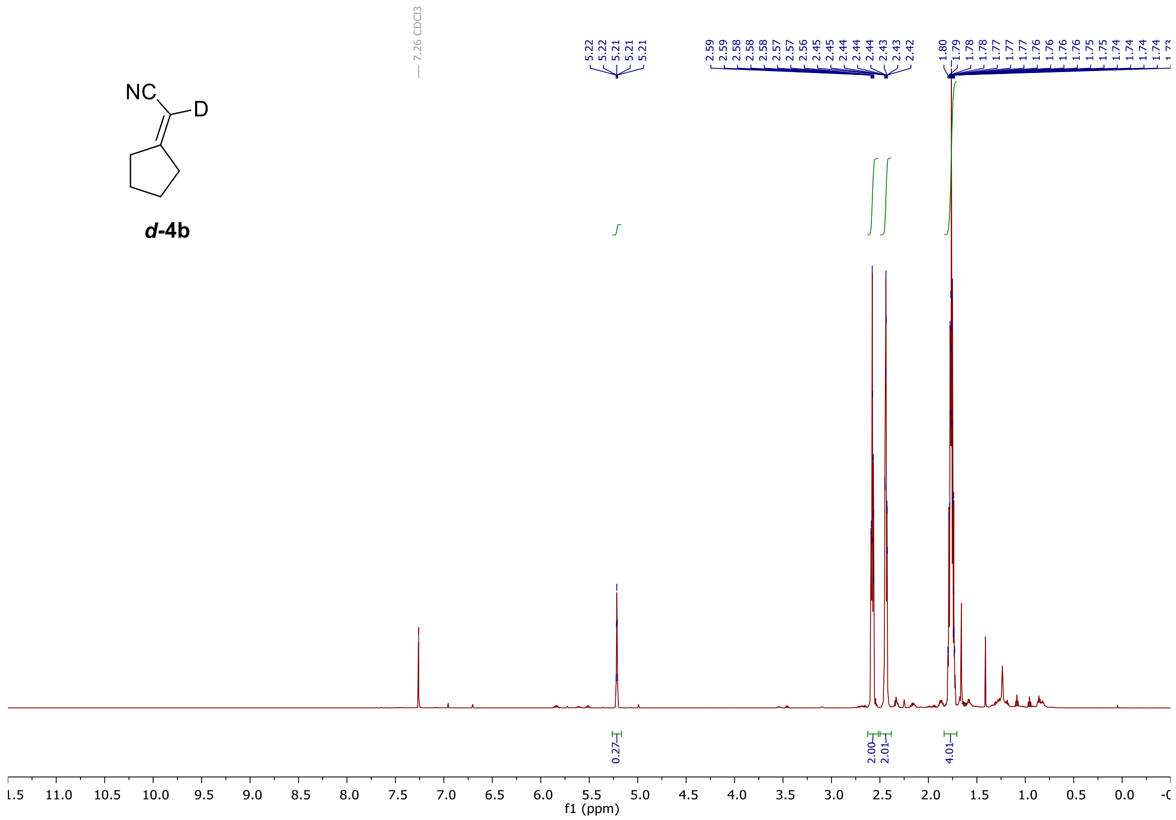

Figure S54 ^{1}H NMR spectrum of ***d*-4b** (600 MHz, $CDCl_3$).

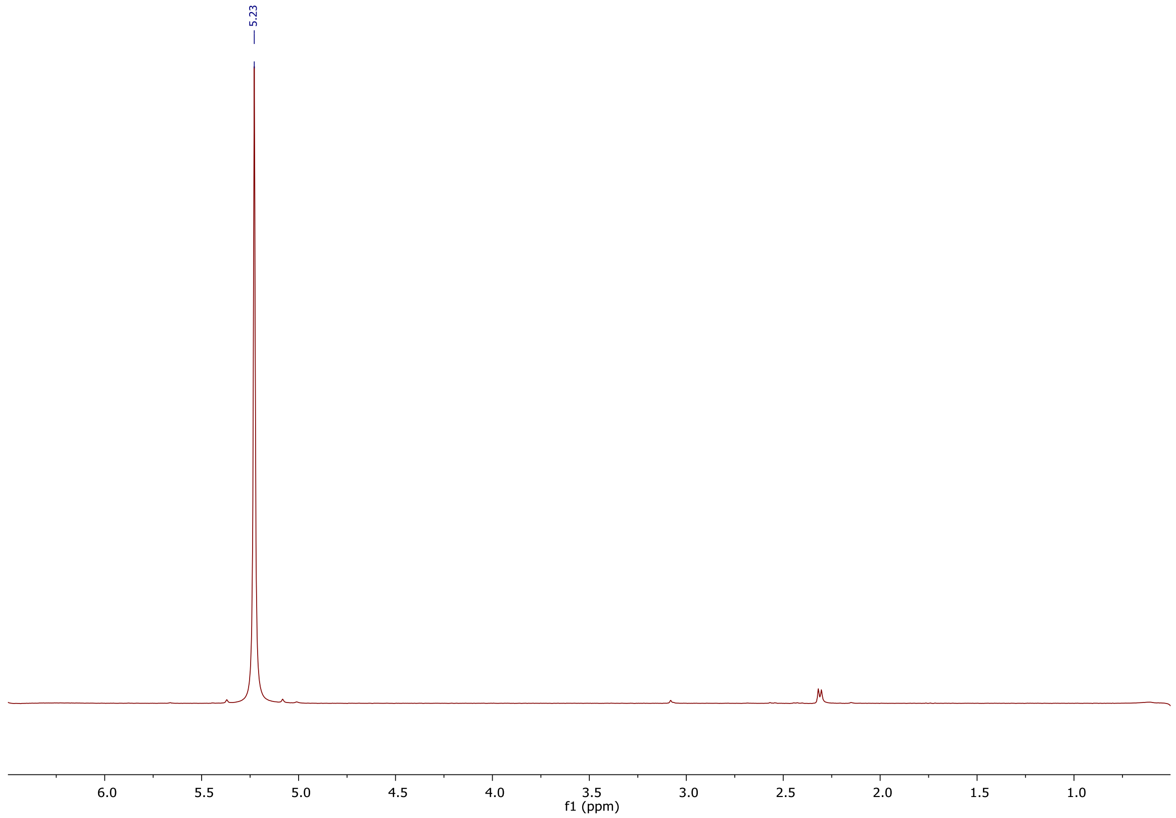

Figure S55 ^{2}H NMR spectrum of ***d*-4b** (92 MHz, $CDCl_3$).

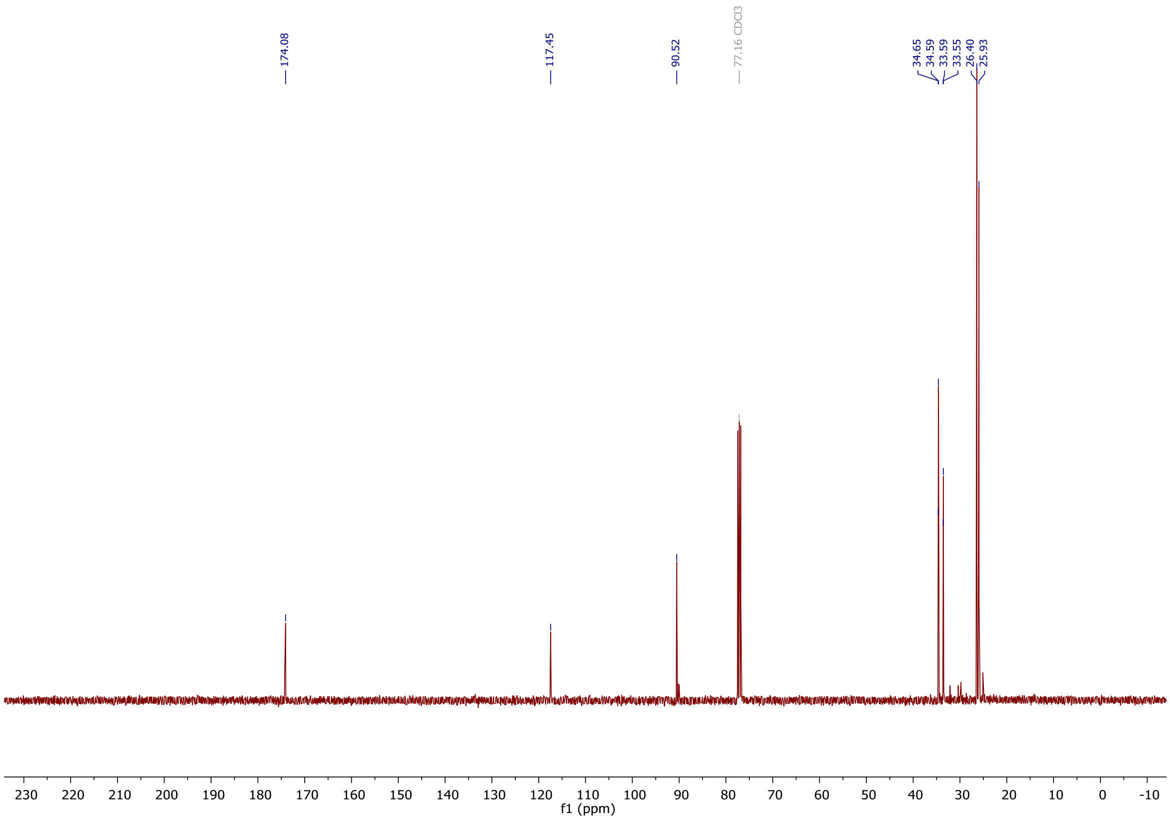

Figure S56 ^{13}C NMR spectrum of ***d*-4b** (101 MHz, $CDCl_3$).

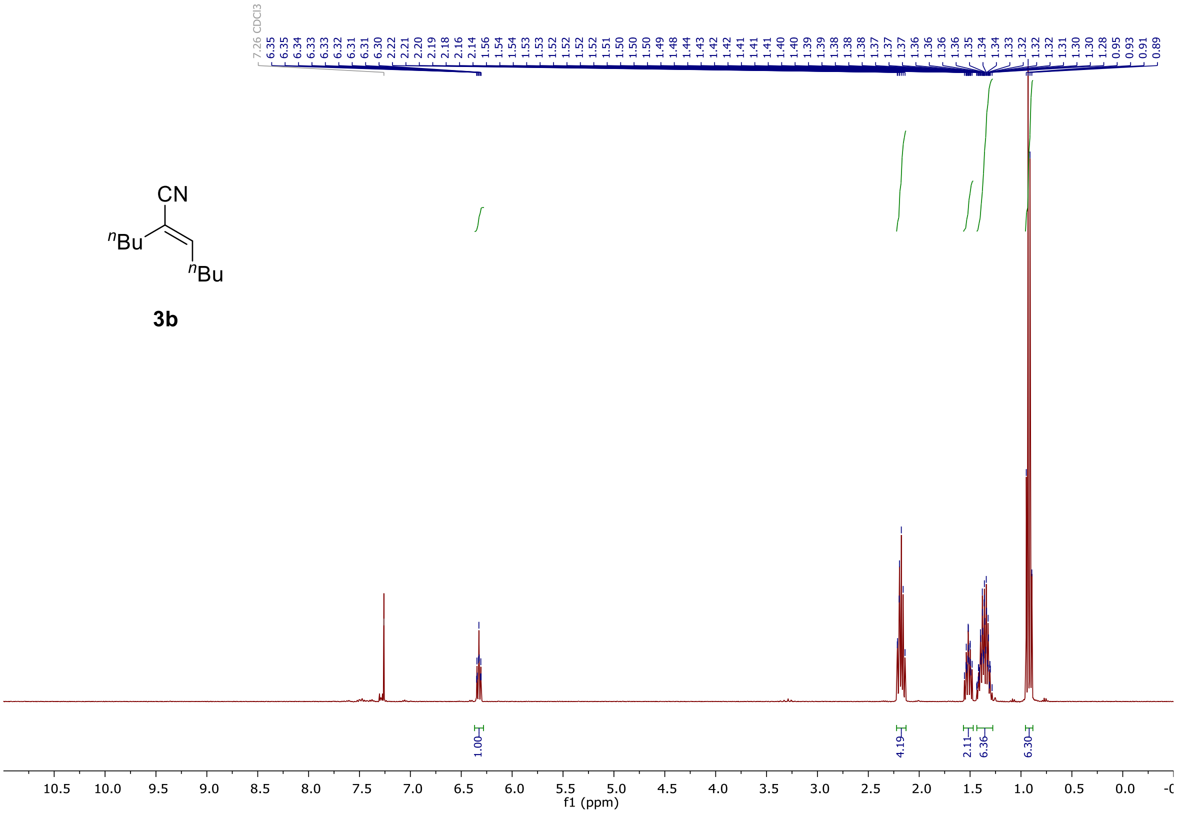

Figure S57 ^{1}H NMR spectrum of **3b** (400 MHz, $CDCl_3$).

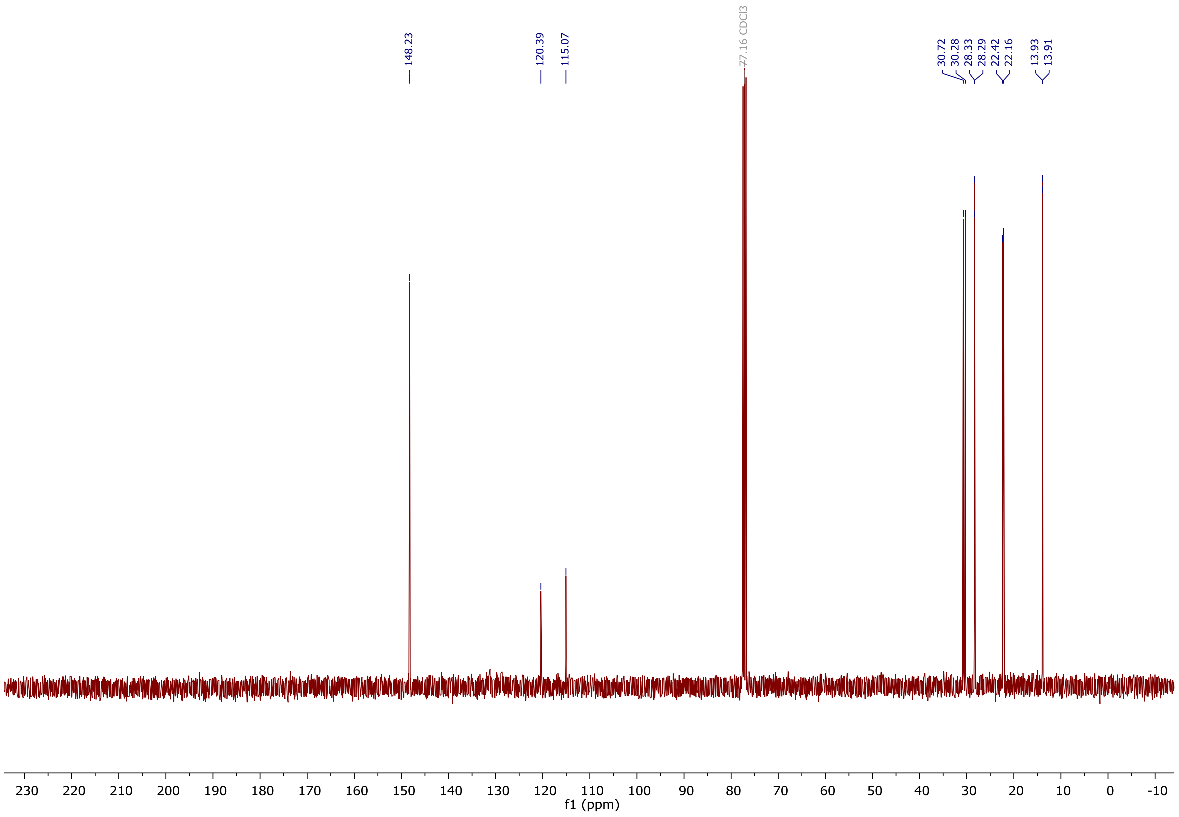

Figure S58 ^{13}C NMR spectrum of **3b** (101 MHz, $CDCl_3$).

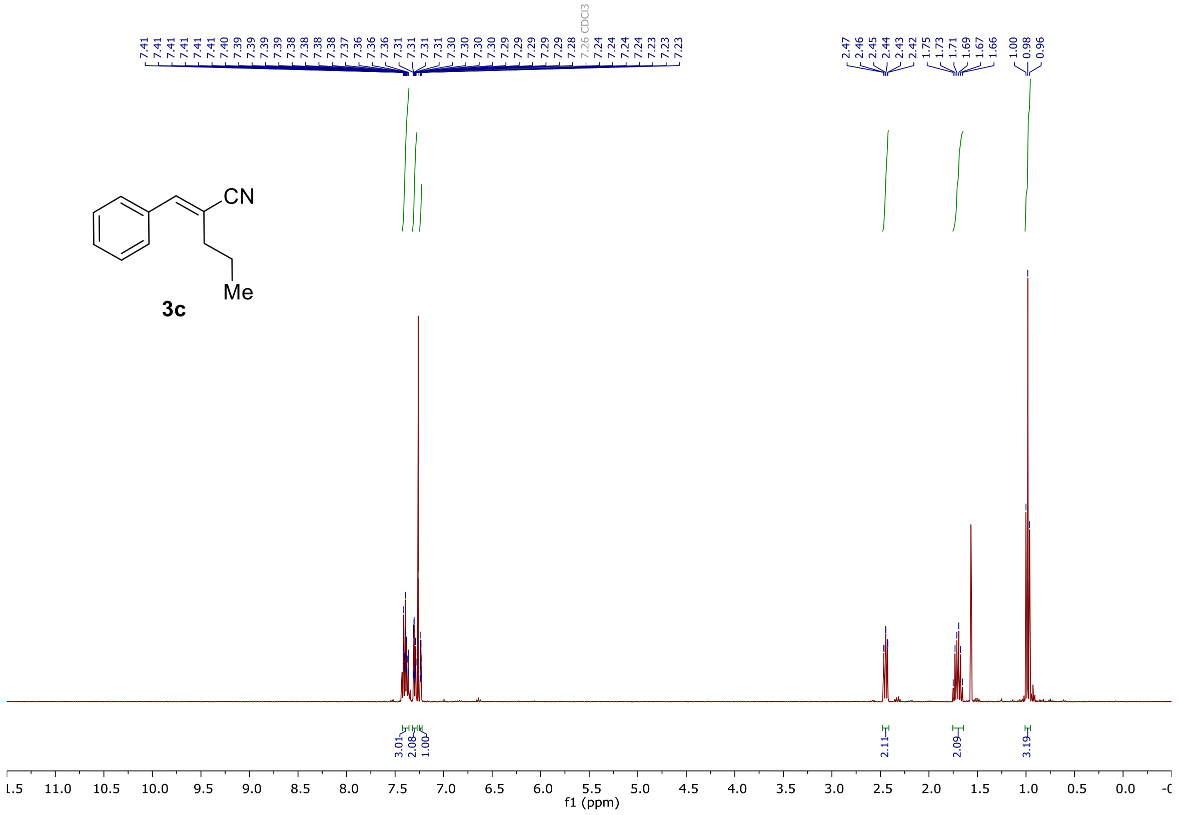

Figure S59 ^{1}H NMR spectrum of **3c** (400 MHz, $CDCl_3$).

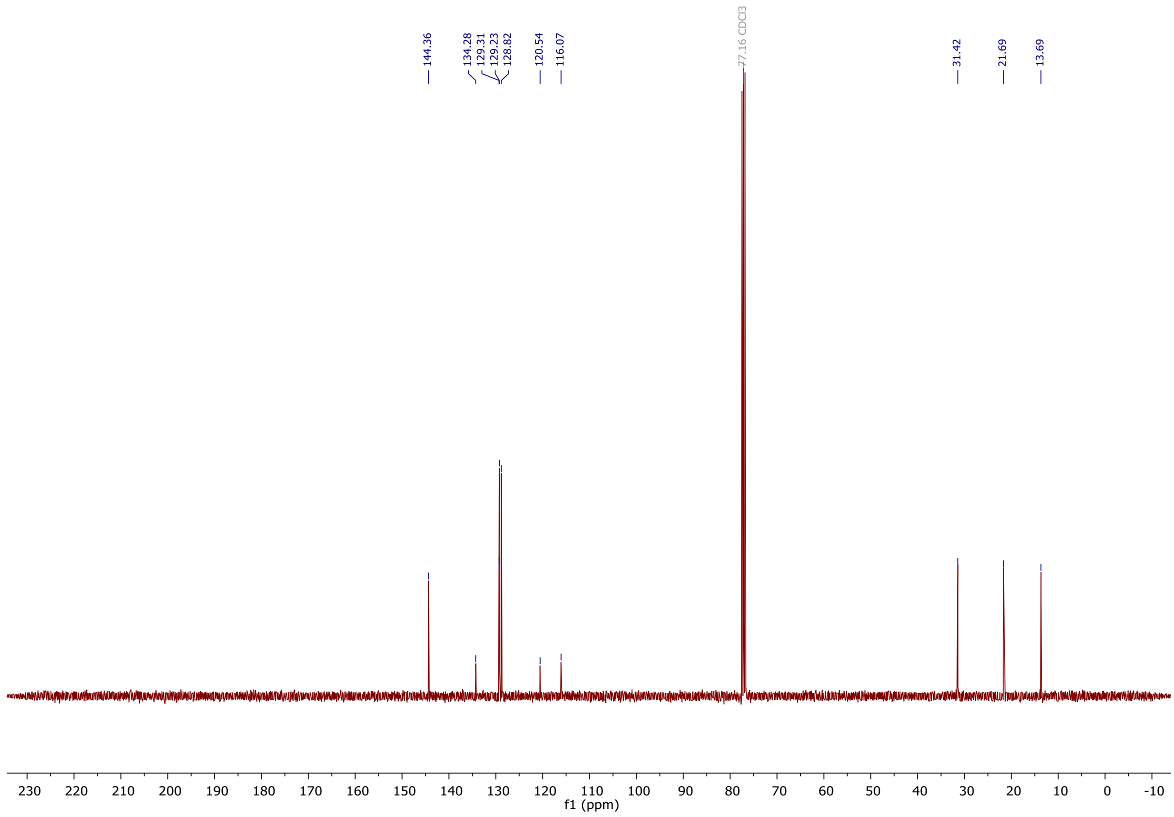

Figure S60 ^{13}C NMR spectrum of **3c** (101 MHz, $CDCl_3$).

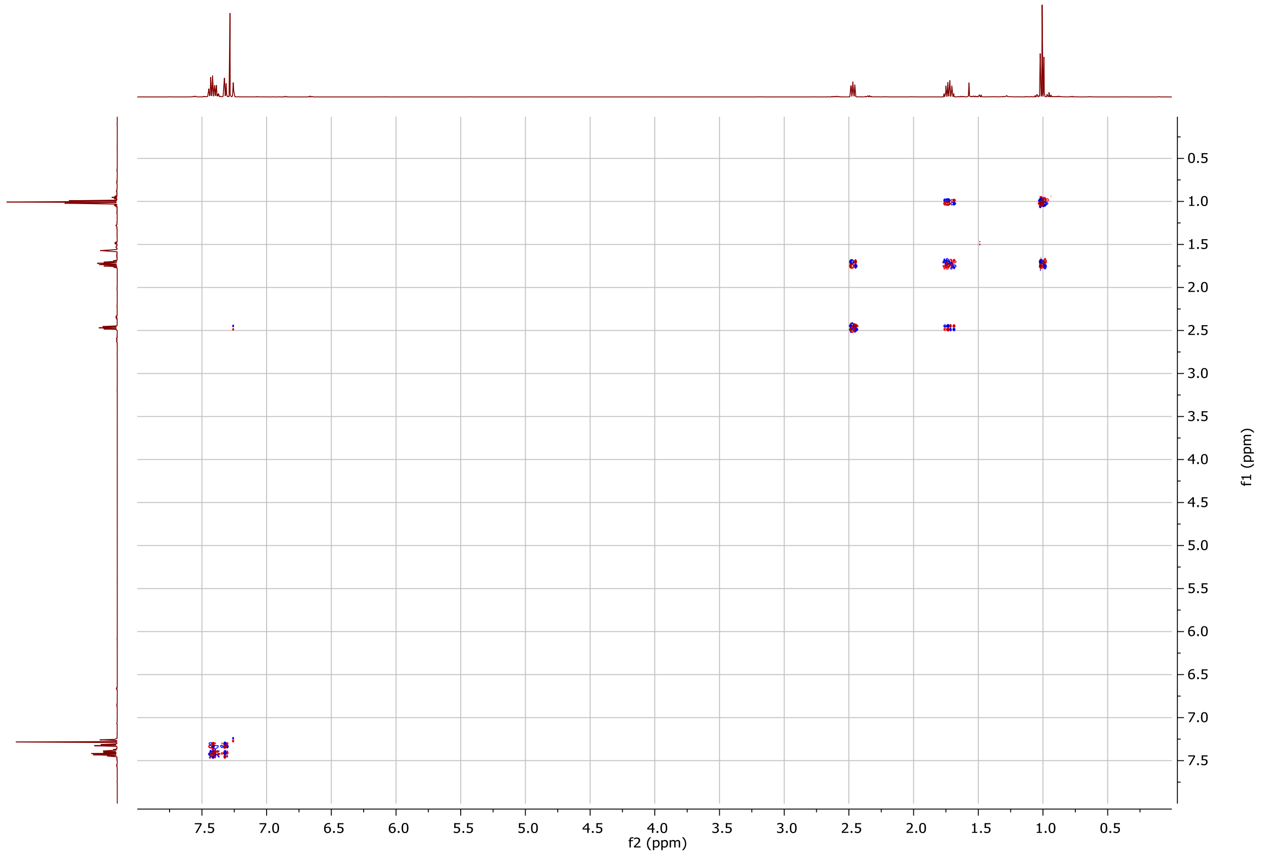

Figure S61 DQF-COSY NMR spectrum of **3c** (500 MHz, $CDCl_3$).

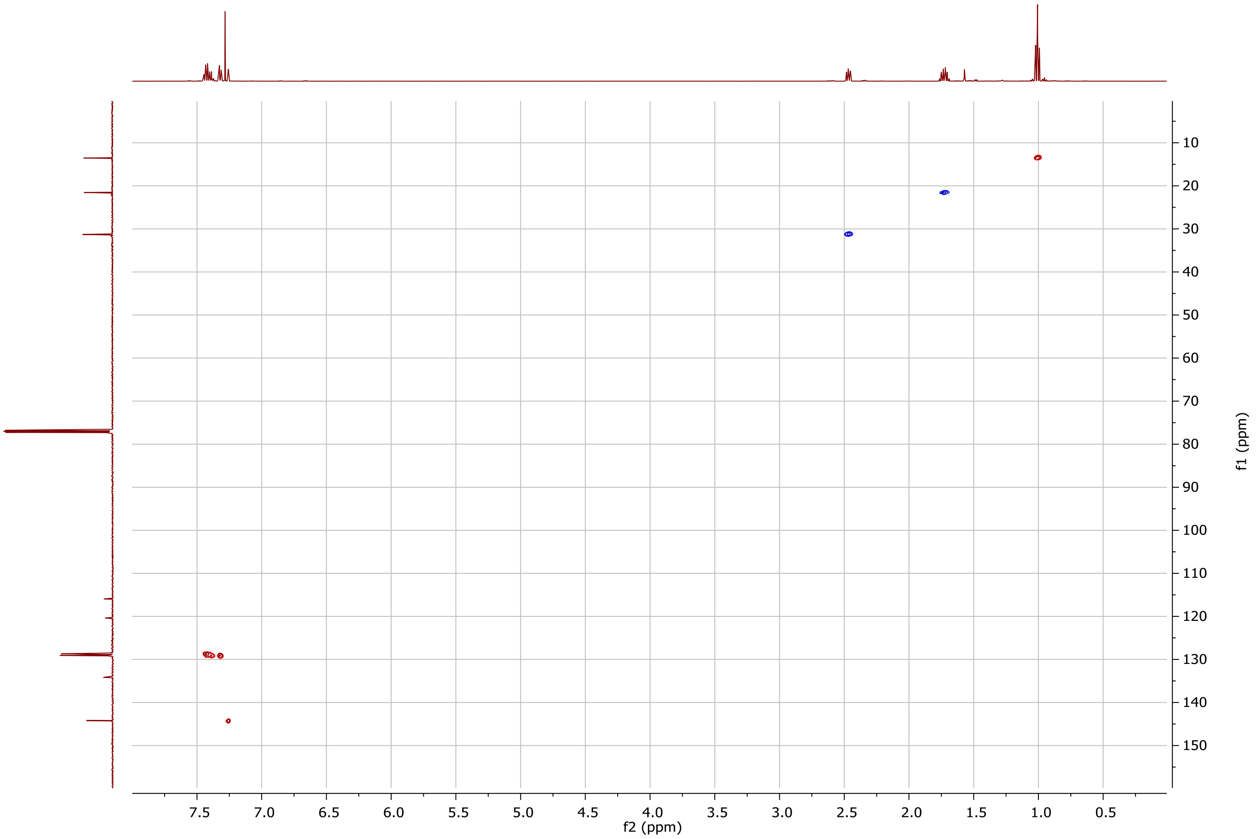

Figure S62 HSQC NMR spectrum of **3c** (500 MHz, $CDCl_3$).

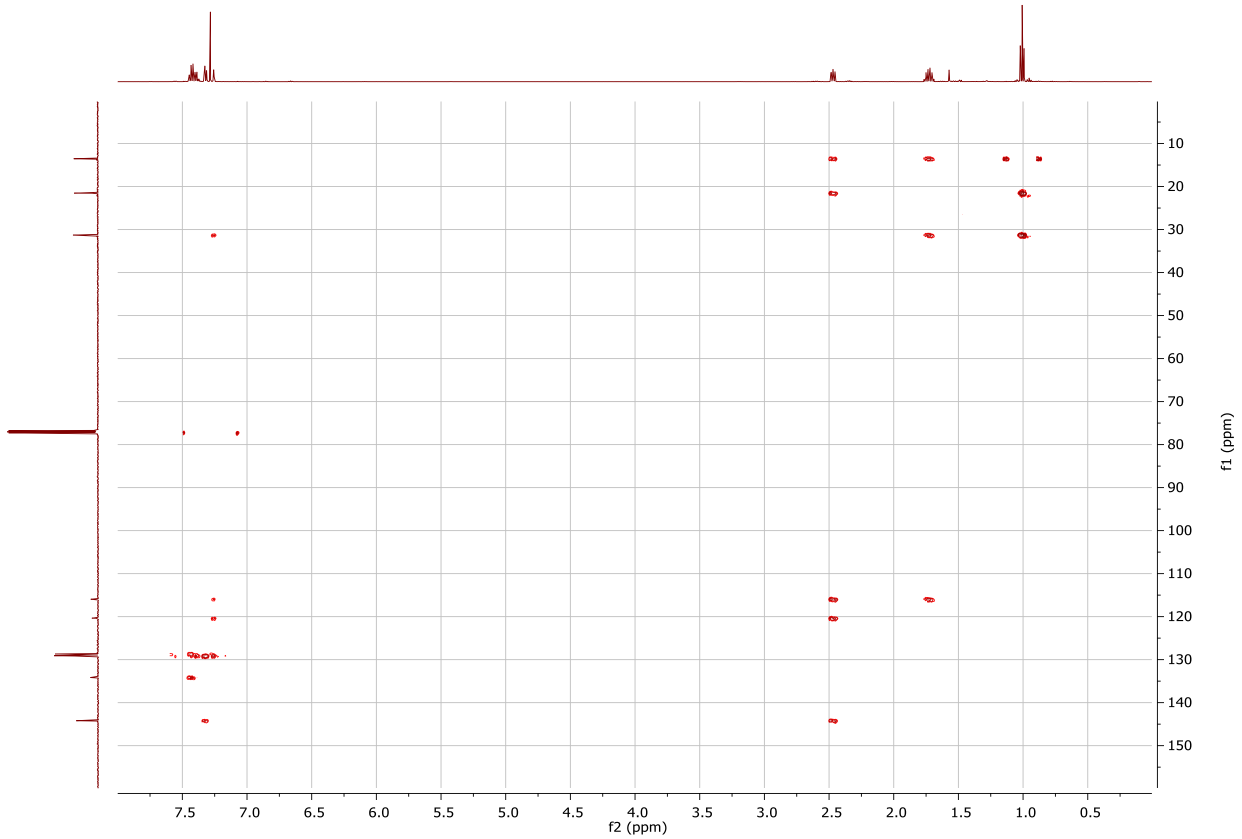

Figure S63 HMBC NMR spectrum of **3c** (500 MHz, $CDCl_3$).

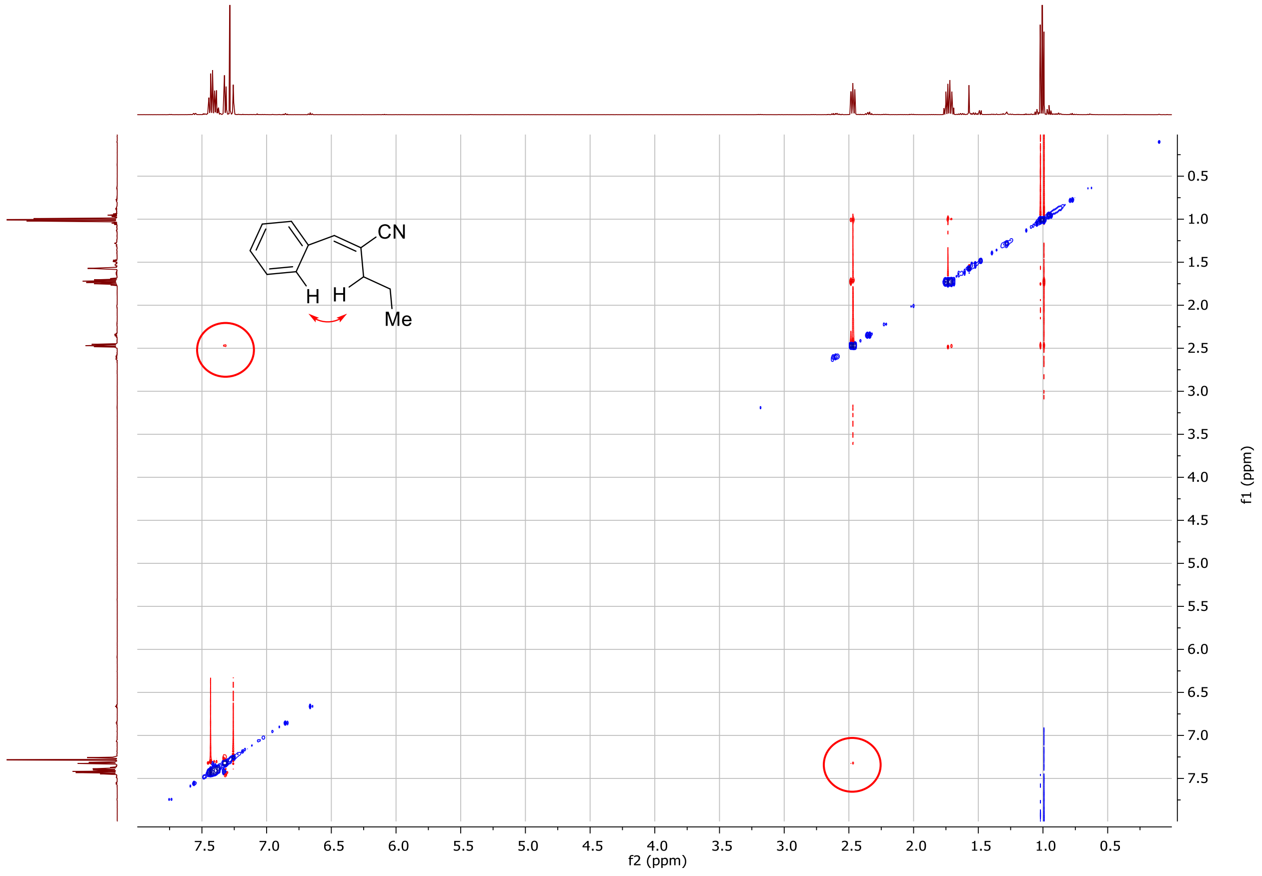

Figure S64 NOESY NMR spectrum of **3c** (500 MHz, $CDCl_3$).

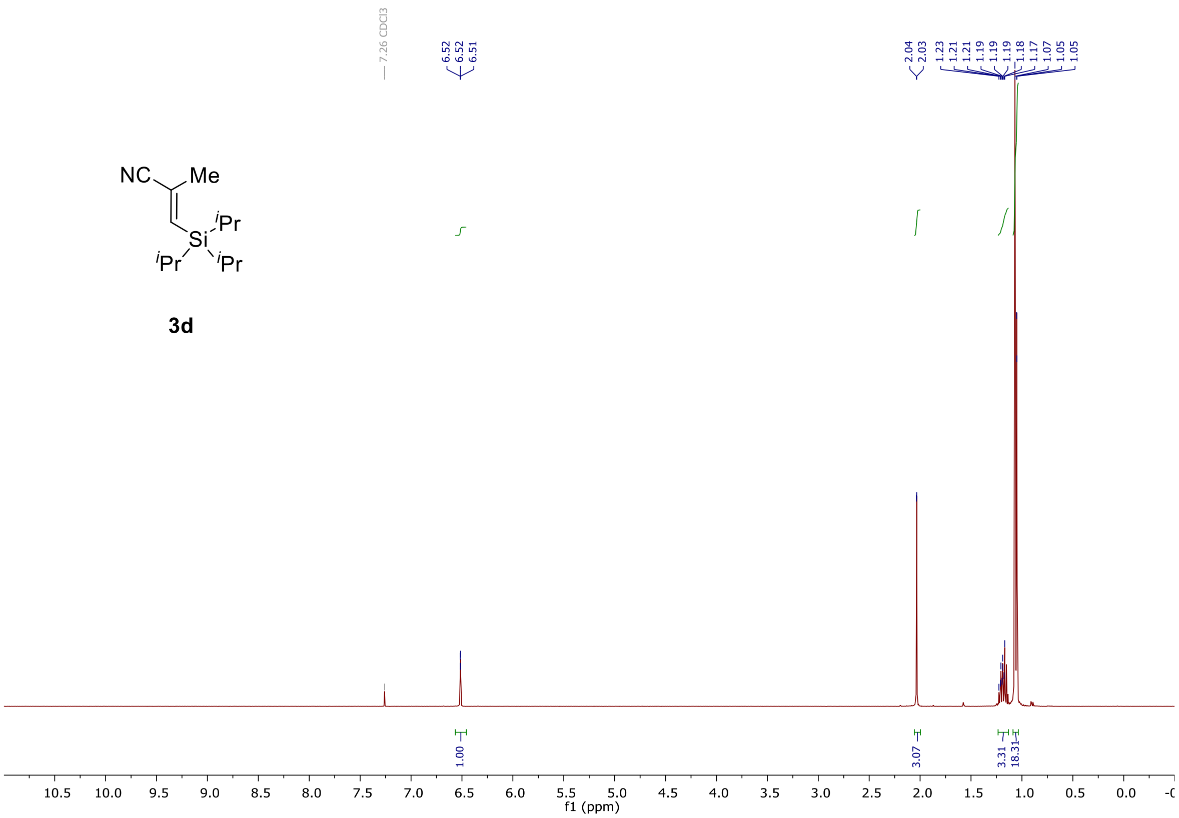

Figure S65 ^{1}H NMR spectrum of **3d** (400 MHz, $CDCl_3$).

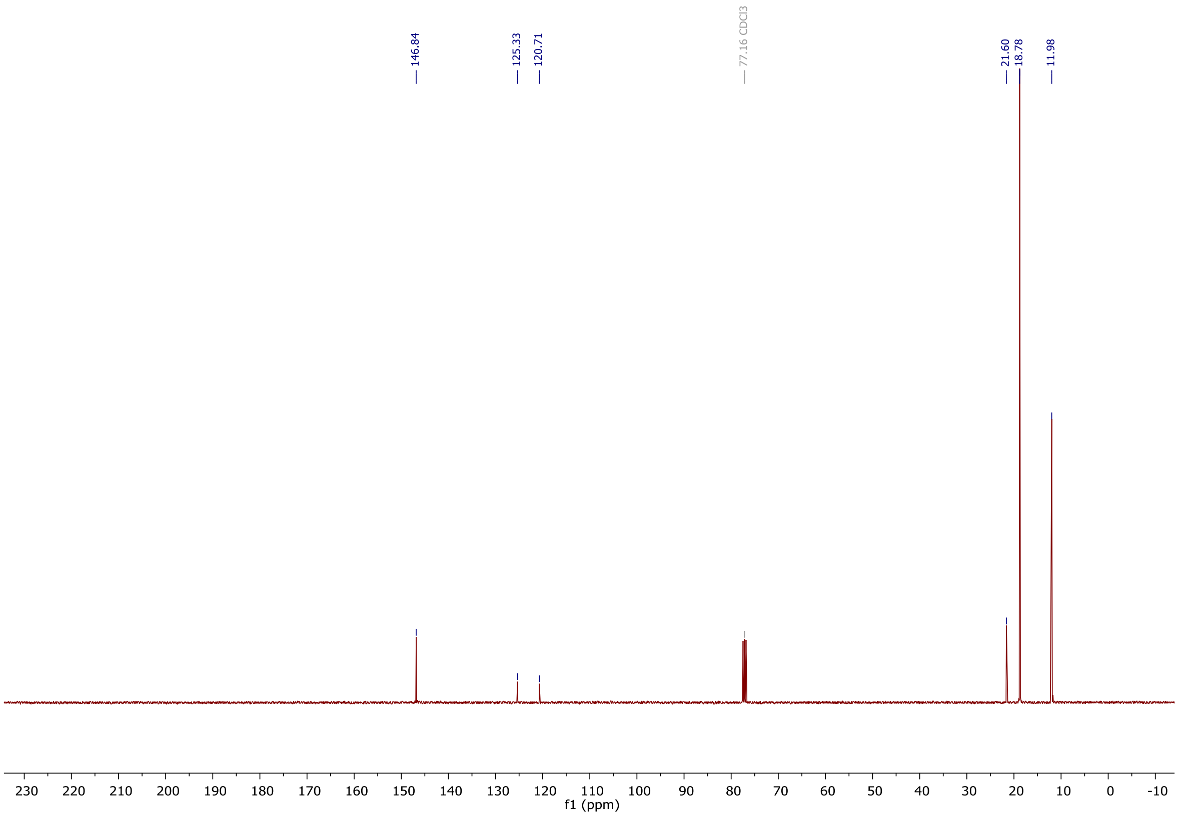

Figure S66 ^{13}C NMR spectrum of **3d** (101 MHz, $CDCl_3$).

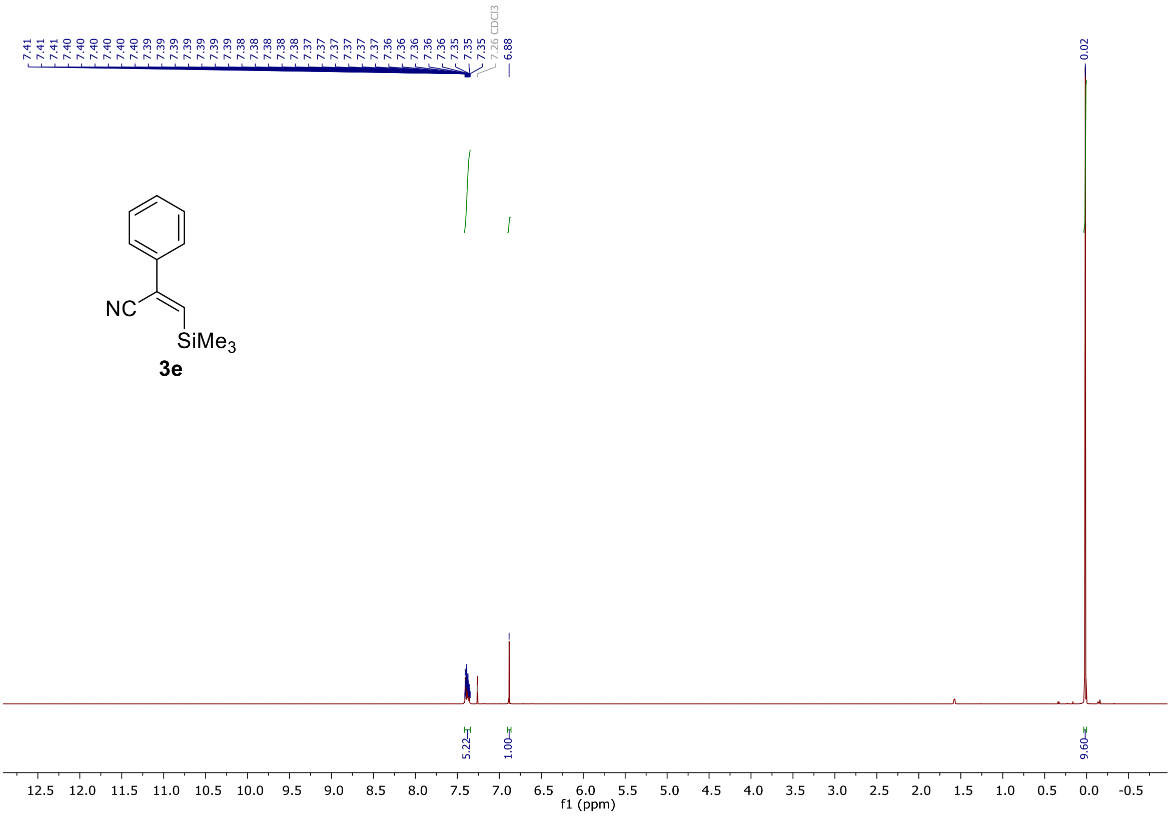

Figure S67 ^{1}H NMR spectrum of **3e** (400 MHz, $CDCl_3$).

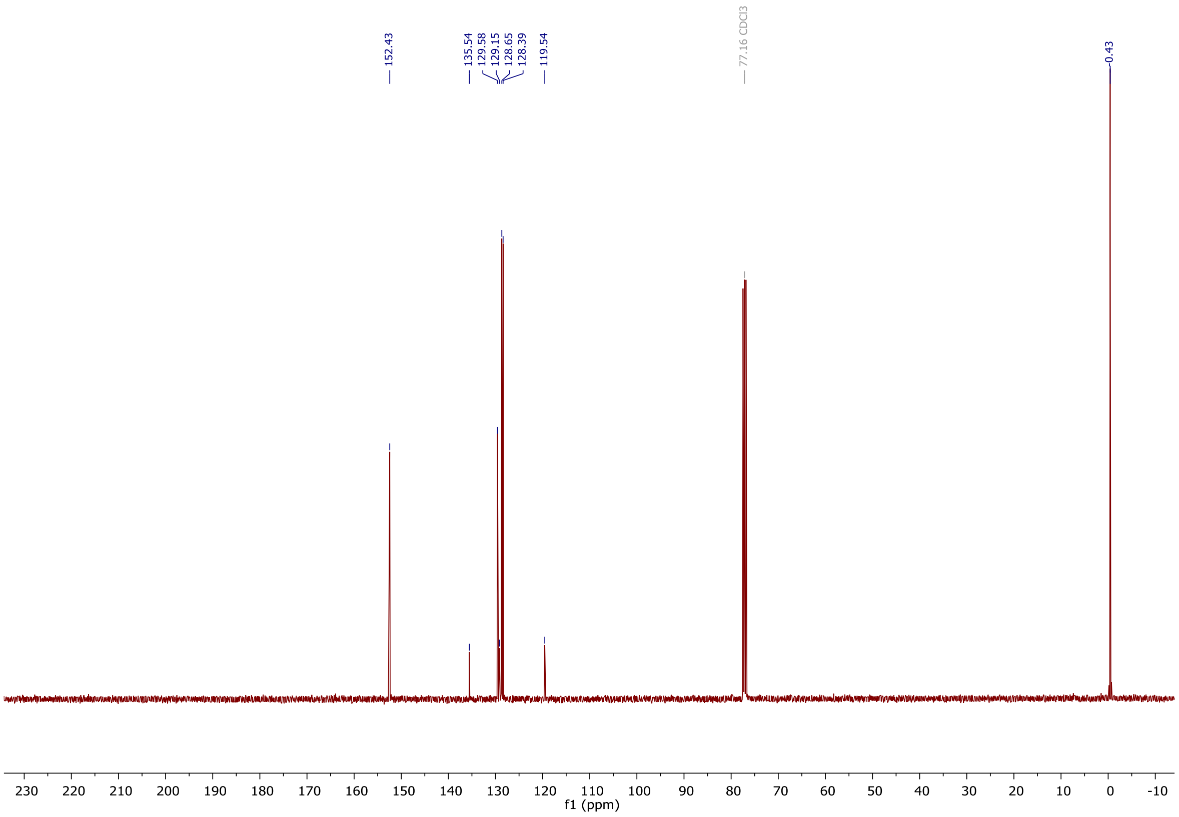

Figure S68 ^{13}C NMR spectrum of **3e** (101 MHz, $CDCl_3$).

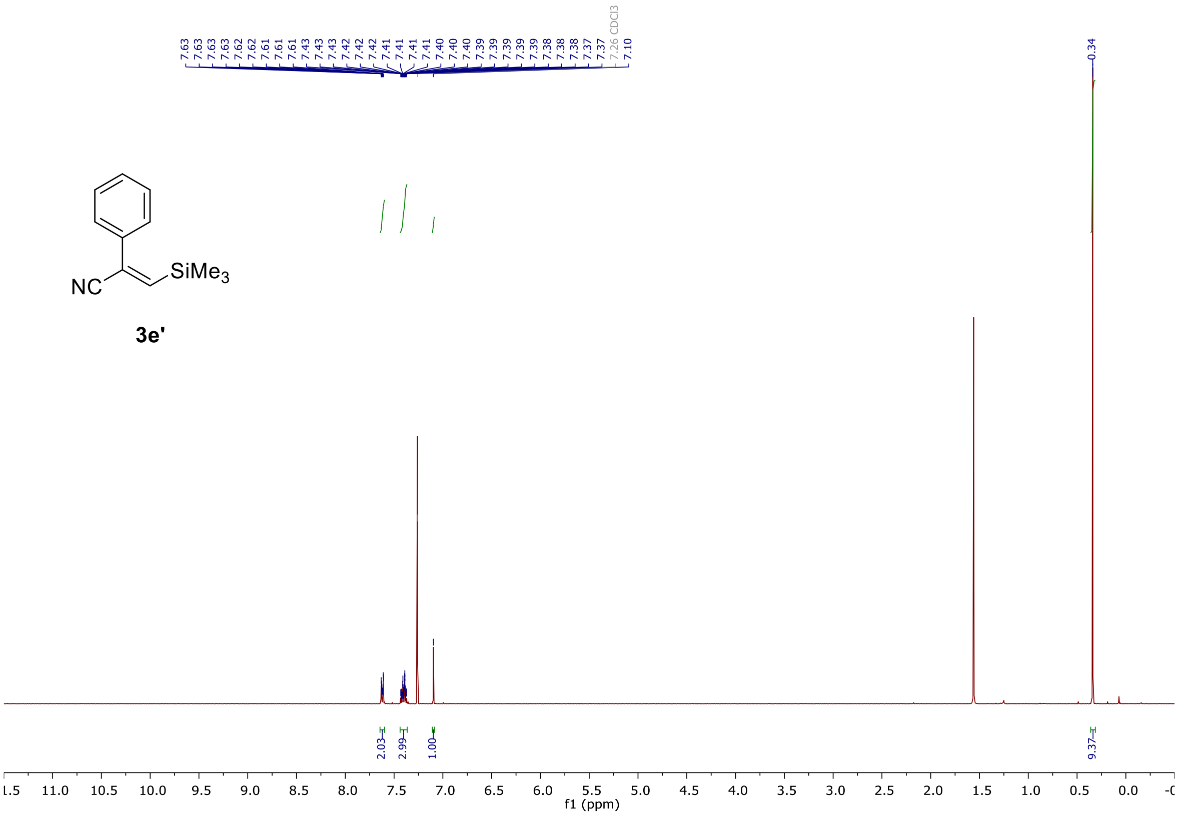

3e'

Figure S69 ^{1}H NMR spectrum of **3e’** (400 MHz, $CDCl_3$).

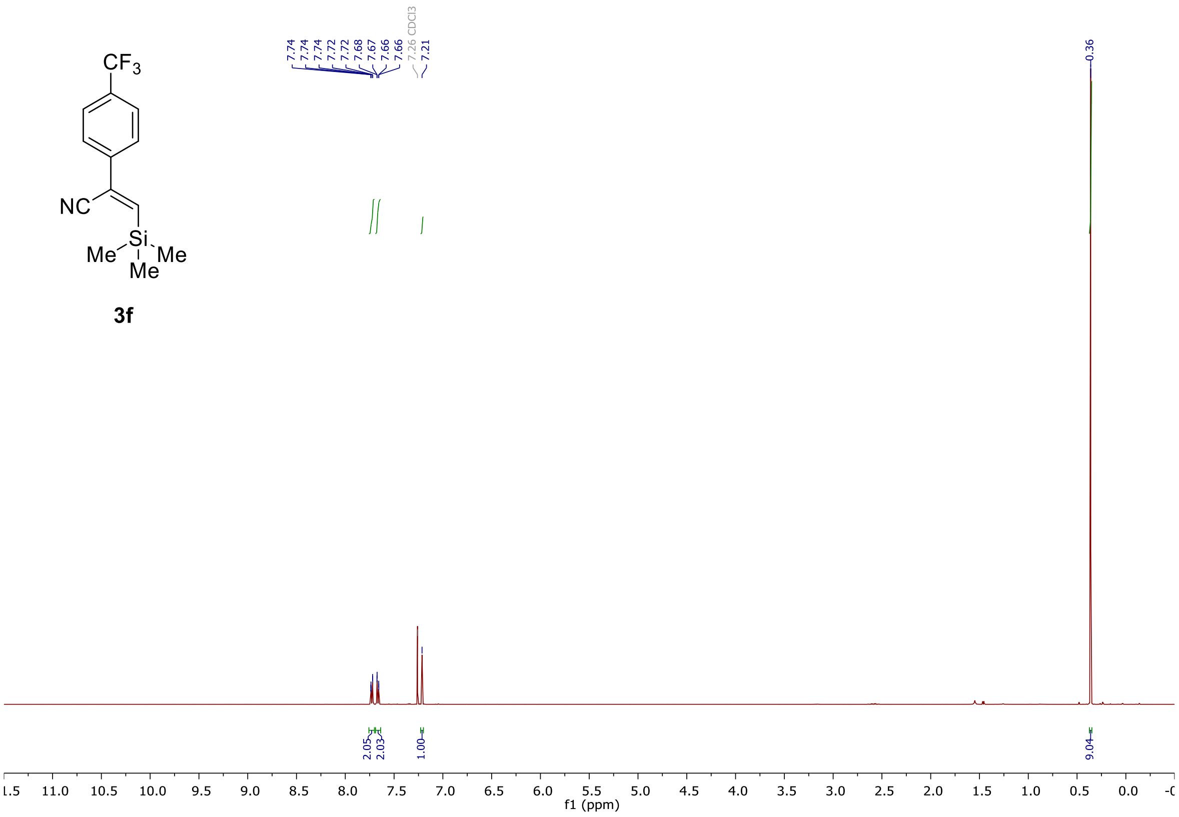

Figure S70 ^{1}H NMR spectrum of **3f** (500 MHz, $CDCl_3$).

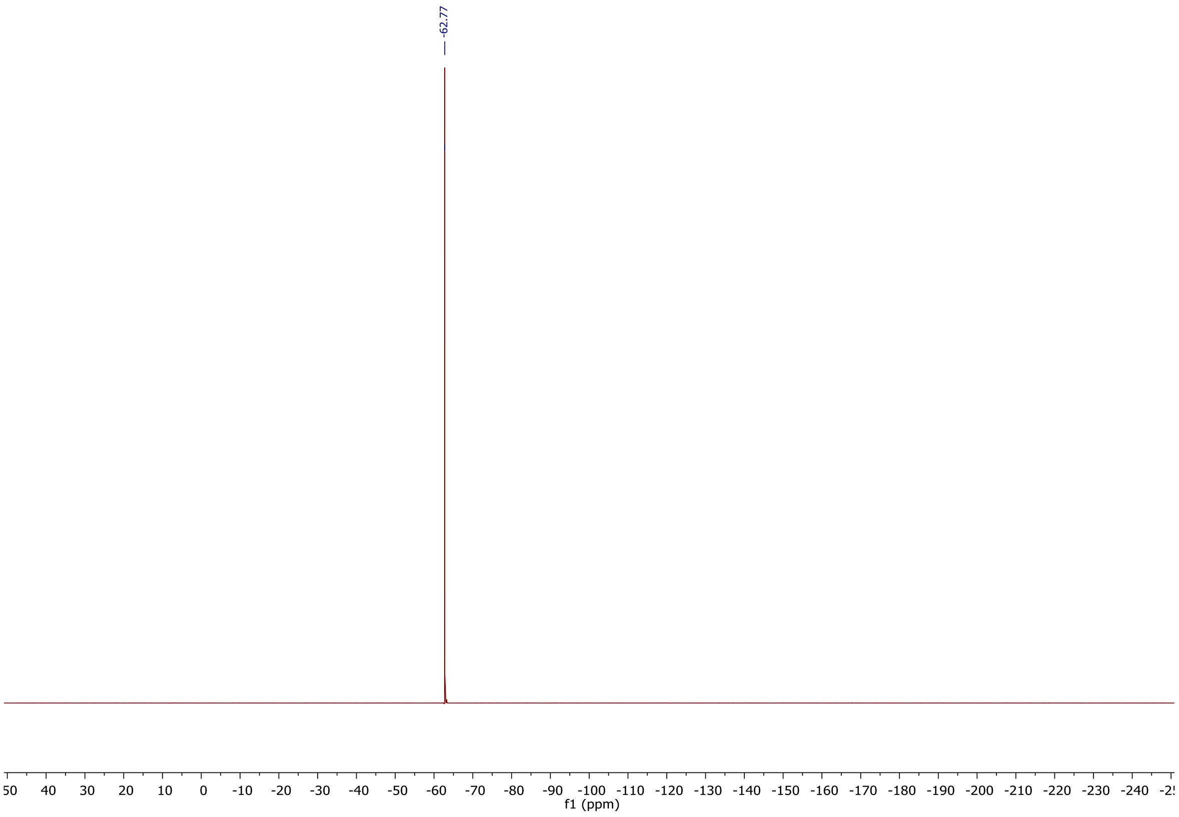

Figure S71 ^{19}F NMR spectrum of **3f** (376 MHz, $CDCl_3$).

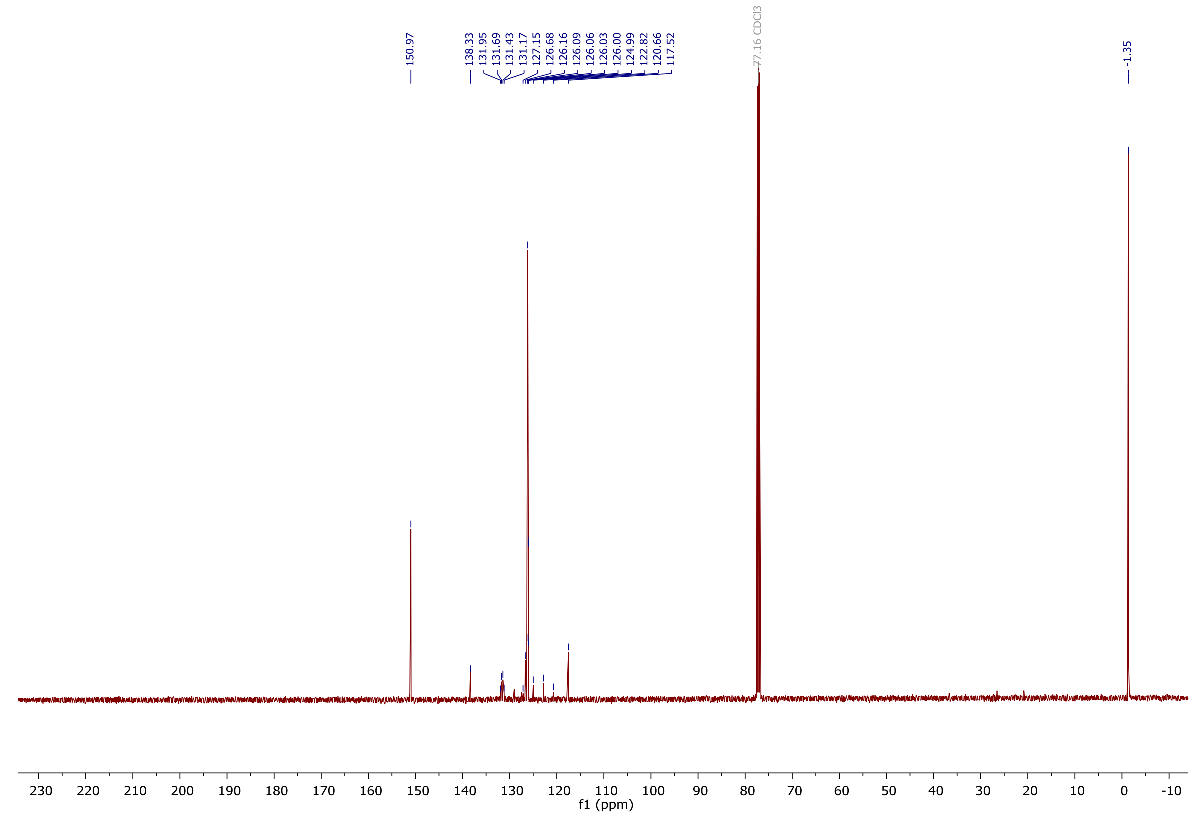

Figure S72 ^{13}C NMR spectrum of **3f** (126 MHz, $CDCl_3$).

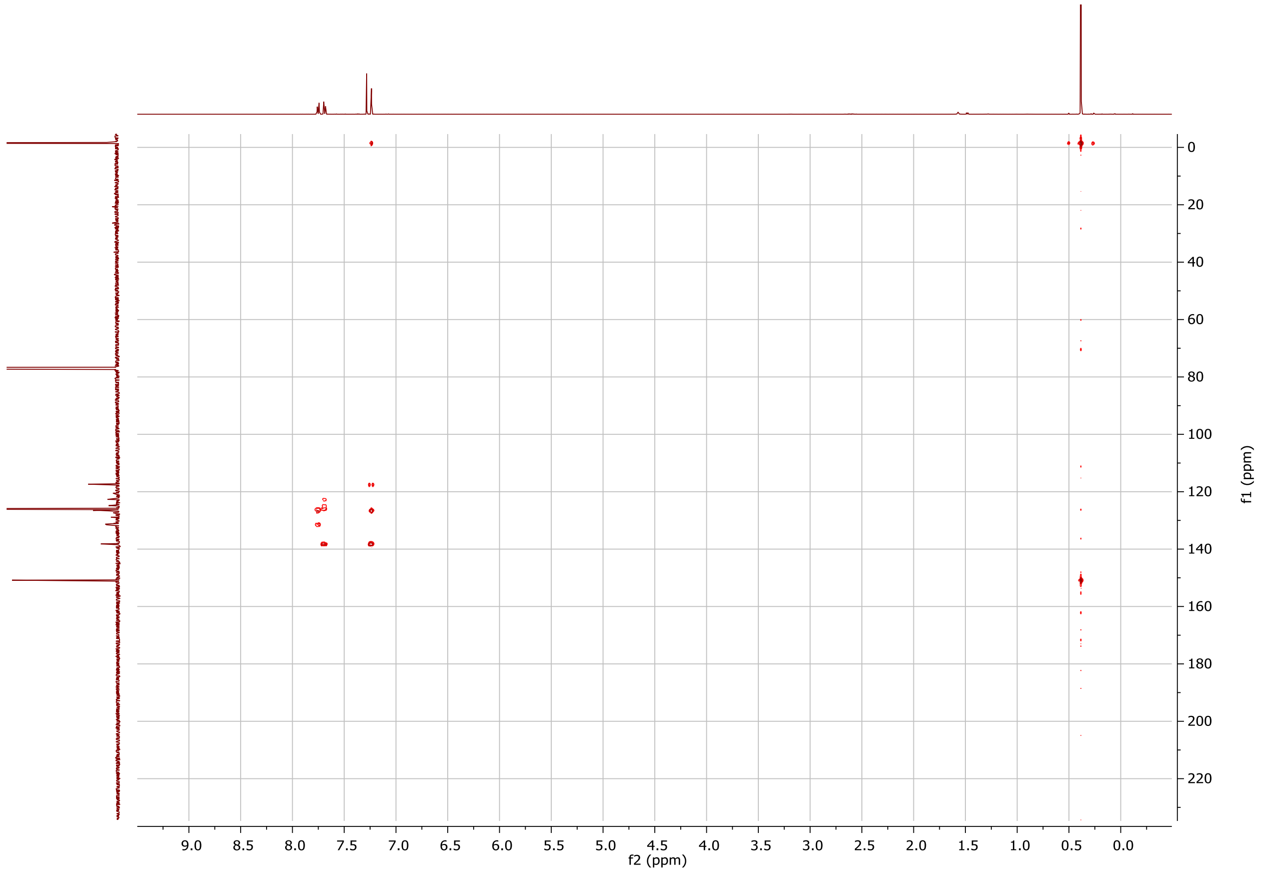

Figure S73 HMBC NMR spectrum of **3f** (500 MHz, $CDCl_3$).

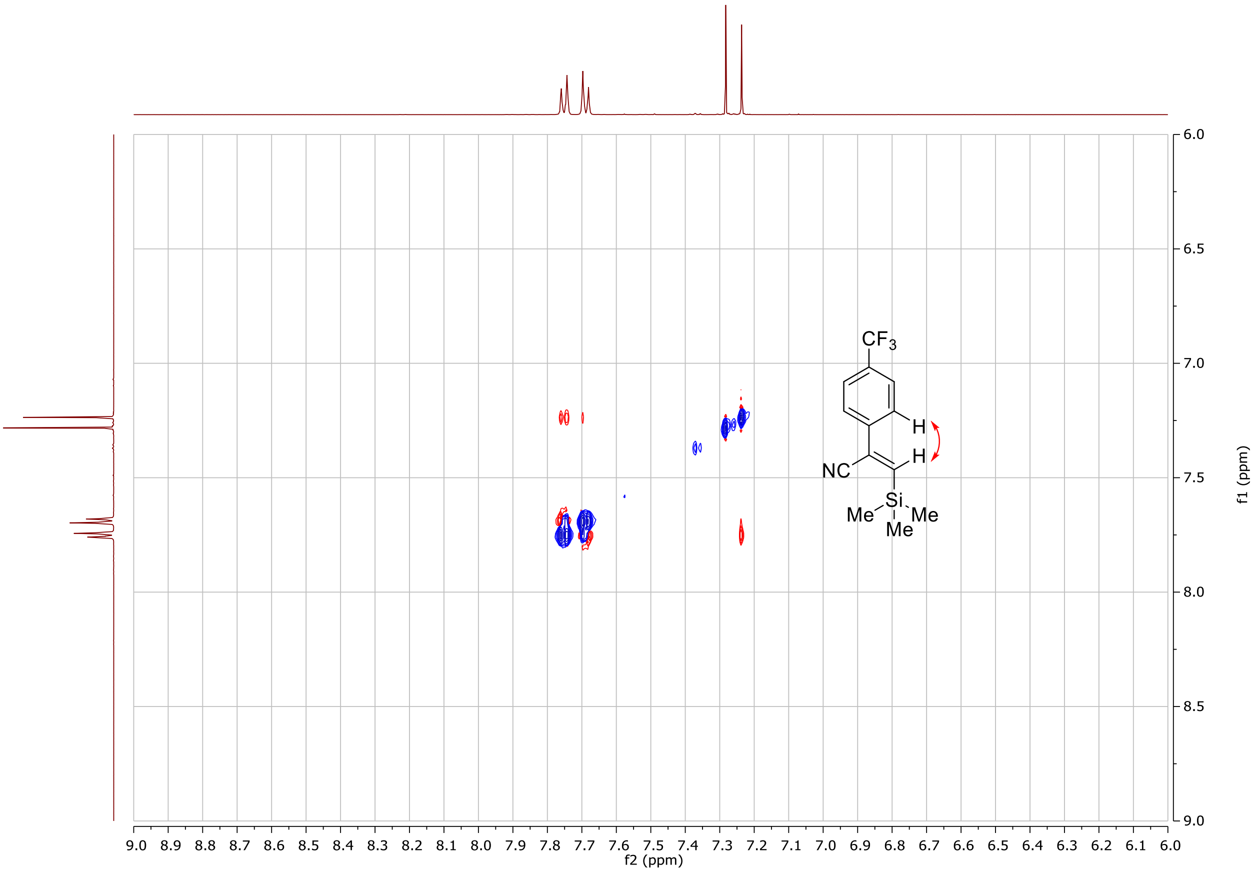

Figure S74 NOESY NMR spectrum of **3f** (500 MHz, $CDCl_3$).

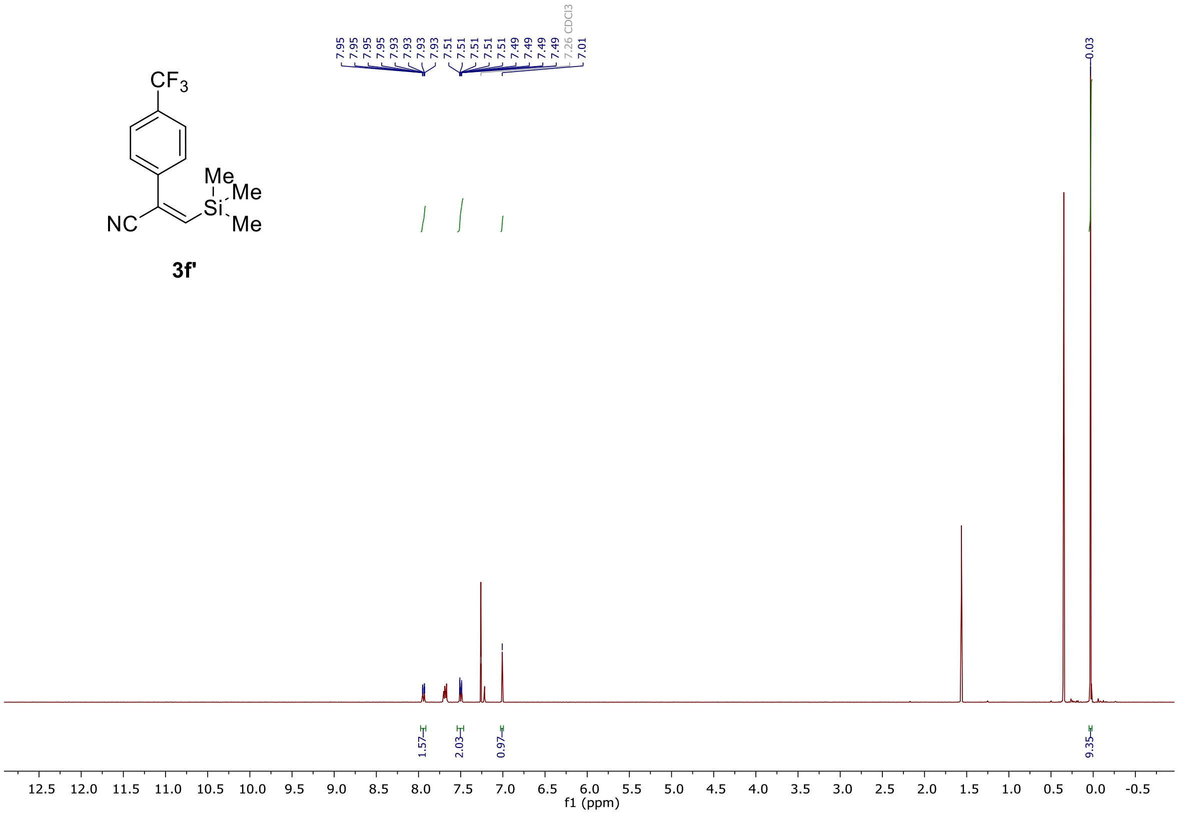

Figure S75 ^{1}H NMR spectrum of **3f'** (400 MHz, $CDCl_3$).

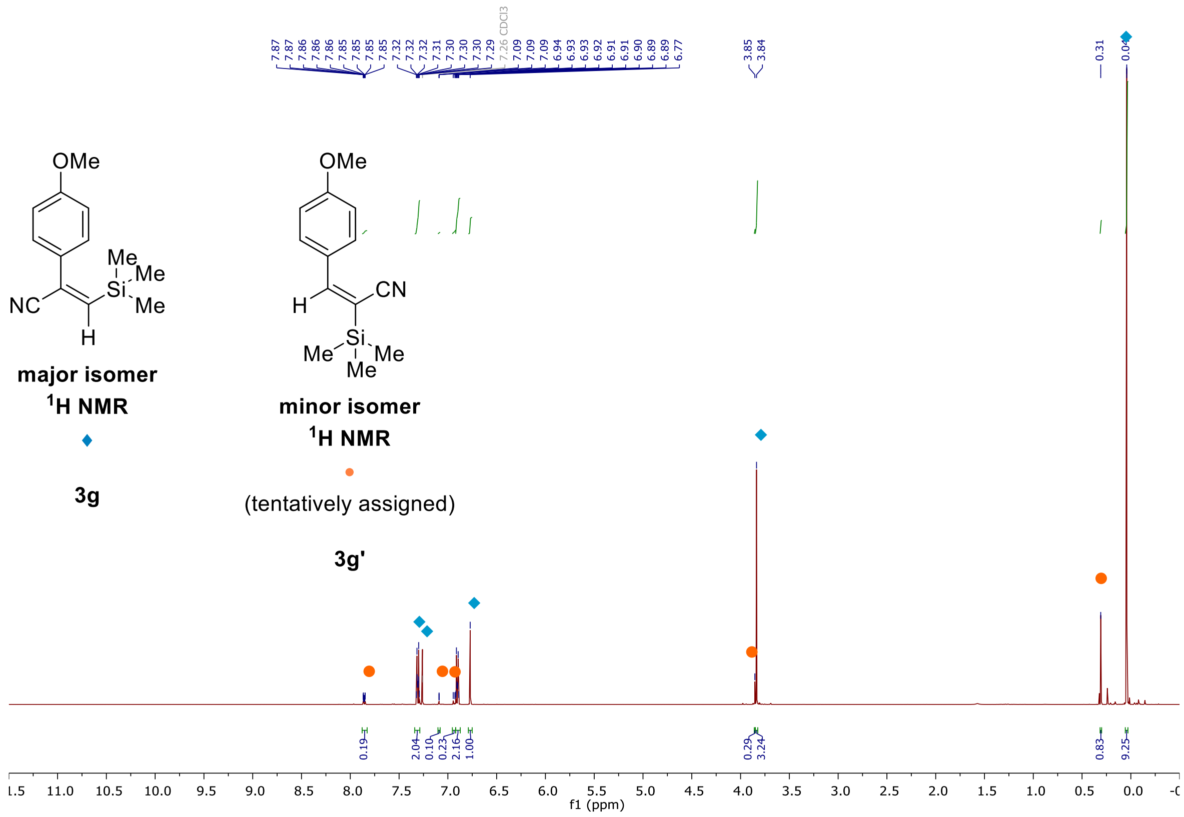

Figure S76 ^{1}H NMR spectrum of **3g** and **3g'** (500 MHz, $CDCl_3$).

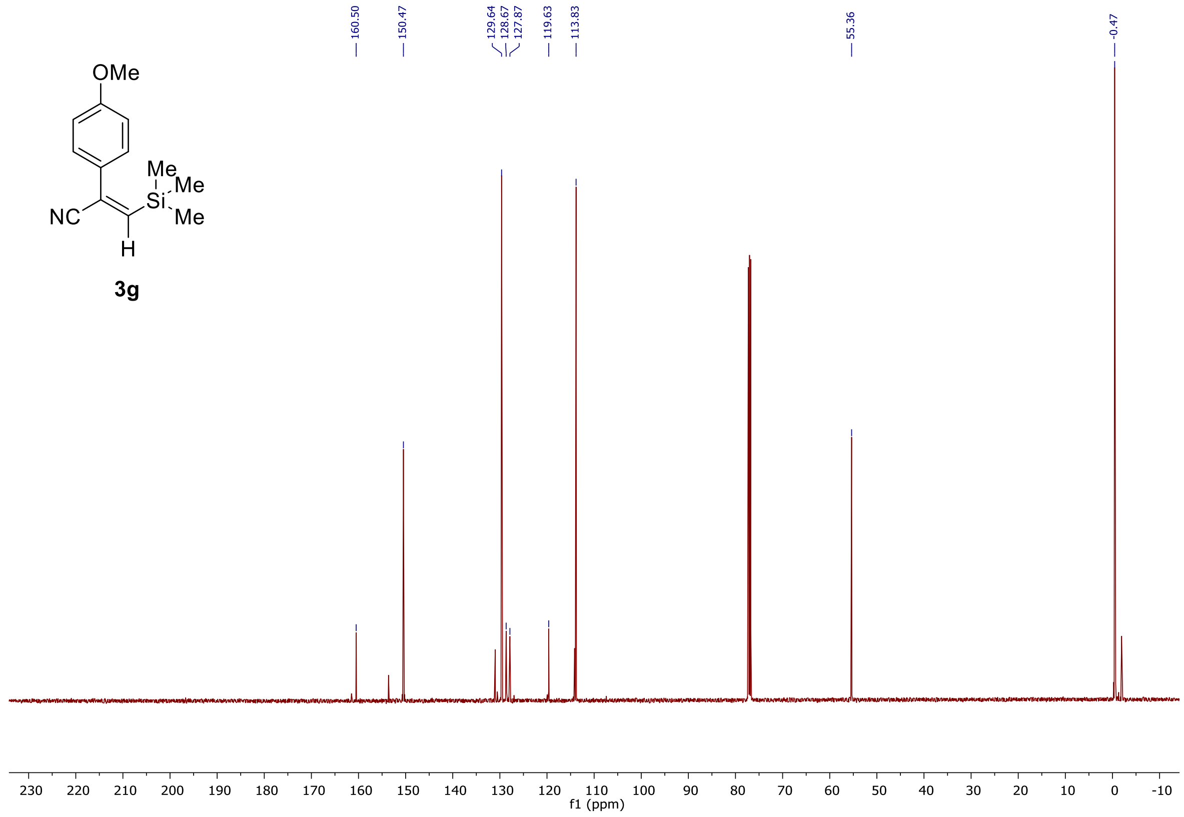

Figure S77 ^{13}C NMR spectrum of **3g** (126 MHz, $CDCl_3$).

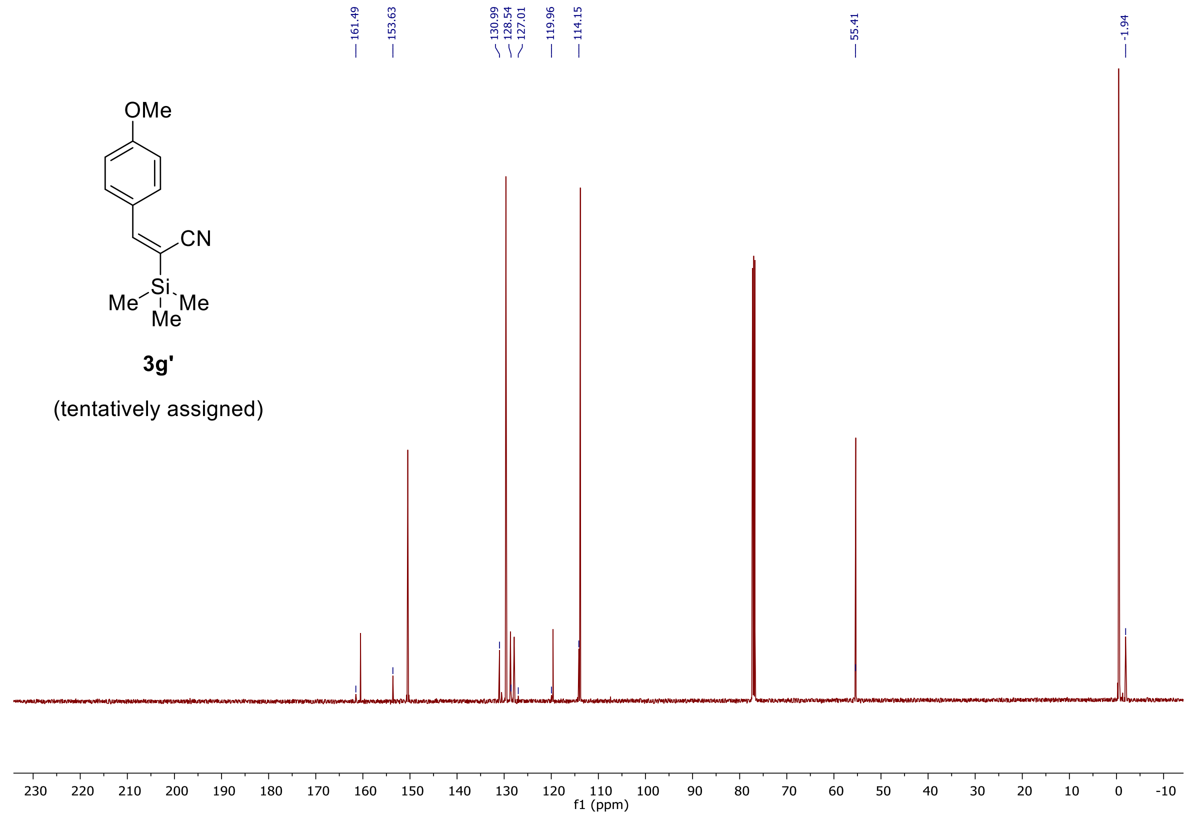

Figure S78 ^{13}C NMR spectrum of **3g’** (126 MHz, $CDCl_3$).

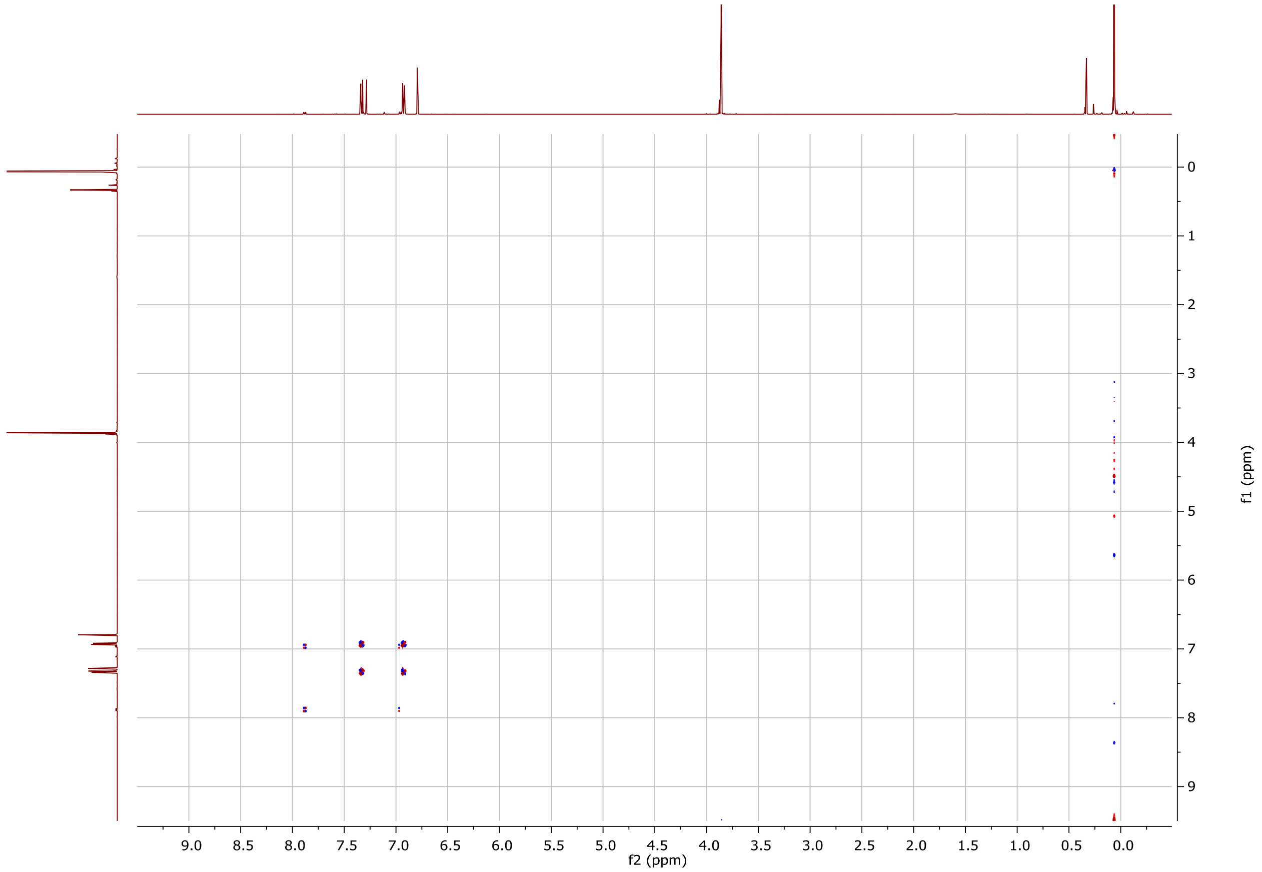

Figure S79 DQF-COSY NMR spectrum of **3g** and **3g’** (500 MHz, $CDCl_3$).

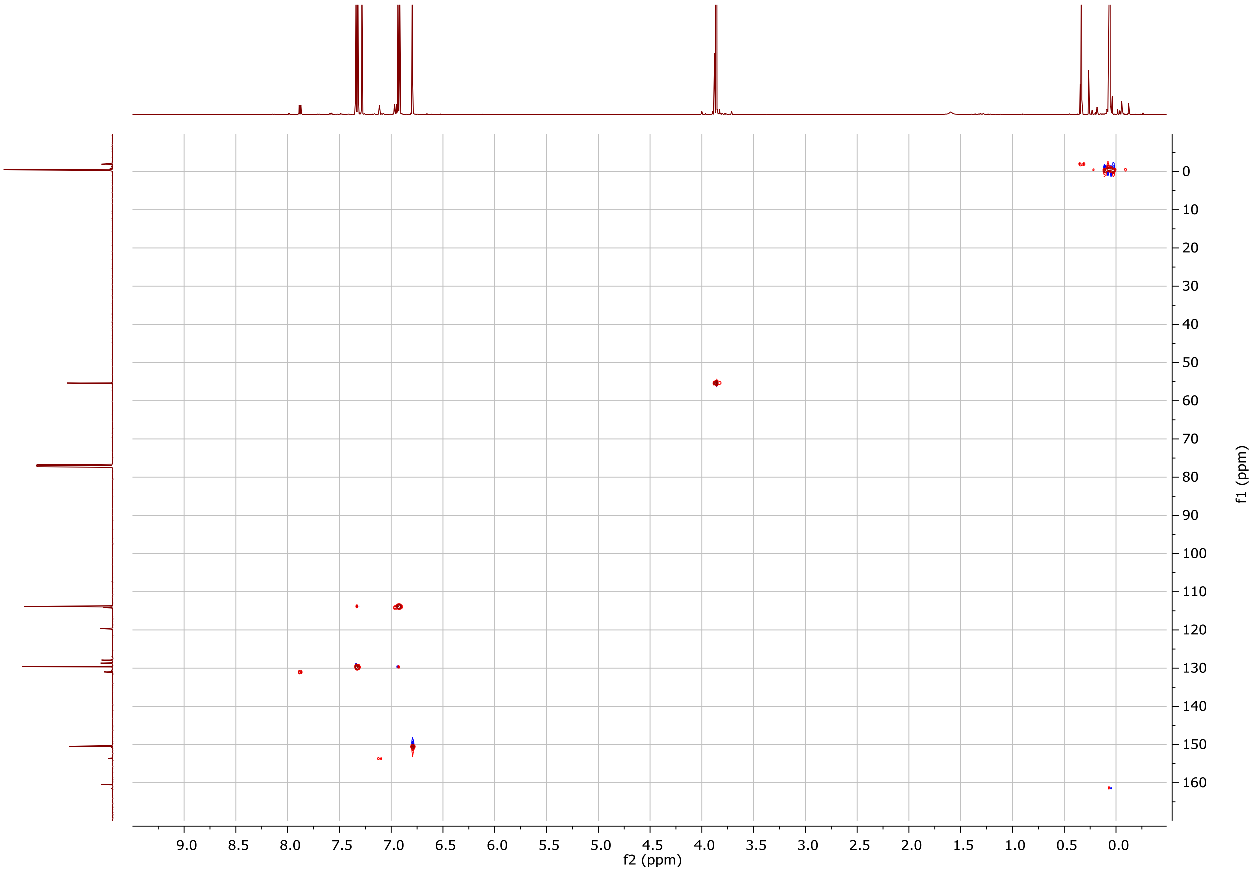

Figure S80 HSQC NMR spectrum of **3g** and **3g’** (500 MHz, $CDCl_3$).

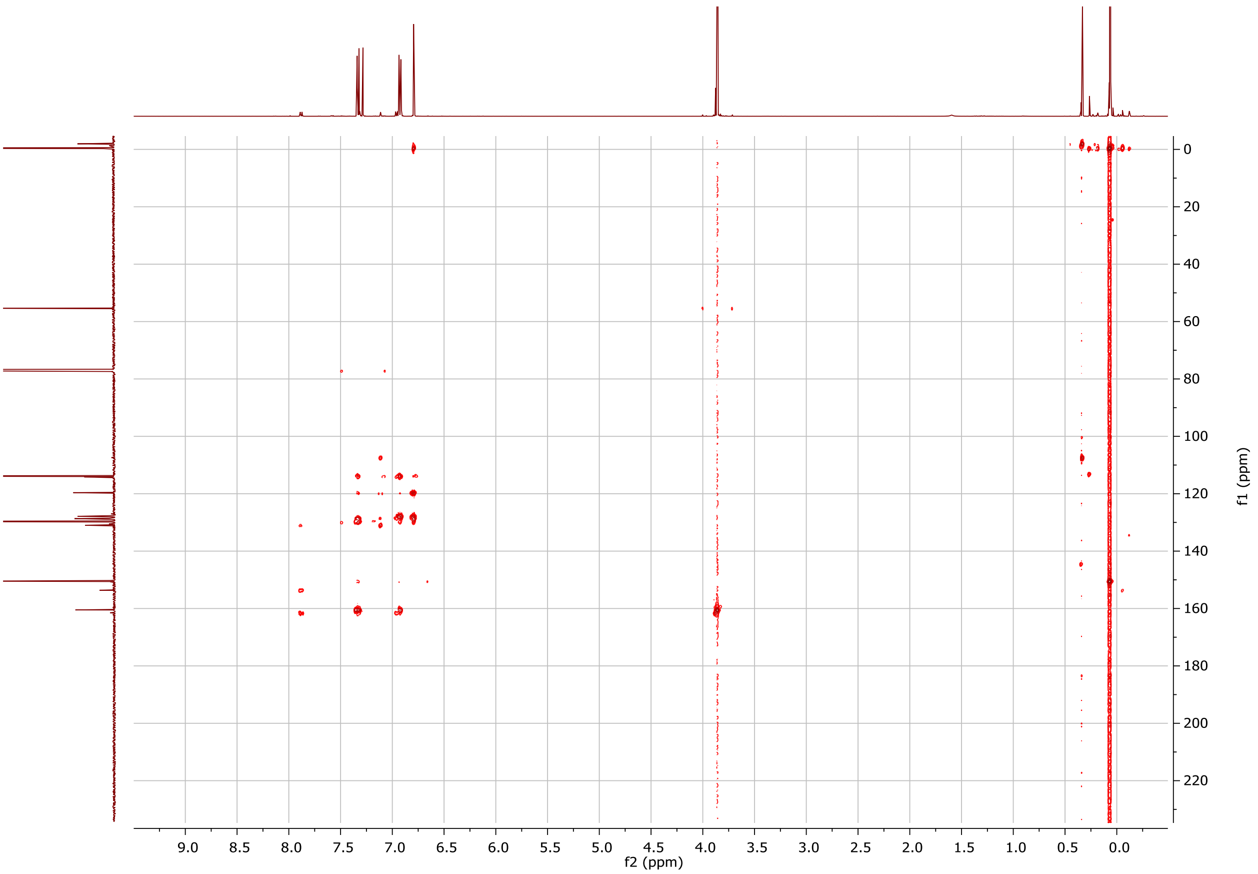

Figure S81 HMBC NMR spectrum of **3g** and **3g’** (500 MHz, $CDCl_3$).

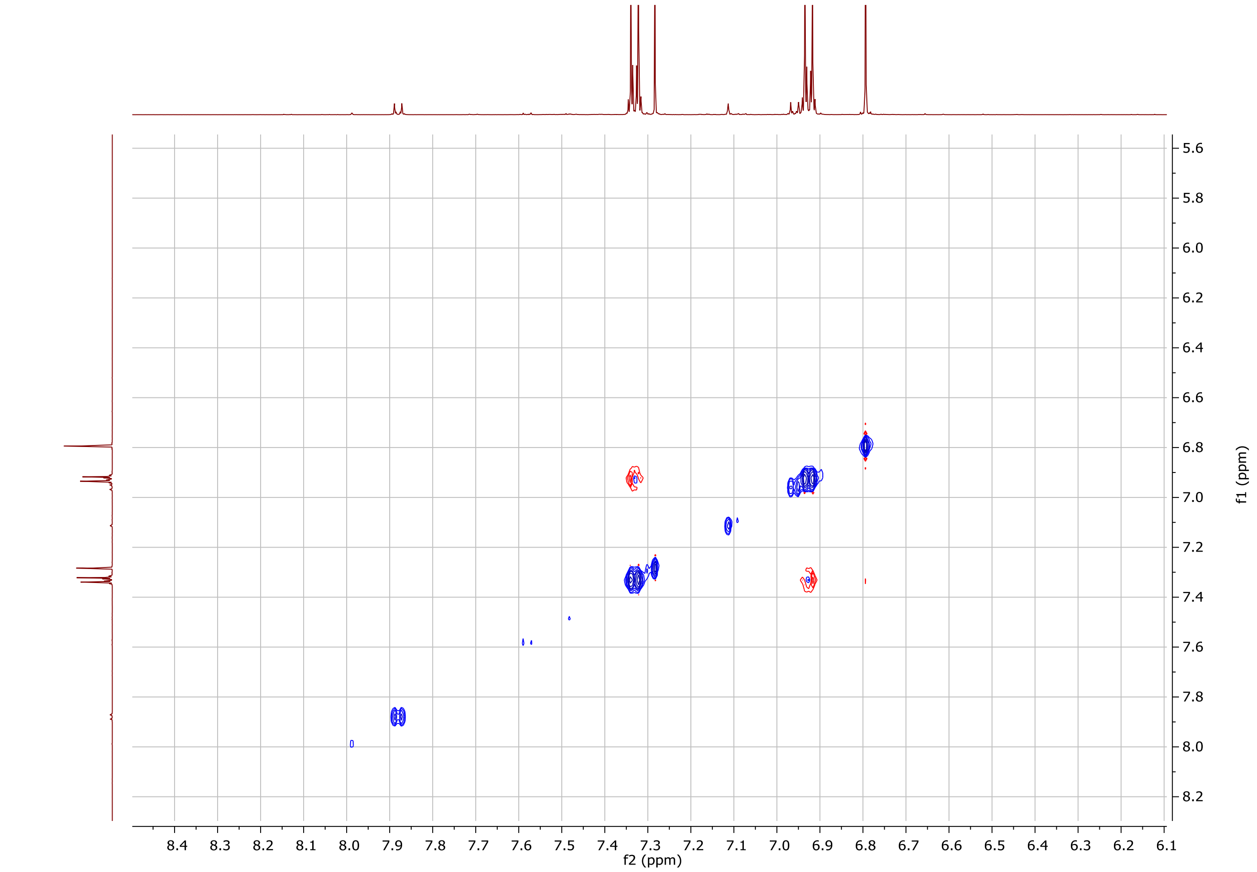

Figure S82 NOESY NMR spectrum of **3g** (500 MHz, $CDCl_3$).

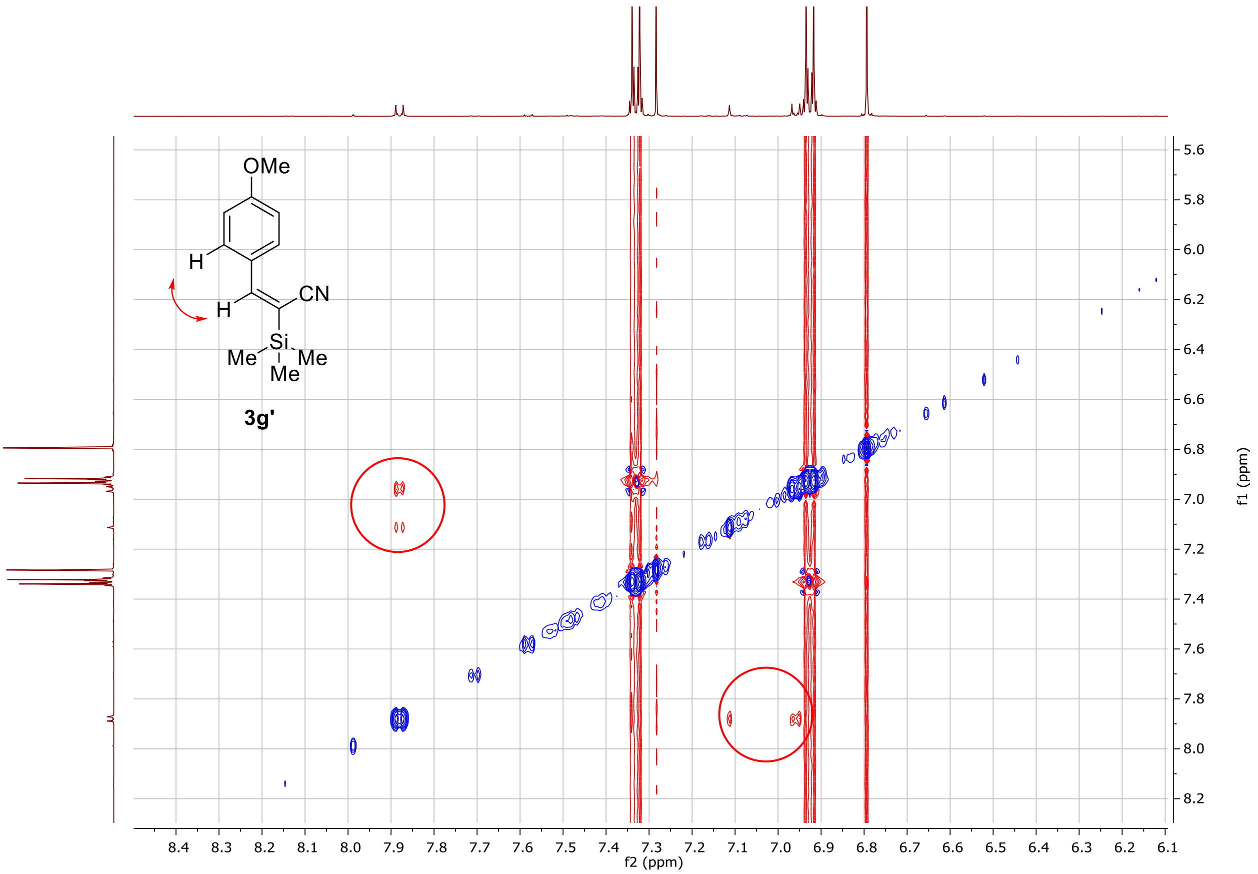

Figure S83 NOESY NMR spectrum of **3g'** (500 MHz, $CDCl_3$).

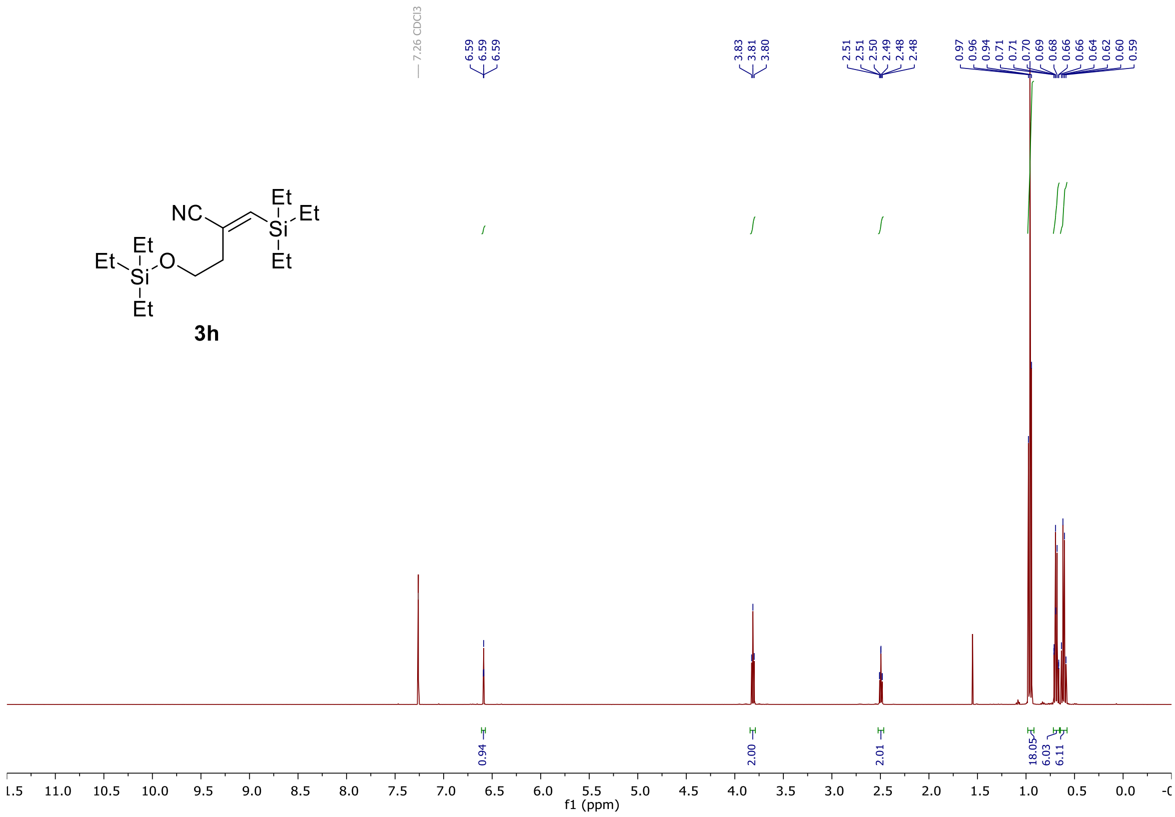

Figure S84 ^{1}H NMR spectrum of **3h** (500 MHz, $CDCl_3$).

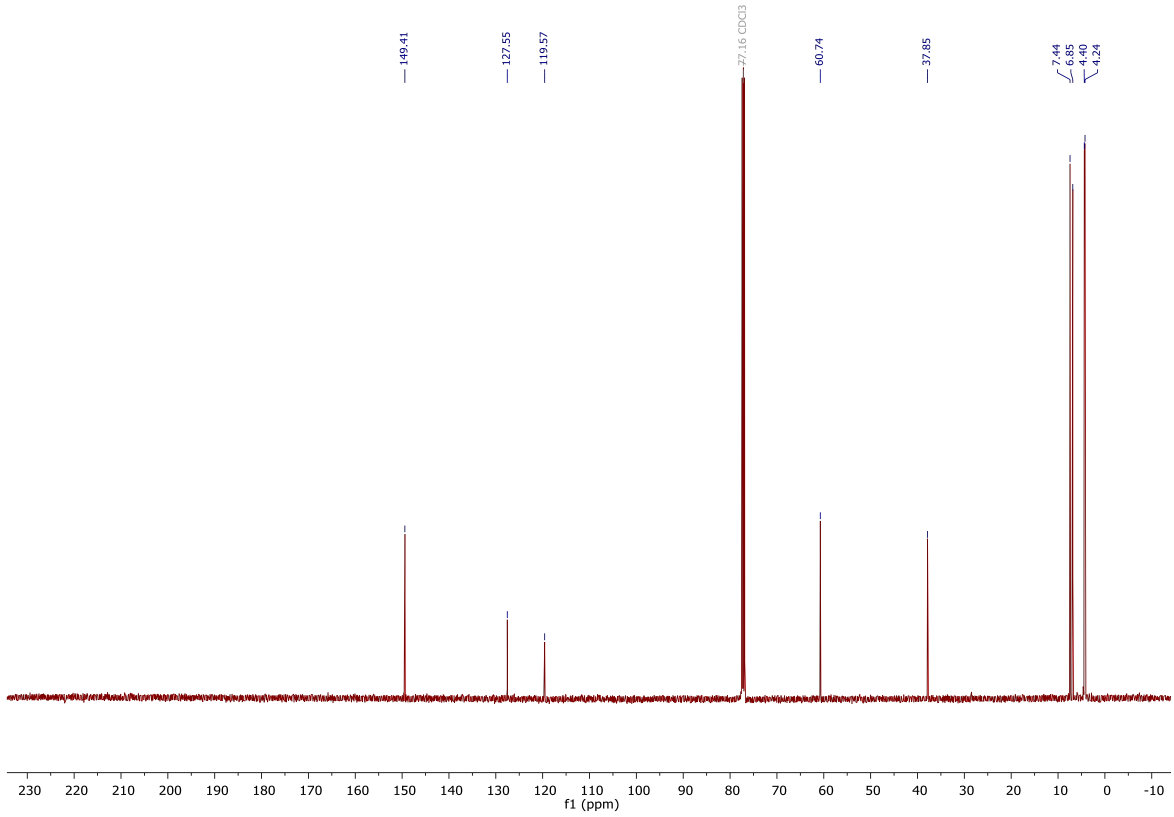

Figure S85 ^{13}C NMR spectrum of **3h** (126 MHz, $CDCl_3$).

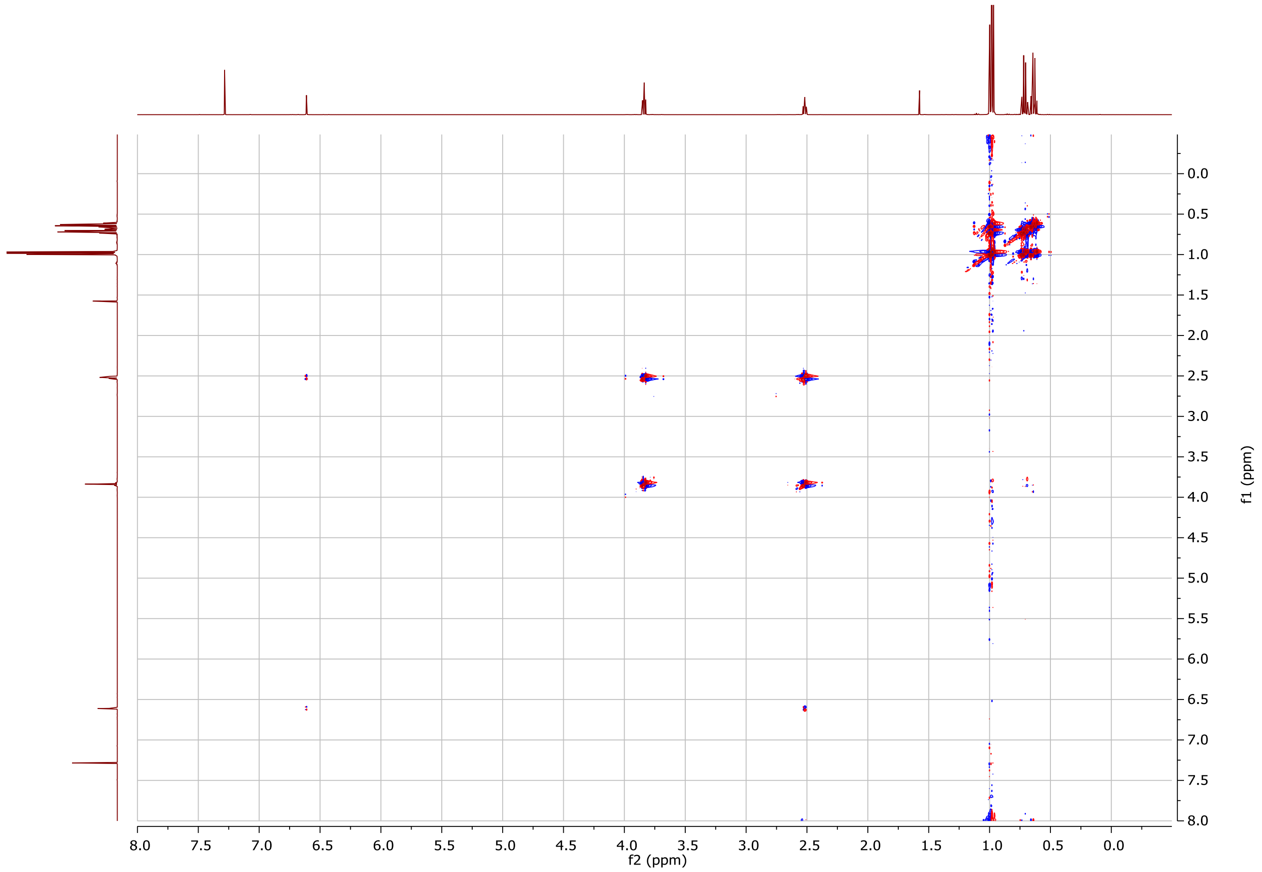

Figure S86 DQF-COSY NMR spectrum of **3h** (500 MHz, $CDCl_3$).

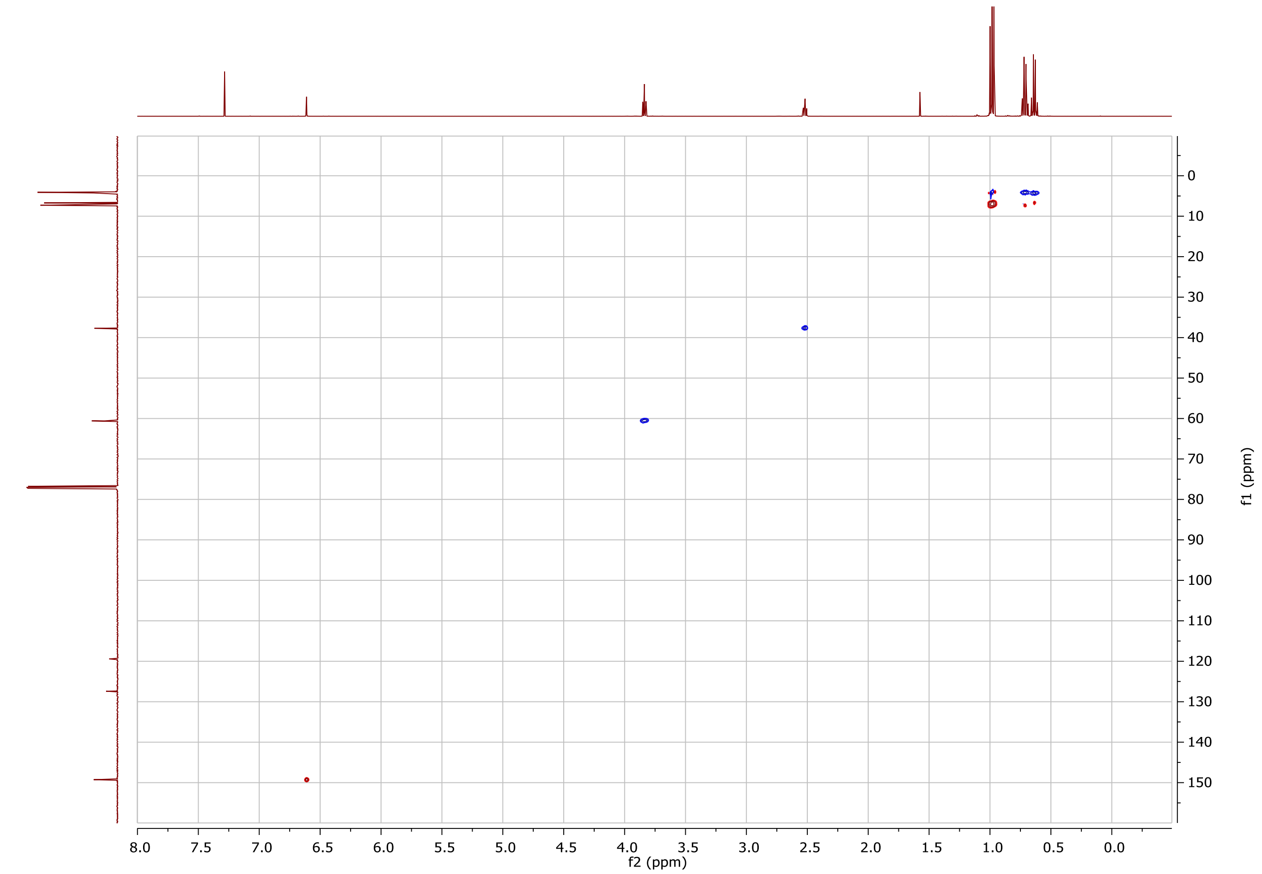

Figure S87 HSQC NMR spectrum of **3h** (500 MHz, $CDCl_3$).

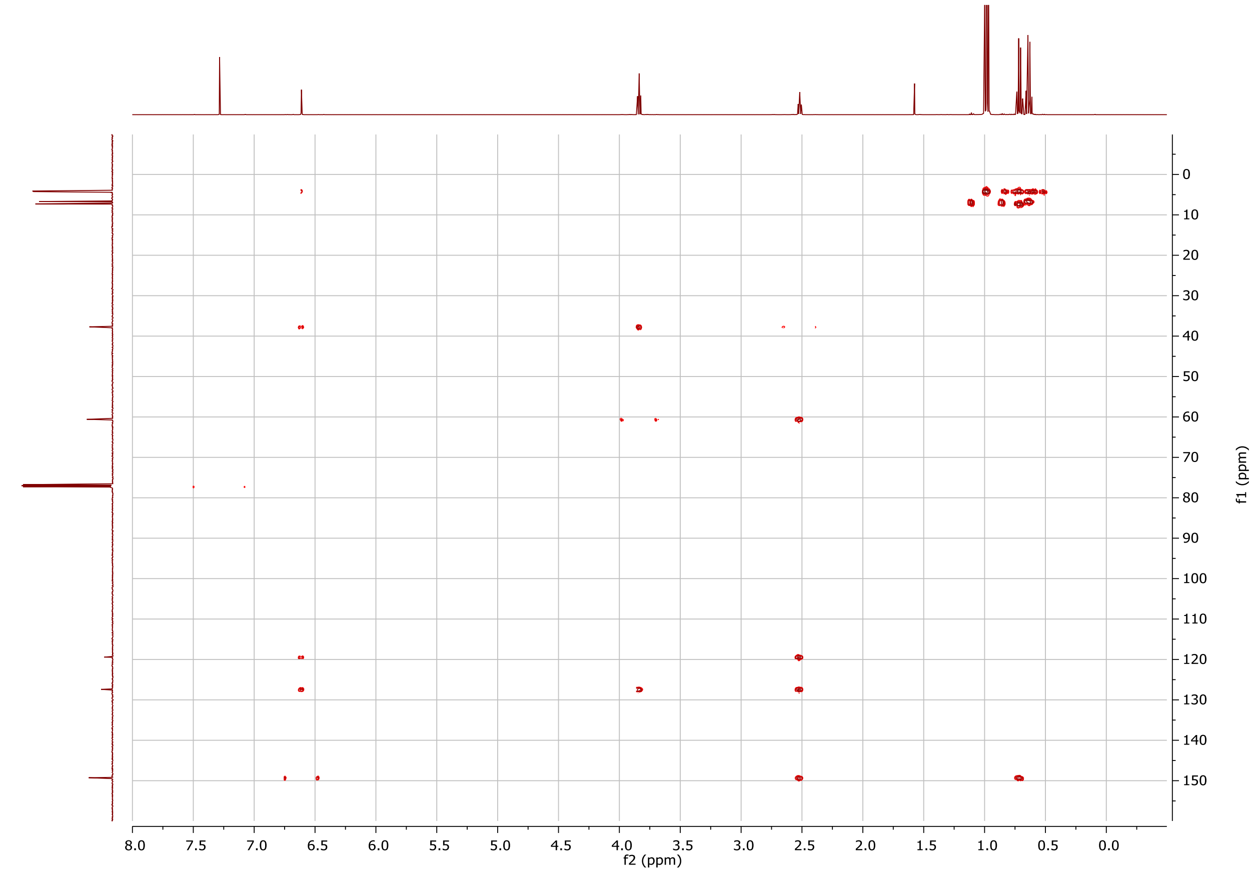

Figure S88 HMBC NMR spectrum of **3h** (500 MHz, $CDCl_3$).

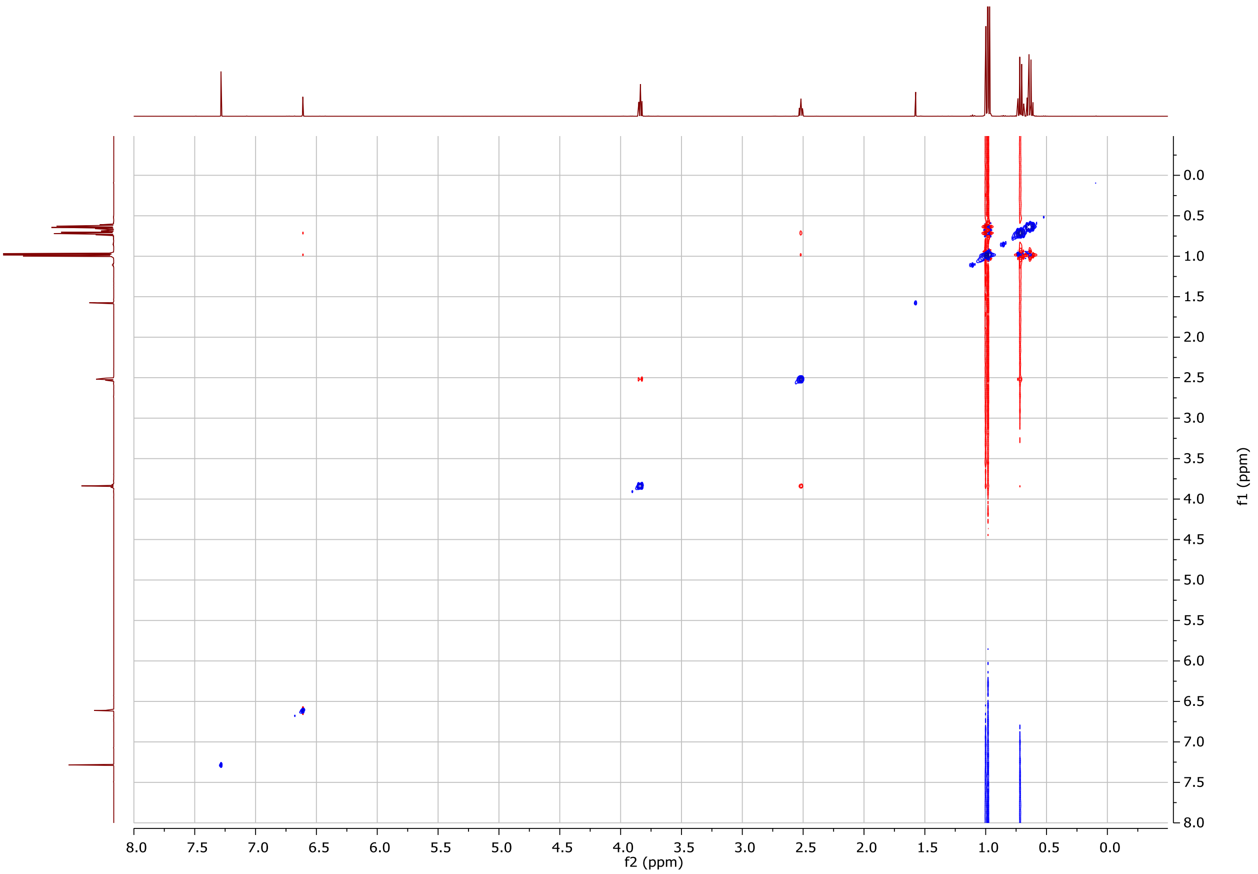

Figure S89 NOESY NMR spectrum of **3h** (500 MHz, $CDCl_3$).

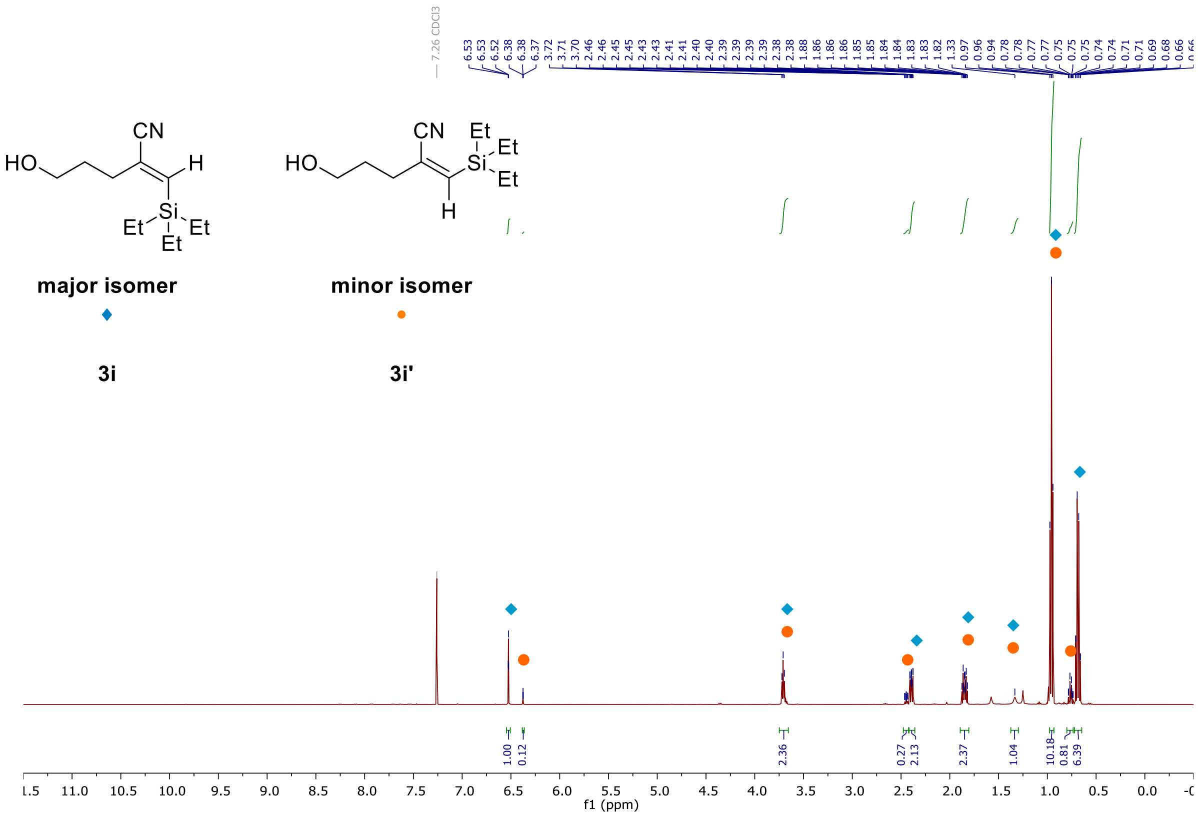

Figure S90 ^{1}H NMR spectrum of **3i** and **3i'** (500 MHz, $CDCl_3$).

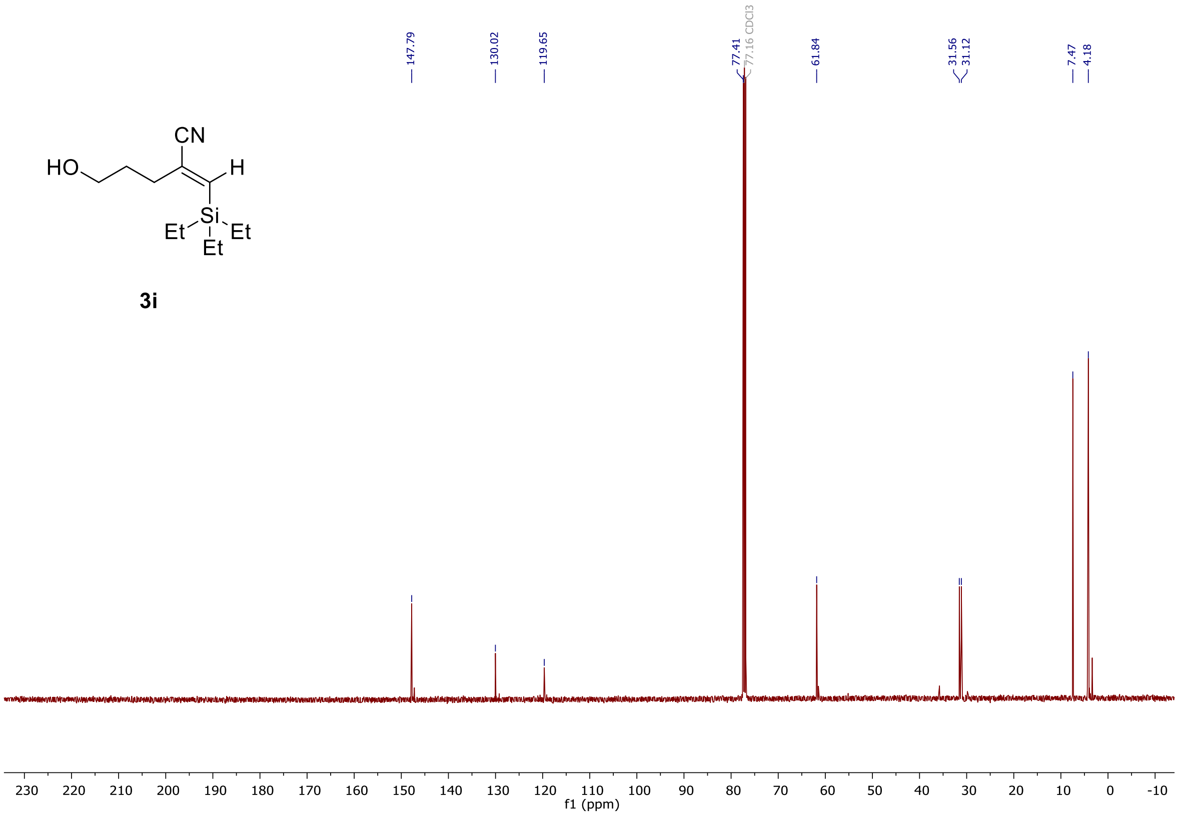

Figure S91 ^{13}C NMR spectrum of **3i** (126 MHz, $CDCl_3$).

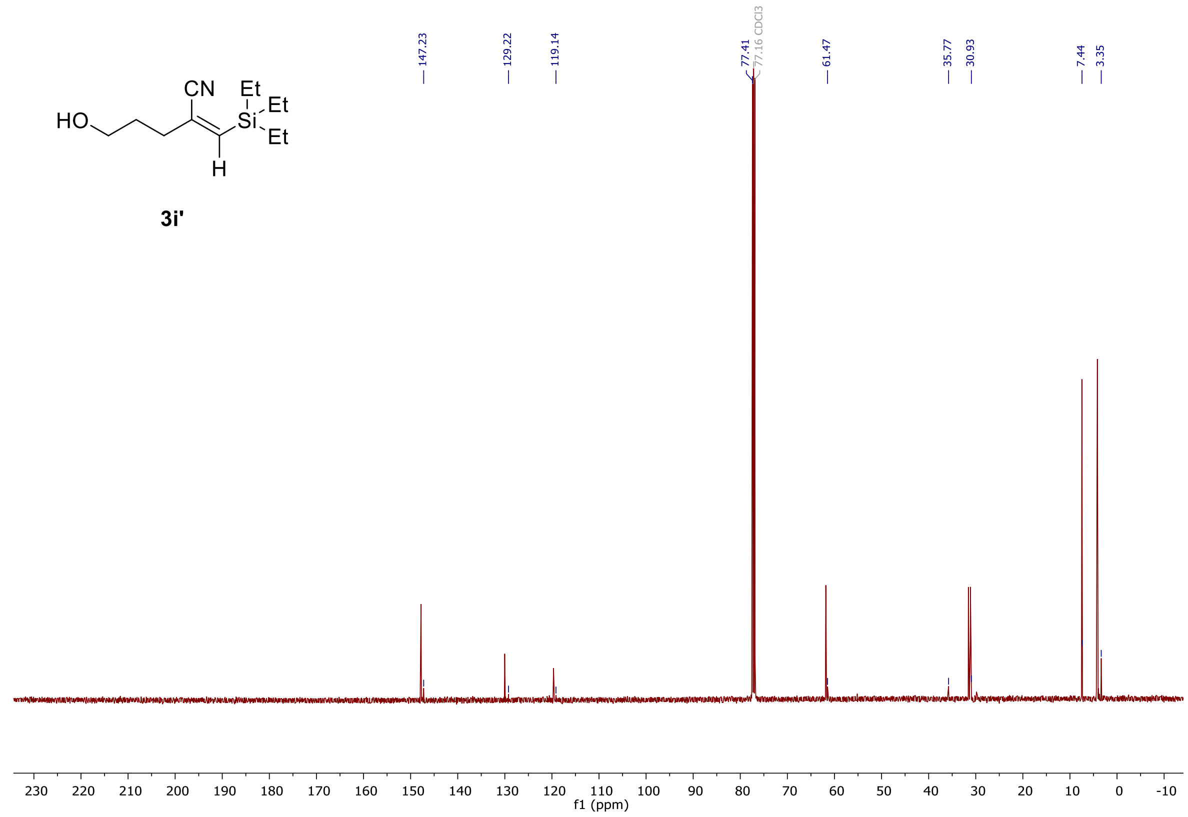

Figure S92 ^{13}C NMR spectrum of **3i'** (126 MHz, $CDCl_3$).

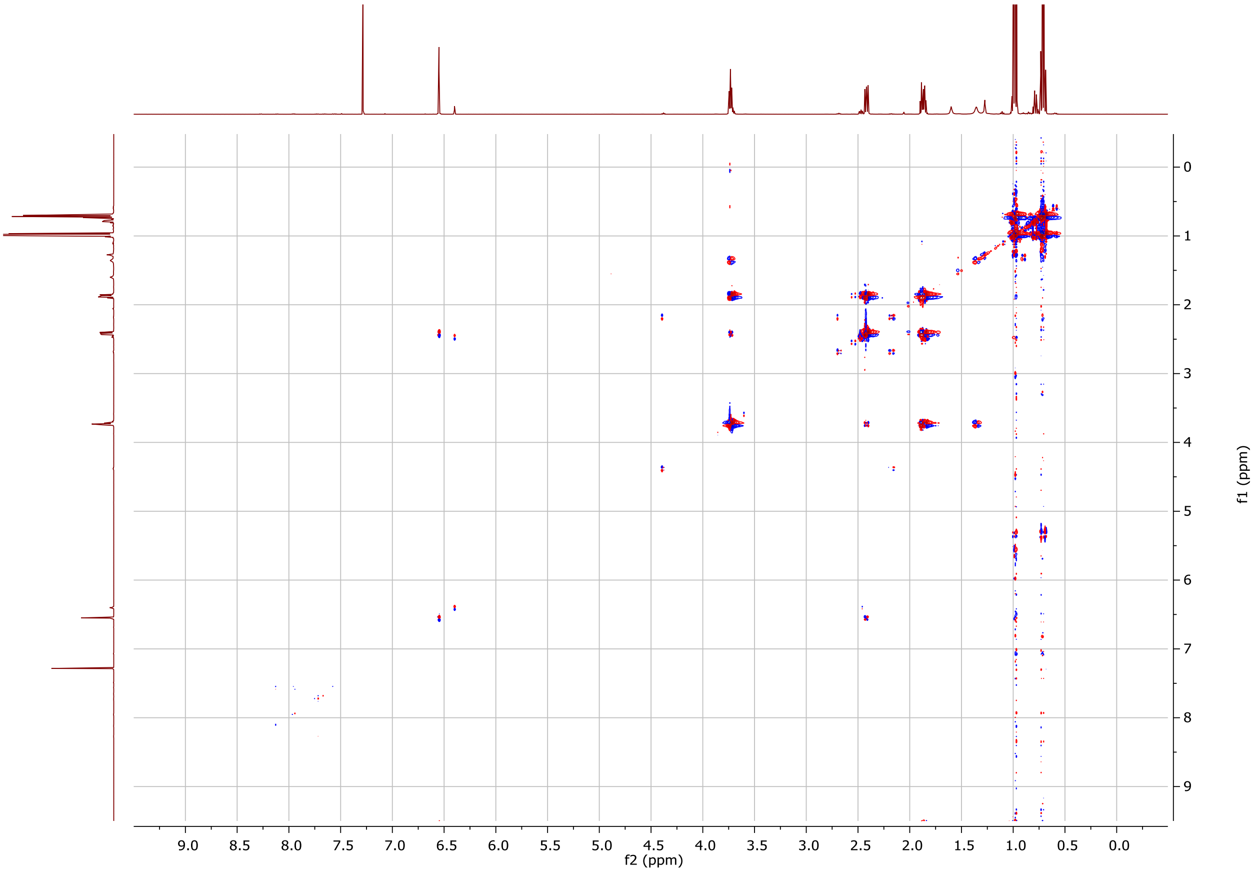

Figure S93 DQF-COSY NMR spectrum of **3i** and **3i'** (500 MHz, $CDCl_3$).

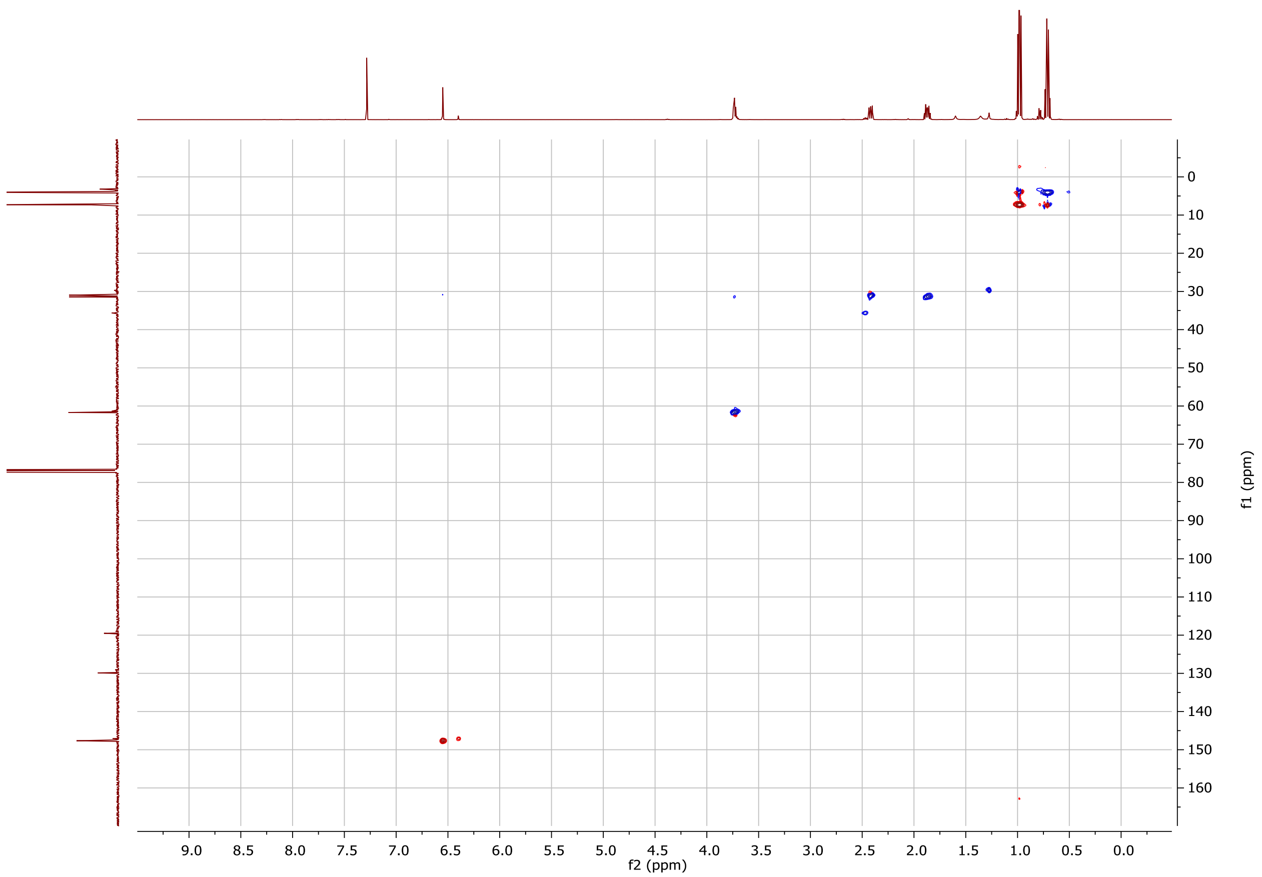

Figure S94 HSQC NMR spectrum of **3i** and **3i’** (500 MHz, $CDCl_3$).

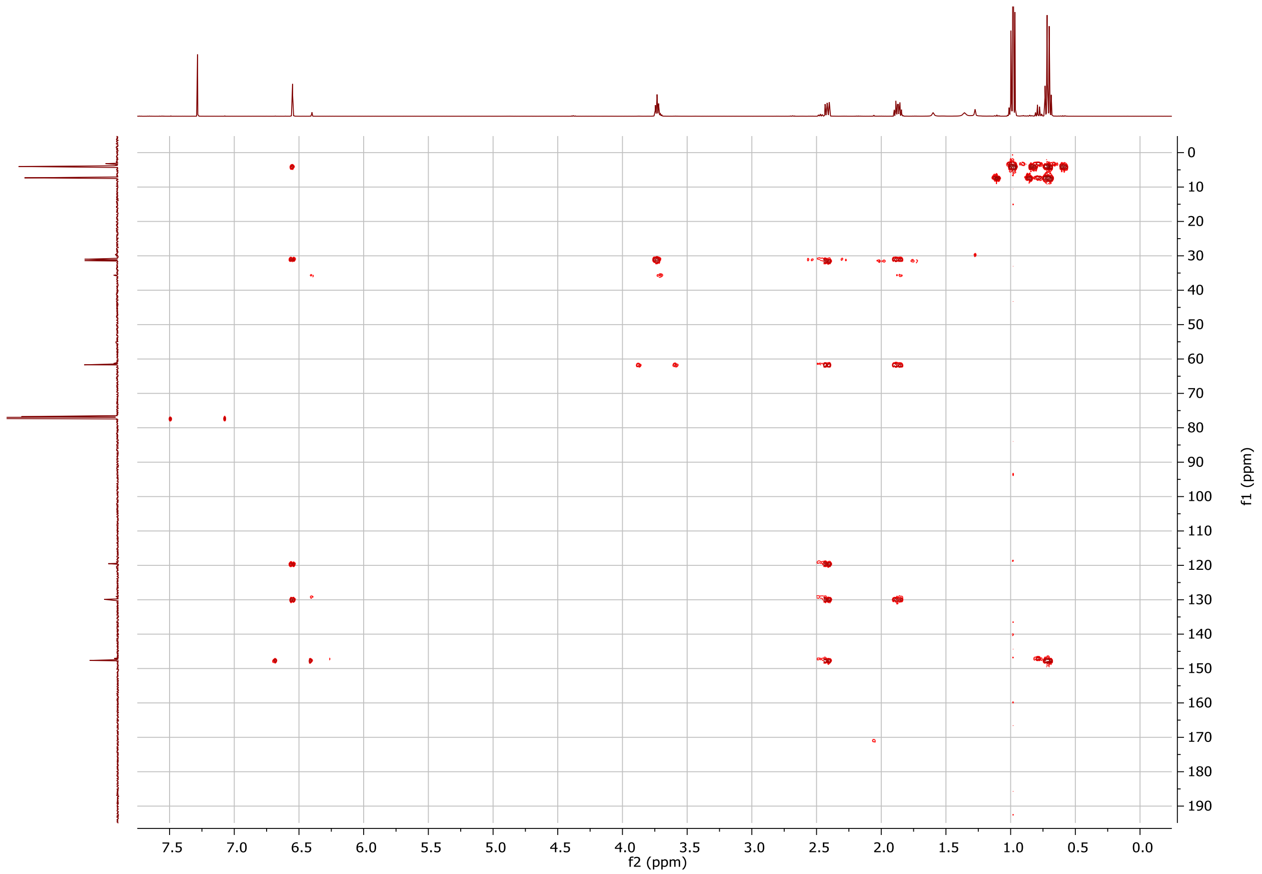

Figure S95 HMBC NMR spectrum of **3i** and **3i’** (500 MHz, $CDCl_3$).

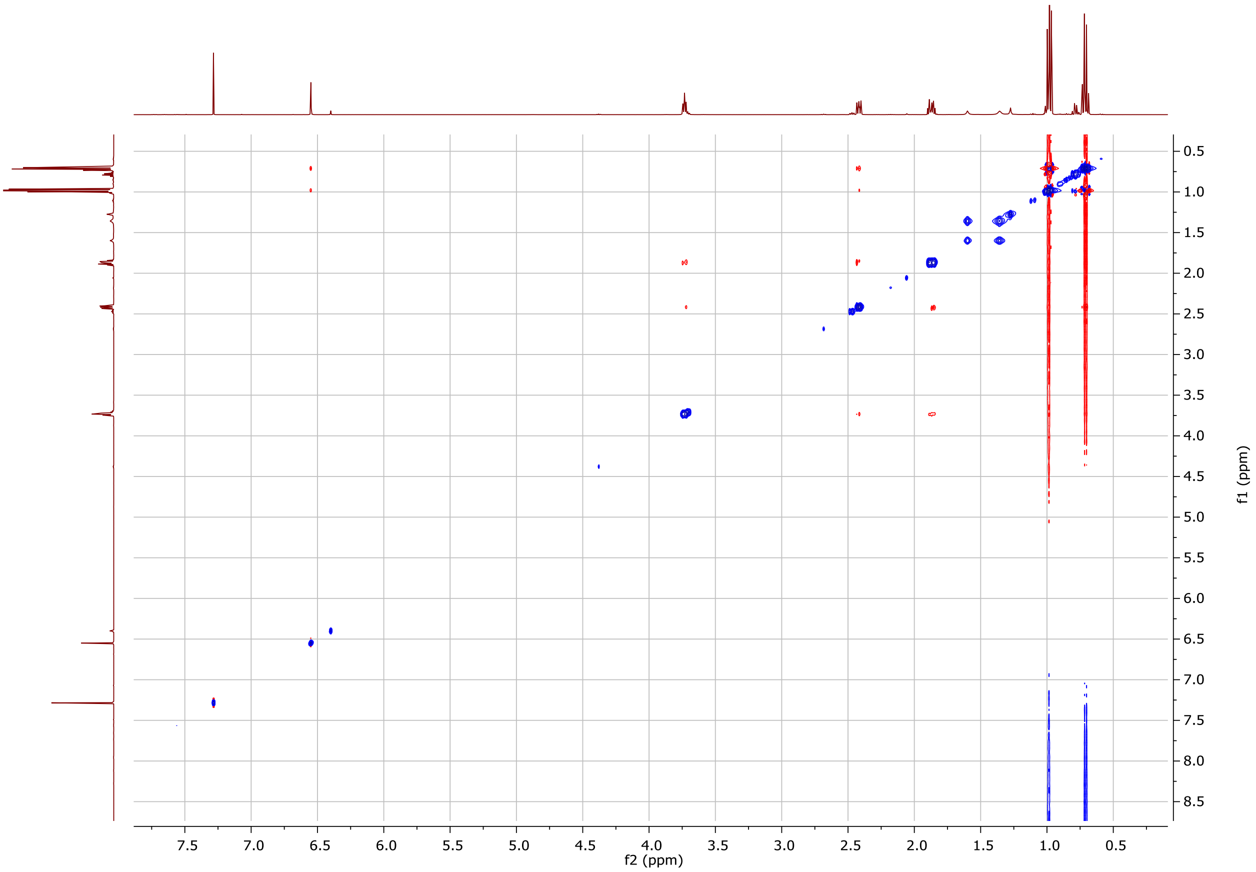

Figure S96 NOESY NMR spectrum of **3i** (500 MHz, $CDCl_3$).

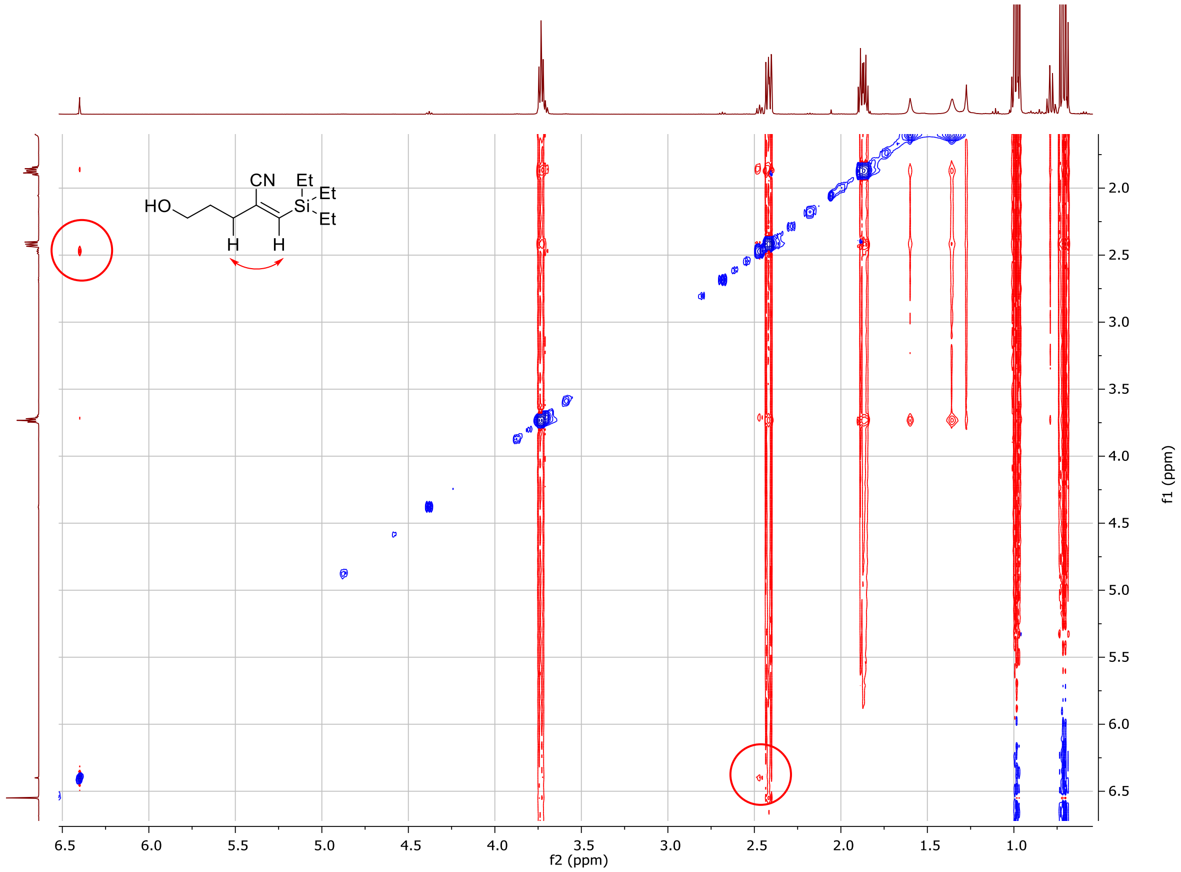

Figure S97 NOESY NMR spectrum of **3i’** (500 MHz, $CDCl_3$).

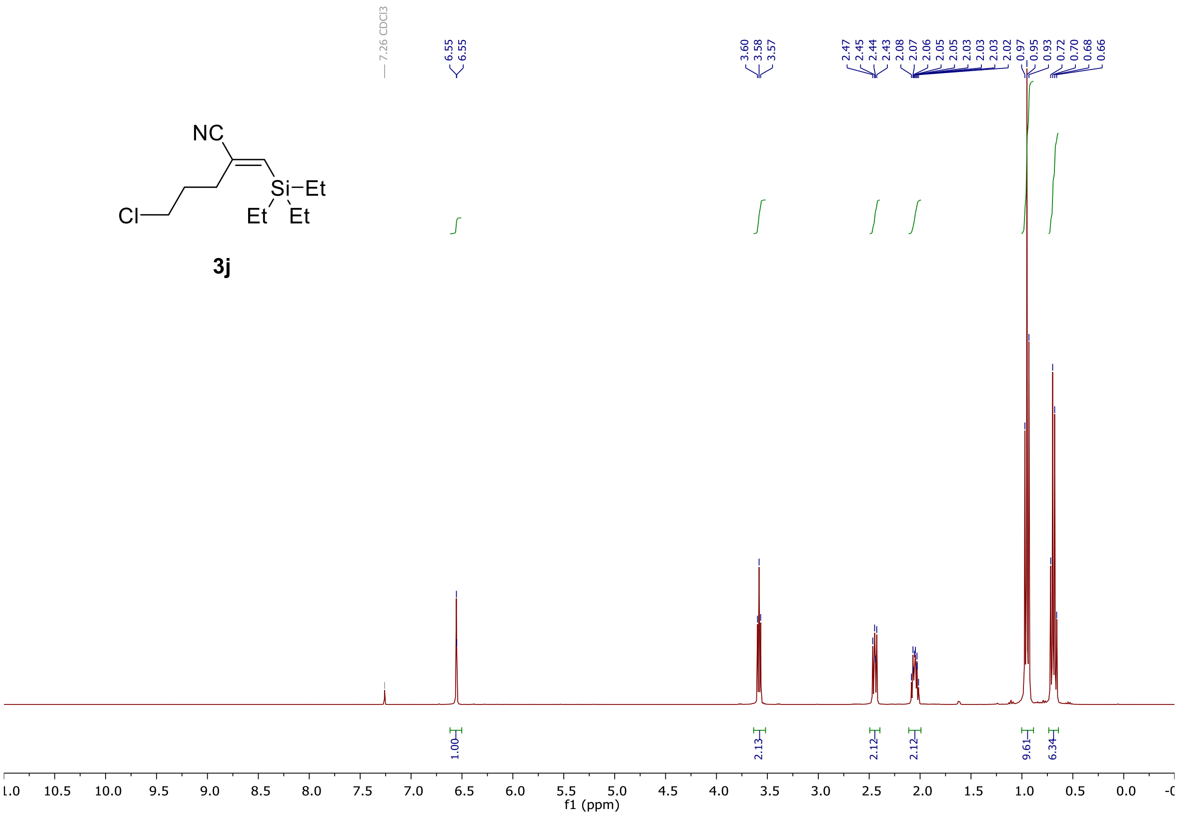

Figure S98 ^{1}H NMR spectrum of **3j** (400 MHz, $CDCl_3$).

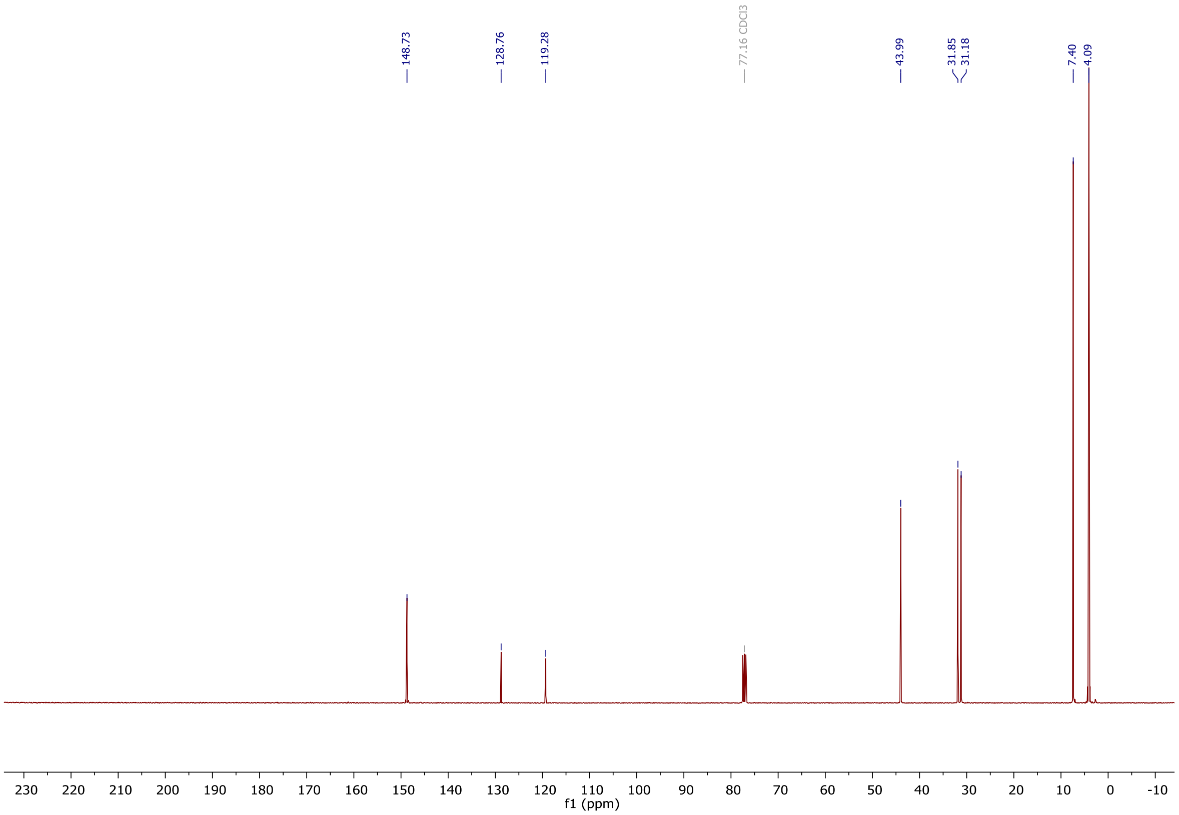

Figure S99 ^{13}C NMR spectrum of **3j** (101 MHz, $CDCl_3$).

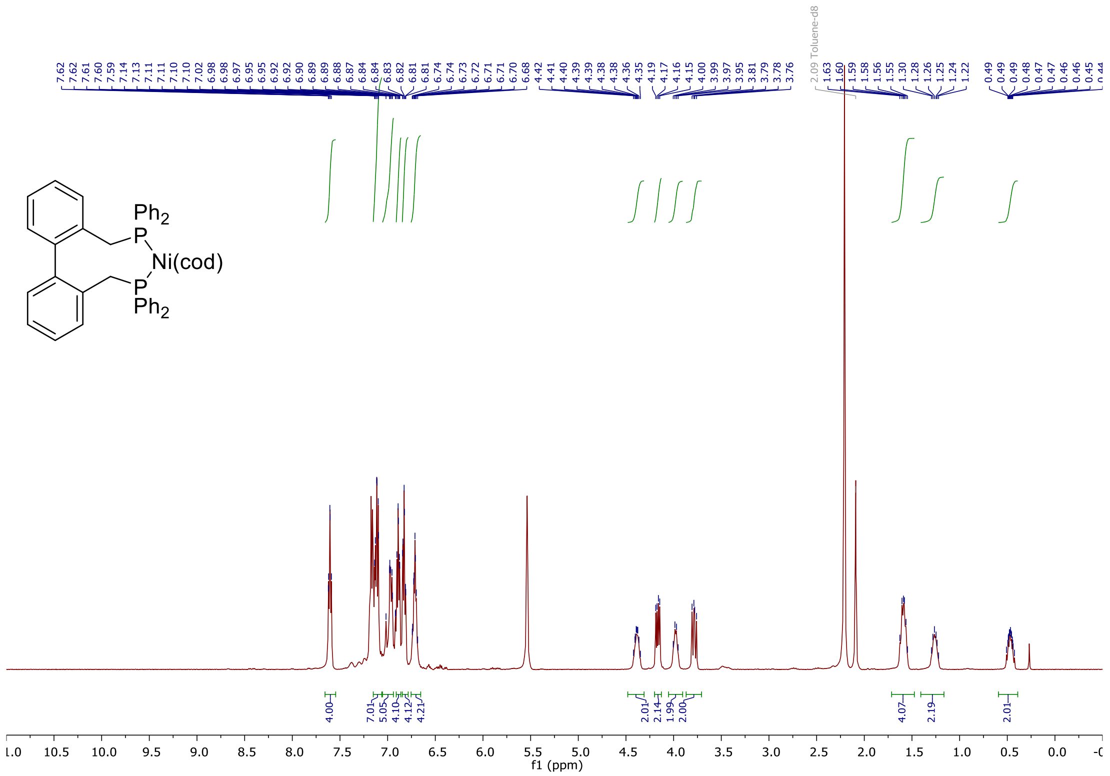

Figure S100 ^{1}H NMR spectrum of [(BISBI)Ni(cod)] (500 MHz, d_8-toluene).

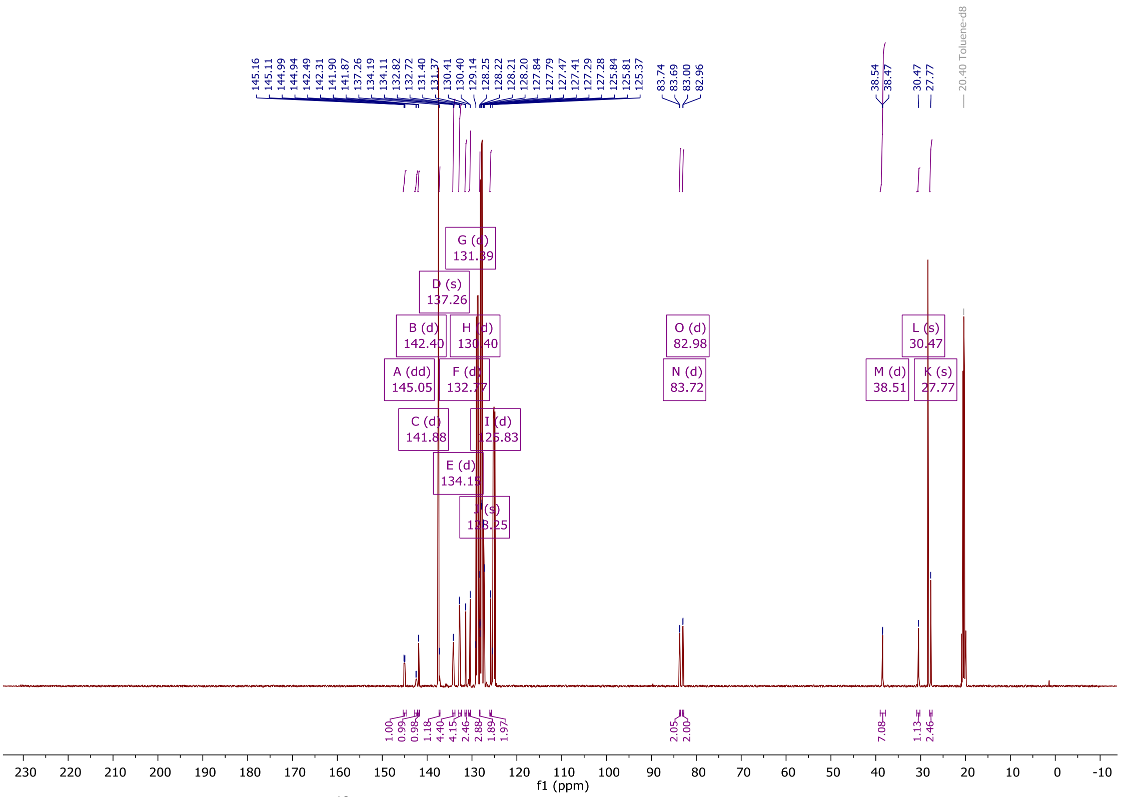

Figure S101 ^{13}C NMR spectrum of [(BISBI)Ni(cod)] (126 MHz, d_8-toluene).

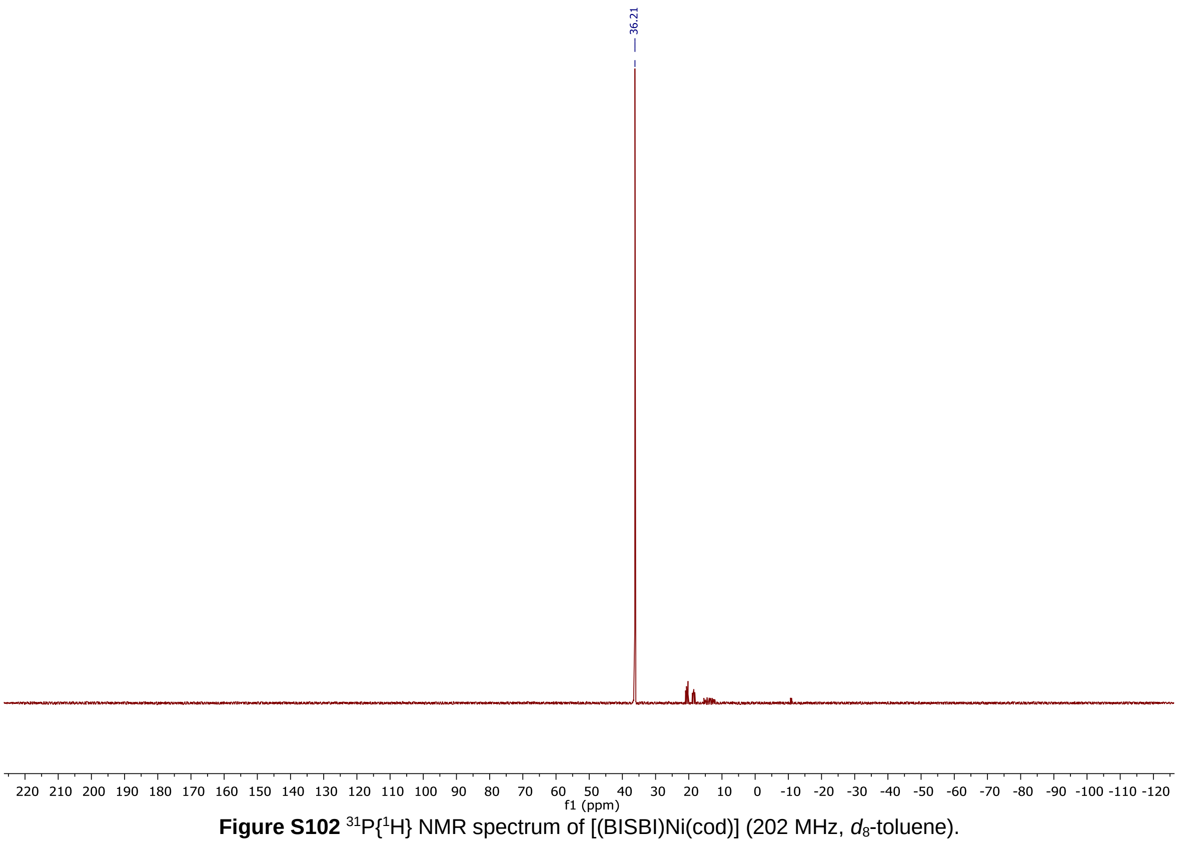

Figure S102 $^{31}P\{^{1}H\}$ NMR spectrum of [(BISBI)Ni(cod)] (202 MHz, d_8-toluene).

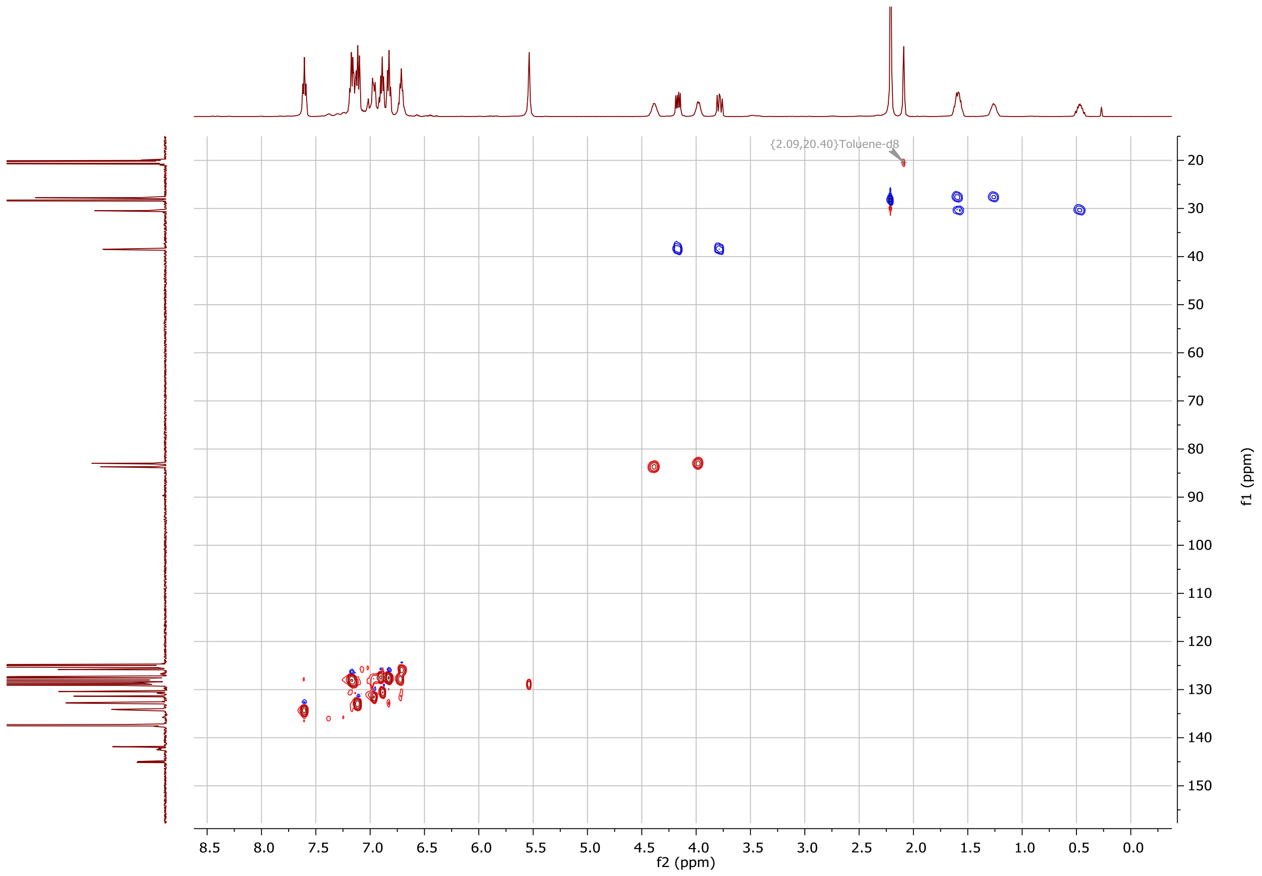

Figure S103 HSQC NMR spectrum of [(BISBI)Ni(cod)] (500 MHz, d_8-toluene).

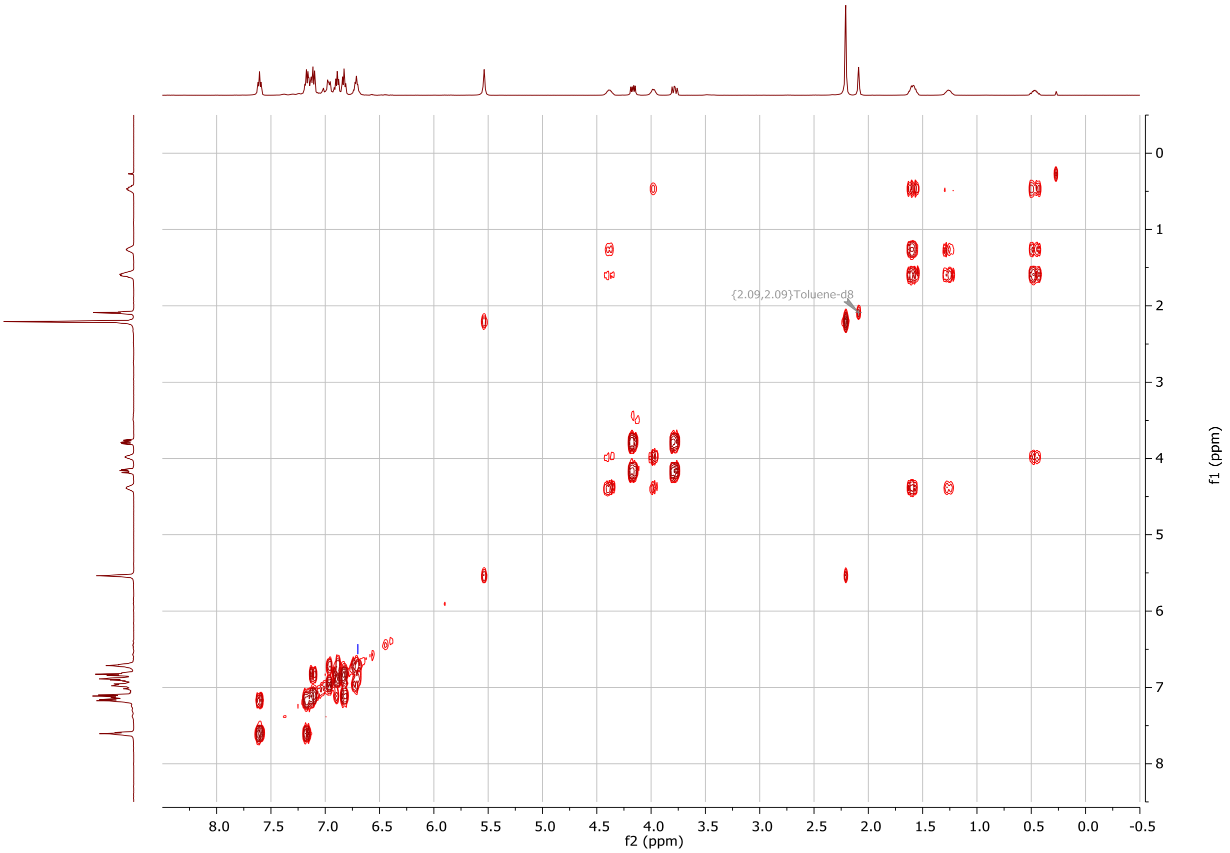

Figure S104 COSY NMR spectrum of [(BISBI)Ni(cod)] (500 MHz, d_8-toluene).

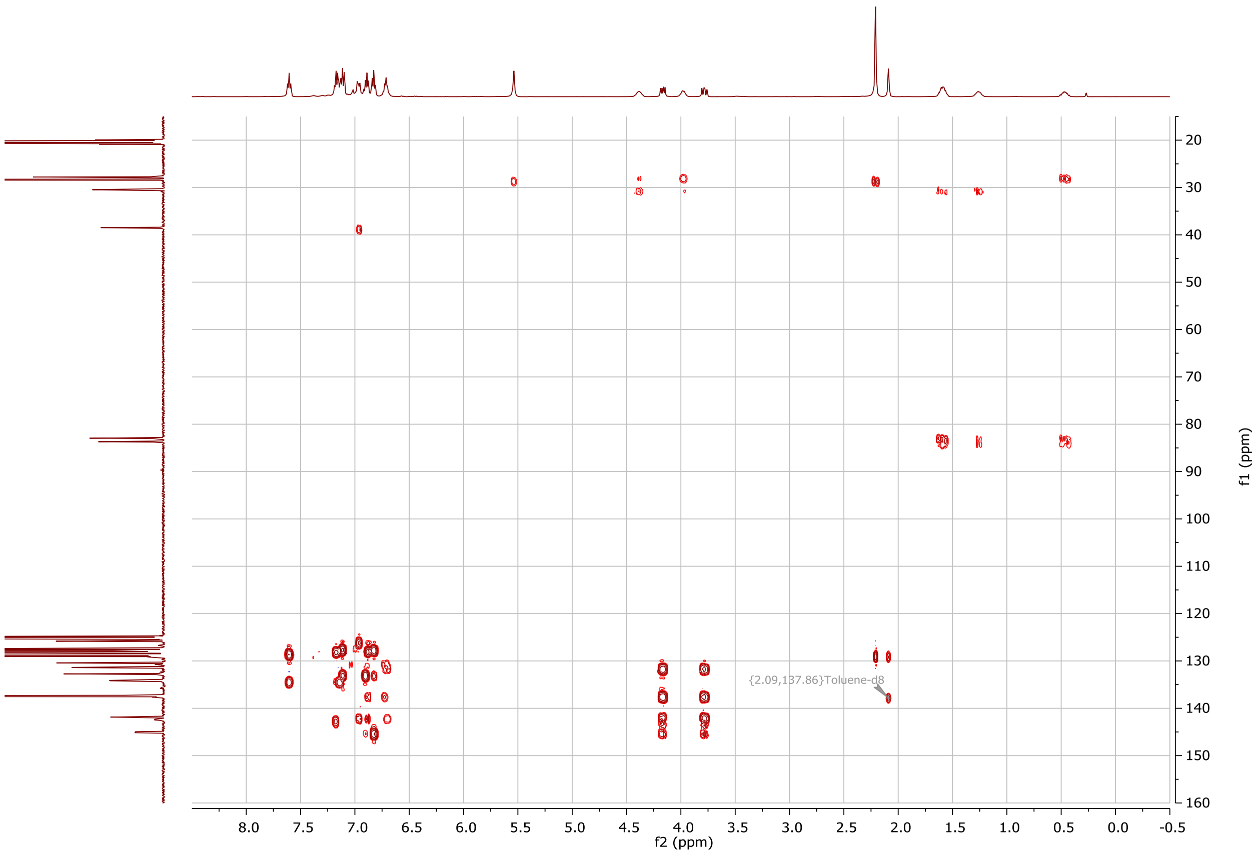

Figure S105 HMBC NMR spectrum of [(BISBI)Ni(cod)] (500 MHz, d_8-toluene).

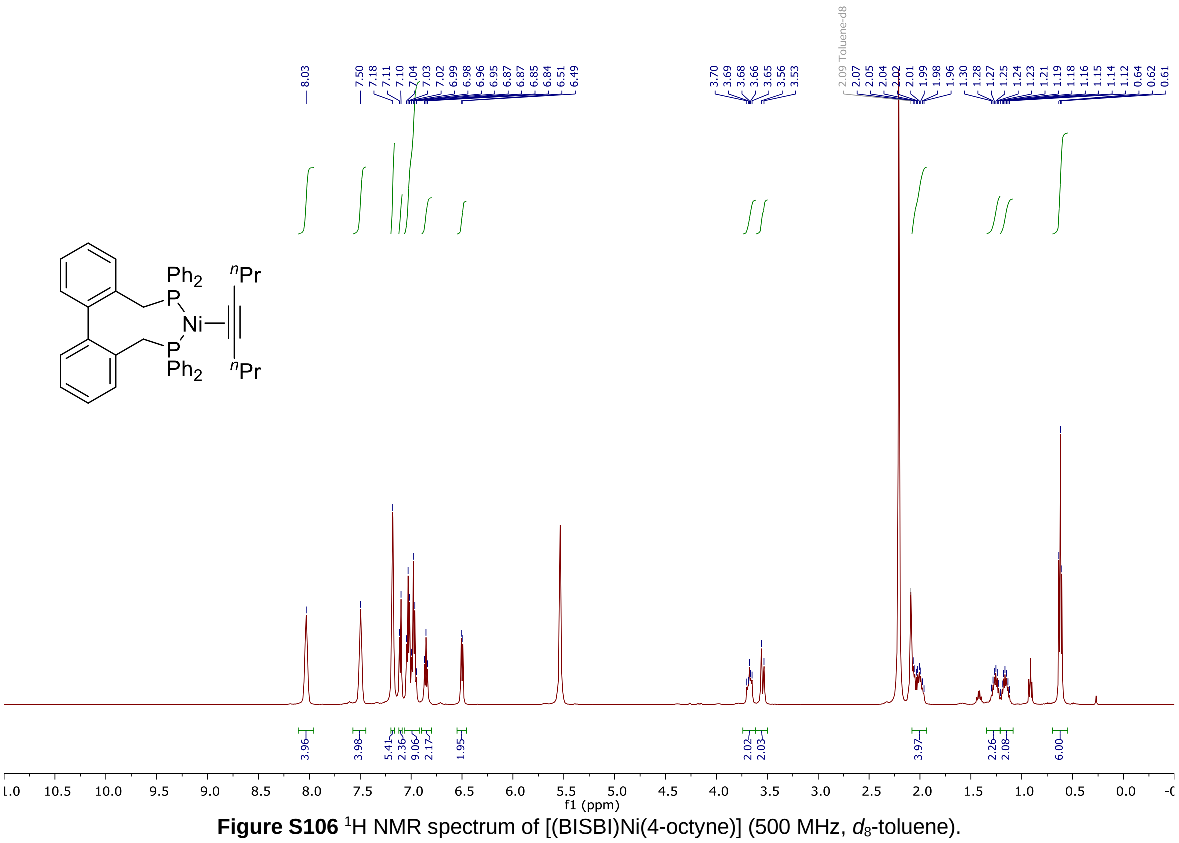

Figure S106 ^{1}H NMR spectrum of [(BISBI)Ni(4-octyne)] (500 MHz, d_8-toluene).

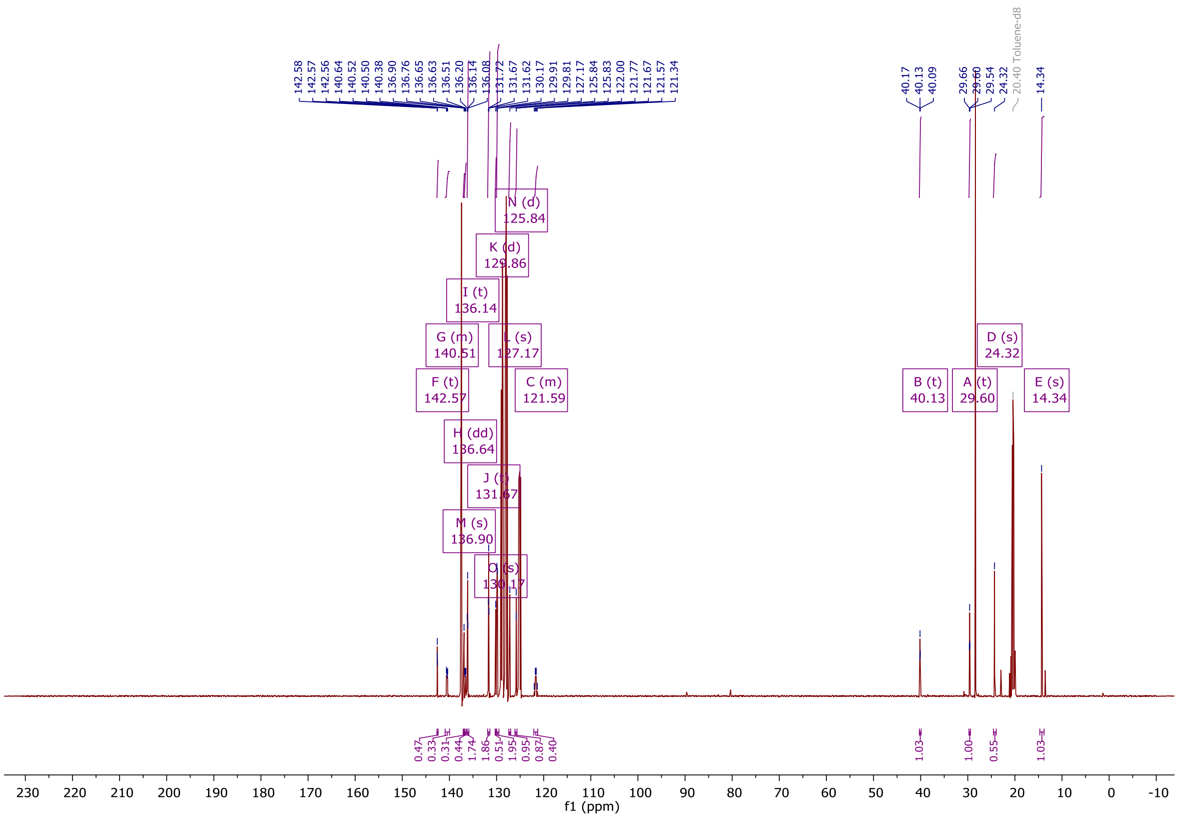

Figure S107 ^{13}C NMR spectrum of [(BISBI)Ni(4-octyne)] (126 MHz, d_8-toluene).

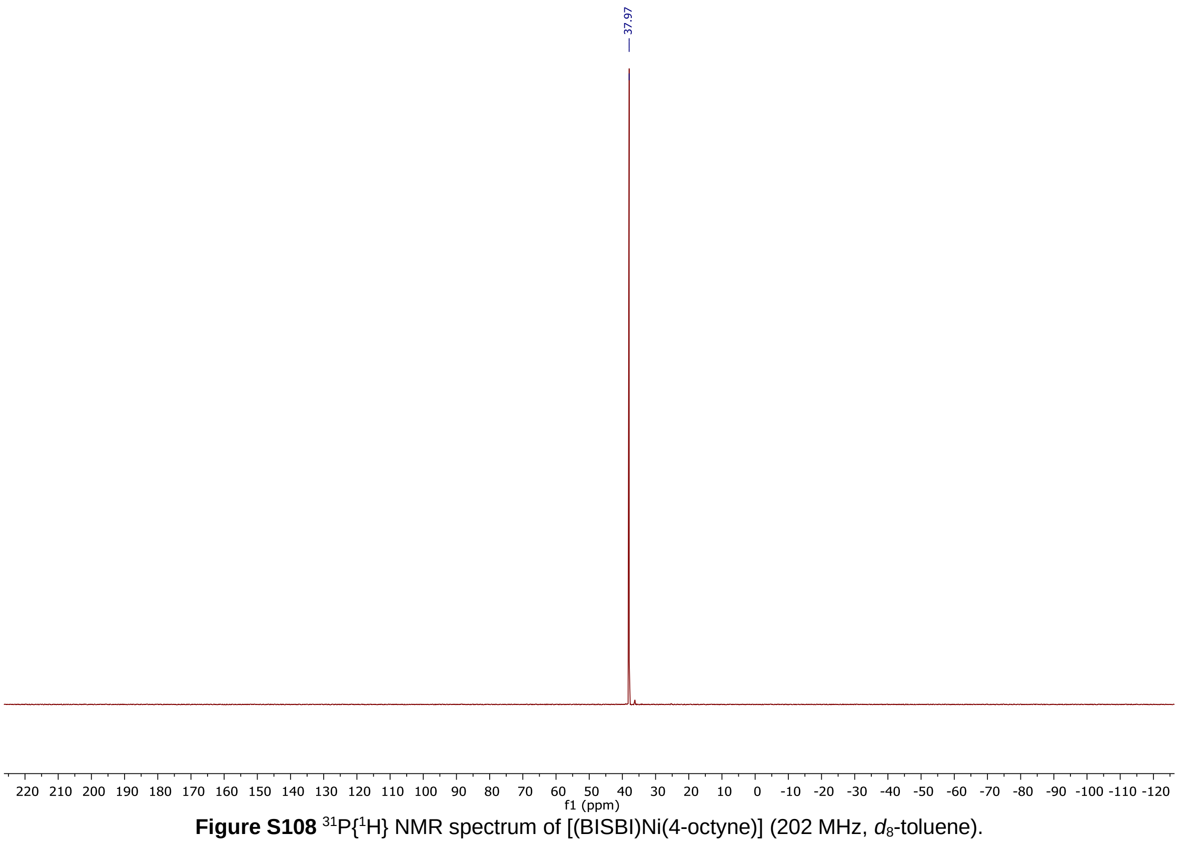

Figure S108 $^{31}P\{^{1}H\}$ NMR spectrum of [(BISBI)Ni(4-octyne)] (202 MHz, d_8-toluene).

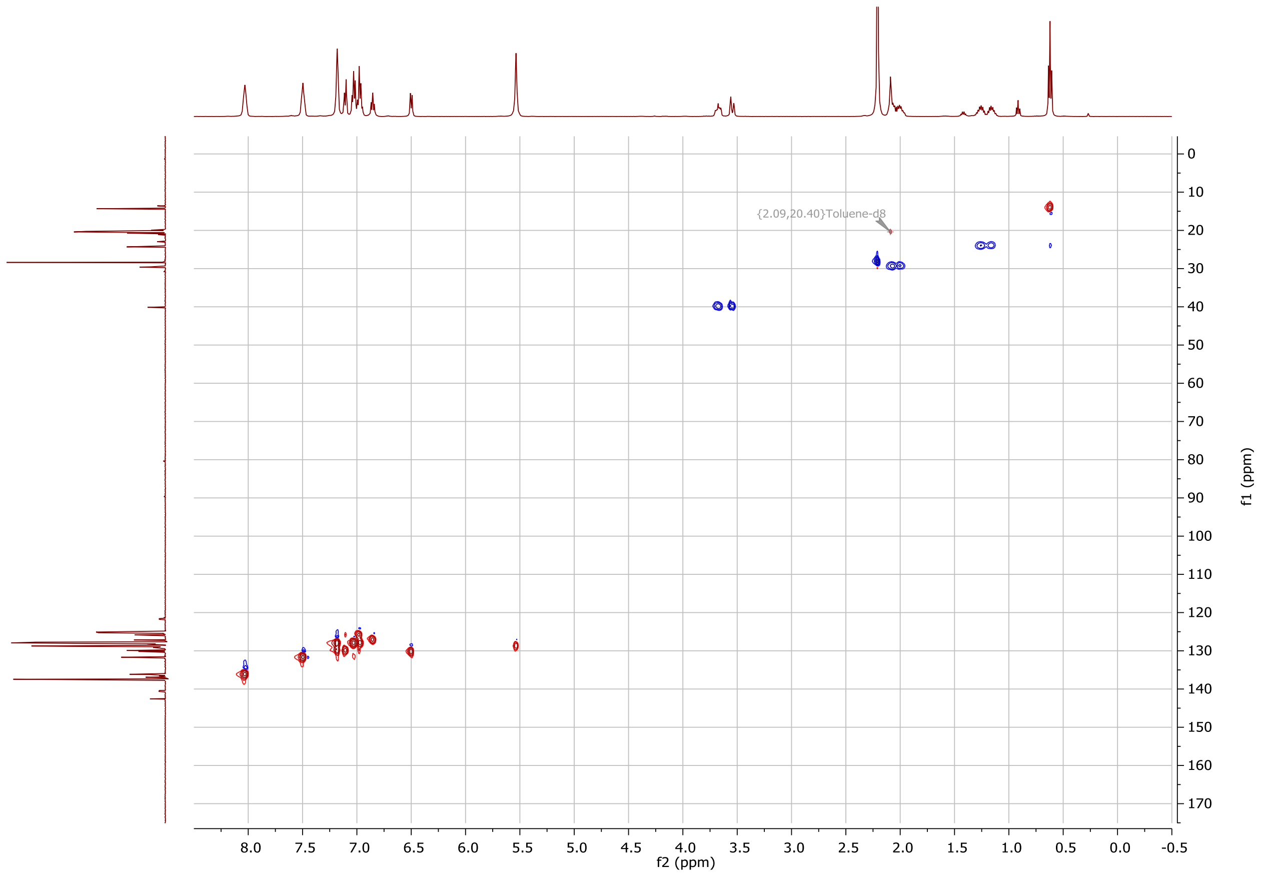

Figure S109 HSQC NMR spectrum of [(BISBI)Ni(4-octyne)] (500 MHz, d_8-toluene).

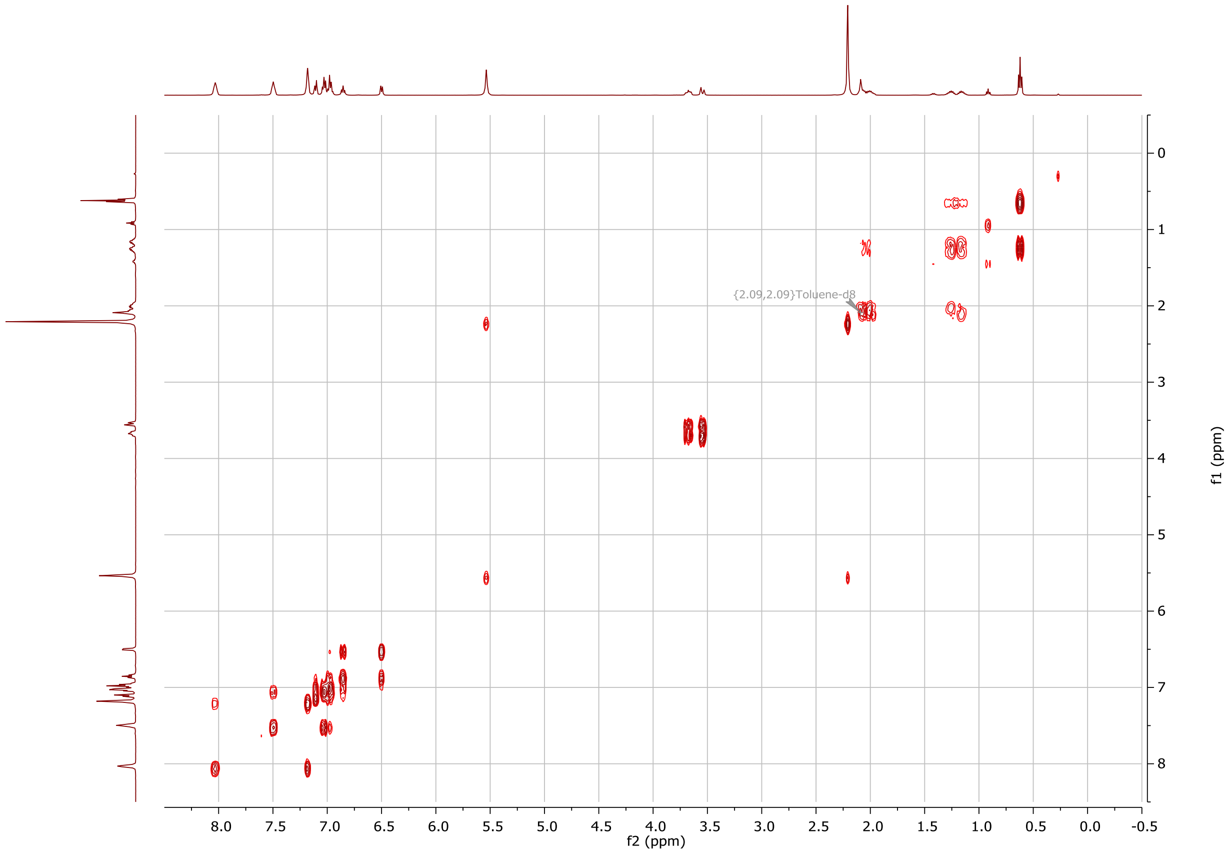

Figure S110 COSY NMR spectrum of [(BISBI)Ni(4-octyne)] (500 MHz, d_8-toluene).

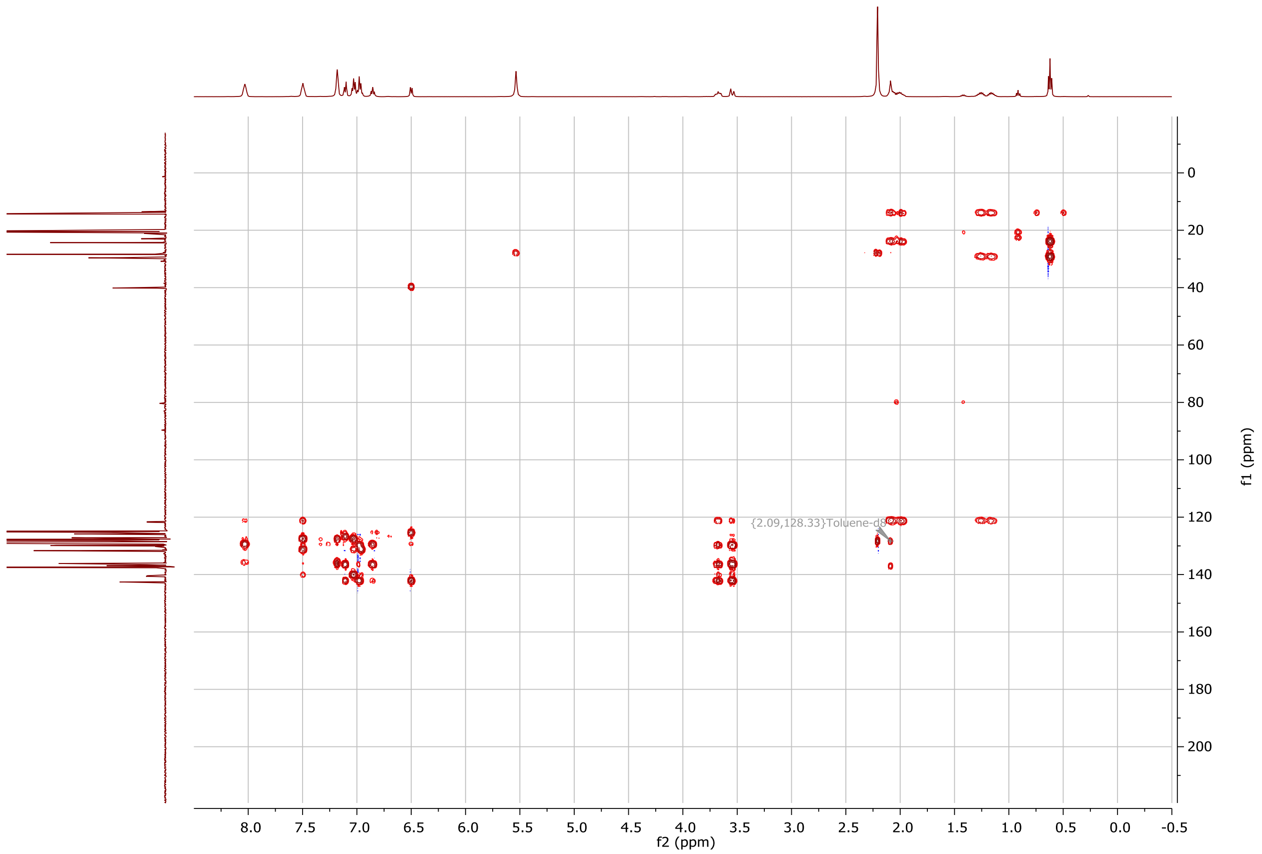

Figure S111 HMBC NMR spectrum of [(BISBI)Ni(4-octyne)] (500 MHz, d_8-toluene).

14. X-ray

a) Crystal data of [(BISBI)Ni(cod)] complex

Identification code	[(BISBI)Ni(cod)]
Empirical formula	$C_{46}H_{44}NiP_2$
Formula weight	717.46
Temperature/K	100.0(1)
Crystal system	monoclinic
Space group	C2/c
a/Å	27.5975(13)
b/Å	13.2663(4)
c/Å	19.5373(6)
α/°	90
β/°	97.063(4)
γ/°	90
Volume/Å^3	7098.7(5)
Z	8
ρ_{calc}g/cm^3	1.343
μ/mm^{-1}	1.880
F(000)	3024.0
Crystal size/mm^3	0.54 × 0.048 × 0.019
Radiation	Cu Kα (λ = 1.54184)
2Θ range for data collection/°	6.454 to 162.018
Index ranges	-35 ≤ h ≤ 33, -16 ≤ k ≤ 12, -24 ≤ l ≤ 24
Reflections collected	31435
Independent reflections	7533 [R_{int} = 0.0904, R_{sigma} = 0.0648]
Data/restraints/parameters	7533/0/442
Goodness-of-fit on F^2	1.044
Final R indexes [I>=2σ (I)]	R_1 = 0.0897, wR_2 = 0.2343
Final R indexes [all data]	R_1 = 0.1191, wR_2 = 0.2645
Largest diff. peak/hole / e Å^{-3}	2.14/-0.87

Table S19 Fractional Atomic Coordinates (×10^4) and Equivalent Isotropic Displacement Parameters (Å^2×10^3) for [(BISBI)Ni(cod)]. U_{eq} is defined as 1/3 of the trace of the orthogonalised U_{IJ} tensor.

Atom	*x*	*y*	*z*	U(eq)
Ni1	6075.7(3)	7034.1(6)	6644.6(4)	32.8(3)
P2	6334.0(5)	8541.3(8)	6957.0(6)	32.8(3)
P1	6468.8(5)	5923.4(8)	7322.0(6)	31.6(3)
C35	6052.8(19)	9299(3)	7606(2)	33.4(10)
C36	5651.1(19)	8945(3)	7898(2)	34.3(10)
C28	7568.7(18)	7440(3)	7777(2)	33.0(10)
C34	6992(2)	8780(4)	7249(3)	37.1(11)
C27	7643.4(19)	7106(3)	7066(2)	34.2(10)
C15	6522(2)	6066(3)	8269(2)	35.4(10)
C30	7769.3(19)	7312(4)	9014(3)	36.2(10)
C10	6265(2)	3965(4)	6755(3)	38.7(11)
C29	7833(2)	6991(4)	8355(2)	35.3(10)
C22	7391(2)	6277(4)	6760(2)	35.4(10)
C33	7241.0(19)	8212(3)	7862(2)	34.0(10)
C21	7107.4(19)	5578(3)	7170(3)	35.6(10)
C9	6190.3(19)	4643(3)	7282(3)	35.3(10)
C26	7941(2)	7684(4)	6685(3)	38.1(11)
C31	7433(2)	8069(4)	9103(2)	38.4(11)
C23	7433(2)	6071(4)	6070(3)	40.9(12)
C41	6273(2)	9473(4)	6244(3)	38.7(11)

Table S19 Fractional Atomic Coordinates (×10^4) and Equivalent Isotropic Displacement Parameters (Å^2×10^3) for [(BISBI)Ni(cod)]. U_{eq} is defined as 1/3 of the trace of the orthogonalised U_{IJ} tensor.

Atom	*x*	*y*	*z*	U(eq)
C40	6232(2)	10268(4)	7800(3)	38.9(11)
C32	7175(2)	8516(3)	8532(3)	35.8(10)
C37	5438(2)	9525(4)	8372(3)	40.3(11)
C39	6015(2)	10837(4)	8279(3)	38.2(11)
C24	7710(2)	6662(4)	5689(3)	42.4(12)
C2	6132(2)	7101(4)	5574(2)	40.3(12)
C6	5356.3(19)	6479(4)	6614(3)	39.5(11)
C38	5620(2)	10474(4)	8565(3)	41.0(12)
C42	5859(2)	10103(4)	6137(3)	42.3(12)
C14	5871(2)	4360(4)	7739(3)	41.9(12)
C5	5343(2)	7437(4)	6342(3)	42.2(12)
C25	7973(2)	7468(4)	5998(3)	43.1(12)
C8	5619(2)	5508(4)	5597(3)	43.3(12)
C20	6181(2)	6664(4)	8540(3)	46.1(13)
C7	5282(2)	5534(4)	6169(3)	44.0(12)
C1	6091(2)	6102(4)	5756(2)	38.2(11)
C11	6020(2)	3045(4)	6687(3)	44.8(13)
C16	6841(2)	5496(4)	8725(3)	43.7(12)
C19	6152(3)	6699(4)	9244(3)	54.3(16)
C3	5712(2)	7701(4)	5209(3)	48.1(14)
C46	6604(2)	9487(4)	5761(3)	44.2(13)
C4	5251(2)	7653(4)	5566(3)	50.0(15)
C45	6529(3)	10106(4)	5183(3)	49.4(14)
C13	5629(2)	3451(4)	7682(3)	49.3(14)
C43	5790(3)	10717(4)	5558(3)	52.5(15)
C12	5700(2)	2789(4)	7148(3)	51.5(14)
C44	6122(3)	10719(4)	5082(3)	55.8(17)
C17	6820(3)	5540(5)	9431(3)	55.1(16)
C18	6476(3)	6142(5)	9689(3)	52.6(15)

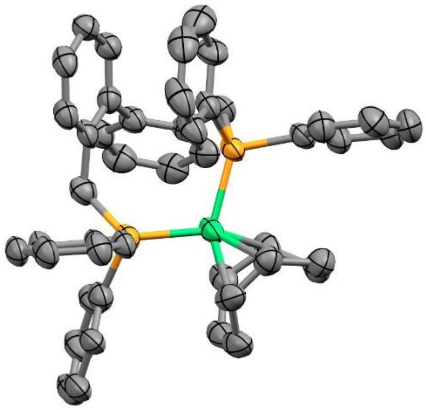

Figure S112: Asymmetric unit of the crystal structure of the [(BISBI)Ni(cod)] complex, ellipsoids depicted at 50% probability.

CCDC 2217066 contains the supplementary crystallographic data for this paper, including structure factors and refinement instructions. These data can be obtained free of charge from The Cambridge Crystallographic Data Centre, 12 Union Road, Cambridge CB2 1EZ, UK (fax: +44(1223)-336-033; e-mail: deposit@ccdc.cam.ac.uk), or via https://www.ccdc.cam.ac.uk/structures.

b) Crystal data of [(BISBI)Ni(4-octyne)] complex

Identification code	[(BISBI)Ni(4-octyne)]
Empirical formula	$C_{46}H_{46}NiP_2$
Formula weight	719.48
Temperature/K	100.0(1)
Crystal system	monoclinic
Space group	C2/c
a/Å	36.4758(2)
b/Å	12.82930(10)
c/Å	34.8171(2)
α/°	90
β/°	105.5920(10)
γ/°	90
Volume/Å^3	15693.39(19)
Z	16
ρ_{calc}g/cm^3	1.218
μ/mm^{-1}	1.701
F(000)	6080.0
Crystal size/mm^3	0.138 × 0.066 × 0.029
Radiation	Cu Kα (λ = 1.54184)
2Θ range for data collection/°	5.03 to 160.874
Index ranges	-46 ≤ h ≤ 46, -13 ≤ k ≤ 15, -43 ≤ l ≤ 44
Reflections collected	163296
Independent reflections	16854 [R_{int} = 0.0544, R_{sigma} = 0.0280]
Data/restraints/parameters	16854/87/962
Goodness-of-fit on F^2	1.046
Final R indexes [I>=2σ (I)]	R_1 = 0.0497, wR_2 = 0.1396
Final R indexes [all data]	R_1 = 0.0605, wR_2 = 0.1473
Largest diff. peak/hole / e Å^{-3}	1.90/-0.43

Table S20 Fractional Atomic Coordinates (×10^4) and Equivalent Isotropic Displacement Parameters (Å^2×10^3) [(BISBI)Ni(4-octyne)]. U_{eq} is defined as 1/3 of the trace of the orthogonalised U_{IJ} tensor.

Atom	*x*	*y*	*z*	U(eq)
Ni1A	4647.2(2)	7332.0(4)	6145.7(2)	31.34(11)
P1A	4805.1(2)	5901.9(5)	5913.1(2)	24.64(12)
P2A	4648.0(2)	8785.7(5)	5841.6(2)	38.98(16)
C1A	4502(3)	7708(6)	7524.2(19)	62(2)
C2A	4560.5(19)	8577(5)	7263.7(16)	49.9(15)
C3A	4351.8(16)	8458(4)	6822.8(15)	36.6(12)
C4A	4482.9(17)	7552(6)	6623.9(18)	24.1(13)
C5A	4563.5(15)	6604(5)	6626.4(16)	22.2(11)
C6A	4571.2(13)	5569(4)	6829.7(12)	26.3(9)
C7A	4176.4(14)	5183(4)	6826.6(16)	38.8(12)
C8A	4192.5(18)	4103(5)	7028(2)	53.0(16)
C9A	4911.8(6)	5721.9(19)	5420.0(6)	26.5(4)
C10A	4619.9(6)	6195.5(18)	5074.7(6)	25.1(4)
C11A	4271.6(6)	5686.1(19)	4922.3(6)	28.2(5)
C12A	3982.6(7)	6134(2)	4623.5(7)	32.5(5)
C13A	4043.8(8)	7082(2)	4464.7(7)	40.1(6)
C14A	4393.9(8)	7567(2)	4595.6(7)	38.7(6)
C15A	4686.2(7)	7140(2)	4903.2(6)	30.0(5)
C16A	5060.8(7)	7696(2)	5041.2(7)	33.9(5)
C17A	5377.8(8)	7323(2)	4934.3(8)	42.2(6)
C18A	5727.3(9)	7832(3)	5055.6(9)	52.3(8)
C19A	5763.3(9)	8714(3)	5292.5(9)	53.7(8)
C20A	5449.3(9)	9096(2)	5398.2(8)	47.6(7)
C21A	5093.8(8)	8611(2)	5271.9(7)	39.2(6)
C22A	4751.4(8)	9091(2)	5358.3(9)	46.3(7)
C23A	4427.0(6)	4923.9(18)	5881.3(6)	26.7(4)

Table S20 Fractional Atomic Coordinates (×10^4) and Equivalent Isotropic Displacement Parameters (Å^2×10^3) [(BISBI)Ni(4-octyne)]. U_{eq} is defined as 1/3 of the trace of the orthogonalised U_{IJ} tensor.

Atom	*x*	*y*	*z*	U(eq)
C24A	4050.7(6)	5252.9(19)	5743.4(7)	29.6(5)
C25A	3752.2(7)	4558(2)	5712.6(7)	34.3(5)
C26A	3824.8(7)	3533(2)	5823.3(8)	37.1(5)
C27A	4195.5(8)	3195(2)	5965.0(10)	46.0(7)
C28A	4495.7(7)	3883(2)	5994.5(8)	39.4(6)
C29A	5231.3(6)	5268(2)	6234.9(7)	32.5(5)
C30A	5385.5(8)	5693(2)	6612.3(8)	43.2(6)
C31A	5714.0(10)	5276(3)	6866.7(10)	61.4(9)
C32A	5889.0(9)	4426(3)	6747.7(10)	61.0(9)
C33A	5743.9(8)	4006(3)	6373.7(9)	47.6(7)
C34A	5418.2(7)	4427(2)	6116.9(8)	36.7(6)
C35A	4955.2(8)	9767(2)	6161.6(10)	51.2(8)
C36A	5206.9(8)	9429(3)	6510.4(11)	61.3(10)
C37A	5450.3(9)	10142(4)	6757.9(13)	76.8(13)
C38A	5442.2(10)	11171(4)	6660.1(14)	81.4(15)
C39A	5200.0(10)	11510(3)	6313.7(15)	75.5(13)
C40A	4954.7(9)	10820(3)	6058.4(13)	61.2(10)
C41A	4165.0(8)	9345(2)	5699.5(10)	49.9(7)
C42A	4044.8(9)	10198(2)	5876.9(14)	68.4(11)
C43A	3666.5(10)	10530(3)	5748.4(16)	77.5(13)
C44A	3414.4(9)	10030(3)	5439.7(14)	69.7(12)
C45A	3527.9(9)	9200(3)	5255.3(11)	59.4(9)
C46A	3898.8(9)	8849(3)	5387.0(10)	50.6(7)
Ni1C	2852.3(2)	5374.8(3)	3801.2(2)	23.93(10)
P1C	2879.6(2)	3972.0(4)	3467.8(2)	23.78(12)
P2C	2660.9(2)	6827.3(4)	3509.6(2)	23.09(12)
C1C	2666.0(11)	5985(3)	5128.1(11)	60.2(9)
C2C	3007.0(8)	6483(2)	5040.4(7)	40.4(6)
C3C	2952.3(7)	6731.5(19)	4598.7(6)	32.5(5)
C4C	2947.4(6)	5796.7(18)	4344.5(6)	27.3(5)
C5C	3029.3(7)	4834.1(18)	4330.9(6)	27.9(5)
C6C	3175.2(7)	3862.6(19)	4556.6(7)	32.0(5)
C7C	2883.9(9)	3314(2)	4724.0(8)	42.6(6)
C8C	3033.3(10)	2274(2)	4916.3(9)	49.6(7)
C9C	2693.4(7)	3766.6(18)	2919.2(6)	26.8(4)
C10C	2855.2(6)	4483.0(18)	2661.1(6)	26.9(5)
C11C	3215.9(7)	4290(2)	2611.3(6)	31.8(5)
C12C	3368.8(7)	4915(2)	2370.3(7)	35.9(6)
C13C	3162.7(7)	5759(2)	2174.2(7)	36.7(6)
C14C	2803.2(7)	5957(2)	2218.3(7)	33.7(5)
C15C	2646.3(6)	5333.4(19)	2460.7(6)	27.8(5)
C16C	2254.4(7)	5570.2(19)	2495.2(6)	28.6(5)
C17C	1949.9(7)	4964(2)	2282.0(7)	35.2(5)
C18C	1582.9(8)	5160(2)	2301.9(8)	43.4(6)
C19C	1514.4(7)	5980(3)	2532.3(8)	43.3(7)
C20C	1811.8(7)	6595(2)	2743.1(7)	35.0(5)
C21C	2186.5(6)	6399.9(19)	2730.5(6)	27.1(5)
C22C	2505.7(6)	7085.2(18)	2959.8(6)	26.8(4)
C23C	3353.1(6)	3372.6(18)	3571.3(6)	27.3(4)
C24C	3650.9(7)	3874(2)	3845.5(7)	30.6(5)
C25C	4013.9(7)	3448(2)	3951.3(8)	38.9(6)
C26C	4088.6(7)	2533(2)	3778.5(8)	39.8(6)
C27C	3799.5(8)	2036(2)	3499.5(8)	38.5(6)
C28C	3431.5(7)	2445(2)	3398.2(7)	33.1(5)
C29C	2586.0(7)	2976.7(18)	3625.2(6)	27.8(5)
C30C	2203.9(7)	3232(2)	3583.1(7)	34.2(5)

Table S20 Fractional Atomic Coordinates (×10[4]) and Equivalent Isotropic Displacement Parameters (Å[2]×10[3]) [(BISBI)Ni(4-octyne)]. U_{eq} is defined as 1/3 of the trace of the orthogonalised U_{IJ} tensor.

Atom	*x*	*y*	*z*	U(eq)
C31C	1964.4(8)	2553(2)	3705.6(8)	40.6(6)
C32C	2101.0(8)	1602(2)	3877.9(8)	41.1(6)
C33C	2475.9(8)	1337(2)	3919.3(8)	38.4(6)
C34C	2717.7(7)	2013.6(19)	3792.3(7)	32.2(5)
C35C	2254.8(6)	7405.0(18)	3649.2(6)	25.9(4)
C36C	2042.7(7)	6770(2)	3833.5(7)	32.9(5)
C37C	1707.1(8)	7122(2)	3901.0(8)	40.4(6)
C38C	1579.2(7)	8118(2)	3790.1(7)	36.9(5)
C39C	1789.4(7)	8772.2(19)	3614.5(7)	30.7(5)
C40C	2124.5(6)	8420.8(18)	3541.6(6)	26.5(4)
C41C	3034.9(6)	7834.1(18)	3637.8(6)	28.0(5)
C42C	3037.9(7)	8648.0(19)	3906.1(7)	33.9(5)
C43C	3335.3(8)	9374(2)	3994.2(9)	45.5(7)
C44C	3631.9(8)	9290(2)	3814.2(9)	46.3(7)
C45C	3634.1(7)	8478(2)	3554.9(8)	42.3(6)
C46C	3339.4(7)	7751(2)	3468.3(7)	32.8(5)
C2B	4235.4(18)	6382(5)	7142.0(16)	44.1(14)
C3B	4441.8(17)	6145(5)	6824.9(14)	33.0(12)
C4B	4501(2)	7064(6)	6591(2)	30.2(19)
C5B	4480(2)	8050(6)	6555.4(18)	37.3(15)
C1B	4448(2)	7090(5)	7471.7(16)	41.7(14)
C7B	4021(2)	9282(5)	6724(2)	56.3(16)
C6B	4430(2)	9097(6)	6731.9(19)	50.6(16)
C8B	3984(3)	10404(6)	6898(3)	90(3)

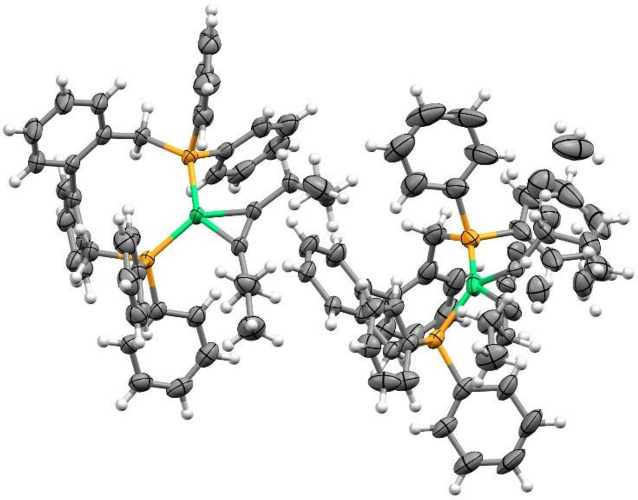

Figure S113 Asymmetric unit of the crystal structure of [(BISBI)Ni(4-octyne)] complex, ellipsoids depicted at 50% probability.

CCDC 2217081 contains the supplementary crystallographic data for this paper, including structure factors and refinement instructions. These data can be obtained free of charge from The Cambridge Crystallographic Data Centre, 12 Union Road, Cambridge CB2 1EZ, UK (fax: +44(1223)-336-033; e-mail: deposit@ccdc.cam.ac.uk), or via https://www.ccdc.cam.ac.uk/structures.

c) Crystal data of hexameric Ni-complex after reaction with 2-isopropylmalononitrile

Identification code	Hexameric Ni-complex after reaction with 2-isopropylmalononitrile
Empirical formula	$C_{303}H_{288}N_{12}Ni_{12}P_{12}$
Formula weight	5173.60

Temperature/K	100.0(1)
Crystal system	trigonal
Space group	R-3
a/Å	24.58740(10)
b/Å	24.58740(10)
c/Å	38.2223(2)
α/°	90
β/°	90
γ/°	120
Volume/Å^3	20011.17(19)
Z	3
ρ_{calc}g/cm^3	1.288
μ/mm^{-1}	1.997
F(000)	8118.0
Crystal size/mm^3	0.347 × 0.259 × 0.205
Radiation	Cu Kα (λ = 1.54184)
2Θ range for data collection/°	4.75 to 160.49
Index ranges	-31 ≤ h ≤ 31, -31 ≤ k ≤ 31, -48 ≤ l ≤ 48
Reflections collected	190383
Independent reflections	9676 [R_{int} = 0.0389, R_{sigma} = 0.0120]
Data/restraints/parameters	9676/532/653
Goodness-of-fit on F^2	1.051
Final R indexes [I>=2σ (I)]	R_1 = 0.0510, wR_2 = 0.1592
Final R indexes [all data]	R_1 = 0.0539, wR_2 = 0.1620
Largest diff. peak/hole / e Å^{-3}	1.07/-0.41

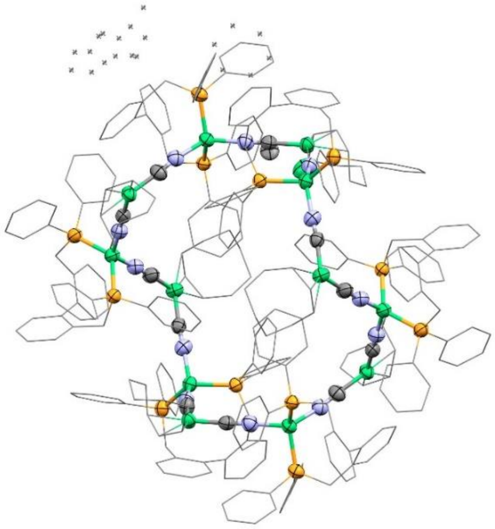

Figure S114 Asymmetric unit of the crystal structure of hexameric Ni-complex after reaction with 2-isopropylmalononitrile, ellipsoids depicted at 50% probability. H-atoms are omitted for clarity.

CCDC 2248846 contains the supplementary crystallographic data for this paper, including structure factors and refinement instructions. These data can be obtained free of charge from The Cambridge Crystallographic Data Centre, 12 Union Road, Cambridge CB2 1EZ, UK (fax: +44(1223)-336-033; e-mail: deposit@ccdc.cam.ac.uk), or via https://www.ccdc.cam.ac.uk/structures.

d) Crystal data of hexameric Ni-complex after reaction with 2-isocyano-2-(*p*-tolyl)acetonitrile

Identification code	Hexameric Ni-complex after reaction with 2-isocyano-2-(*p*-tolyl)acetontrile
Empirical formula	$C_{360}H_{378}N_{12}Ni_6O_{18}P_{12}$
Formula weight	5884.62

Temperature/K	100.0(1)
Crystal system	trigonal
Space group	R-3
a/Å	40.87200(10)
b/Å	40.87200(10)
c/Å	15.93560(10)
α/°	90
β/°	90
γ/°	120
Volume/Å^3	23054.23(18)
Z	3
ρ_{calc}g/cm^3	1.272
μ/mm^{-1}	1.500
F(000)	9342.0
Crystal size/mm^3	0.17 × 0.111 × 0.073
Radiation	Cu Kα (λ = 1.54184)
2Θ range for data collection/°	4.324 to 160.678
Index ranges	-52 ≤ h ≤ 52, -51 ≤ k ≤ 52, -19 ≤ l ≤ 17
Reflections collected	225948
Independent reflections	11087 [R_{int} = 0.0414, R_{sigma} = 0.0132]
Data/restraints/parameters	11087/297/661
Goodness-of-fit on F^2	1.029
Final R indexes [I>=2σ (I)]	R_1 = 0.0415, wR_2 = 0.1096
Final R indexes [all data]	R_1 = 0.0444, wR_2 = 0.1116
Largest diff. peak/hole / e Å^{-3}	1.05/-0.34

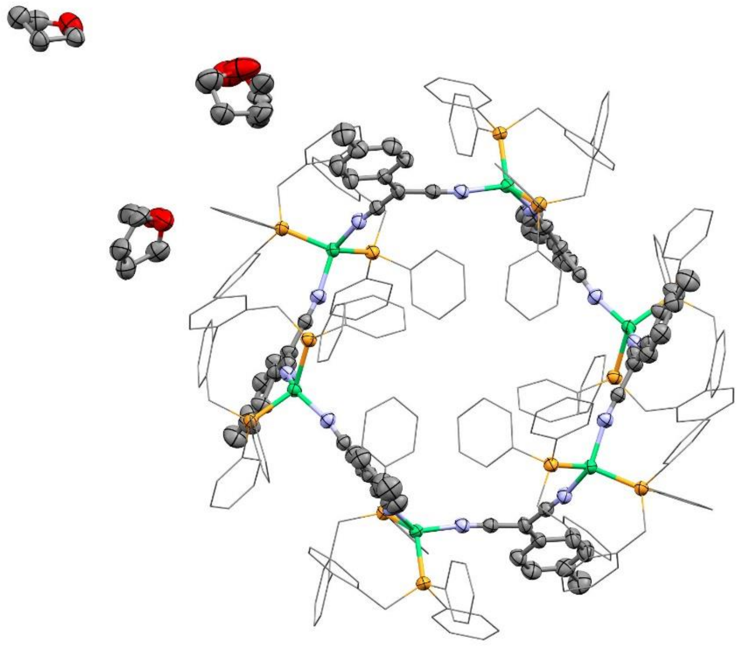

Figure S115 Asymmetric unit of the crystal structure of hexameric Ni-complex after reaction with 2-isocyano-2-(*p*-tolyl)acetonitrile, ellipsoids depicted at 50% probability. H-atoms are omitted for clarity.

CCDC 2248853 contains the supplementary crystallographic data for this paper, including structure factors and refinement instructions. These data can be obtained free of charge from The Cambridge Crystallographic Data Centre, 12 Union Road, Cambridge CB2 1EZ, UK (fax: +44(1223)-336-033; e-mail: deposit@ccdc.cam.ac.uk), or via https://www.ccdc.cam.ac.uk/structures.

15. References

(1) CrysAlisPro and ABSPACK. Rigaku Oxford Diffraction. **2016**.

(2) Sheldrick, G. M. SHELXT – Integrated Space-Group and Crystal-Structure Determination. *Acta Cryst.* **2015**, *A71*, 3–8. https://doi.org/10.1107/S2053273314026370.

(3) Sheldrick, G. M. A Short History of SHELX. *Acta Cryst.* **2008**, *A64*, 112–122. https://doi.org/10.1107/S0108767307043930.

(4) Sheldrick, G. M. Crystal Structure Refinement with SHELXL. *Acta Cryst.* **2015**, *C71*, 3–8. https://doi.org/10.1107/S2053229614024218.

(5) Dolomanov, O. V.; Bourhis, L. J.; Gildea, R. J.; Howard, J. A. K.; Puschmann, H. OLEX2: A Complete Structure Solution, Refinement and Analysis Program. *J. Appl. Cryst.* **2009**, *42*, 339–341. https://doi.org/10.1107/S0021889808042726.

(6) Bhawal, B. N.; Reisenbauer, J. C.; Ehinger, C.; Morandi, B. Overcoming Selectivity Issues in Reversible Catalysis: A Transfer Hydrocyanation Exhibiting High Kinetic Control. *J. Am. Chem. Soc.* **2020**, *142* (25), 10914–10920. https://doi.org/10.1021/jacs.0c03184.

(7) Weweler, J.; Younas, S. L.; Streuff, J. Titanium(III)-Catalyzed Reductive Decyanation of Geminal Dinitriles by a Non-Free-Radical Mechanism. *Angew. Chemie Int. Ed.* **2019**, *58* (49), 17700–17703. https://doi.org/10.1002/ANIE.201908372.

(8) Zhang, D.; Lian, M.; Liu, J.; Tang, S.; Liu, G.; Ma, C.; Meng, Q.; Peng, H.; Zhu, D. Preparation of O-Protected Cyanohydrins by Aerobic Oxidation of α-Substituted Malononitriles in the Presence of Diarylphosphine Oxides. *Org. Lett.* **2019**, *21* (8), 2597–2601. https://doi.org/10.1021/acs.orglett.9b00569.

(9) Reisenbauer, J.; Bhawal, B.; Jelmini, N.; Morandi, B. Development of an Operationally Simple, Scalable, and HCN-Free Transfer Hydrocyanation Protocol Using an Air-Stable Nickel Precatalyst. *Org. Process Res. Dev. 26* (4), 1165–1173. https://doi.org/10.1021/acs.oprd.1c00442.

(10) Frye, N. L.; Bhunia, A.; Studer, A. Nickel-Catalyzed Markovnikov Transfer Hydrocyanation in the Absence of Lewis Acid. *Org. Lett.* **2020**, *22* (11), 4456–4460. https://doi.org/10.1021/ACS.ORGLETT.0C01454.

(11) Bhunia, A.; Bergander, K.; Studer, A. Cooperative Palladium/Lewis Acid-Catalyzed Transfer Hydrocyanation of Alkenes and Alkynes Using 1-Methylcyclohexa-2,5-Diene-1-Carbonitrile. *J. Am. Chem. Soc.* **2018**, *140* (47), 16353–16359. https://doi.org/10.1021/jacs.8b10651.

(12) Blackmond, D. G. Kinetic Profiling of Catalytic Organic Reactions as a Mechanistic Tool. *J. Am. Chem. Soc.* **2015**, *137* (34), 10852–10866. https://doi.org/10.1021/jacs.5b05841.

(13) Blackmond, D. G. Reaction Progress Kinetic Analysis: A Powerful Methodology for Mechanistic Studies of Complex Catalytic Reactions. *Angew. Chemie Int. Ed.* **2005**, *44*, 4302–4320. https://doi.org/10.1002/ANIE.200462544.

(14) Martínez-Carrión, A.; Howlett, M. G.; Alamillo-Ferrer, C.; Clayton, A. D.; Bourne, R. A.; Codina, A.; Vidal-Ferran, A.; Adams, R. W.; Burés, J. Kinetic Treatments for Catalyst Activation and Deactivation Processes Based on Variable Time Normalization Analysis. *Angew. Chemie Int. Ed.* **2019**, *58*, 10189–10193. https://doi.org/10.1002/ANGE.201903878.

(15) J. Shiner, V.; S. Humphrey, J. The Effects of Deuterium Substitution on the Rates of Organic Reactions. IX. Bridgehead β-Deuterium in a Carbonium Ion Solvolysis. *J. Am. Chem. Soc.* **2002**, *85* (16), 2416–2419. https://doi.org/10.1021/ja00899a017.

(16) Berger, S.; Diehl, B. W. K.; Künzer, H. Deuterium Isotope Effects as a Probe for C–C Hyperconjugation. *Chem. Ber.* **1987**, *120* (6), 1059–1062. https://doi.org/10.1002/cber.19871200628.

(17) Singleton, D. A.; Thomas, A. A. High-Precision Simultaneous Determination of Multiple Small Kinetic Isotope Effects at Natural Abundance. *J. Am. Chem. Soc.* **1995**, *117* (36), 9357–9358. https://doi.org/10.1021/ja00141a030.

(18) *Chemcraft - Citation*. https://www.chemcraftprog.com/citation.html (accessed 2022-04-28).

(19) Pracht, P.; Bohle, F.; Grimme, S. Automated Exploration of the Low-Energy Chemical Space with Fast Quantum Chemical Methods. *Phys. Chem. Chem. Phys.* **2020**, *22* (14), 7169–7192. https://doi.org/10.1039/C9CP06869D.

(20) Grimme, S. Exploration of Chemical Compound, Conformer, and Reaction Space with Meta-Dynamics Simulations Based on Tight-Binding Quantum Chemical Calculations. *J. Chem. Theory Comput.* **2019**, *15* (5), 2847–2862. https://doi.org/10.1021/acs.jctc.9b00143.

(21) Bannwarth, C.; Ehlert, S.; Grimme, S. GFN2-XTB—An Accurate and Broadly Parametrized Self-Consistent Tight-Binding Quantum Chemical Method with Multipole Electrostatics and Density-Dependent Dispersion Contributions. *J. Chem. Theory Comput.* **2019**, *15* (3), 1652–1671. https://doi.org/10.1021/acs.jctc.8b01176.

(22) Bannwarth, C.; Caldeweyher, E.; Ehlert, S.; Hansen, A.; Pracht, P.; Seibert, J.; Spicher, S.; Grimme, S. Extended Tight-Binding Quantum Chemistry Methods. *Wiley Interdiscip. Rev. Comput. Mol. Sci.* **2021**, *11* (2), e1493. https://doi.org/10.1002/WCMS.1493.

(23) Neese, F. Software Update: The ORCA Program System—Version 5.0. *Wiley Interdiscip. Rev. Comput. Mol. Sci.* **2022**, *12* (5), e1606. https://doi.org/10.1002/WCMS.1606.

(24) Adamo, C.; Barone, V. Toward Reliable Density Functional Methods without Adjustable Parameters: The PBE0 Model. *J. Chem. Phys.* **1999**, *110* (13), 6158–6170. https://doi.org/10.1063/1.478522.

(25) Weigend, F.; Ahlrichs, R. Balanced Basis Sets of Split Valence, Triple Zeta Valence and Quadruple Zeta Valence Quality for H to Rn: Design and Assessment of Accuracy. *Phys. Chem. Chem. Phys.* **2005**, *7* (18), 3297–3305. https://doi.org/10.1039/B508541A.

(26) Grimme, S.; Ehrlich, S.; Goerigk, L. Effect of the Damping Function in Dispersion Corrected Density Functional Theory. *J. Comput. Chem.* **2011**, *32* (7), 1456–1465. https://doi.org/10.1002/JCC.21759.

(27) Grimme, S.; Antony, J.; Ehrlich, S.; Krieg, H. A Consistent and Accurate Ab Initio Parametrization of Density Functional Dispersion Correction (DFT-D) for the 94 Elements H-Pu. *J. Chem. Phys.* **2010**, *132* (15), 154104. https://doi.org/10.1063/1.3382344.

(28) Neese, F.; Wennmohs, F.; Hansen, A.; Becker, U. Efficient, Approximate and Parallel Hartree–Fock and Hybrid DFT Calculations. A 'Chain-of-Spheres' Algorithm for the Hartree–Fock Exchange. *Chem. Phys.* **2009**, *356* (1), 98–109. https://doi.org/https://doi.org/10.1016/j.chemphys.2008.10.036.

(29) Weigend, F. Accurate Coulomb-Fitting Basis Sets for H to Rn. *Phys. Chem. Chem. Phys.* **2006**, *8* (9), 1057–1065. https://doi.org/10.1039/B515623H.

(30) Leugault C. Y. (Université de Sherbrooke). *CYLview20*. http://www.cylview.org.

(31) Anderson, T. L.; Kwan, E. E. *PyQuiver*. www.github.com/ekwan/PyQuiver.
